# Exploring shared pathways and the shared biomarker ERRFI1 in Obstructive sleep apnoea and atherosclerosis using integrated bioinformatics analysis

**DOI:** 10.1038/s41598-023-42184-0

**Published:** 2023-09-12

**Authors:** Bowen Chen, Liping Dong, Jihua Zhang, Ying Hao, Weiwei Chi, Dongmei Song

**Affiliations:** 1 Clinical Biobank, The First Hospital of Hebei Medical University, Shijiazhuang, China; 2 Department of Otolaryngology, The First Hospital of Hebei Medical University, Shijiazhuang, China

**Keywords:** Biomarkers, Cardiology, Diseases, Pathogenesis

## Abstract

Obstructive sleep apnea (OSA) is an upper airway disorder occurring during sleep and is associated with atherosclerosis (AS). AS is a cardiovascular disease caused by environmental and genetic factors, with a high global mortality rate. This study investigated common pathways and potential biomarkers of OSA and AS. Microarray data were downloaded from the Gene Expression Omnibus (GEO) database and used to screen for differentially expressed genes (DEGs) in the OSA and AS datasets. A weighted gene co-expression network analysis (WGCNA) was used to identify the co-expression modules of OSA and AS. The least absolute shrinkage and selection operators (LASSO) were used to determine critical biomarkers. Immune cell infiltration analysis was used to investigate the correlation between immune cell infiltration and common biomarkers of OSA and AS. Results revealed that differentially expressed genes may be involved in inflammatory processes, chemokine signaling pathways, and molecular changes in cell adhesion. ERBB receptor feedback inhibitor 1 (ERRFI1) was the best-shared biomarker for OSA and AS. Immune infiltration analysis showed that ERRFI1 expression was correlated with immune cell changes. Changes in immune pathways, inflammatory processes, and cell adhesion molecules may underlie the pathogenesis of both diseases, and ERRFI1 may be a potential diagnostic marker for patients with OSA and AS.

OSA is a major public health problem affecting 5–20% of the general population, with a worldwide prevalence of approximately 100 million individuals aged 30–69 years^1^. OSA is a condition of apnea and hypoventilation caused by the collapse of the upper airway during sleep, accompanied by snoring, disturbed sleep architecture, decreased oxygen saturation, and daytime sleepiness^2^. Long-term OSA can lead to cardiovascular diseases, diabetes, depression, motor vehicle accidents, and workplace accidents, resulting in a considerable burden on the global healthcare system and economic burden^3^.

Atherosclerosis is a chronic inflammation of blood vessels triggered by a complex interaction of risk factors and arterial wall cells. Previous studies have shown that OSA can lead to atherosclerosis (AS). In a series of prospective studies, the incidence of coronary artery disease was found to be higher in patients with OSA (16.2%) than in those without OSA (5.4%)^4^. And the prevalence of atherosclerosis increases significantly with the severity of OSA, up to 42% in patients with moderate to severe OSA^5^. Recent evidence also suggests that individuals at high risk for OSA are more likely to develop coronary plaque^6^. OSA can cause atherosclerosis and can induce AS through the activation of inflammatory pathways including hypoxia-inducible factor, nuclear factor kappa-light-chain-enhancer of activated B cells pathway, Toll-like receptor 4 (TLR4), adhesion molecules, and tumor necrosis factor^7^. It also contributes to the development of AS by causing abnormal platelet aggregation^8^. Oxidative stress^9^ and abnormal glucolipid metabolism mediate the development of AS^10^. Previous studies have shown that a high-cholesterol diet (HCD) is the traditional induction modality for inducing AS in mice^11^. Notably, OSA and a high-cholesterol diet (HCD) may cause AS by distinct mechanisms^12^. The main pathological process of OSA is chronic intermittent hypoxia (CIH)^13^. Previous studies have reported that CIH is a weaker inducer of AS8 and there are important differences between CIH- and HCD-induced activation of inflammatory pathways. Histologically, CIH-induced atherosclerotic plaques did not have a necrotic core, whereas HCD-induced plaques have a typical necrotic core and a fibrous cap. Furthermore, HCD leads to the formation of macrophage foam cells, but CIH does not. These findings reflect the inherent differences in pathology and underlying mechanisms between the two types of atherosclerosis in terms of the nature of the pathology and underlying mechanisms. OSA and AS are closely related and mechanistically different, so it is important to study the mechanisms of the two diseases for early intervention and treatment. However, there is a paucity of research into the similarities and differences between the two diseases and further exploration is needed.

In this study, we collected data from the GEO database, then screened for differential genes, performed enrichment analysis, WGCNA analysis, Gene Set Enrichment Analysis (GSEA) analysis^14^, and LASSO^15^ regression models to screen for shared pathways and key biomarkers for OSA and AS, and analyzed the expression levels and diagnostic value of the genes, validated the dataset for validation, and examined monocytes from blood samples collected from patients. By these similar methods, Zhang et al.^16^ found that the MAPK signaling pathway may be associated with both pathogenesis of ankylosing spondylitis and ulcerative colitis and that poly(A) specific ribonuclease subunit PAN3 Gene (PAN3) may be a potential diagnostic marker for patients with ulcerative colitis complicated by ankylosing spondylitis. Similarly, Gao^17^ used these similar approaches to find that immune responses may be associated with both epilepsy and subarachnoid hemorrhage pathogenesis and that Matrix metalloproteinase-9 (MMP9) and Complement C3a Receptor 1 (C3aR1) may be potential diagnostic markers for subarachnoid hemorrhage complicated by epilepsy. Using these methods, we aim to explore possible shared pathways of action between OSA and AS and identify new possible diagnostic and therapeutic strategies for patients with AS secondary to OSA (Fig. 1).

**Figure 1:**
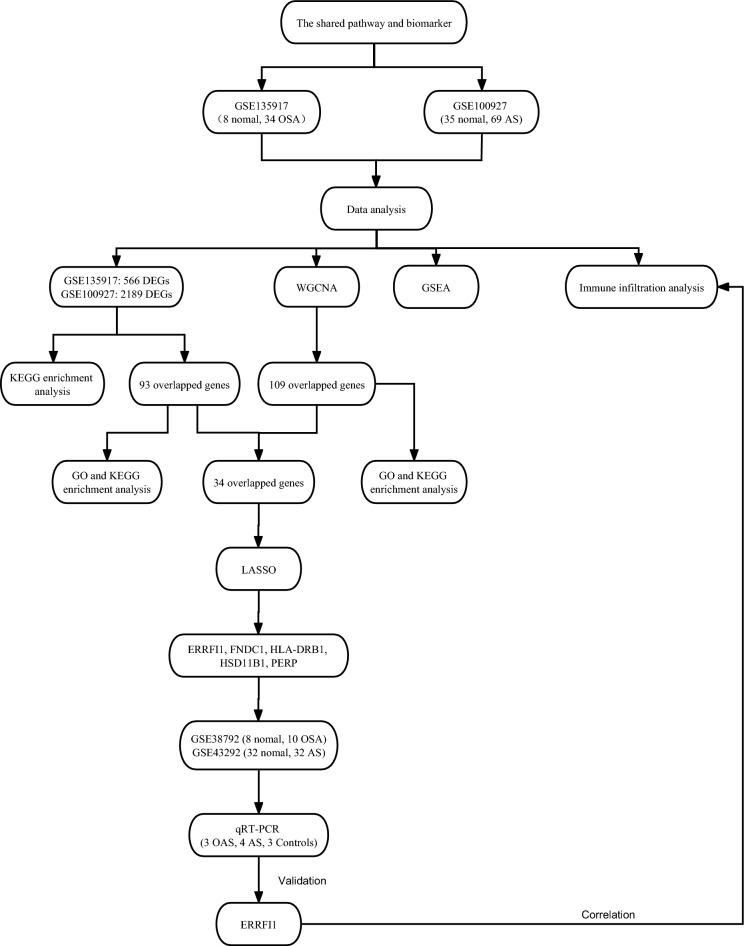
Schematic presentation of the analysis process.

## Results

### Differential genetic screening

**Figure 2:**
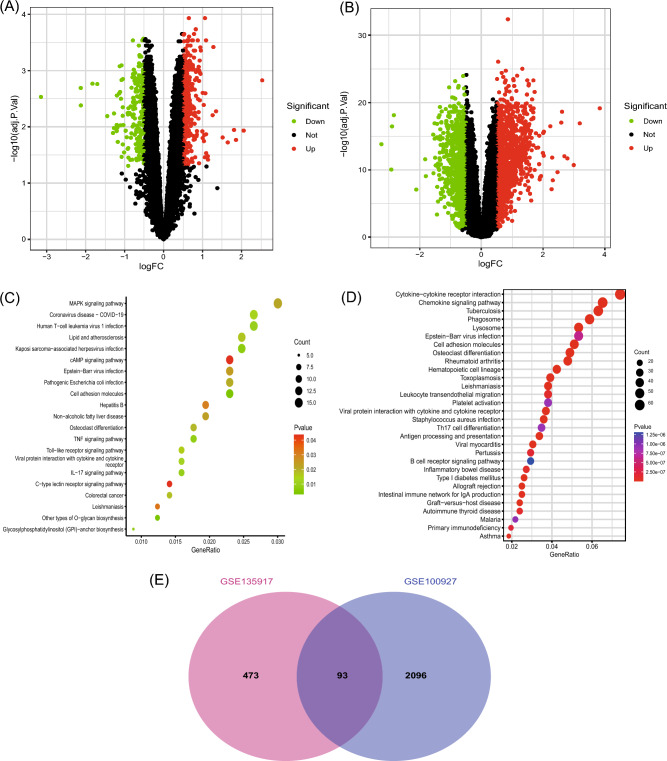
Differential genetic screening. (**A**) The volcano plots of DEGs in GSE135917 (*n* = 566, *p* < 0.05). (**B**) The volcano plots of DEGs in GSE100927 (*n* = 2189, *p* < 0.05). (**C**) The KEGG analysis of DEGs in GSE135917. (**D**) The KEGG analysis of DEGs in GSE100927. (**E**) Venn diagram of DEGs from GSE100927 and GSE135917.

**Figure 3:**
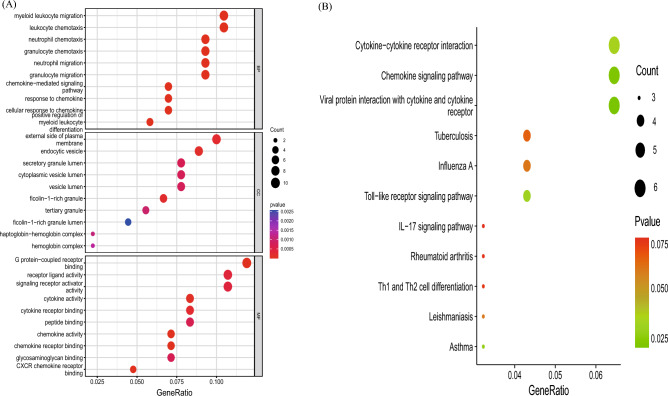
(**A**) The GO analysis of 93 genes. (**B**) The KEGG analysis of 93 genes.

A total of 566 DEGs were identified in patients with OSA and normal controls, including 369 upregulated and 197 downregulated genes, as shown in the volcano plot (Fig. 2A). A total of 2189 DEGs (see Supplementary Table S1 online) of patients with AS and normal controls were screened, identifying 1256 up-regulated and 933 downregulated genes, as shown in the volcano plot (Fig. 2B). The DEGs were analyzed using the Kyoto Encyclopedia of Gene and Genome (KEGG, https://www.genome.jp/kegg/) pathway enrichment analysis. KEGG analysis showed that both OSA and AS were enriched in the cell adhesion molecule pathways (Fig. 2C,D, Supplementary Table S2 online). The two GEGs were taken to intersect to obtain differentially expressed genes common to AS and OSA, with a total of 93 genes (Fig. 2E, Supplementary Table S3 online). The co-expressed differentially expressed genes were subjected to KEGG and Gene Ontology (GO) analysis (see Supplementary Table S4 online), and GO analysis (Fig. 3A) showed that a series of important processes were involved, myeloid leukocyte migration, leukocyte chemotaxis, and response to chemokine, etc., and in terms of KEGG (Fig. 3B), several important pathways are involved, such as Cytokine-cytokine receptor interaction, Chemokine signaling pathway, and these results strongly suggest that inflammatory processes are associated with both OSA and AS are related.

### Construction and module analysis of weighted gene co-expression network analysis (WGCNA)

**Figure 4:**
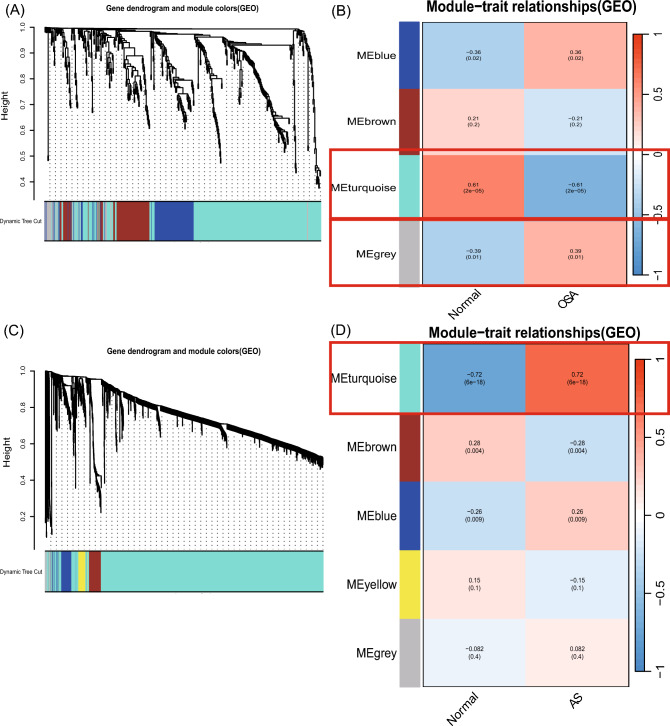
Construction and module analysis of weighted gene co-expression network (WGCNA). (**A**) The cluster dendrogram of co-expression in OSA. (**B**) Correlation between modules and clinical traits in OSA. (**C**) The cluster dendrogram of co-expression in AS. (**D**) Correlation between modules and clinical traits in AS.

WGCNA was used to identify differentially expressed co-expressed clusters of genes between OSA and AS, and to calculate the correlation between the combined modules and disease characterization. Two datasets, GSE135917 and GSE100927, were used for the WGCNA analysis (see Supplementary Table S5 online). Outliers were checked using sample clustering, and no samples were removed from either GSE135917 or GSE100927 (see Supplementary Figs. S1, S2 online). According to the approximate scale-free topology criterion, *β* = 6 was chosen to determine the soft threshold in the OSA model, and *β* = 5 was chosen to determine the soft threshold in the AS model (see Supplementary Figs. S3, S4 online). Cluster dendrogram of the co-expression of AS and OSA (Fig. 4A,C). After merging similar gene modules, four modules (Fig. 4B) were identified in the OSA model and five modules (Fig. 4D) were identified in the AS model. Among the OSA modules, the gray module had the strongest positive correlation with OSA (*R* = 0.39), and the turquoise module had the strongest negative correlation with the occurrence of OSA (*R* =  − 0.61). The turquoise module positively correlated with the occurrence of AS (*R* = 0.72) (see Supplementary Table S6 online).

### Identifying shared genes and shared pathways

**Figure 5:**
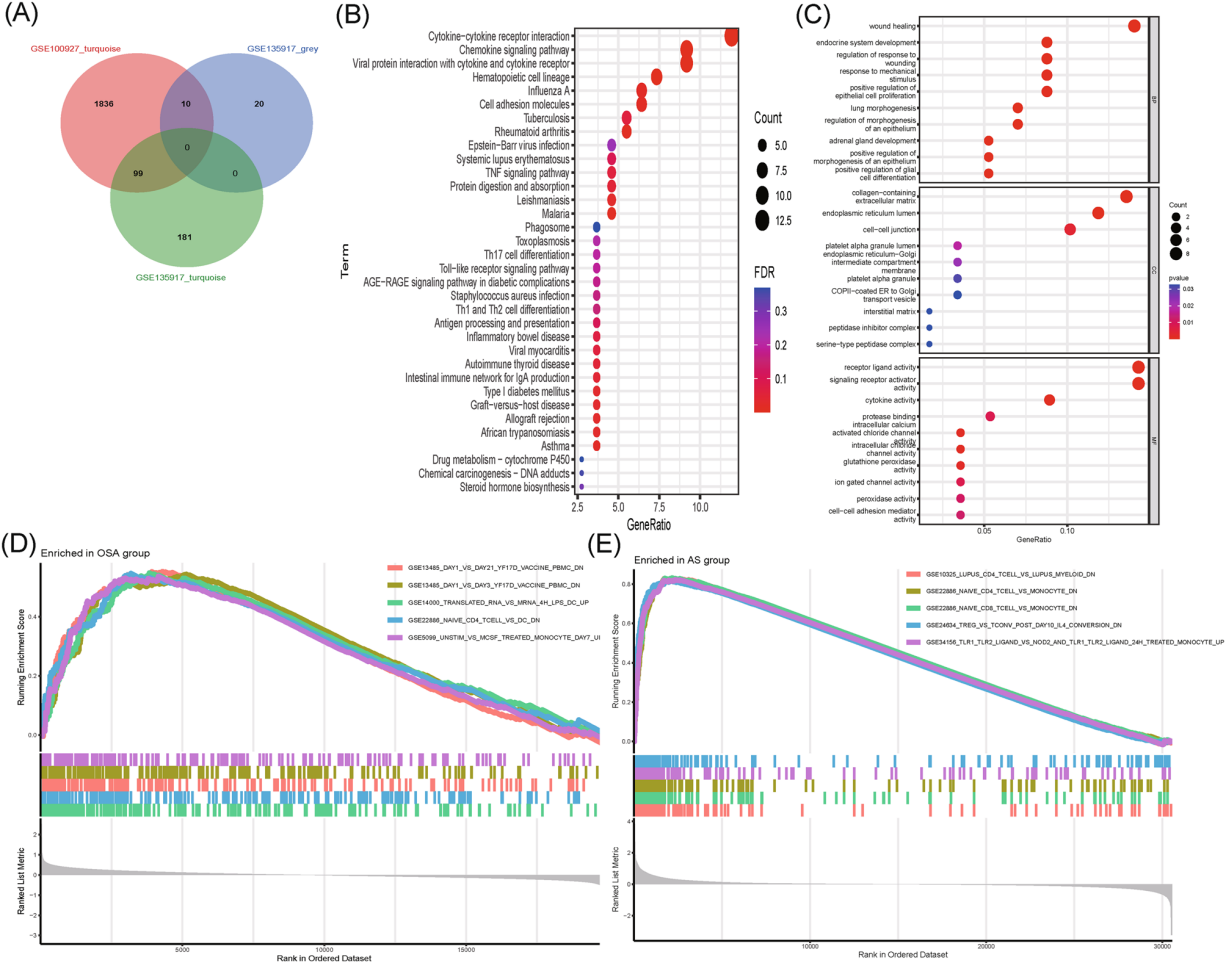
Identifying shared genes and shared pathways. (**A**) Venn diagram showing the overlap of 109 genes in the OSA and AS modules. (**B**) Bubble map of KEGG analysis of 109 shared genes between OSA and AS from the WGCNA screen. (**C**) Bubble map of GO analysis of 109 shared genes between OSA and AS screened by WGCNA. (**D**) Results of the Single-gene GSEA analyses of DEGs in OSA. (**E**) Results of the Single-gene GSEA analyses of DEGs in AS.

In total, 109 genes (Fig. 5A, see Supplementary Table S7 online) overlapped between the strongest positive and negative modules of OSA and AS, which may be associated with the pathogenesis of OSA and AS. Enrichment analysis was performed for these 109 genes. KEGG analysis indicated that these genes may be involved in chemokine signaling pathways and cell adhesion molecule pathways (Fig. 5B, see Supplementary Table S8 online). GO analysis showed that these genes were present in the extracellular matrix (Fig. 5C, see Supplementary Table S9 online). GSEA was then performed on OSA and AS samples and immune responses were found to be involved in common pathogenic processes (Fig. 5D,E). Therefore, we propose that the onset of OSA and AS is driven by a combination of immune responses and changes in cell adhesion molecules.

### Potential shared diagnostic genes selection by least absolute shrinkage and selection operator

**Figure 6:**
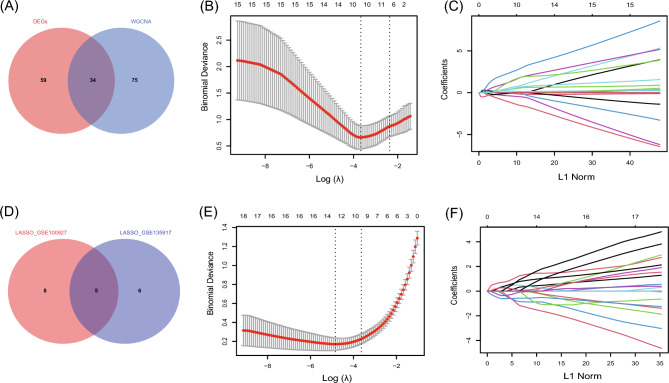
Potential shared diagnostic genes selection by least absolute shrinkage and selection operator. (**A**) The Venn diagram shows that 34 key genes are overlapping between the genes screened by WGCNA and those screened by DEGs. (**B**) LASSO regression models were used to identify potential shared diagnostic genes. Ten-time cross-validation was performed to select the optimal tuning parameter log (lambda) in the GSE135917 database. (**C**) LASSO coefficient profiles of shared genes in the GSE135917 dataset. (**D**) Venn diagram showing the key shared biomarkers. (**E**) Tenfold cross-validation to select the optimal tuning parameter log (lambda) in the GSE100927 database. (**F**) LASSO coefficient profiles of shared genes in the GSE100927 dataset.

After taking the intersection of the Venn diagrams for DEGs and modular hub genes (Fig. 6A), 34 shared genes were identified (see Supplementary Table S10 online). To narrow the range of potentially shared diagnostic gene biomarkers among the 34 DEGs, we used the LASSO model (Fig. 6B,C,E,F, see Supplementary Table S11 online). Five shared key genes (Fig. 6D, see Supplementary Table S12 online) were mined using LASSO regression: ERRFI1, FNDC1, HLA-DRB1, HSD11B1, and PERP.

### Candidate biomarker expression levels and diagnostic value

**Figure 7:**
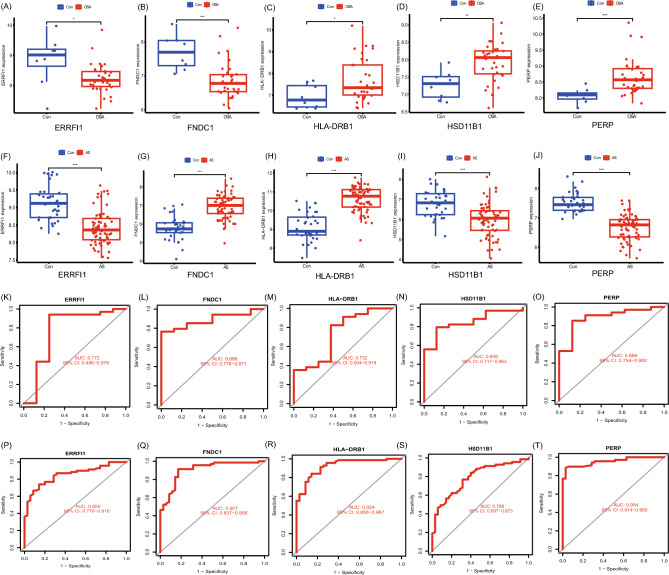
Candidate gene expression levels and diagnostic value. (**A**–**E**) Expression of *ERRFI1*, *FNDC1*, *HLA-DRB1*, *HSD11B1*, and *PERP* in GSE135917. (**F**–**J**) Expression of *ERRFI1*, *FNDC1*, *HLA-DRB1*, *HSD11B1*, and *PERP* in GSE100927. (**K**–**O**) ROC curves of *ERRFI1*, *FNDC1*, *HLA-DRB1*, *HSD11B1*, and *PERP* genes in GSE135917. (**P**–**T**) ROC curves of *ERRFI1*, *FNDC1*, *HLA-DRB1*, *HSD11B1*, *PERP* genes in GSE100927. Con: control; * represents *p* < 0.05; ** represents *p* < 0.01; *** represents P < 0.001.

Expression of the five key genes identified in the GSE135917 and GSE100927 datasets. Interestingly, differential expression analysis showed that ERRFI1 and FNDC1 were significantly under-expressed in OSA, and HLA-DRB1, HSD11B1, and PERP were significantly overexpressed in OSA (Fig. 7A–E). However, the FNDC1 and HLA-DRB1 expression was significantly elevated, and the ERRFI1, HSD11B1, and PERP expression were significantly decreased in AS (Fig. 7F–J). We also determined the diagnostic capabilities of these five shared markers based on receiver operating characteristic (ROC) analysis. In GSE135917, we obtained an area under the curve (AUC) > 0.6 for all five genes (Fig. 7K–O). In GSE100927, the AUC of the five genes obtained was > 0.6 (Fig. 7P–T), indicating relatively satisfactory diagnostic efficiency.

### Validation of external datasets and qRT-PCR results

**Figure 8:**
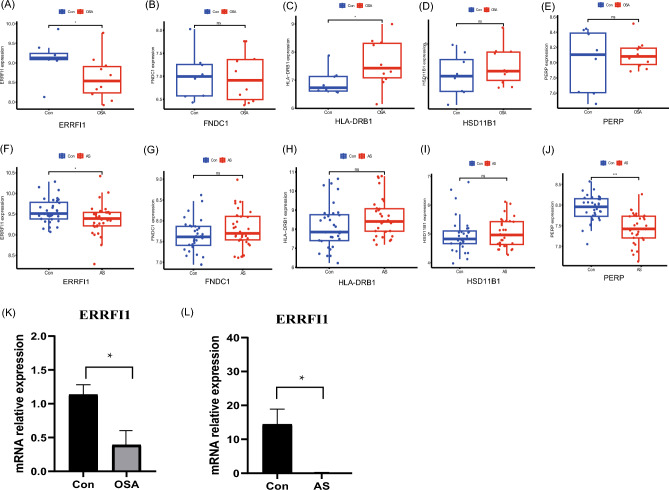
Validation of external datasets and qRT-PCR results. (**A**–**E**) Expression of *ERRFI1*, *FNDC1*, *HLA-DRB1*, *HSD11B1*, and *PERP* in GSE38792. (**F**–**J**) Expression of *ERRFI1*, *FNDC1*, *HLA-DRB1*, *HSD11B1*, and *PERP* in GSE43292. (**K**, **L**) qRT-PCR analysis of mRNA expression levels of *ERRFI1* in PBMC from patients and healthy controls. * represents *p* < 0.05, *** represents *p* < 0.001, ns represents no statistical difference.

We validated the expression of ERRFI1, FNDC1, HLA-DRB1, HSD11B1, and PERP in OSA using the external validation dataset, GSE38792. Differential expression analysis showed significant differences (*p* < 0.05) in ERRFI1 and HLA-DRB1, similar to the above results (Fig. 8A–E); however, the remaining three genes were not statistically significant (*p* > 0.05) in the validation set. In another independent AS validation set, GSE43292, the expression levels of ERRFI1 and PERP differed significantly across samples, similar to the above results (Fig. 8F–J); however, the remaining three genes were not statistically significant (*p* > 0.05) in the validation set. Interestingly, there were significant differences (*p* < 0.05) in ERRFI1 expression in the OSA and AS groups compared to controls. For this differential gene, fresh whole blood samples were collected from ten patients, PBMC was extracted, and qRT-PCR analysis was performed to further validate the differential expression of ERRFI1 in the patient samples. The results showed reduced ERRFI1 expression in patients with OSA compared to normal subjects, and similarly reduced ERRFI1 expression in patients with AS (*p* < 0.05) (Fig. 8K,L), suggesting that ERRFI1 has the potential to be a shared diagnostic marker for both diseases.

### Immune cell infiltration and its correlation with candidate biomarkers

**Figure 9:**
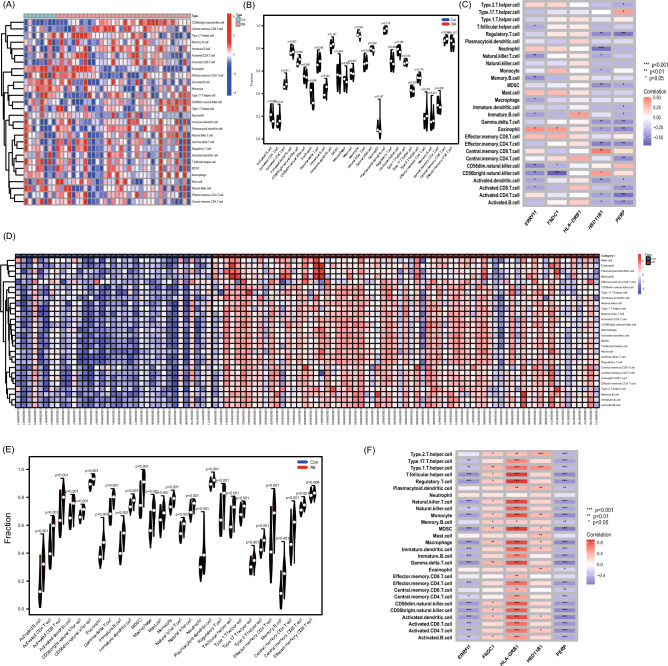
Analysis of immune infiltration associated with OSA and AS. Heatmap (**A**) and violin plot (**B**) show the distribution of 28 immune cells in the GSE135917 sample. (**C**) Association of key shared genes with immune cell infiltration in GSE135917 samples. Heatmap (**D**) and violin plot (**E**) show the distribution of 28 immune cells in the GSE100927 sample. (**F**) Association of key shared genes with immune cell infiltration in GSE100927 samples.

This study investigated the differences in immune cell infiltration in different samples, taking into account the important role of the immune response in the development of OSA and AS. In GSE135917, 28 immune cells were identified and shown in the heat and violin plots (Fig. 9A,B), and in GSE100927 28 immune cells were identified and shown in the heat and violin plots (Fig. 9D,E). Additionally, the screened candidate biomarkers were found to be closely related to immune cells (Fig. 9C,F). ERRFI1 expression was significantly associated (*p* < 0.05) with T follicular helper cells, natural killer T cells, Memory B cells, macrophages, immature B cells, eosinophils, CD56dim natural killer cell, CD56brght natural killer cells, activated dendritic cells, and Activated CD8 T cells in OSA samples. In the AS samples, ERRFI1 expression was significantly associated (*p* < 0.05) with type 17 T helper cells, type 1 T helper cells, T follicular helper cells, regulatory T cells, natural killer T cells, natural killer cells, monocytes, MDSC, macrophages, immature dendritic cells, immature B cells, gamma delta T cells, effector memory CD4 T cells, central memory CD4 T cells, CD56dim natural killer cell, CD56brght natural killer cells, activated dendritic cells, activated CD8 T cells, activated CD4 T cells, and activated B cells.

## Discussion

OSA has a high prevalence globally and is one of the main causes of atherosclerosis^18^. Previous studies have shown a close association between OSA and AS^19^ and have also identified mechanistic differences^12^. We consider that OSA and AS share a common mechanism of response to systemic injury; however, there is little research on this mechanism. We, therefore, conducted a systematic biological approach in order to explore possible shared pathways of action in OSA and AS, as well as new possible potential diagnostic and therapeutic targets in OSA and AS, and found that OSA and AS may be driven by a combination of immune responses and molecular changes in cell adhesion, with ERRFI1 as a key shared gene steadily declining in OSA and AS patients with a well diagnostic role.

We obtained common differential genes by taking the intersection of differential genes from the OSA and AS datasets. Enrichment analysis of these common differential genes revealed the involvement of immune, inflammatory processes, and cell adhesion molecular pathways. Previous studies have shown the involvement of immune responses in the pathogenesis of both OSA and AS, with the presence of proinflammatory factors^20^ and immune cell aggregation in atherosclerotic plaques^21^. Immune cells such as B lymphocytes, NK cells, and CD8 + /CD56 + cells are involved in the inflammatory process, leading to the development of cardiovascular complications in OSA^22,23^. Cell adhesion molecules are molecules that cause adhesion between cells, between cells and the matrix, or between cell–matrix cells. They are involved in cell recognition, activation, signaling, cell proliferation, and differentiation and are the molecular basis of a series of important physiological and pathological processes, such as immune response, inflammatory response, and tumor metastasis. Intermittent hypoxia increases cell permeability by generating reactive oxygen species that cause VE-cadherin to be excluded^24^. Potential role of monocyte adhesion, chemotaxis, and macrophage polarization in the development of OSA-induced cardiovascular disease^25^. OSA also acts on tumors through integrin-based adhesion^26^. Similarly, cell adhesion factors play an important role in the disease progression. Cell adhesion molecules have been found to potentially contribute to atherosclerotic plaque rupture during AS progression of AS^27^. Therefore, we consider that OSA and AS share a common pathogenesis, involving immune responses, inflammatory processes, and cell adhesion factors.

A total of 109 genes overlapped in the strongest positive and negative modules of the WGCNA for OSA and AS. Thus, these genes may be involved in the pathogenesis of OSA and AS. We identified 34 shared genes based on the intersection of DEGs and modular hub genes using Venn diagrams. To narrow down the range of potentially shared diagnostic genes among the 34 DEGs, we used the LASSO model. Using LASSO regression, five shared key genes were mined: ERRFI1, FNDC1, HLA-DRB1, HSD11B1, and PERP. We then validated these five genes with a validation dataset, collected blood samples from patients with OSA and AS, and found that only the ERRFI1 gene was stably decreased in OSA and AS. This is the first time that ERRFI1 has been identified and reported as a potential diagnostic marker for these two diseases, which has never been addressed in previous studies. ERRFI1 (also known as Mig6, RALT, or gene 33) is an adapter protein with complex functions in cell biology and human diseases. Interestingly, ERRFI1 was found to play different roles in different cells and tissues^28^. In human umbilical vein endothelial cells, ERRFI1 exerts antiapoptotic and antiangiogenic effects by inhibiting EGFR signaling^29^. Knockdown of ERRFI1 in human lung microvascular endothelial cells promotes apoptosis^30^, which induces endothelial proliferation of vascular smooth muscle cells and increased cell migration and proliferation of vascular smooth muscle cells^31^. ERRFI1 can also act on EGFR expressed in intimal smooth muscle cells of human atherosclerotic plaques and plays a key role in the development of atherosclerosis. It has also been shown that smooth muscle cells in atherosclerosis show increased EGFR downstream signaling and EGFR phosphorylation^31^. Furthermore, ERRFI1 reduces the production of inflammatory mediators^32^ and regulates excessive inflammatory responses by regulating the activation of EGFR in endotoxemia. This inflammatory factor expression is significantly increased (*p* < 0.05) when ERRFI1 is knocked down^33^. Therefore, we postulated that decreased ERRFI1 in OSA and AS could cause apoptosis of vascular endothelial cells, induce vascular smooth muscle cell proliferation, and increase inflammatory. These findings suggest a reduced protective factor against the pathogenesis of both diseases. This study provides new ideas for future research on the protective mechanisms of both OSA and AS.

Considering the important role of the immune response in the development of OSA and AS, we investigated the differences in immune cell infiltration in different samples. Our results showed that both OSA and AS were closely associated with immune cell infiltration, consistent with the findings of previous studies^23^. OSA has a significantly effect on the number of circulating inflammatory cells, lymphocytes, natural killer (NK and NKT-like cells^34^. Furthermore, aggregation of pro-inflammatory factors and immune cells was also present in atherosclerotic plaques^20,21^. Interestingly, Activated CD8 T cells and CD56 bright natural killer cells showed the same trends in OSA and AS. Previous studies have shown that T lymphocyte abnormalities play an important role in endothelial cell dysfunction^35^. The study Zhang et al. showed elevated levels of CD8 + T lymphocytes in patients with OSA compared to controls^16^ and that activated CD8 + T lymphocytes can have a killing effect on vascular endothelial cells, thus aiding the progression of secondary atherosclerosis. Atherosclerosis is a chronic inflammatory disease with immune infiltration^36^, and Patients with atherosclerosis have significantly elevated CD8 T cells^37^, mainly in the fibrous cap area. Second, NKs cells play an important role in atherosclerosis^38,39^. A study of 48 patients with OSA found that both NK and NKT cells were increased in patients with OSA compared with controls^34^. CD56bright natural killer cells cause cell lysis, vascular endothelial cell damage, and atherosclerosis by releasing proinflammatory factors, antibody-dependent cytotoxicity, perforins, and granzymes^40,41^. In the correlations between ERRFI1 and 28 immune cells, we found a significant negative correlation between ERRFI1 and cellular immunity, suggesting a decrease in ERRFI1 and an increase in cellular immunity, which may be associated with inflammation and impaired vascular endothelial function in both diseases.

Our study has certain limitations. First, we only studied peripheral blood lymphocytes from patients; therefore, we could not explore the systemic changes in ERRFI1 in OSA and AS. Second, the small patient sample raises the possibility of bias. We plan to expand the collection of blood and tissue samples in future research in order to validate these results.

## Conclusion

Despite these limitations, this is the first study to use bioinformatics to explore the common mechanisms of action of OSA and AS, and our results suggest that immune pathways, inflammatory processes, and cell adhesion molecules are implicated in the pathogenesis of OSA and AS. Furthermore, we identified for the first time that ERRFI1 may be a potential diagnostic marker for OSA and AS, declining in both diseases. This suggests that a common underlying mechanism involving ERRFI1 may exist. Immune cell infiltration results showed that both OSA and AS were closely associated with immune cell infiltration, and ERRFI1 was closely associated with cellular immunity. This study offers a new perspective for exploring the common pathogenesis of OSA and AS and the intrinsic link between the two diseases and screening for a stable and reliable shared disease marker that can provide potential diagnostic and therapeutic targets for both diseases. We plan to further investigate the mechanisms of cell adhesion molecular pathways and ERRFI1 expression in OSA and AS based on both in vivo and in vitro experiments.

## Methods

### Datasets and data pre-processing

Figure 1 illustrates the workflow chart of data preparation, processing, analysis, and validation. Gene expression data for OSA and atherosclerosis were downloaded from the GEO database^42^. Further information is available online (https://www.ncbi.nlm.nih.gov/geo/ datasets). The dataset screening criterion was that each dataset should have no fewer than 12 samples (Control sample + sample size of disease patients) to ensure the accuracy of the WGCNA. The GSE135917 dataset, based on the GPL6244 platform, was then downloaded and contained samples from eight control groups and 34 patients with OSA. To validate the diagnostic effect of the gene, the GSE38792 dataset based on GPL6244 was downloaded. The data set contained samples from 8 control subjects and 10 patients with OSA. Adipose tissue samples were collected from patients in both datasets.

To investigate atherosclerosis, the GSE100927 dataset based on the GPL17077 platform, which included 35 controls and 69 atherosclerotic samples, was downloaded. To validate the diagnostic value of the gene, the GSE43292 dataset based on GPL6244, which contained samples from 32 controls and 32 atherosclerotic patients, was downloaded. Samples from both datasets were collected from arterial blood vessels. All data annotation and extraction were performed using the R software ((R.4.2.1, https://www.r-project.org/).

### Differential genetic screening

Screening of the DEGs datasets for GSE135917 and GSE10092 was conducted. Limma R packag to screen DEGs. An adjusted *p*-value < 0.05 and |LogFC|> 0.5 were selected as the cut-off standard. R software was used to plot the differential gene clustering of the volcanoes.

### Construction and module analysis of weighted gene co-expression network analysis (WGCNA)

(Weighted Gene Co-expression Network Analysis) WGCNA^43^ is a bioinformatics analysis method used to describe gene association patterns among different samples, The WGCNA software package is used to perform WGCNA analysis. Co-expression networks corresponding to the clinical features of DEGs in OSA and AS were constructed using the WGCNA R package. First, hierarchical cluster analysis was performed using the cluster function in R to exclude outlier samples. Then, according to the criteria for scale-free networks, the "pickSoftThreshold" function in the WGCNA package was used to select a suitable soft-power threshold β (with values ranging from 1 to 20) for automatic network construction. The results were clustered using a topological overlap matrix analysis, which contained module assignments labeled by color and module eigengenes. Pearson’s correlation analysis was used to calculate the correlation between module feature (ME) and clinical characteristics. |correlation coefficient (*R*)|> 0.3 and *p*-values < 0.05 were considered significant for interactions with clinical features.

### Enrichment analysis

GO enrichment analysis is a bioinformatics method commonly used for comprehensive information retrieval from large-scale genetic data. The KEGG pathway enrichment analysis has been widely used to understand biological mechanisms and functions^44^. The GO plot program package was used to visualize the GO and KEGG pathways. Finally, important signaling pathways were explored using the cluster profile and GSVA packages. The gene sets and expression matrices of OSA and AS were analyzed using GSEA to explore their possible regulatory pathways.

### Identification of shared genes and Feature selection by the least absolute shrinkage and selection operator

Combinatorial analysis of genes from the WGCNA and DEG screens was performed by plotting Venn diagrams. Overlapping genes were considered core-shared genes. Lasso is an important method for regression that uses an ℓ1 penalty to achieve a sparse solution. We used the "glmnet" package in R to perform LASSO regression to filter the best predictors of OSA and AS in the intersection of the above DEGs and WCGNA.

### Candidate biomarker expression levels and diagnostic value

The expression levels of key shared genes (*p* < 0.05) were detected using the R software ggplot2 package box plots (GSE135917, GSE38792, GSE100927, and GSE43292). The AUC of ROC was used to determine the diagnostic value of potential biomarkers in the dataset (GSE135917, and GSE100927) using the pROC R package.

### Extraction of peripheral blood mononuclear cells (PBMC)

Purple anticoagulation tubes were used to collect 10 ml of whole blood samples from three patients with OSA, four patients with AS, and three normal controls. Human peripheral blood and phosphate buffer were mixed in a homogeneous ratio of 1:1 (10 ml:10 ml). Ten milliliters of lymphocyte separation liquid (SIGMA, 10,771) was applied in each 50 ml centrifuge tube, and 20 ml of blood was gently added to the phosphate buffer on the upper surface of the lymphocyte separation liquid. The tubes were centrifuged in a centrifuge for 30 min (2000 rpm, 20 °C). The middle and upper interface cloud layers were carefully collected and transferred to new 50 mL tubes. Finally, the PBMC was washed with phosphate buffer, centrifuged, and the supernatant was discarded. This study was approved by the Ethics Committee of First Hospital of Hebei Medical University and all participants provided their written informed consent to participate in the study. And the study was conducted in accordance with the relevant guidelines and the regulations.

### Quantitative Real-Time PCR (qRT-PCR)

Total RNA was extracted from the PBMC using the Eastep® Super Total RNA Extraction Kit (Promega Shanghai Ltd.). Pipette 1.5 μL of total RNA through the Nanodrop 1000 for measurement. RNA-to-cDNA transcription was performed using GoScriptTM Reverse Transcription Mix, Random Primer Protocol, and Oligo (dT) Protocol from Promega. qRT-PCR was performed using a Roche LightCycler 480 qRT-PCR amplifier following the manufacturer's instructions. Relative mRNA levels were normalized to the level of glyceraldehyde-3-phosphate dehydrogenase, using the 2-ΔΔCt method. (*ERRFI1*-Fwd: 5'-GAAGACCTACTGGAGCAGTCG-3'; *ERRFI1*-Rev: 5'-GACTTTTGAGATGGACCATTTCTG-3').

### ssGSEA

ssGSEA analysis using the "GSVA" R package was used to analyze the infiltration of 28 immune cells in lesions and normal samples. The correlation between core genes and the abundance of infiltrating immune cells was analyzed using Spearman's correlation coefficients and a *p*-value < 0.05 was considered statistically significant.

### Supplementary Information

Supplementary Information 1.

Supplementary Information 2.

## Data Availability

The datasets GSE135917, GSE38792, GSE100927 and GSE43292 for this study can be found in the GEO datasets (https://www.ncbi.nlm.nih.gov/geo/).
