# Supplementary material for: Exploring shared pathways and the shared biomarker ERRFI1 in Obstructive sleep apnoea and atherosclerosis using integrated bioinformatics analysis
Source: Sci Rep. 2023 Sep 12;13:15103. doi: 10.1038/s41598-023-42184-0 (PMC10497545; doi:10.1038/s41598-023-42184-0)
Supplement: Supplementary file 1 — Supplementary Information 1. [file 41598_2023_42184_MOESM1_ESM.pdf]

Exploring shared pathways and the shared biomarker ERRFI1 in Obstructive sleep apnoea and atherosclerosis using integrated bioinformatics analysis

Bowen Chen[1], Liping Dong[1], Jihua Zhang[2], Ying Hao[1], Weiwei Chi[1], Dongmei Song[1,2]

[1]Clinical Biobank, The First Hospital of Hebei Medical University, Shijiazhuang, China; [2]Department of Otolaryngology, The First Hospital of Hebei Medical University, Shijiazhuang, China

Correspondence to: Dongmei Song. The First Hospital of Hebei Medical University, Shijiazhuang, China.

Email: songdongmei@hebmu.edu.cn.

Supplementary Figure S1

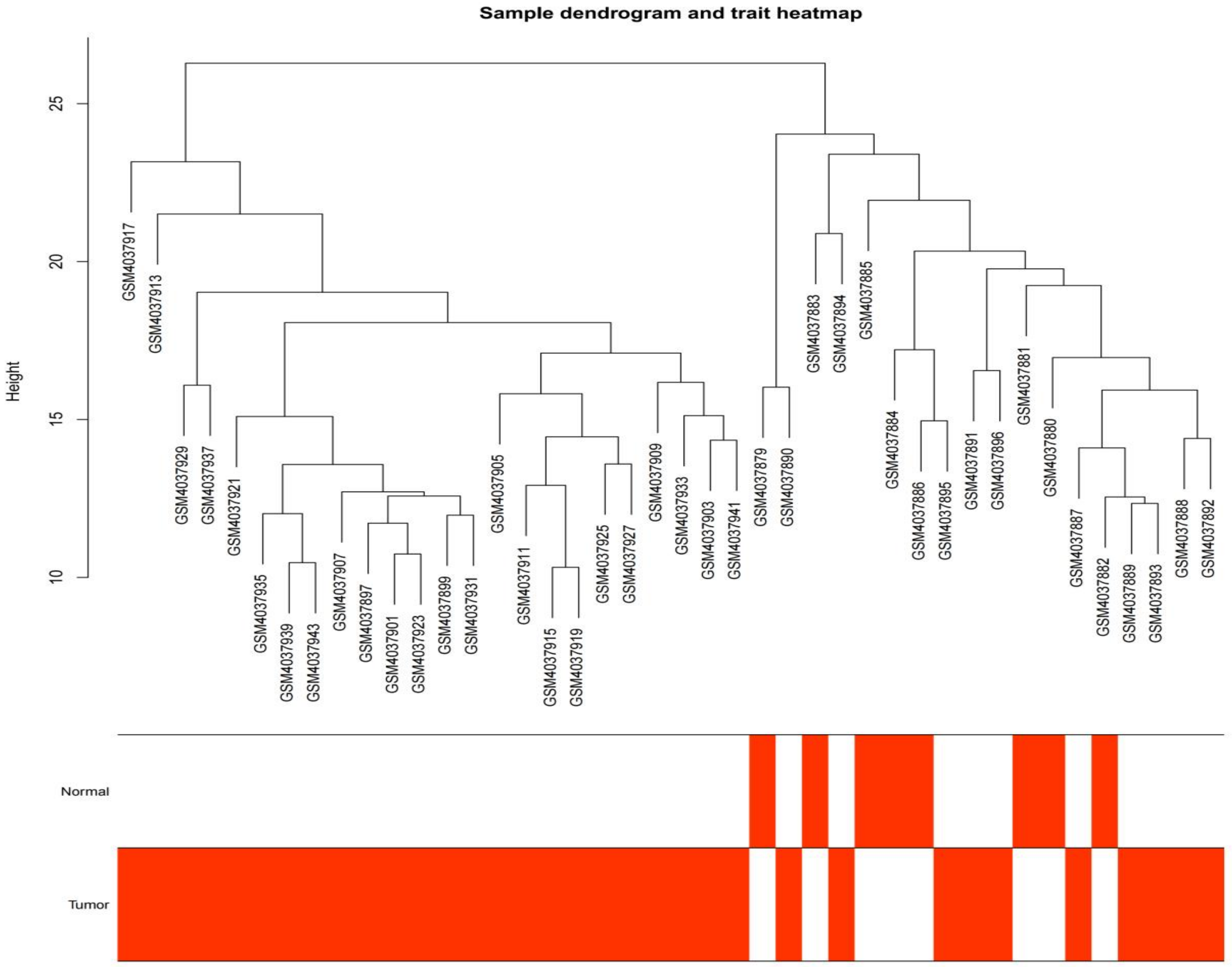

Sample dendrogram and trait heatmap in GSE135917

Supplementary Figure S2

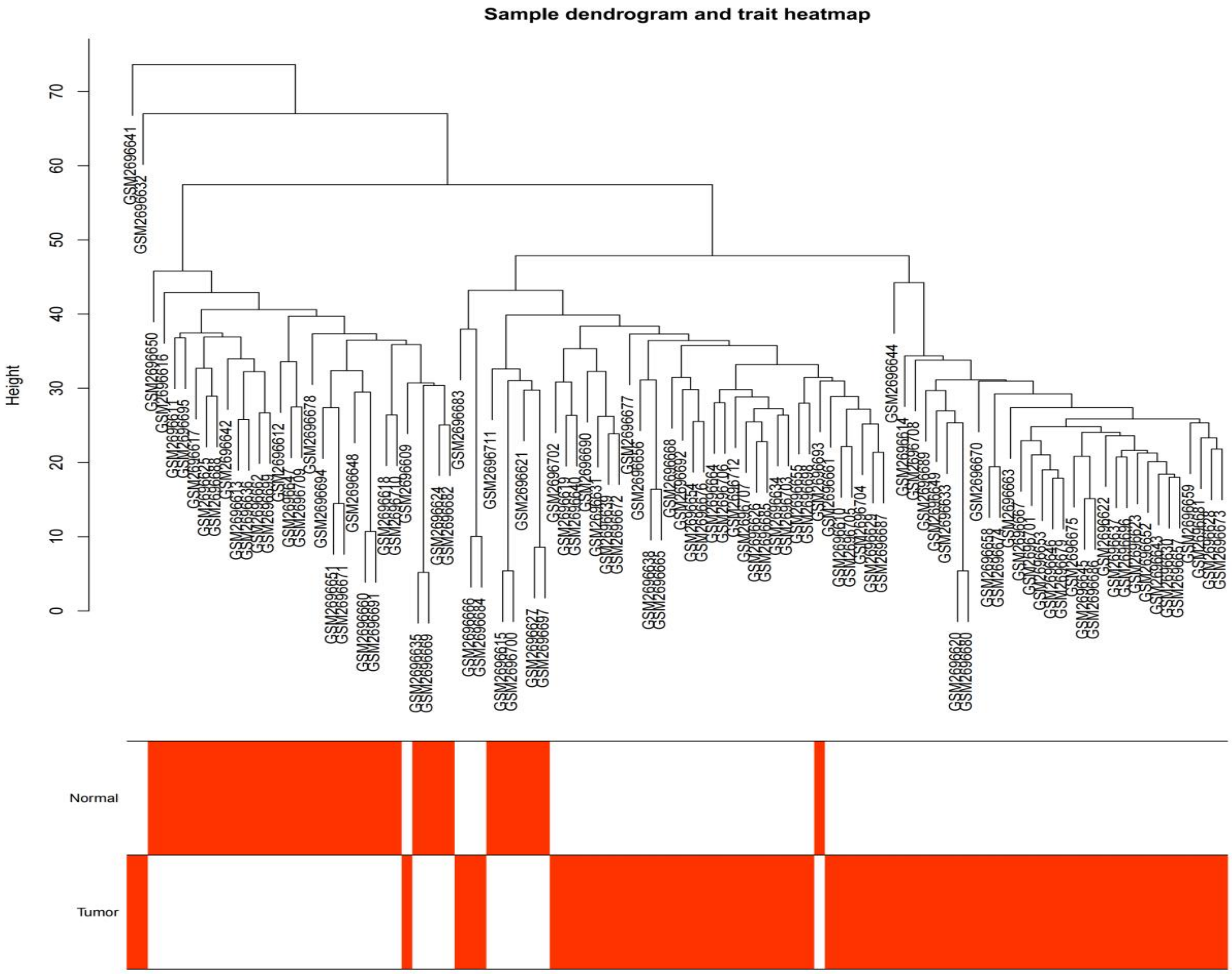

Sample dendrogram and trait heatmap in GSE100927

Supplementary Figure S3

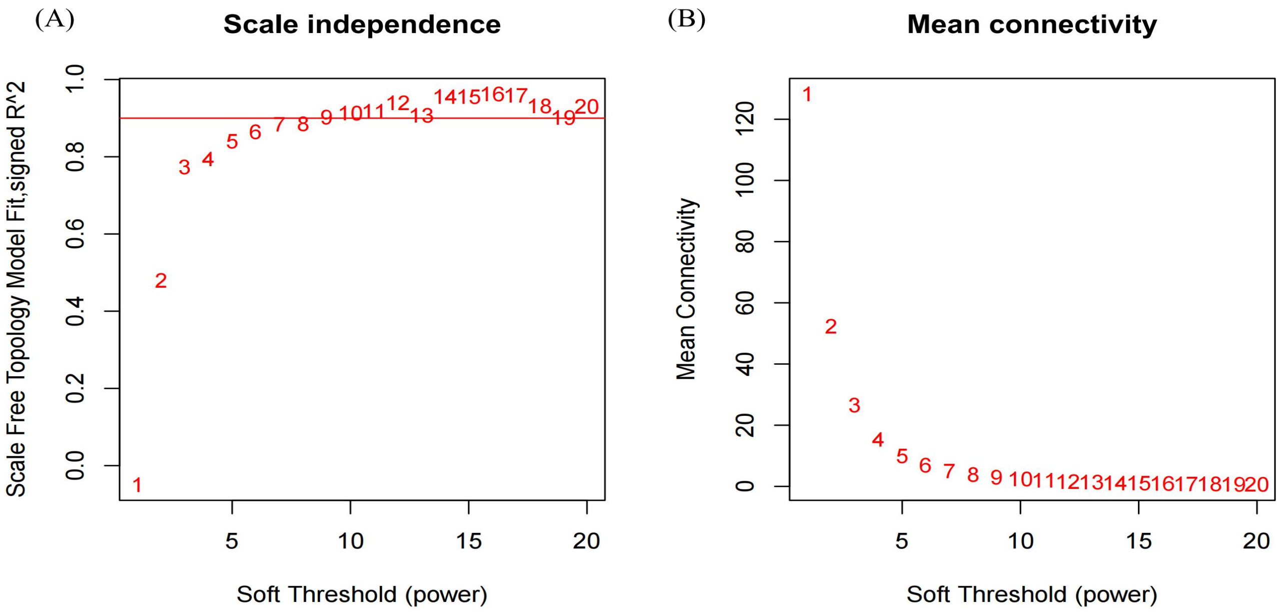

Scale-free exponent and average connectivity for each soft threshold
(A) Analysis of the scale-free index for various soft-threshold powers (β) in GSE135917.
(B) Analysis of the mean connectivity for various soft-threshold powers in GSE135917.

Supplementary Figure S4

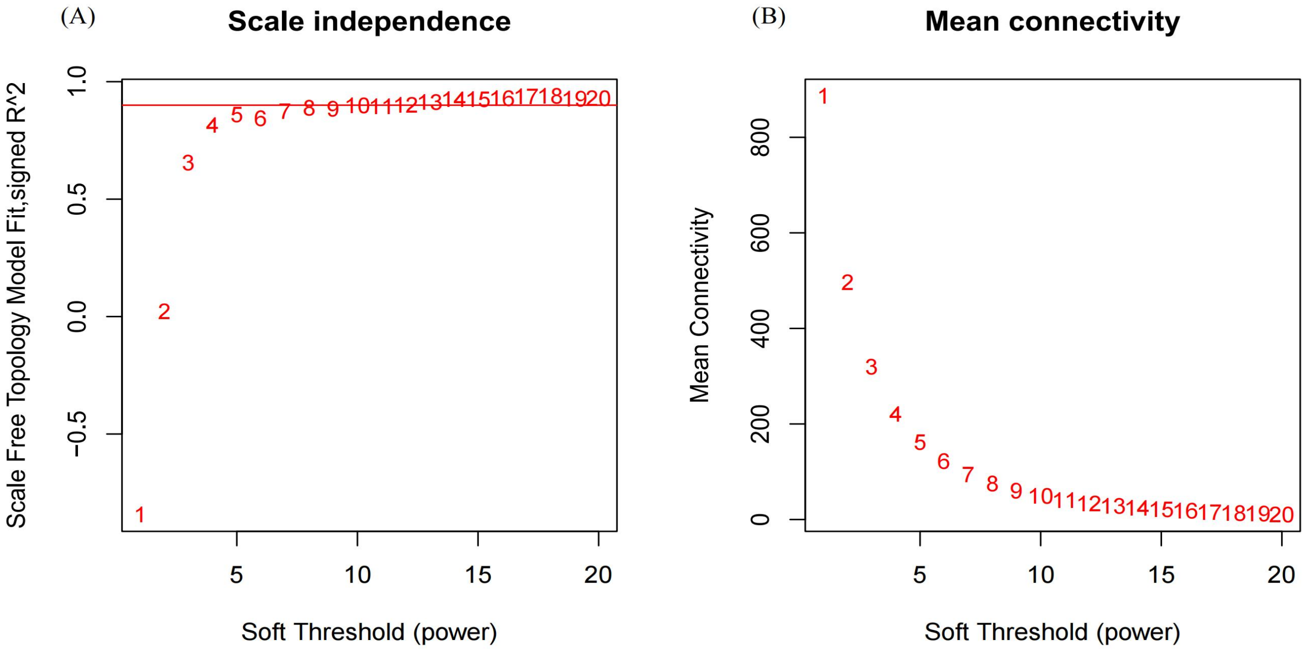

Scale-free exponent and average connectivity for each soft threshold
(A) Analysis of the scale-free index for various soft-threshold powers (β) in GSE100927.
(B) Analysis of the mean connectivity for various soft-threshold powers in GSE100927.

Supplementary Table S1

The DEGs of GSE135917								The DEGs of GSE100927						
id	logFC	AveExpr	t	P.Value	adj.P.Val	B		id	logFC	AveExpr	t	P.Value	adj.P.Val	B
C1GALT1	1.062199172	6.59491901	7.046814962	7.96E-09	0.000116861	10.00125728		LOC100506075	0.857643694	6.1298908	19.99630342	1.45E-37	4.42E-33	74.65656902
CCNYL1	0.646844626	7.110132967	6.935316212	1.17E-08	0.000116861	9.645496507		TMEM79	0.540895621	5.015157036	16.52647513	5.80E-31	8.86E-27	59.81238908
NIPSNAP3A	0.825467599	7.92348519	6.686178191	2.76E-08	0.000183995	8.848594364		SGK223	1.325693126	7.059483175	15.91680862	9.81E-30	9.99E-26	57.03962844
KATNAL1	0.528250198	8.37216435	6.512535837	5.03E-08	0.000222819	8.292042501		C2CD2L	0.788698912	7.205531802	15.71282728	2.55E-29	1.95E-25	56.10112827
LRIF1	0.779667828	6.169038058	6.429991653	6.69E-08	0.000222819	8.02728869		DGKZ	0.656879264	6.307041291	15.50853167	6.69E-29	4.09E-25	55.15582065
ATL1	0.51317266	6.331258487	6.376934316	8.03E-08	0.000229388	7.857079153		PLXND1	1.156697693	8.40727324	15.23511124	2.45E-28	1.07E-24	53.88234824
UCHL5	0.65141389	7.668934319	6.316129245	9.91E-08	0.000247599	7.662003165		YAF2	-0.592250469	5.827658033	-15.18900516	3.05E-28	1.16E-24	53.66667664
TPPP	-0.516506106	7.028014064	-6.256431507	1.22E-07	0.000270449	7.470486403		RNF44	0.706399786	6.607013366	15.09770136	4.71E-28	1.60E-24	53.23879676
SUGT1P1	0.729564809	4.226214962	6.153125358	1.74E-07	0.00028945	7.139142529		ZSWIM3	0.505811155	4.812582225	14.92680526	1.07E-27	3.26E-24	52.43513124
SPDL1	0.747175764	5.622259298	6.12017663	1.95E-07	0.00028945	7.03349627		MARCKS	1.034371828	8.806338517	14.9018469	1.20E-27	3.35E-24	52.31745814
FAM210A	0.611572542	6.165004178	6.11334563	1.99E-07	0.00028945	7.011596113		C16orf54	1.685032821	6.12340375	14.76321297	2.35E-27	5.29E-24	51.66243707
RPL34	-0.544357159	8.541259614	-6.094065063	2.13E-07	0.00028945	6.949788082		ADRBK1	0.785130925	7.945961147	14.74960612	2.50E-27	5.29E-24	51.59802037
YKT6	0.633734759	8.81710444	6.054533101	2.44E-07	0.00028945	6.823087235		ARHGAP27	1.040881067	6.819312946	14.74405185	2.57E-27	5.29E-24	51.57171918
BTG2	-0.786437548	8.639335929	-6.049616568	2.48E-07	0.00028945	6.80733244		LTB	1.487660437	5.876093177	14.74213186	2.60E-27	5.29E-24	51.56262661
RBBP4	0.750663032	8.975279827	6.0123398	2.83E-07	0.00028945	6.6879032		MYBL1	-0.848318178	5.146243594	-14.6773281	3.55E-27	6.78E-24	51.25546854
HSP90AA2P	0.875004066	7.962649038	6.012112532	2.83E-07	0.00028945	6.687175194		FLJ90757	0.761319172	5.488862759	14.56375327	6.15E-27	1.10E-23	50.71591844
GCA	1.087867762	6.910866325	6.005146067	2.90E-07	0.00028945	6.664860365		RASAL3	1.538596449	7.475984116	14.45188725	1.06E-26	1.79E-23	50.18297176
DPP8	0.520249048	8.426048425	5.952330662	3.47E-07	0.000301121	6.49573601		ARHGAP25	1.530362783	7.008088353	14.33318501	1.88E-26	3.03E-23	49.61582868
PRCP	0.613163537	9.030659298	5.906459872	4.07E-07	0.000301121	6.348933583		TRIM25	0.880298098	7.400802898	14.28503992	2.38E-26	3.55E-23	49.38532425
RPS25	-0.632467637	10.82760478	-5.816363824	5.54E-07	0.000357984	6.060863339		RIMBP3	1.06511236	5.967456759	14.27361668	2.52E-26	3.55E-23	49.33059324

CRLF3	0.614770779	7.591520935	5.808316573	5.70E-07	0.000357984	6.035152664		*RNF115*	-0.738101117	6.618517256	-14.26763435	2.59E-26	3.55E-23	49.30192465
LYSMD3	0.690220383	7.108047307	5.78992126	6.07E-07	0.000357984	5.976393269		*FAM110A*	1.06185258	6.981790466	14.2610791	2.67E-26	3.55E-23	49.27050568
DLEU2	1.277267727	6.422368958	5.762570848	6.67E-07	0.000380811	5.88906355		*HSPB7*	-1.046035068	5.260102941	-14.14363707	4.74E-26	5.80E-23	48.70676411
FAM200B	0.942819625	5.528122374	5.750832843	6.94E-07	0.000385446	5.851597338		*LARP6*	-0.753379511	7.237959151	-14.11357656	5.49E-26	6.46E-23	48.56221176
RALA	0.560793141	7.655706843	5.719823221	7.72E-07	0.000406129	5.752658399		*SNORA8*	-0.559757245	6.356492307	-14.05668294	7.26E-26	8.21E-23	48.28834213
PTPN22	0.95608515	4.992721838	5.681628342	8.80E-07	0.000439136	5.630877733		*ZMIZ2*	0.801133407	7.302611784	14.04384418	7.73E-26	8.43E-23	48.22648868
DSCC1	0.542278053	5.217484249	5.680408452	8.84E-07	0.000439136	5.626989817		*MFNG*	1.132644757	6.429786387	13.86244555	1.88E-25	1.98E-22	47.35056215
ZNF724	0.775698443	4.926446313	5.669072366	9.19E-07	0.000439136	5.590865345		*ZGLP1*	0.813832167	5.795813543	13.84621472	2.04E-25	2.08E-22	47.27200709
MAPK9	0.575351942	8.772878035	5.661043228	9.44E-07	0.000439136	5.565284393		*DOK1*	0.908443547	7.920529156	13.8105779	2.43E-25	2.40E-22	47.09942646
CYP4F12	0.804901306	6.190989497	5.646791499	9.92E-07	0.000440439	5.519889277		*ADAP2*	1.726375049	8.173636886	13.7648272	3.05E-25	2.82E-22	46.87765984
LTV1	0.617223966	7.638683144	5.611455708	1.12E-06	0.000475891	5.407399713		*ST8SIA4*	0.570764006	4.936955877	13.73688765	3.49E-25	3.14E-22	46.74211505
SNRPD2	-0.616313463	8.348150294	-5.57474846	1.27E-06	0.000528279	5.290644181		*FAM78A*	1.476953604	5.952814498	13.5774548	7.68E-25	6.52E-22	45.96701368
RAB28	0.595273754	6.781853768	5.555682626	1.35E-06	0.00053262	5.230043219		*MNAT1*	-0.54580774	6.320441392	-13.57144245	7.91E-25	6.54E-22	45.93772996
OCIAD1	0.515600148	7.67964068	5.551726583	1.37E-06	0.00053262	5.217472611		*CPT1B*	0.764581572	5.014067921	13.56494357	8.17E-25	6.57E-22	45.90607217
C8orf22	0.673111375	4.903841929	5.548825046	1.39E-06	0.00053262	5.208253597		*SRRM4*	-0.946239521	4.572338249	-13.36645508	2.19E-24	1.71E-21	44.93701423
CD58	0.527826138	7.670501622	5.54372188	1.41E-06	0.00053262	5.192041072		*AMPH*	-0.894334512	4.55446728	-13.33893462	2.51E-24	1.92E-21	44.8023268
GTPBP8	0.592555505	6.318039017	5.54334281	1.41E-06	0.00053262	5.190836872		*CMTM3*	0.98245095	8.323832576	13.30616406	2.95E-24	2.20E-21	44.64184229
PUS7L	0.690237804	6.825314632	5.534160534	1.46E-06	0.000539404	5.161670999		*DCDC2C*	-0.848661344	4.297575453	-13.26536417	3.62E-24	2.63E-21	44.44188146
WT1-AS	-0.607229431	5.76741378	-5.507142845	1.60E-06	0.000564476	5.075895988		*HMGA1P4*	0.987305063	5.442843815	13.22109012	4.51E-24	3.21E-21	44.22470054
ACTL6A	0.565110869	6.6987422	5.497314992	1.65E-06	0.000564476	5.044710645		*TBC1D10C*	1.643521422	6.90739424	13.20199652	4.96E-24	3.38E-21	44.13097752
TCTEX1D2	0.535493553	5.318227754	5.471601274	1.80E-06	0.000564476	4.963158111		*MOB3A*	0.692791093	6.614720169	13.18517941	5.40E-24	3.55E-21	44.04839827
WNT11	-0.570928053	8.092813095	-5.470134158	1.81E-06	0.000564476	4.958506894		*HDAC10*	0.619197666	4.897528943	13.18256226	5.47E-24	3.55E-21	44.03554435

UCHL1	*0.778431779*	*9.230388512*	*5.437207355*	*2.03E-06*	*0.000587497*	*4.854172016*		*FHOD1*	*0.739666725*	*6.809259778*	*13.11264332*	*7.75E-24*	*4.83E-21*	*43.69188803*
MCMDC2	*0.548796451*	*4.927490619*	*5.394191307*	*2.35E-06*	*0.000625685*	*4.718026648*		*SAMD9L*	*0.837994936*	*6.370870157*	*13.03311768*	*1.15E-23*	*7.05E-21*	*43.30042114*
HIST1H2BC	*0.924438025*	*6.460786709*	*5.358562966*	*2.65E-06*	*0.000662078*	*4.605406542*		*ABCC5*	*0.828187675*	*6.457407552*	*12.94528023*	*1.79E-23*	*1.05E-20*	*42.86731678*
RNF14	*0.66740365*	*9.327977223*	*5.342839458*	*2.80E-06*	*0.000668531*	*4.555747933*		*HMHA1*	*1.469246721*	*8.724062665*	*12.88188847*	*2.46E-23*	*1.35E-20*	*42.55428408*
VAMP4	*0.639354533*	*7.350668168*	*5.320499246*	*3.02E-06*	*0.000692165*	*4.485238761*		*C21orf63*	*-0.734831927*	*5.807035548*	*-12.87675866*	*2.52E-23*	*1.35E-20*	*42.52893588*
HAUS6	*0.591403152*	*6.945586228*	*5.303628399*	*3.19E-06*	*0.000701318*	*4.432028783*		*RXRA*	*0.522810886*	*8.098535485*	*12.8767059*	*2.53E-23*	*1.35E-20*	*42.52867517*
DCK	*0.531045397*	*6.781018912*	*5.288315733*	*3.36E-06*	*0.00070221*	*4.383761427*		*PTPRE*	*0.847554526*	*5.887732723*	*12.87345036*	*2.57E-23*	*1.35E-20*	*42.51258709*
TIGD1	*0.654948199*	*5.942298722*	*5.28075312*	*3.45E-06*	*0.00070221*	*4.359933212*		*DDX60L*	*1.123760932*	*6.253025552*	*12.87319476*	*2.57E-23*	*1.35E-20*	*42.51132395*
C1GALT1C1	*0.640105984*	*6.690139906*	*5.273017378*	*3.54E-06*	*0.00070221*	*4.335566454*		*STK10*	*1.18436621*	*6.685269284*	*12.85394305*	*2.83E-23*	*1.47E-20*	*42.41616573*
NAE1	*0.650961173*	*7.342949307*	*5.268069879*	*3.60E-06*	*0.00070221*	*4.31998608*		*TNF*	*1.437917099*	*5.64051551*	*12.84036607*	*3.03E-23*	*1.54E-20*	*42.34903568*
IRAK4	*0.554016923*	*7.014700433*	*5.263839368*	*3.65E-06*	*0.00070221*	*4.306665914*		*CSK*	*0.916856121*	*6.121959906*	*12.79977968*	*3.72E-23*	*1.86E-20*	*42.1482566*
CALU	*0.677754308*	*9.066063352*	*5.255348606*	*3.76E-06*	*0.000705734*	*4.279938424*		*HENMT1*	*0.651156152*	*6.388627474*	*12.71407962*	*5.72E-23*	*2.77E-20*	*41.72379719*
LPCAT2	*0.689009926*	*7.604520699*	*5.232827542*	*4.06E-06*	*0.000730719*	*4.20908835*		*SNORD116-4*	*-0.884177072*	*5.034244071*	*-12.690253*	*6.45E-23*	*3.03E-20*	*41.60566717*
RPL13P5	*0.592295247*	*5.356934671*	*5.219344309*	*4.25E-06*	*0.000740643*	*4.16670081*		*SLC12A9*	*0.558442455*	*6.28323844*	*12.67013407*	*7.13E-23*	*3.22E-20*	*41.50587912*
NUDT5	*0.521079764*	*7.36427707*	*5.212499104*	*4.35E-06*	*0.000740643*	*4.14519012*		*CCDC142*	*0.683424199*	*6.200145588*	*12.66930759*	*7.16E-23*	*3.22E-20*	*41.50177909*
LHX9	*0.503750706*	*5.638772156*	*5.208904071*	*4.40E-06*	*0.000740643*	*4.133895298*		*STAB1*	*1.396533524*	*7.769887511*	*12.65199042*	*7.82E-23*	*3.46E-20*	*41.41585674*
FUCA2	*0.641677869*	*8.276791181*	*5.20880025*	*4.40E-06*	*0.000740643*	*4.133569139*		*PPP1R9A*	*-1.015354255*	*4.601066722*	*-12.64918747*	*7.93E-23*	*3.46E-20*	*41.40194686*
SNORD115-32	*-0.62583251*	*4.22409662*	*-5.201066896*	*4.52E-06*	*0.000740643*	*4.109278287*		*CSF1R*	*1.586858921*	*9.675234077*	*12.64626092*	*8.04E-23*	*3.46E-20*	*41.38742281*
TMEM106B	*0.603442113*	*8.311025351*	*5.199073024*	*4.55E-06*	*0.000740643*	*4.103016675*		*OSBPL3*	*1.040010381*	*5.731065384*	*12.63267819*	*8.61E-23*	*3.64E-20*	*41.32000361*
TCEANC2	*0.533134845*	*7.132066553*	*5.18116388*	*4.83E-06*	*0.000772626*	*4.046797364*		*AGPAT4-IT1*	*0.988669456*	*6.213273922*	*12.63056595*	*8.71E-23*	*3.64E-20*	*41.30951779*
OR52J3	*-0.503929431*	*4.880830135*	*-5.169211188*	*5.03E-06*	*0.000778716*	*4.009299536*		*ANPEP*	*1.311233336*	*5.958915921*	*12.62620403*	*8.90E-23*	*3.67E-20*	*41.2878626*
BRMS1L	*0.585776165*	*5.566905713*	*5.160194545*	*5.19E-06*	*0.000778716*	*3.981025178*		*XLOC_009509*	*-0.64739604*	*5.321581169*	*-12.61674812*	*9.33E-23*	*3.80E-20*	*41.24091196*

DNAJC15	*0.611581864*	*7.79754287*	*5.136182844*	*5.62E-06*	*0.000797789*	*3.905782798*		*AMICA1*	*0.940748339*	*6.037143461*	*12.60950646*	*9.68E-23*	*3.89E-20*	*41.20495012*
ATP6AP2	*0.748606395*	*9.983775606*	*5.120306284*	*5.93E-06*	*0.000797789*	*3.856075842*		*PRKG1*	*-0.642777605*	*4.843119287*	*-12.60279327*	*1.00E-22*	*3.93E-20*	*41.17160844*
CEP44	*0.603726506*	*5.812996716*	*5.116064458*	*6.02E-06*	*0.000797789*	*3.842801303*		*VASH1*	*0.748182074*	*8.076737997*	*12.60246966*	*1.00E-22*	*3.93E-20*	*41.1700011*
KCNJ1	*-0.565719867*	*4.317544086*	*-5.115599342*	*6.03E-06*	*0.000797789*	*3.841345904*		*C6orf62*	*0.593188834*	*8.6287207*	*12.57823798*	*1.13E-22*	*4.38E-20*	*41.04961815*
PIGK	*0.68022248*	*7.069526276*	*5.109119928*	*6.16E-06*	*0.000804721*	*3.821074266*		*TC2N*	*-1.344225404*	*5.965810779*	*-12.56686404*	*1.20E-22*	*4.58E-20*	*40.9930944*
CSRNP1	*-1.078142215*	*7.320021991*	*-5.106192067*	*6.22E-06*	*0.000807412*	*3.811916038*		*CYTH4*	*1.172509149*	*6.3958471*	*12.56404678*	*1.22E-22*	*4.59E-20*	*40.979092*
TPTE2P3	*0.680897011*	*5.222049301*	*5.103750462*	*6.27E-06*	*0.000808821*	*3.804279726*		*PPP1CB*	*-0.828618676*	*7.801120331*	*-12.53731644*	*1.39E-22*	*5.14E-20*	*40.84620171*
TPPP3	*-0.605572988*	*7.467378926*	*-5.089485753*	*6.58E-06*	*0.000832466*	*3.759682713*		*SH2B3*	*0.824846541*	*6.412304242*	*12.53704046*	*1.40E-22*	*5.14E-20*	*40.84482934*
CRYZL1	*0.577422501*	*7.106080251*	*5.084936105*	*6.68E-06*	*0.000834816*	*3.745464893*		*SIPA1*	*0.656509596*	*7.030116697*	*12.52568009*	*1.48E-22*	*5.27E-20*	*40.78833178*
BBS5	*0.587129174*	*6.911440835*	*5.080811532*	*6.77E-06*	*0.000834816*	*3.732578037*		*IRF7*	*1.097941214*	*7.897516265*	*12.52422943*	*1.49E-22*	*5.27E-20*	*40.7811165*
PDLIM3	*-1.123347053*	*6.552005538*	*-5.08012578*	*6.79E-06*	*0.000834816*	*3.730435707*		*RHEBL1*	*0.814360287*	*6.041371386*	*12.52264067*	*1.50E-22*	*5.27E-20*	*40.77321417*
PDE10A	*-0.636248913*	*5.552366755*	*-5.075752801*	*6.89E-06*	*0.000834816*	*3.716775862*		*XLOC_l2_010245*	*-0.536150375*	*4.848353534*	*-12.51149985*	*1.59E-22*	*5.51E-20*	*40.71779454*
TMEM107	*0.625896739*	*6.968823828*	*5.073601885*	*6.94E-06*	*0.000835807*	*3.710058087*		*NAP1L5*	*-0.891758328*	*5.968826124*	*-12.47527472*	*1.91E-22*	*6.47E-20*	*40.53751916*
NPY5R	*0.900647241*	*7.362262285*	*5.057664852*	*7.32E-06*	*0.000854724*	*3.660304456*		*FGD3*	*1.412802561*	*6.320806507*	*12.45903991*	*2.07E-22*	*6.87E-20*	*40.45668934*
PARP12	*0.530004899*	*7.138490787*	*5.054993106*	*7.39E-06*	*0.000854724*	*3.651967242*		*MMP9*	*3.835084831*	*8.965966698*	*12.45131904*	*2.15E-22*	*7.07E-20*	*40.41824069*
SCML1	*0.710609358*	*6.588002414*	*5.054780061*	*7.39E-06*	*0.000854724*	*3.651302479*		*PLCB2*	*1.69168816*	*7.749004577*	*12.44499046*	*2.22E-22*	*7.22E-20*	*40.38672163*
OTUD4	*0.750625858*	*6.751737983*	*5.04290065*	*7.69E-06*	*0.00085929*	*3.614245941*		*ARMC7*	*0.557607138*	*5.750511367*	*12.41197978*	*2.63E-22*	*8.44E-20*	*40.22225871*
ICMT	*0.635703122*	*7.890576084*	*5.018965798*	*8.34E-06*	*0.000900926*	*3.539648612*		*PDGFB*	*0.966263498*	*5.602138426*	*12.39696006*	*2.83E-22*	*9.02E-20*	*40.14739811*
EXOSC3	*0.703656519*	*7.718147444*	*5.004662034*	*8.75E-06*	*0.000933851*	*3.495110376*		*PTPLA*	*-1.155098501*	*6.084161229*	*-12.39004763*	*2.93E-22*	*9.24E-20*	*40.11293907*
TMEM254	*0.509627576*	*7.812238291*	*5.003195599*	*8.79E-06*	*0.000933851*	*3.490546074*		*XLOC_l2_009273*	*0.825382355*	*5.176650234*	*12.36575045*	*3.32E-22*	*1.03E-19*	*39.99178367*
SHCBP1	*0.771167135*	*5.039297827*	*4.991848102*	*9.13E-06*	*0.00093888*	*3.455238239*		*CD163L1*	*1.397796183*	*5.969446097*	*12.34066303*	*3.77E-22*	*1.16E-19*	*39.86663596*
LYPLAL1	*0.879013828*	*6.876073327*	*4.984074445*	*9.37E-06*	*0.000955801*	*3.431062185*		*IFIH1*	*0.809079889*	*7.055333463*	*12.32797152*	*4.02E-22*	*1.23E-19*	*39.80330489*

USP41	0.663979712	7.605474456	4.974134099	9.69E-06	0.000963601	3.400161771		*MYO1F*	1.611865908	7.647533538	12.31929996	4.20E-22	1.27E-19	39.76002581
LOC100101478	0.633562982	5.304077846	4.973849584	9.70E-06	0.000963601	3.399277567		*LYN*	1.18434052	7.541903573	12.28271432	5.05E-22	1.51E-19	39.57736157
FAM200A	0.737967914	4.758236224	4.971394	9.78E-06	0.000963601	3.391646729		*LOC100127983*	-0.81687824	5.069783934	-12.27084518	5.37E-22	1.59E-19	39.51807799
PDIA3P1	0.590023848	10.66428848	4.970017144	9.82E-06	0.000963601	3.387368513		*RAD9A*	0.53767333	5.782336094	12.26444745	5.54E-22	1.63E-19	39.48611806
TIMM8A	0.592122304	6.380832389	4.965469672	9.97E-06	0.000963601	3.373240622		*DENND4B*	0.858948319	8.234919848	12.25722349	5.75E-22	1.67E-19	39.45002669
LOC102724428	-0.764098935	7.381244407	-4.965299423	9.98E-06	0.000963601	3.372711764		*SUB1*	-0.585871622	6.811382972	-12.25247613	5.89E-22	1.70E-19	39.42630626
ZNF33B	0.515643676	6.244256807	4.959108278	1.02E-05	0.000963601	3.353482943		*LASP1*	0.636913565	7.869937702	12.2460608	6.08E-22	1.74E-19	39.39424883
LOC400002	-0.527623935	5.916435974	-4.958187029	1.02E-05	0.000963601	3.350622206		*MPEG1*	1.358333303	6.52735325	12.24379278	6.15E-22	1.74E-19	39.38291476
ANAPC1P1	0.577533757	7.15588541	4.949954906	1.05E-05	0.000981228	3.325065325		*NAGPA*	0.866382976	7.270928859	12.21835479	7.00E-22	1.95E-19	39.25576381
GLDN	0.909276621	7.720859527	4.943358871	1.07E-05	0.000989625	3.304595769		*IL17D*	-1.286699854	5.84146046	-12.20025009	7.68E-22	2.09E-19	39.16523647
FAR1	0.501627421	8.695418968	4.941305257	1.08E-05	0.000991467	3.298224229		*TTLL11*	-0.70861572	5.157635144	-12.18945865	8.11E-22	2.19E-19	39.11126461
HUNK	-0.529008626	6.127654591	-4.924695644	1.14E-05	0.001033706	3.246717081		*ARHGAP9*	1.424090325	6.907551723	12.18452172	8.31E-22	2.23E-19	39.08657015
DCTN6	0.601275985	7.545146353	4.919388877	1.16E-05	0.001033706	3.230270308		*ASCL2*	1.168840558	5.678759257	12.18003964	8.50E-22	2.26E-19	39.06414921
FAM96A	0.827660137	7.486376257	4.90869783	1.21E-05	0.001054996	3.197151018		*ROR1*	-0.848375414	5.741470386	-12.17279688	8.82E-22	2.32E-19	39.02791495
IMPAD1	0.933884926	9.589870032	4.894716239	1.26E-05	0.001097109	3.153867567		*IBSP*	2.615474893	6.072925051	12.1697419	8.96E-22	2.34E-19	39.01263021
RCAN1	-0.50289102	7.905603068	-4.892970926	1.27E-05	0.001097109	3.148466885		*SNORD107*	-0.886005139	5.558082229	-12.16622955	9.12E-22	2.36E-19	38.99505624
EPPIN-WFDC6	-0.727969643	4.745323378	-4.889837165	1.28E-05	0.001097109	3.138771125		*KLF9*	-0.957639072	8.533598372	-12.16417968	9.22E-22	2.36E-19	38.98479928
MBD2	0.55121127	9.856977153	4.877335257	1.34E-05	0.001124137	3.100107609		*LAT2*	0.849692323	8.603776997	12.16291288	9.28E-22	2.36E-19	38.9784604
COMMD9	0.583073204	7.321512399	4.875417768	1.35E-05	0.001126603	3.094179983		*TECPR1*	0.662368669	6.819843557	12.15747149	9.54E-22	2.41E-19	38.95123119
CBWD6	0.559924493	7.929769394	4.868350575	1.38E-05	0.001141922	3.072338414		*GMFG*	1.030436847	9.481757157	12.14360082	1.02E-21	2.56E-19	38.88181054
C10orf120	-0.502277728	4.715490351	-4.867339003	1.38E-05	0.001141922	3.069212814		*BEX5*	-1.048311949	5.458497985	-12.12046875	1.15E-21	2.78E-19	38.76600453
SNORA13	0.558005454	4.63561415	4.866949895	1.39E-05	0.001141922	3.068010576		*LOC100630918*	-0.591095959	5.143008174	-12.11929633	1.16E-21	2.78E-19	38.76013394

MSMO1	0.946951944	7.061993051	4.866374823	1.39E-05	0.001141922	3.066233814		SH3KBP1	0.733610154	6.466629016	12.11652441	1.17E-21	2.78E-19	38.74625389
MYH11	-0.781117113	6.917951194	-4.857160051	1.43E-05	0.001153737	3.037771537		SLC16A7	-0.700254957	5.141636906	-12.11626992	1.18E-21	2.78E-19	38.74497951
GALNT1	0.605131424	7.905831761	4.850099438	1.47E-05	0.001157808	3.015973202		CD36	1.680178026	7.720804326	12.10627641	1.24E-21	2.91E-19	38.69493296
BIRC8	-0.534038219	4.877579256	-4.84162508	1.51E-05	0.001179829	2.98982202		LIG1	0.596909205	6.364122026	12.07736002	1.43E-21	3.34E-19	38.5500792
ZNF542P	0.566556421	6.515334342	4.825596206	1.59E-05	0.001207743	2.940393821		LINC00256A	0.559619354	4.62768891	12.0477582	1.67E-21	3.86E-19	38.40172583
BHMT2	0.585447875	9.555819491	4.824193739	1.60E-05	0.001207743	2.936071268		NDUFAF2	-0.572213192	7.294075417	-12.02530171	1.87E-21	4.29E-19	38.2891381
ZFP36L1	-0.500715172	9.168185845	-4.823008445	1.60E-05	0.001207743	2.932418348		ST7-AS1	-0.721940435	5.426330176	-12.01333025	1.98E-21	4.53E-19	38.22910257
CALCB	-0.629397983	5.951528524	-4.815005744	1.65E-05	0.001207743	2.907761849		NCF1	1.390162106	6.153464716	12.00853736	2.03E-21	4.60E-19	38.20506381
CLEC2B	0.55125712	6.389727196	4.810101875	1.67E-05	0.001216543	2.892658774		TRAF3IP3	1.482314134	6.043117591	11.98245828	2.32E-21	5.21E-19	38.07423405
ERLIN1	0.610942062	8.690986643	4.804218527	1.71E-05	0.001231566	2.874544966		C9orf167	1.012733275	5.748336599	11.98143854	2.33E-21	5.21E-19	38.06911733
SIPA1L1	0.509963766	7.681482852	4.779689691	1.85E-05	0.001276821	2.799094952		CCDC154	0.718437291	5.96479251	11.97790058	2.38E-21	5.26E-19	38.0513645
TPT1	-0.598770775	8.935365365	-4.779497687	1.85E-05	0.001276821	2.798504805		FSCN1	0.980725729	8.684620582	11.96124055	2.59E-21	5.69E-19	37.96775505
MAT2B	0.525255579	8.031946753	4.773530657	1.89E-05	0.001297833	2.780167857		HLA-J	0.990083675	10.33288907	11.93419093	2.97E-21	6.48E-19	37.83196173
SNORD43	-0.744013166	7.316335878	-4.767952511	1.92E-05	0.001308537	2.763032136		ARHGDIB	0.914598081	9.749983031	11.92233582	3.15E-21	6.83E-19	37.77243052
NFASC	-0.515312342	6.47436779	-4.763257065	1.95E-05	0.001311367	2.748612657		MAP3K11	0.727496499	8.165733146	11.92108991	3.17E-21	6.83E-19	37.76617353
GXYLT1	0.556512432	6.42275098	4.762068812	1.96E-05	0.001311367	2.744964267		CDC37L1	-0.572300015	5.959058009	-11.91362287	3.30E-21	7.05E-19	37.72867143
SCYL2	0.669697511	8.288509815	4.76019077	1.97E-05	0.001311367	2.739198515		INPP5A	-0.753683417	5.881824489	-11.91001483	3.36E-21	7.08E-19	37.71054917
POGLUT1	0.647001601	6.350358602	4.756684633	2.00E-05	0.001315395	2.728436201		ZNF837	0.501364047	5.876446773	11.89637223	3.60E-21	7.54E-19	37.64201743
JUND	-0.796674992	8.533094769	-4.755753598	2.00E-05	0.001315395	2.725578733		KLHDC5	-0.708208543	6.026577053	-11.89369722	3.65E-21	7.56E-19	37.62857833
P2RY6	-0.501103918	6.538707305	-4.746156555	2.07E-05	0.001324236	2.696133967		FAM71E1	-1.359202915	6.504394385	-11.89298456	3.66E-21	7.56E-19	37.62499786
HPF1	0.559727302	6.24689129	4.745041183	2.08E-05	0.001324236	2.692713053		PI16	-2.841941362	6.843075063	-11.89182349	3.69E-21	7.56E-19	37.6191645
FAM177A1	0.847959199	6.968135487	4.732981587	2.16E-05	0.00135105	2.655741157		GMIP	1.246988505	6.634147288	11.86380968	4.25E-21	8.66E-19	37.47839051

KLF6	-0.739198657	9.146328478	-4.731495958	2.17E-05	0.00135105	2.651188555		*ABHD6*	0.668460897	6.278477041	11.848433	4.60E-21	9.24E-19	37.40109672
CNTN4	-0.754474409	4.632928432	-4.728841229	2.19E-05	0.001351329	2.643054416		*MYEF2*	-1.027274726	5.806303002	-11.82664309	5.14E-21	1.03E-18	37.29153741
KRCC1	0.524410411	7.157333718	4.718796968	2.26E-05	0.001375675	2.612291313		*SYNC*	-1.078749242	7.272022205	-11.82485533	5.19E-21	1.03E-18	37.28254715
VIPR2	-0.653678134	5.366856627	-4.714327362	2.30E-05	0.001383507	2.598608495		*HSPB8*	-0.750241993	5.365502964	-11.80979081	5.60E-21	1.10E-18	37.20678192
SGPP2	-0.585311359	5.118914578	-4.711614738	2.32E-05	0.001391757	2.590306289		*RNF125*	0.899991743	6.317264818	11.78839079	6.25E-21	1.22E-18	37.09912632
MYO5B	-0.726321626	5.500684993	-4.710112967	2.33E-05	0.001394093	2.585710633		*DUSP18*	0.579483431	5.10468658	11.77478243	6.70E-21	1.29E-18	37.03065152
SIMC1	-0.665392128	6.000051607	-4.705710629	2.36E-05	0.00139693	2.572241408		*ATG16L2*	1.104375421	8.292786499	11.77385846	6.73E-21	1.29E-18	37.02600179
AADAT	0.557082397	5.837196928	4.705446344	2.37E-05	0.00139693	2.571432937		*PARP12*	0.933702443	6.78812238	11.76530559	7.03E-21	1.33E-18	36.98295835
FAM206A	0.630778772	6.924892997	4.705078118	2.37E-05	0.00139693	2.570306525		*SNORD116-26*	-0.623432195	4.779824849	-11.75286036	7.49E-21	1.41E-18	36.92031732
CCDC3	-1.04403235	8.808136142	-4.699727976	2.41E-05	0.00141346	2.553943403		*AEBP2*	-0.602684537	6.599926797	-11.73941557	8.02E-21	1.49E-18	36.85263351
ZNF529	0.518434404	6.540976632	4.695128835	2.45E-05	0.001414321	2.539881829		*CNTN4*	-1.034055447	4.753995315	-11.73672004	8.13E-21	1.50E-18	36.8390622
PIGH	0.515384781	4.452413937	4.683576065	2.54E-05	0.001435031	2.504579064		*H3F3B*	-0.586086219	8.741501392	-11.73339716	8.27E-21	1.51E-18	36.82233171
ZNF613	0.511359357	6.193926525	4.683468933	2.54E-05	0.001435031	2.504251819		*TRERF1*	0.597742677	5.22758821	11.7297396	8.43E-21	1.53E-18	36.80391529
GSG1L	-0.509130874	6.937444844	-4.665527961	2.70E-05	0.001469955	2.449483068		*ARID3A*	0.799840309	5.763595392	11.72458043	8.65E-21	1.56E-18	36.77793647
HIST1H4C	-1.077496905	8.547431527	-4.663047999	2.72E-05	0.001473332	2.441917706		*UNC13D*	0.828088191	5.305167239	11.7220029	8.77E-21	1.58E-18	36.76495674
PMM2	0.65872533	7.06484827	4.662356655	2.73E-05	0.001473332	2.439808925		*LOC100652963*	-1.390479112	6.843525614	-11.72016813	8.85E-21	1.58E-18	36.75571712
CCNC	0.607540656	8.488739202	4.659137571	2.76E-05	0.0014753	2.429991188		*GIMAP1*	0.94481075	6.801532742	11.70902118	9.37E-21	1.66E-18	36.69957781
CSTF3	0.603562521	7.254277353	4.654666342	2.80E-05	0.0014753	2.416358205		*NFAM1*	1.684392122	6.726771289	11.69228442	1.02E-20	1.80E-18	36.61527137
NIPSNAP3B	0.807317124	7.271198076	4.654381516	2.80E-05	0.0014753	2.4154899		*ZBTB46*	0.548298245	5.007418506	11.68590271	1.05E-20	1.85E-18	36.58312061
ARF4	0.587452139	10.39800865	4.653176132	2.81E-05	0.0014753	2.411815418		*HCST*	1.335074588	8.691419758	11.68230694	1.07E-20	1.88E-18	36.56500409
MR1	0.63183528	7.250138371	4.65065987	2.83E-05	0.0014753	2.404145863		*FGF2*	-0.692165709	4.952455717	-11.67072991	1.14E-20	1.98E-18	36.5066701
AKTIP	0.567887917	8.02366943	4.649027071	2.85E-05	0.001479389	2.39916981		*SPG20*	-0.68671351	5.888566392	-11.64418441	1.31E-20	2.25E-18	36.37288102

SNORD83B	-0.515926503	7.189155676	-4.646640816	2.87E-05	0.001484544	2.391898575		*GNAI2*	0.515237895	11.14931538	11.6426027	1.32E-20	2.26E-18	36.3649078
EGFL6	2.528246594	9.532033221	4.64350443	2.90E-05	0.001491197	2.382343425		*FCGR3A*	1.678062358	8.751667539	11.62834622	1.42E-20	2.40E-18	36.29303532
KCNA2	0.65428559	6.213803155	4.635295468	2.98E-05	0.001508497	2.35734434		*C21orf96*	0.990358239	5.277751044	11.61733954	1.50E-20	2.51E-18	36.23753757
NT5C3A	0.580647965	6.302587871	4.629569156	3.04E-05	0.001521727	2.339914295		*INPP5D*	1.195325876	7.585780333	11.60993164	1.56E-20	2.60E-18	36.2001813
GPR1	-0.744634009	4.775433514	-4.625060301	3.08E-05	0.001524458	2.326194961		*NUP62*	0.609849645	8.517250125	11.57045092	1.90E-20	3.14E-18	36.00103197
CITED2	-0.579734905	9.066898062	-4.624809539	3.09E-05	0.001524458	2.325432083		*CCR5*	1.61908754	6.958077276	11.56824464	1.92E-20	3.16E-18	35.98990017
ZFP14	0.532905948	5.337099517	4.622956758	3.10E-05	0.001524578	2.319795896		*HLA-E*	0.688708727	9.974776206	11.56107636	2.00E-20	3.25E-18	35.95373058
MIR224	0.908390367	8.126838587	4.621059512	3.12E-05	0.001530321	2.314025215		*LPAR1*	-0.820345706	6.543125661	-11.55771029	2.03E-20	3.28E-18	35.93674502
ABCB4	0.517440111	5.527280946	4.619693738	3.14E-05	0.001532836	2.309871546		*IPW*	-0.56299074	5.380663733	-11.55368017	2.07E-20	3.33E-18	35.91640772
SCN7A	-0.640263678	4.315637967	-4.608025041	3.26E-05	0.001566466	2.274400541		*C22orf34*	0.604247125	4.777668639	11.543341	2.19E-20	3.46E-18	35.86422834
RP9	0.514439981	6.982389597	4.598348456	3.37E-05	0.00160538	2.245007777		*PCYOX1L*	0.57213075	6.423498821	11.52337126	2.42E-20	3.81E-18	35.76342759
MIR141	-0.638291091	5.712592483	-4.597503706	3.37E-05	0.001605814	2.242442814		*PIK3CD*	0.586490726	5.197967041	11.49776632	2.76E-20	4.33E-18	35.63414744
DND1	-0.650394826	6.548445175	-4.596329328	3.39E-05	0.001605814	2.238877238		*SNURF*	-0.649043523	7.746795048	-11.46809248	3.21E-20	4.98E-18	35.48427513
OR10K2	-0.627136461	4.60142566	-4.595366588	3.40E-05	0.001605814	2.235954456		*DUSP6*	0.882882387	8.592617181	11.4675243	3.22E-20	4.98E-18	35.48140493
KLRA1P	0.507234016	4.829880526	4.592137995	3.43E-05	0.001619064	2.226154254		*PYCARD*	1.288933995	8.005819806	11.46166558	3.32E-20	5.10E-18	35.45180833
LOC100128233	-0.515433513	4.442033394	-4.586352893	3.50E-05	0.001638453	2.208599689		*VAV3*	1.196287642	6.313334148	11.45322312	3.47E-20	5.27E-18	35.40915598
MIR23A	-0.719529447	7.396456773	-4.567572326	3.72E-05	0.001706348	2.151662473		*RNF135*	0.815406452	7.762018393	11.44944858	3.54E-20	5.35E-18	35.39008518
RHD	0.574748676	5.762044041	4.566776972	3.73E-05	0.001706878	2.149252935		*DKFZp451A211*	-1.137178689	5.121902156	-11.44416754	3.63E-20	5.47E-18	35.3634015
ALS2CR11	0.574485533	4.852898848	4.565794234	3.74E-05	0.001708464	2.146275911		*MNDA*	1.282572266	6.58062784	11.42559575	4.00E-20	5.93E-18	35.26955054
MIR21	-1.825815879	7.682501017	-4.563501375	3.77E-05	0.001709613	2.139330964		*PACSIN3*	-0.786528375	4.802198928	-11.4244422	4.02E-20	5.94E-18	35.26372055
DRG1	0.535175615	7.94505588	4.553576946	3.90E-05	0.001733486	2.109284105		*C1orf93*	1.016801419	7.20411664	11.41003771	4.33E-20	6.30E-18	35.19091437
SNORA15	0.511494192	5.500039825	4.553039279	3.90E-05	0.001733486	2.107656923		*LAYN*	-0.804955183	6.599696551	-11.40532735	4.44E-20	6.42E-18	35.16710377

MFSD5	*0.54555176*	*6.392205347*	*4.552231846*	*3.91E-05*	*0.001733486*	*2.105213448*		*DPP4*	*1.43026052*	*5.717448355*	*11.4028271*	*4.49E-20*	*6.47E-18*	*35.15446466*
RAP1B	*0.661270883*	*9.757681869*	*4.550941219*	*3.93E-05*	*0.001733486*	*2.101308027*		*LST1*	*1.241196786*	*7.259030666*	*11.40032057*	*4.55E-20*	*6.53E-18*	*35.14179348*
PMS2	*0.514715458*	*7.737750911*	*4.547184712*	*3.98E-05*	*0.001733486*	*2.089943047*		*SLC22A3*	*-0.961365251*	*5.278708245*	*-11.37524531*	*5.18E-20*	*7.34E-18*	*35.01501192*
DUSP1	*-1.701292824*	*10.33292583*	*-4.54698206*	*3.98E-05*	*0.001733486*	*2.089330033*		*ENAH*	*-0.58342334*	*4.552373736*	*-11.36692454*	*5.40E-20*	*7.60E-18*	*34.97293416*
GLB1	*0.741025153*	*8.087891344*	*4.543297632*	*4.03E-05*	*0.001733486*	*2.078186427*		*GUCA2B*	*-1.296697664*	*5.947159741*	*-11.35259576*	*5.81E-20*	*8.15E-18*	*34.90046537*
WARS	*0.691749744*	*8.66992079*	*4.540973867*	*4.06E-05*	*0.001733486*	*2.071159761*		*FCHO1*	*1.025688047*	*6.124078695*	*11.34684954*	*5.99E-20*	*8.35E-18*	*34.87140034*
NUP43	*0.529614477*	*8.004269806*	*4.537517868*	*4.11E-05*	*0.00174558*	*2.060711713*		*TSPAN33*	*0.836137049*	*5.776164206*	*11.34612305*	*6.01E-20*	*8.35E-18*	*34.86772552*
ANAPC4	*0.537210189*	*7.621472958*	*4.535194937*	*4.14E-05*	*0.001752092*	*2.053690654*		*ITGAL*	*1.314314077*	*5.837744992*	*11.3262306*	*6.66E-20*	*9.16E-18*	*34.76709294*
RPP38	*0.507122002*	*6.055253875*	*4.533870252*	*4.16E-05*	*0.001755963*	*2.049687353*		*CCL3*	*2.591214954*	*9.172498809*	*11.32056429*	*6.85E-20*	*9.39E-18*	*34.73842417*
FAM26E	*0.647426865*	*8.094525176*	*4.531698415*	*4.18E-05*	*0.001757025*	*2.043124765*		*ARL5A*	*-0.50428764*	*6.72049591*	*-11.31808051*	*6.94E-20*	*9.47E-18*	*34.72585692*
FAR2	*0.66353349*	*7.246926596*	*4.531104528*	*4.19E-05*	*0.001757025*	*2.041330421*		*CENPBD1*	*0.571050158*	*6.051312676*	*11.31550492*	*7.03E-20*	*9.55E-18*	*34.71282481*
EFTUD1P1	*0.679099081*	*6.713655328*	*4.530292963*	*4.20E-05*	*0.001757998*	*2.038878529*		*MT1M*	*-1.299423087*	*8.276757287*	*-11.31191469*	*7.16E-20*	*9.68E-18*	*34.69465818*
TMOD1	*-0.660548079*	*7.943080205*	*-4.52886036*	*4.22E-05*	*0.001762548*	*2.034550728*		*CTPS*	*-0.636431605*	*7.057266953*	*-11.30941522*	*7.26E-20*	*9.77E-18*	*34.68201042*
ADGRD2	*-0.543664795*	*7.279600363*	*-4.525166909*	*4.27E-05*	*0.001767067*	*2.02339525*		*MRPL33*	*-0.505182993*	*8.278655975*	*-11.29738425*	*7.72E-20*	*1.03E-17*	*34.621127*
NABP1	*0.631235402*	*8.821502207*	*4.524892004*	*4.28E-05*	*0.001767067*	*2.022565072*		*XLOC_009582*	*-0.643289703*	*4.629361925*	*-11.28441576*	*8.25E-20*	*1.10E-17*	*34.55549079*
PCDHB13	*0.514714419*	*5.296251553*	*4.519898692*	*4.35E-05*	*0.001774085*	*2.007488927*		*LRMP*	*0.951046771*	*6.021132409*	*11.28241281*	*8.33E-20*	*1.11E-17*	*34.54535267*
ARPP19	*0.514098101*	*8.755635198*	*4.517957179*	*4.38E-05*	*0.001781715*	*2.001628549*		*CHKA*	*0.656547775*	*6.563130936*	*11.2730177*	*8.75E-20*	*1.16E-17*	*34.49779574*
DCUN1D5	*0.513693508*	*8.121043389*	*4.509729472*	*4.50E-05*	*0.001811691*	*1.976803327*		*KCNQ1*	*0.630192189*	*5.348665235*	*11.27195542*	*8.79E-20*	*1.16E-17*	*34.49241832*
NEK7	*0.542466955*	*10.44467527*	*4.507880866*	*4.52E-05*	*0.001818968*	*1.971227763*		*MAFB*	*1.369671253*	*8.584077825*	*11.26217572*	*9.25E-20*	*1.21E-17*	*34.44290932*
NNMT	*-0.656324477*	*9.146557249*	*-4.503233749*	*4.59E-05*	*0.001835633*	*1.957215191*		*PRRX2*	*-0.82107815*	*6.883114033*	*-11.25707521*	*9.49E-20*	*1.24E-17*	*34.4170864*
MIR133B	*-0.52703902*	*6.621248473*	*-4.501262162*	*4.62E-05*	*0.001843769*	*1.951271752*		*ACP5*	*3.190751113*	*9.542937537*	*11.25667848*	*9.51E-20*	*1.24E-17*	*34.41507779*
HMHB1	*-0.511667598*	*7.108009574*	*-4.48074519*	*4.94E-05*	*0.001936289*	*1.889477144*		*SCARA5*	*-1.517923779*	*5.625843721*	*-11.25322169*	*9.68E-20*	*1.25E-17*	*34.39757593*

STK17A	*0.572723909*	*7.767063161*	*4.477251262*	*5.00E-05*	*0.001947136*	*1.878963876*		*XAF1*	*0.86887213*	*6.468503463*	*11.25318406*	*9.68E-20*	*1.25E-17*	*34.39738543*
ZNF492	*0.566865536*	*5.817656997*	*4.468245911*	*5.15E-05*	*0.001973837*	*1.851880221*		*XLOC_000983*	*-0.777660977*	*5.076644354*	*-11.24956347*	*9.87E-20*	*1.27E-17*	*34.37905357*
OR56A4	*-0.574292207*	*5.378651165*	*-4.4665042*	*5.17E-05*	*0.001973837*	*1.846644273*		*CFL2*	*-1.139629459*	*6.300631971*	*-11.2468272*	*1.00E-19*	*1.28E-17*	*34.36519883*
RAP1A	*0.522687542*	*8.739530165*	*4.46150419*	*5.26E-05*	*0.00199394*	*1.831617281*		*TNFAIP8L2*	*1.398064774*	*6.24869625*	*11.23966577*	*1.04E-19*	*1.32E-17*	*34.32893603*
PYROXD1	*0.73047927*	*7.17576482*	*4.460883587*	*5.27E-05*	*0.00199394*	*1.82975255*		*XLOC_l2_012953*	*0.989207933*	*10.6701874*	*11.23955565*	*1.04E-19*	*1.32E-17*	*34.32837838*
PI16	*-0.820986355*	*8.439581902*	*-4.454323883*	*5.38E-05*	*0.002018798*	*1.810048274*		*PSMB10*	*0.630912738*	*9.219523083*	*11.2311491*	*1.08E-19*	*1.37E-17*	*34.28580747*
CXorf21	*0.716890488*	*5.377834557*	*4.45328722*	*5.40E-05*	*0.002020533*	*1.80693527*		*RGS19*	*0.994689603*	*7.601669002*	*11.21977023*	*1.15E-19*	*1.44E-17*	*34.22817912*
PECAM1	*0.699107428*	*8.266161323*	*4.451529605*	*5.43E-05*	*0.002022015*	*1.801657911*		*LINC00312*	*-1.436963368*	*6.859078644*	*-11.21651473*	*1.17E-19*	*1.46E-17*	*34.21169047*
POLR2K	*0.527391929*	*9.076026591*	*4.445205008*	*5.54E-05*	*0.002039589*	*1.782674144*		*IKZF1*	*0.871132085*	*5.738936255*	*11.20068288*	*1.27E-19*	*1.58E-17*	*34.13149691*
EGR1	*-2.126221825*	*8.873232071*	*-4.443341387*	*5.58E-05*	*0.002039589*	*1.777082213*		*SAP25*	*1.553273477*	*7.669801743*	*11.19599506*	*1.30E-19*	*1.61E-17*	*34.10774923*
C11orf71	*0.537610863*	*6.387965847*	*4.435614055*	*5.72E-05*	*0.002071275*	*1.753904932*		*BBC3*	*0.611605709*	*7.332275237*	*11.19268534*	*1.32E-19*	*1.62E-17*	*34.09098211*
PQLC2L	*0.630439216*	*8.384436313*	*4.432926846*	*5.77E-05*	*0.002080475*	*1.745848407*		*AMDHD2*	*0.530289271*	*6.313327075*	*11.19003518*	*1.34E-19*	*1.63E-17*	*34.07755596*
GABARAP	*0.554719014*	*9.420254011*	*4.432568789*	*5.78E-05*	*0.002080475*	*1.744775049*		*SLC38A7*	*0.916526579*	*6.859128952*	*11.18937983*	*1.34E-19*	*1.63E-17*	*34.0742358*
NLRP3	*-0.703578595*	*6.782200277*	*-4.423059644*	*5.96E-05*	*0.002111366*	*1.716280907*		*PARP14*	*0.73464366*	*7.299595047*	*11.18803925*	*1.35E-19*	*1.63E-17*	*34.06744403*
TMEM144	*0.55116556*	*7.153658897*	*4.418758468*	*6.04E-05*	*0.00212965*	*1.703399846*		*PTPN18*	*0.662597238*	*6.889861114*	*11.17389871*	*1.46E-19*	*1.73E-17*	*33.99579873*
FBXL13	*-0.519140467*	*4.373934485*	*-4.410867166*	*6.20E-05*	*0.002169441*	*1.679779219*		*DCAF8L2*	*-0.656340664*	*4.223698767*	*-11.16014135*	*1.56E-19*	*1.84E-17*	*33.92608574*
CCDC140	*-0.523912122*	*5.947689262*	*-4.407221825*	*6.27E-05*	*0.0021762*	*1.668873096*		*ADH5*	*-0.830882746*	*6.858681332*	*-11.1597416*	*1.57E-19*	*1.84E-17*	*33.92405995*
TPRG1	*0.796803729*	*8.097963904*	*4.406026371*	*6.29E-05*	*0.0021762*	*1.665297271*		*CLEC4A*	*0.932756938*	*6.137946859*	*11.15903891*	*1.57E-19*	*1.84E-17*	*33.92049896*
FAM198B	*0.721058587*	*9.520562005*	*4.400970213*	*6.40E-05*	*0.002194523*	*1.650177356*		*ABCA7*	*1.016710099*	*5.674866148*	*11.15009714*	*1.64E-19*	*1.92E-17*	*33.87518291*
C12orf75	*-0.596779654*	*5.831221017*	*-4.390822787*	*6.61E-05*	*0.002243961*	*1.619852113*		*SNHG13*	*-0.549020199*	*5.975007867*	*-11.14772669*	*1.66E-19*	*1.93E-17*	*33.8631691*
GPR6	*-0.53091113*	*7.260841917*	*-4.390225955*	*6.62E-05*	*0.002244482*	*1.618069317*		*HLA-G*	*0.84961483*	*11.26690739*	*11.14385496*	*1.70E-19*	*1.96E-17*	*33.84354593*
SLC35A1	*0.552173047*	*7.880253288*	*4.382079675*	*6.80E-05*	*0.002277301*	*1.593744695*		*SPI1*	*1.49911991*	*7.401585812*	*11.14345948*	*1.70E-19*	*1.96E-17*	*33.8415415*

CETN3	0.626672685	5.596604704	4.380917374	6.83E-05	0.002280705	1.590275476		*CCDC88B*	0.731604145	5.556369709	11.13657261	1.76E-19	2.03E-17	33.80663484
LOC653653	0.564367319	7.757143087	4.376430053	6.93E-05	0.002296139	1.576885044		*COTL1*	1.131994357	7.891066435	11.12423331	1.88E-19	2.13E-17	33.74408666
CREB5	-0.508468491	6.94477552	-4.362625881	7.24E-05	0.002354382	1.535725202		*UAP1*	-1.098047418	6.364931262	-11.11608069	1.96E-19	2.21E-17	33.70275708
EDF1	-0.669287231	9.426814125	-4.359558385	7.31E-05	0.002361883	1.526585581		*FLI1*	0.71522211	6.735654975	11.11232738	2.00E-19	2.24E-17	33.68372871
RGS4	-0.705950489	6.405634225	-4.358785542	7.33E-05	0.002363955	1.52428328		*DCUN1D4*	-0.588415943	5.777317903	-11.11200789	2.00E-19	2.24E-17	33.68210892
ADAMTSL3	-0.716327636	6.923566734	-4.356829213	7.38E-05	0.002367452	1.518456063		*IRF9*	0.54816466	7.31580861	11.09590724	2.17E-19	2.41E-17	33.60047517
FAM114A2	0.547747291	7.293731167	4.352919276	7.47E-05	0.002373513	1.506812732		*CTSC*	0.753005728	6.64268069	11.09362047	2.20E-19	2.43E-17	33.58887982
TRIM16L	0.633434501	8.406807231	4.351584282	7.50E-05	0.002373513	1.502838195		*C22orf29*	0.511527223	6.795353102	11.08794702	2.26E-19	2.49E-17	33.56011086
SNRPE	0.57846507	6.767328088	4.350658506	7.53E-05	0.002376841	1.500082253		*H2AFJ*	-0.544476685	7.049306457	-11.08316508	2.32E-19	2.54E-17	33.53586148
OGN	-1.115648826	8.568357735	-4.347229783	7.61E-05	0.002395665	1.489877252		*GALNT6*	1.411955261	5.802832533	11.08104237	2.35E-19	2.56E-17	33.52509682
RPL13AP3	-0.550361777	7.073625648	-4.342184996	7.74E-05	0.002412103	1.474867938		*SNORD116-6*	-0.934415826	5.545391543	-11.07697947	2.40E-19	2.60E-17	33.50449257
TAF1B	0.554198536	7.465348605	4.340630403	7.77E-05	0.002420428	1.470244046		*VGLL3*	-0.804456493	6.222568891	-11.05961453	2.62E-19	2.83E-17	33.41642127
PF4	1.00399178	6.064539355	4.325436517	8.16E-05	0.00249709	1.425085948		*AIM1*	0.898335658	5.680415766	11.05155099	2.73E-19	2.93E-17	33.3755203
PPIP5K1	0.502298365	7.5320611	4.325180068	8.17E-05	0.00249709	1.424324276		*ZEB1*	-0.856140765	7.592148987	-11.05065247	2.74E-19	2.93E-17	33.37096253
LOC728323	1.071555431	8.05331029	4.31955083	8.32E-05	0.00253493	1.407609482		*LONRF2*	-1.032139652	5.144525109	-11.04744876	2.79E-19	2.96E-17	33.35471135
SOCS4	0.604119167	6.727007255	4.319002395	8.33E-05	0.002535537	1.405981476		*PLCG2*	0.763247404	5.849480936	11.04519963	2.82E-19	2.98E-17	33.34330209
LINC00917	-1.14066094	5.815031035	-4.311275774	8.54E-05	0.002567989	1.38305393		*NPR3*	-0.880307614	5.148844396	-11.03921317	2.91E-19	3.07E-17	33.31293332
PAK1IP1	0.52012801	7.010393826	4.301563606	8.81E-05	0.002613544	1.354257409		*LOC100506948*	-0.711811186	6.059897081	-11.03796327	2.93E-19	3.07E-17	33.30659251
ID2	0.544887332	7.49453296	4.301148453	8.83E-05	0.002613544	1.353027049		*LINC00256B*	0.721646608	5.283405541	11.03623533	2.95E-19	3.08E-17	33.29782647
RPS15A	-0.574503964	10.44405633	-4.28476573	9.30E-05	0.002679797	1.304512266		*SYK*	1.528836821	6.851162607	11.03394538	2.99E-19	3.11E-17	33.28620911
SCOC	0.549298277	6.685837894	4.284222617	9.32E-05	0.002679797	1.302905181		*NECAP2*	0.654815778	7.921712768	11.02729412	3.09E-19	3.18E-17	33.25246461
RPP40	0.506878412	5.655743575	4.283339277	9.34E-05	0.002679797	1.300291528		*FSTL3*	-1.116263023	7.307099966	-11.0251009	3.13E-19	3.21E-17	33.24133714

NDUFB4	0.646810397	8.395714653	4.280686391	9.42E-05	0.002694939	1.292443376		TOM1L1	-0.533993577	5.584325589	-11.01968037	3.22E-19	3.29E-17	33.2138348
ZNF506	0.590521275	8.016125171	4.277246923	9.53E-05	0.002713149	1.282271125		CCL4	2.236318462	7.850494474	11.01404023	3.31E-19	3.36E-17	33.1852169
NR4A1	-1.197691298	7.835694285	-4.266551651	9.86E-05	0.002779934	1.250660638		MXRA5	0.833519875	5.425070881	11.00877693	3.40E-19	3.43E-17	33.1585099
DDIT3	0.830375417	7.341352754	4.261950808	0.000100065	0.00280533	1.237072332		LOC100131541	0.587538143	5.873753995	11.0087657	3.40E-19	3.43E-17	33.1584529
CHSY1	-0.524200775	7.871580301	-4.259165809	0.00010096	0.002816536	1.228849871		USP53	-0.789775042	6.576165588	-11.00829952	3.41E-19	3.43E-17	33.15608737
CDKN1A	-0.676529762	7.708175008	-4.252084963	0.000103272	0.002863113	1.207954054		RRP15	-0.543517778	4.869502609	-11.00724886	3.43E-19	3.44E-17	33.15075597
RPS14	-0.636582311	10.45398369	-4.241742296	0.000106741	0.00292137	1.177457741		LMCD1	-1.07440471	8.975654794	-11.00375425	3.49E-19	3.49E-17	33.13302281
TMEM267	0.514430624	6.561614303	4.241040453	0.000106981	0.00292137	1.175389382		LOC100505905	-0.741449382	4.605491072	-11.00260158	3.51E-19	3.50E-17	33.12717353
FOS	-3.144174287	8.946902215	-4.238189895	0.000107959	0.002939203	1.166990107		IL10RA	1.468073164	8.266559528	10.99982281	3.56E-19	3.53E-17	33.11307236
FLJ16734	-0.509185442	5.971734496	-4.237700238	0.000108128	0.002939203	1.165547544		XLOC_005062	-0.801889848	4.449949671	-10.99861417	3.59E-19	3.55E-17	33.1069389
RPS27	-0.64225578	11.29869315	-4.237516403	0.000108192	0.002939203	1.165005974		MYOC	-2.901229111	6.717146011	-10.99716139	3.61E-19	3.56E-17	33.09956638
ATP5I	-0.549533695	9.031982733	-4.237002231	0.000108369	0.002939203	1.163491289		CDH19	-1.114557896	4.688956465	-10.99566266	3.64E-19	3.58E-17	33.09196063
RPL17	0.613630829	6.7450927	4.225415547	0.000112451	0.002979586	1.129378274		CEP70	-0.55899898	5.305542142	-10.99411917	3.67E-19	3.58E-17	33.08412763
GTF3C6	0.646067093	8.243976207	4.223465855	0.000113152	0.002991791	1.123641828		CITED4	-0.726418666	7.915424326	-10.98203318	3.91E-19	3.80E-17	33.0227895
TRAPPC6B	0.567206966	7.972179848	4.222774276	0.000113402	0.002994435	1.121607302		DTX3L	0.574997486	6.159431619	10.98009021	3.94E-19	3.83E-17	33.01292807
RPS9	-0.566768069	10.90556798	-4.22082221	0.00011411	0.003005189	1.115865342		BARD1	-0.51855657	5.673267236	-10.97873099	3.97E-19	3.84E-17	33.0060294
LOC100131581	-0.582690133	5.105700469	-4.213740618	0.000116714	0.003041725	1.095044143		TSPAN4	0.663927146	7.762082539	10.97499578	4.05E-19	3.90E-17	32.98707099
FNDC1	-0.778746615	7.023630068	-4.211060218	0.000117715	0.003050791	1.087167017		FCGRT	0.9864265	9.876223765	10.97345625	4.08E-19	3.92E-17	32.97925681
BTF3L4	0.598769239	8.344340921	4.184717506	0.000128007	0.003170779	1.00986189		PTAFR	0.792007058	6.99990045	10.96195587	4.33E-19	4.13E-17	32.9208814
ADAM32	0.556574207	6.530630925	4.184707149	0.000128011	0.003170779	1.009831535		CORO1A	1.042662209	6.718224107	10.95148593	4.57E-19	4.33E-17	32.86773193
ATF3	-1.092191422	7.2240461	-4.183869192	0.000128353	0.00317194	1.007375788		DTYMK	0.50161483	7.70166229	10.95119271	4.58E-19	4.33E-17	32.86624334
LOC1720	0.884797833	8.210281096	4.175590636	0.000131773	0.003220831	0.983125399		C6orf26	0.640913869	5.338073264	10.94030847	4.84E-19	4.54E-17	32.81098612

CRIPT	*0.608710653*	*8.498168288*	*4.174006473*	*0.000132438*	*0.003228412*	*0.978487201*		*CLIC4*	*-0.678126147*	*8.100189413*	*-10.93770234*	*4.91E-19*	*4.58E-17*	*32.79775461*
MIRLET7G	*0.523714239*	*5.403054591*	*4.173374258*	*0.000132704*	*0.003230411*	*0.976636373*		*SMPD3*	*0.965646611*	*5.007089006*	*10.90604067*	*5.78E-19*	*5.36E-17*	*32.63698549*
LOC101060256	*-0.764835039*	*8.001194877*	*-4.168009585*	*0.000134983*	*0.003260302*	*0.960935887*		*HIGD1A*	*-0.509071546*	*6.225330125*	*-10.89336205*	*6.17E-19*	*5.71E-17*	*32.57259653*
ACTG2	*-0.559933571*	*6.148715537*	*-4.166639706*	*0.000135571*	*0.003268918*	*0.956928102*		*DLEC1*	*0.538325678*	*4.659937923*	*10.89268457*	*6.19E-19*	*5.71E-17*	*32.56915577*
PTTG3P	*0.562356799*	*4.365011817*	*4.164043073*	*0.000136693*	*0.003288029*	*0.949332787*		*GUSB*	*0.792519932*	*8.845914006*	*10.88671791*	*6.38E-19*	*5.85E-17*	*32.53885159*
MRPS18C	*0.516331545*	*5.069932446*	*4.155890032*	*0.000140273*	*0.003345972*	*0.925497625*		*P2RY13*	*0.787571319*	*4.537082317*	*10.88583061*	*6.41E-19*	*5.85E-17*	*32.53434499*
HIGD1A	*0.523551846*	*8.80301608*	*4.155398631*	*0.000140492*	*0.003347194*	*0.924061661*		*CX3CR1*	*1.393867172*	*6.709644324*	*10.88206294*	*6.53E-19*	*5.94E-17*	*32.51520851*
RAB1C	*0.67282542*	*7.63636011*	*4.15200589*	*0.000142011*	*0.00337133*	*0.914149411*		*STBD1*	*-0.627941825*	*5.456506699*	*-10.88171756*	*6.55E-19*	*5.94E-17*	*32.51345427*
GPLD1	*0.927616311*	*7.659906912*	*4.150414783*	*0.000142729*	*0.003381618*	*0.909502006*		*LCP2*	*1.294442242*	*6.791719055*	*10.86361711*	*7.19E-19*	*6.45E-17*	*32.42151258*
MIR196A1	*-0.574569397*	*6.234327097*	*-4.132671201*	*0.000150977*	*0.003492924*	*0.857726862*		*SLC46A1*	*0.695014403*	*6.279668476*	*10.84532811*	*7.90E-19*	*7.04E-17*	*32.32860138*
GCLM	*0.520449303*	*6.781766453*	*4.12862447*	*0.000152922*	*0.003519644*	*0.845931926*		*SEMA4D*	*0.583535836*	*4.888545511*	*10.84522676*	*7.90E-19*	*7.04E-17*	*32.3280865*
ZFAND5	*-0.610126079*	*9.812239028*	*-4.124915443*	*0.000154727*	*0.003546823*	*0.835125642*		*DHRS9*	*1.507682791*	*5.258528327*	*10.82748381*	*8.66E-19*	*7.62E-17*	*32.23793823*
SUMO1	*0.58478656*	*6.919449969*	*4.115108103*	*0.000159597*	*0.003602113*	*0.806572007*		*LGALS9C*	*1.464987498*	*9.473705594*	*10.82413084*	*8.81E-19*	*7.71E-17*	*32.22090131*
CDO1	*0.663152042*	*9.155089555*	*4.112025378*	*0.000161159*	*0.003619669*	*0.79760285*		*ABCA8*	*-1.082570455*	*5.048134883*	*-10.81638216*	*9.17E-19*	*7.98E-17*	*32.18152763*
CA3	*-0.78577852*	*7.140240869*	*-4.109408986*	*0.000162496*	*0.003632199*	*0.789992761*		*SLC25A4*	*-0.92443782*	*8.116373045*	*-10.81418111*	*9.27E-19*	*8.03E-17*	*32.17034295*
PDPN	*-0.700713301*	*6.501384115*	*-4.098380364*	*0.000168251*	*0.003703926*	*0.757937798*		*TBC1D3G*	*0.63480276*	*5.990561525*	*10.80673794*	*9.63E-19*	*8.32E-17*	*32.13251928*
GRIA3	*0.614751641*	*5.38315039*	*4.084908832*	*0.000175549*	*0.00378948*	*0.718833381*		*PPP2R3B-AS1*	*0.510236912*	*5.178005791*	*10.8052247*	*9.71E-19*	*8.36E-17*	*32.12482928*
GTF2A2	*0.68195308*	*6.510997867*	*4.068117797*	*0.000185078*	*0.0039107*	*0.670172361*		*GAB3*	*0.697717266*	*5.568106067*	*10.80320733*	*9.81E-19*	*8.42E-17*	*32.11457729*
BAMBI	*0.520660718*	*6.854686685*	*4.063337398*	*0.000187881*	*0.003950205*	*0.656334751*		*BIN2*	*1.122628787*	*5.379850784*	*10.80145096*	*9.90E-19*	*8.47E-17*	*32.10565151*
PPP1R1B	*-0.528113221*	*8.227421434*	*-4.04891773*	*0.000196589*	*0.004067927*	*0.614638476*		*MXRA7*	*-0.872040278*	*7.460835687*	*-10.79195072*	*1.04E-18*	*8.85E-17*	*32.05737018*
HSP90AA4P	*0.656675885*	*6.018416523*	*4.046365993*	*0.00019817*	*0.004077097*	*0.607266675*		*TRPM2*	*1.322439507*	*5.904370548*	*10.78965251*	*1.05E-18*	*8.93E-17*	*32.04568998*
HSD17B13	*1.030112089*	*7.437297337*	*4.043424559*	*0.000200008*	*0.004096275*	*0.598771636*		*BMPR1A*	*-0.767907574*	*5.984047405*	*-10.78399199*	*1.08E-18*	*9.14E-17*	*32.01692073*

PPOX	0.534504079	6.409095387	4.037898276	0.000203506	0.00413507	0.58281886		*FBXW4*	-0.580830179	6.955090805	-10.78352852	1.09E-18	9.14E-17	32.01456513
SYT4	-0.746486928	5.840327293	-4.037658207	0.000203659	0.00413507	0.58212607		*WDR81*	0.755520006	7.658395624	10.7817636	1.10E-18	9.20E-17	32.00559481
FOSB	-2.123781719	8.137346602	-4.03462986	0.000205603	0.004167046	0.573388486		*CD53*	1.501828147	7.326492714	10.78086175	1.10E-18	9.22E-17	32.00101108
MAMDC2	-0.752522745	8.242745926	-4.031479573	0.000207644	0.004184059	0.56430219		*RAMP1*	-1.332216213	9.123324552	-10.77424804	1.14E-18	9.51E-17	31.96739539
ABRACL	0.502377102	6.377612504	4.018189224	0.000216471	0.0042842	0.526004317		*TRIM65*	0.632134501	7.17993272	10.7470184	1.31E-18	1.08E-16	31.82898002
RRM2	0.70469678	5.638245784	4.016796666	0.000217417	0.004293096	0.52199478		*BLNK*	1.023928572	5.268403037	10.74045946	1.36E-18	1.12E-16	31.79563579
MSC	0.741575452	7.76188943	4.014916437	0.0002187	0.004306993	0.516582109		*C16orf70*	0.649200945	6.657066877	10.73700079	1.38E-18	1.13E-16	31.77805215
SQSTM1	0.626158359	10.2790696	4.009573033	0.000222387	0.00434012	0.501206155		*DOK7*	-0.681414422	4.689132326	-10.73634551	1.39E-18	1.13E-16	31.77472073
WT1	-0.843727439	6.310422533	-4.008072443	0.000223434	0.004348798	0.496889788		*KCTD9*	-0.559206281	6.653502553	-10.73078039	1.43E-18	1.16E-16	31.74642716
FRG1	0.575502822	6.853272169	3.995778446	0.000232187	0.004458392	0.461554441		*SLC9A3R1*	0.584160728	6.537297891	10.72762323	1.45E-18	1.18E-16	31.73037546
ZFP36	-1.045138276	7.597819418	-3.992008791	0.000234937	0.004481063	0.45072963		*PRSS56*	-0.706030606	4.461869359	-10.7222908	1.49E-18	1.20E-16	31.70326358
KRT18P55	0.770745161	6.221922671	3.987592589	0.000238199	0.00450467	0.438054159		*IL21R*	0.981587777	5.27030525	10.71790344	1.52E-18	1.22E-16	31.68095611
RIDA	0.505472087	7.686952112	3.979268394	0.000244466	0.004558433	0.414179372		*LOC282997*	0.662562892	5.741482407	10.70639205	1.62E-18	1.30E-16	31.62242398
LAIR1	0.569670039	9.528583337	3.977764644	0.000245616	0.004571332	0.409868879		*ZNF862*	0.529727492	6.341767102	10.70457723	1.63E-18	1.30E-16	31.61319575
LOC100128775	0.724209624	6.720113773	3.965488353	0.000255195	0.004649005	0.374707046		*KCND3*	-0.528437433	5.193159067	-10.70426557	1.63E-18	1.30E-16	31.61161101
KLF2	-0.521903516	8.782935022	-3.96453769	0.000255952	0.004653347	0.371986246		*ARRB1*	0.703686083	5.605964708	10.70422392	1.63E-18	1.30E-16	31.61139919
APOB	-0.900879435	6.655857055	-3.961431449	0.000258439	0.004683541	0.363098276		*IL17RA*	0.831126425	6.673716048	10.70078586	1.66E-18	1.32E-16	31.59391673
TRIB1	-0.726117262	8.028277646	-3.958250621	0.000261011	0.004713055	0.354000234		*ARHGAP30*	1.035126429	5.808172771	10.69197901	1.74E-18	1.38E-16	31.54913242
MRPL42	0.569199665	7.835782367	3.957919318	0.00026128	0.004713659	0.353052811		*ITGB1BP2*	-0.792861579	6.141354987	-10.68159601	1.84E-18	1.45E-16	31.49633032
USPL1	0.504040739	7.564267754	3.957476016	0.000261641	0.004714489	0.351785164		*PTPN6*	1.137204421	5.775793174	10.68079395	1.84E-18	1.45E-16	31.49225138
HSD11B1	0.781297576	7.766906399	3.956001478	0.000262845	0.004716337	0.347569115		*SGCD*	-0.765147185	5.854908501	-10.67585864	1.89E-18	1.48E-16	31.46715198
MIR10A	0.705653657	4.153875044	3.952726464	0.000265536	0.0047561	0.338207692		*S1PR4*	1.246851897	7.074454704	10.67350514	1.92E-18	1.50E-16	31.45518261

PHACTR3	*0.500335574*	*6.556736763*	*3.943834327*	*0.000272981*	*0.004837421*	*0.312808283*		*SEZ6L2*	*1.386584996*	*5.665317533*	*10.65481482*	*2.11E-18*	*1.64E-16*	*31.36012264*
TIPARP	*-0.775587185*	*8.109613133*	*-3.939631645*	*0.000276569*	*0.004862213*	*0.300813077*		*APOBR*	*1.612947762*	*6.285235922*	*10.6525205*	*2.13E-18*	*1.65E-16*	*31.34845292*
HLA-J	*0.658840047*	*11.62403894*	*3.933318973*	*0.000282045*	*0.004923834*	*0.28280683*		*ICAM3*	*0.652411225*	*6.990024467*	*10.64951083*	*2.17E-18*	*1.67E-16*	*31.33314456*
PPP1R36	*0.694094708*	*6.713379602*	*3.9282192*	*0.000286545*	*0.004963381*	*0.268270142*		*HLA-F*	*0.664762809*	*9.022476214*	*10.64429718*	*2.23E-18*	*1.71E-16*	*31.30662525*
BTBD8	*0.528091458*	*5.751324228*	*3.925074798*	*0.000289354*	*0.004986115*	*0.259311579*		*RGS14*	*0.56996657*	*5.019376201*	*10.64028613*	*2.27E-18*	*1.74E-16*	*31.28622248*
SLC1A1	*0.506170918*	*6.520450088*	*3.921986543*	*0.000292139*	*0.005012866*	*0.250516266*		*CXCR3*	*0.777477287*	*5.149198733*	*10.63886642*	*2.29E-18*	*1.74E-16*	*31.27900083*
RNF138	*0.576885873*	*7.181558255*	*3.913344008*	*0.000300071*	*0.005087461*	*0.225919798*		*NCSTN*	*0.528697934*	*8.054668214*	*10.63835683*	*2.30E-18*	*1.75E-16*	*31.27640868*
PLRG1	*0.532223399*	*9.10428439*	*3.912030267*	*0.000301295*	*0.005095245*	*0.222183164*		*ITGB7*	*1.577073338*	*6.846003018*	*10.63382411*	*2.35E-18*	*1.78E-16*	*31.25335167*
CYP19A1	*0.504200161*	*5.021677262*	*3.911493406*	*0.000301796*	*0.005099412*	*0.22065636*		*FYB*	*1.363622386*	*6.434238028*	*10.63158349*	*2.38E-18*	*1.79E-16*	*31.24195393*
DGKK	*-0.502670087*	*5.287023886*	*-3.909397705*	*0.000303762*	*0.005119639*	*0.21469724*		*HLA-DRB1*	*1.45842232*	*10.11963909*	*10.6285076*	*2.42E-18*	*1.82E-16*	*31.22630701*
TMEM159	*0.54979693*	*8.115956303*	*3.904127153*	*0.00030876*	*0.005184225*	*0.199717135*		*RNPEP*	*0.598758838*	*8.743374984*	*10.62477608*	*2.46E-18*	*1.84E-16*	*31.20732465*
CARMN	*0.589887437*	*7.12365819*	*3.901182109*	*0.000311587*	*0.005211972*	*0.191350832*		*MILR1*	*1.061143698*	*6.280811852*	*10.61815175*	*2.55E-18*	*1.90E-16*	*31.1736256*
MIR15A	*0.607940372*	*6.37758101*	*3.895346144*	*0.000317263*	*0.005267255*	*0.174780856*		*CLIP1*	*-0.512223241*	*7.638936069*	*-10.6146097*	*2.60E-18*	*1.93E-16*	*31.15560617*
CWC15	*0.501458965*	*5.997751114*	*3.892088578*	*0.000320475*	*0.005294631*	*0.165536838*		*PDS5B*	*-0.574539661*	*6.066554956*	*-10.61243652*	*2.62E-18*	*1.95E-16*	*31.14455042*
ERAP2	*1.345623946*	*7.463600314*	*3.889733362*	*0.000322817*	*0.005324082*	*0.158855724*		*LOC441268*	*0.823917198*	*6.624650133*	*10.60168794*	*2.77E-18*	*2.04E-16*	*31.08986678*
ABHD4	*0.501884278*	*8.404162209*	*3.885612383*	*0.000326954*	*0.005365753*	*0.147170268*		*MAP6*	*-0.681322935*	*5.717977349*	*-10.5966636*	*2.85E-18*	*2.09E-16*	*31.0643044*
FHDC1	*-0.545977936*	*5.217658596*	*-3.885275903*	*0.000327294*	*0.005366927*	*0.146216407*		*LOC606724*	*0.795916051*	*4.84039714*	*10.59268624*	*2.91E-18*	*2.12E-16*	*31.04406828*
LOC440173	*-0.574989435*	*5.206216464*	*-3.881059441*	*0.000331585*	*0.00541148*	*0.134266836*		*SLAMF8*	*2.025149824*	*7.527745509*	*10.58932001*	*2.96E-18*	*2.15E-16*	*31.02694123*
SPAG17	*0.899374704*	*5.530313594*	*3.874014439*	*0.000338875*	*0.00549373*	*0.114315003*		*RTP4*	*0.789112157*	*6.032419631*	*10.5797724*	*3.11E-18*	*2.25E-16*	*30.97836245*
HDDC2	*0.556573271*	*8.673201196*	*3.869565546*	*0.000343558*	*0.005532558*	*0.101724478*		*RRAS2*	*-0.650476657*	*6.722966514*	*-10.57590417*	*3.17E-18*	*2.29E-16*	*30.95868007*
TUBB4A	*0.693812332*	*6.535988278*	*3.869374134*	*0.000343761*	*0.005532558*	*0.101182931*		*TP53I3*	*0.716949301*	*6.887622429*	*10.5756027*	*3.17E-18*	*2.29E-16*	*30.95714608*
TMEM182	*0.801275732*	*6.419899461*	*3.859780697*	*0.000354079*	*0.00563062*	*0.074057583*		*RAC2*	*0.810116067*	*6.175404024*	*10.57443244*	*3.19E-18*	*2.29E-16*	*30.95119148*

LMBRD2	0.501693951	7.413100855	3.855720359	0.000358536	0.005669915	0.06258683		*LRFN4*	0.593929313	7.265910004	10.5691505	3.28E-18	2.35E-16	30.92431513
GOLGA7B	0.507856857	5.741702947	3.855443819	0.000358841	0.005670002	0.061805797		*TMC8*	0.673876614	5.085458387	10.55904605	3.46E-18	2.46E-16	30.87289832
SAA2-SAA4	-0.872850126	6.149212946	-3.855202032	0.000359109	0.005670002	0.06112294		*ARHGAP4*	0.592375854	9.312618159	10.55449918	3.54E-18	2.51E-16	30.84976071
AZGP1	-0.918794639	7.881056193	-3.852169501	0.000362478	0.005691724	0.052560195		*CARD8*	0.595343792	5.889120496	10.55420881	3.54E-18	2.51E-16	30.84828308
GCSHP3	0.552244328	8.27020253	3.849921051	0.000364995	0.005695464	0.046213517		*PPM1J*	-0.545920082	5.035975533	-10.54777611	3.66E-18	2.59E-16	30.81554826
SLC6A6	0.634615751	8.595222299	3.847147486	0.000368124	0.005735332	0.038387084		*NRP1*	0.543613292	6.680141956	10.54193816	3.78E-18	2.66E-16	30.78583924
PRSS50	-0.502487103	6.285001841	-3.845639205	0.000369836	0.005753042	0.034132176		*IGFBP6*	-1.091223609	8.580431926	-10.54001128	3.81E-18	2.68E-16	30.77603327
SLCO4A1	-0.555662767	6.299238001	-3.845226469	0.000370306	0.005755872	0.032967978		*GCOM1*	-0.57117785	6.826993686	-10.53006058	4.02E-18	2.81E-16	30.72539252
JUN	-1.270922244	9.015267591	-3.841785269	0.000374247	0.005790105	0.023263797		*MS4A14*	0.916327346	4.933303507	10.52925132	4.03E-18	2.81E-16	30.721274
PIGB	0.549732688	7.00515006	3.83460965	0.000382594	0.005855794	0.003042201		*SESTD1*	-0.597803002	5.607922556	-10.51980014	4.23E-18	2.94E-16	30.6731733
CCNB1	0.5631752	5.605312286	3.833880147	0.000383452	0.005859961	0.000987426		*ASAP1-IT1*	0.599516629	6.404848031	10.51458086	4.35E-18	3.01E-16	30.6466096
NDUFB6	0.666507887	5.990110755	3.822270327	0.000397369	0.006008335	-0.031687907		*STAT2*	0.562508856	9.006305223	10.51123871	4.43E-18	3.05E-16	30.62959935
PSMA3	0.627011537	8.56270692	3.818947959	0.000401441	0.006042257	-0.041029604		*RAB7B*	0.993225336	6.514220567	10.50028278	4.68E-18	3.22E-16	30.57383626
MYC	-0.913197535	7.975634847	-3.818430042	0.000402079	0.006042257	-0.042485501		*TSPAN15*	0.935169476	5.234817179	10.49459139	4.82E-18	3.30E-16	30.54486753
MAP1LC3B	-0.54621089	6.943365656	-3.817433205	0.000403311	0.00604331	-0.045287398		*APBB2*	-0.644606875	5.789979241	-10.49353845	4.85E-18	3.31E-16	30.53950804
PTGIS	-0.767020821	8.256087962	-3.816712152	0.000404204	0.006044625	-0.047313903		*OAS3*	0.579989683	4.995009245	10.49329363	4.85E-18	3.31E-16	30.53826191
ZNF718	0.773852586	6.100199679	3.809542592	0.000413186	0.006113386	-0.067453508		*TLR7*	1.371182416	5.977004578	10.49265729	4.87E-18	3.31E-16	30.53502294
DSC3	-0.817696674	4.057781634	-3.808826595	0.000414094	0.006122283	-0.069463746		*ZNF587*	0.502883531	6.390703275	10.49074701	4.92E-18	3.34E-16	30.52529952
ZNF252P	0.575494868	6.716232269	3.80132731	0.000423716	0.006177729	-0.090507562		*LOC100507554*	-1.233215307	6.006065813	-10.4895824	4.95E-18	3.35E-16	30.51937161
TUBB1	1.258858939	6.041633329	3.797015324	0.000429346	0.006222962	-0.102598151		*CCL3L3*	2.004805538	6.83827437	10.48289006	5.12E-18	3.45E-16	30.48530663
SOCS3	-1.048520672	7.540441527	-3.784525077	0.000446064	0.006373393	-0.137581617		*SLAMF7*	1.768567575	6.379838854	10.46981115	5.48E-18	3.66E-16	30.41873071
ZBED8	0.818511516	5.209390098	3.779123604	0.000453487	0.006460972	-0.152692577		*VAV1*	1.414756096	6.805299781	10.46572083	5.60E-18	3.73E-16	30.39790899

PTGS2	-1.444417126	5.448674805	-3.778517177	0.000454327	0.00646834	-0.154388421		PERP	-0.872719061	6.964750912	-10.46416671	5.64E-18	3.74E-16	30.38999769
MTCH2	0.556373801	9.949416341	3.777521969	0.00045571	0.006474392	-0.157171176		EGR2	1.576386075	7.034388187	10.4356521	6.54E-18	4.30E-16	30.24483556
GAS5	0.617571919	5.201516328	3.771029247	0.000464833	0.006561823	-0.175316805		DAGLB	0.561214497	5.284425317	10.42396581	6.94E-18	4.55E-16	30.18533908
CLEC4A	0.62158877	6.142827651	3.769557571	0.000466925	0.00657997	-0.179427615		GPR64	-0.604948352	4.311939161	-10.42229003	7.01E-18	4.57E-16	30.17680724
MARCO	-0.569842845	8.327637062	-3.766135093	0.000471825	0.006621492	-0.18898445		SIGLEC1	1.331593946	5.829457224	10.41773689	7.17E-18	4.66E-16	30.15362584
NDUFA13	-0.520904748	8.011832092	-3.765960713	0.000472076	0.006621492	-0.189471268		ANO5	-0.690922645	4.949584369	-10.40832144	7.53E-18	4.88E-16	30.10568802
SLC2A14	-0.781566778	7.370097899	-3.759133464	0.000482005	0.00668619	-0.208521987		LEF1	0.87054112	5.659922136	10.40369435	7.71E-18	4.99E-16	30.08212909
ZFAS1	0.511013733	8.374595693	3.754781454	0.000488439	0.00675074	-0.22065671		LRCH2	-0.760967237	5.113304472	-10.40210446	7.78E-18	5.02E-16	30.07403405
STC1	-0.51778864	6.066989793	-3.740262477	0.000510506	0.006899603	-0.261088505		OAS2	0.684737351	5.357200437	10.39822898	7.93E-18	5.10E-16	30.05430165
UTP15	0.551182694	6.794737726	3.731540978	0.00052422	0.007006524	-0.285337452		GVINP1	0.558670597	4.585013069	10.39725182	7.97E-18	5.12E-16	30.04932635
SLC25A25	-0.60659864	6.957541127	-3.730907257	0.000525231	0.007008569	-0.287098301		CYB561D1	0.503247865	6.449746941	10.38289985	8.59E-18	5.48E-16	29.97624969
PIEZO2	-0.517258833	6.049717934	-3.726417555	0.000532441	0.007068184	-0.299568965		C3orf70	-1.040945184	5.154064919	-10.37257019	9.06E-18	5.76E-16	29.92365179
ADGRD1	-0.735994364	7.087903851	-3.722990339	0.00053801	0.007112615	-0.309083283		DNAJB5	-0.930398868	7.977765542	-10.37180854	9.09E-18	5.78E-16	29.91977347
GPC4	0.894187235	7.304922821	3.720170343	0.000542633	0.007154813	-0.316908532		ADRBK2	1.006929358	6.87009889	10.37138343	9.11E-18	5.78E-16	29.9176088
ADAM22	0.633352166	6.935369302	3.7199101	0.000543062	0.007155745	-0.317630533		RASSF8	-0.617539838	5.136323701	-10.37053055	9.15E-18	5.79E-16	29.91326594
TPT1-AS1	0.530745389	7.341346663	3.714893162	0.000551389	0.007241596	-0.331544091		UAP1L1	0.892150847	5.411828302	10.36965255	9.19E-18	5.80E-16	29.90879513
ABCE1	0.602311179	7.338911934	3.701730578	0.000573823	0.007390938	-0.368002171		DUSP26	-1.561402636	7.880501706	-10.36782441	9.28E-18	5.85E-16	29.89948618
IQCF2	0.616841934	4.337269464	3.698940184	0.00057869	0.007434089	-0.375722504		CORO7	1.015187965	7.089750978	10.36379047	9.48E-18	5.96E-16	29.87894498
SLC9A7	0.71839116	6.162709054	3.698596214	0.000579293	0.007437053	-0.376673978		RELL2	0.543885353	5.910237655	10.36100566	9.61E-18	6.03E-16	29.86476436
SEMA3A	0.509623959	8.16414662	3.686606443	0.000600684	0.00758981	-0.409810809		RBMS3	-0.664954715	5.042278276	-10.36022942	9.65E-18	6.04E-16	29.8608116
AZI2	0.531218957	8.425364451	3.67381772	0.000624337	0.007790184	-0.445094095		ITGA4	0.893259634	5.510376815	10.35025754	1.02E-17	6.32E-16	29.81003252
OR1G1	-0.508829204	4.831343183	-3.666782409	0.000637728	0.007893214	-0.46447679		DAPP1	1.031454635	5.325228396	10.33584912	1.09E-17	6.76E-16	29.7366594

ACOT4	0.567438288	6.341810873	3.651498449	0.000667776	0.008089799	-0.506517849		*SCAMP5*	0.706210837	5.213370075	10.33396886	1.11E-17	6.81E-16	29.72708421
IKZF5	0.582994816	7.454468103	3.650162419	0.000670466	0.008112557	-0.51018843		*MAF*	0.975643138	7.623873357	10.3300878	1.13E-17	6.91E-16	29.70731989
RB1	0.503185207	7.915770167	3.647296657	0.000676272	0.008167972	-0.518059383		*GAA*	0.97484829	9.335282585	10.32583296	1.15E-17	7.03E-16	29.68565182
CD163L1	-0.503267593	8.295659736	-3.639415115	0.000692489	0.008333628	-0.539689575		*FERMT2*	-0.928327361	7.101360186	-10.30955689	1.25E-17	7.63E-16	29.60276309
IFT43	0.505430622	6.30540759	3.636724413	0.00069811	0.008363025	-0.547068302		*TXNDC3*	0.887941766	5.112141061	10.30452641	1.29E-17	7.82E-16	29.57714392
NMRK1	0.642086418	6.14560394	3.636644061	0.000698279	0.008363025	-0.547288608		*MAP4K1*	0.819797611	5.050744476	10.29090684	1.38E-17	8.29E-16	29.50778098
TPRKB	0.503373447	6.226198514	3.634910023	0.000701925	0.008374051	-0.552042281		*CD52*	1.875628613	8.681146007	10.27933025	1.47E-17	8.78E-16	29.44882131
LOC541472	-0.615389197	5.555133687	-3.631454306	0.000709247	0.008418732	-0.561512165		*LOC100506459*	0.533056862	5.109969154	10.27071413	1.53E-17	9.17E-16	29.40493852
RAB31	0.527306507	8.92983619	3.62966895	0.000713059	0.008448458	-0.566402798		*XLOC_013657*	-0.529210868	4.228629454	-10.26935801	1.54E-17	9.20E-16	29.39803163
GPR160	0.639806451	5.880373351	3.627698263	0.000717289	0.008463923	-0.571799626		*LRRC33*	0.894551659	4.992921036	10.26931846	1.54E-17	9.20E-16	29.39783018
MIR148B	0.516919582	4.685726063	3.62451212	0.000724179	0.008520074	-0.580521747		*PRR5L*	0.663992339	4.687738775	10.26191507	1.60E-17	9.50E-16	29.36012337
DHFR	0.607286882	8.765803079	3.621425681	0.000730913	0.008579112	-0.588967037		*FBXL16*	0.66407964	4.621873182	10.26096608	1.61E-17	9.53E-16	29.35528993
LOX	0.730052219	9.206923894	3.615727274	0.000743506	0.008665852	-0.604549266		*XLOC_013983*	-1.334223742	5.006906843	-10.24729323	1.73E-17	1.02E-15	29.28565027
RRN3P1	0.58986477	9.390396282	3.60697009	0.000763264	0.008837893	-0.628470128		*STK38L*	-0.87014676	5.706358729	-10.24600661	1.74E-17	1.02E-15	29.27909709
CRHBP	0.79704129	7.236823253	3.606102297	0.000765249	0.008843947	-0.630838874		*CASP1*	0.978515208	7.178875115	10.24024694	1.79E-17	1.05E-15	29.249761
LACE1	0.54255322	6.719080369	3.605226391	0.000767258	0.008849669	-0.633229457		*FBLN5*	-1.093680153	9.020752135	-10.23973083	1.80E-17	1.05E-15	29.24713223
KLHL4	-0.766543507	4.44553443	-3.593899749	0.000793692	0.009091755	-0.664114809		*NCF4*	1.121887345	6.556601295	10.23897184	1.81E-17	1.05E-15	29.2432664
MOSPD2	0.558366957	7.331656242	3.593546367	0.000794531	0.009093279	-0.665077565		*UBA2*	-0.599246507	7.234179885	-10.22831102	1.91E-17	1.10E-15	29.18896628
HBEGF	-0.589035615	7.193740723	-3.591253033	0.000799994	0.00913254	-0.671324307		*DOK3*	0.884855961	6.435138301	10.22807618	1.91E-17	1.10E-15	29.18777009
SLC2A3	-1.200992306	8.662065878	-3.583539054	0.000818635	0.009262616	-0.69232039		*HIPK3*	-0.514198639	6.230255748	-10.2280133	1.91E-17	1.10E-15	29.18744984
SULF1	-1.030490889	7.453506109	-3.579078856	0.000829602	0.009334379	-0.704449128		*MFAP3L*	-0.575039592	4.970453673	-10.20686034	2.13E-17	1.22E-15	29.07970643
EGR3	-0.533800608	6.210803231	-3.578743956	0.000830432	0.009334379	-0.7053595		*CSDC2*	-0.976365056	5.730939852	-10.19922604	2.22E-17	1.27E-15	29.0408202

RBP7	0.576881437	8.503861692	3.577595013	0.000833282	0.009341827	-0.70848237		TYROBP	1.562928665	10.22280962	10.19643478	2.25E-17	1.28E-15	29.02660252
CXCL2	-1.104119454	5.697311849	-3.57413112	0.000841933	0.009403767	-0.717894069		MCM5	0.500197768	6.21055786	10.1942762	2.28E-17	1.29E-15	29.01560743
CTGF	-0.682472195	8.746680636	-3.564778339	0.000865724	0.009566476	-0.743281666		MYO15B	0.570420315	5.692095071	10.19348126	2.28E-17	1.29E-15	29.01155825
SLC27A2	-0.510036532	4.724087575	-3.562766514	0.000870925	0.009607567	-0.748737924		AP1G2	0.623606939	5.650409026	10.19115249	2.31E-17	1.30E-15	28.99969628
SLAMF7	0.696590128	6.088925039	3.556382602	0.000887627	0.009717942	-0.766040574		CLTC-IT1	0.629896453	5.204093871	10.18782737	2.35E-17	1.32E-15	28.98275913
DPP4	-0.793128452	7.192900267	-3.545653333	0.000916391	0.009912193	-0.795082467		PPP1R12A	-0.674715717	5.656673118	-10.1873374	2.36E-17	1.32E-15	28.98026338
TLR1	0.597255425	6.569008224	3.541476716	0.000927827	0.00996579	-0.806374699		VAMP8	1.422076243	9.630848923	10.1804117	2.44E-17	1.37E-15	28.94498582
ABCG2	0.565633001	5.552860751	3.530966453	0.000957214	0.010157798	-0.834758709		EVI2B	1.273944482	6.554932713	10.17451775	2.52E-17	1.40E-15	28.91496353
KLF4	-1.186425522	8.465398246	-3.517402505	0.000996456	0.010357852	-0.87132071		PLCB4	-0.870516301	4.72681501	-10.17083749	2.57E-17	1.43E-15	28.89621716
SNORA61	0.602789665	7.230282892	3.516430696	0.000999325	0.010360949	-0.873937261		LIMS3L	-0.912459609	6.113020803	-10.17054362	2.57E-17	1.43E-15	28.89472024
PARM1	0.535958325	7.680096076	3.516149572	0.001000157	0.010360949	-0.8746941		COL1A1	1.469567997	10.00587517	10.16895507	2.59E-17	1.44E-15	28.88662853
CYR61	-1.271047507	7.627466865	-3.505980533	0.001030689	0.010565355	-0.902048483		MYD88	0.807644943	6.750342307	10.16814319	2.60E-17	1.44E-15	28.882493
OSR2	-0.629168834	7.265212185	-3.495023633	0.001064583	0.010829489	-0.931472837		PLN	-1.442463879	6.306757041	-10.16234353	2.68E-17	1.48E-15	28.85295076
PIGF	0.548883025	6.516537559	3.491720964	0.001075006	0.010896704	-0.940331954		MPP7	-0.770147049	4.871437925	-10.15913414	2.73E-17	1.50E-15	28.83660272
SNORD59B	0.514969422	6.510332653	3.484368683	0.001098562	0.011021234	-0.96003697		WASF3	-0.729078476	5.50586748	-10.1555527	2.78E-17	1.52E-15	28.81835951
ZNRD1ASP	0.532289732	5.051017602	3.479505213	0.001114414	0.011115776	-0.973058875		CLEC11A	0.787148109	8.019470936	10.15101375	2.85E-17	1.56E-15	28.7952389
SCARNA6	-0.69832156	7.557604385	-3.472754518	0.001136779	0.011243481	-0.991116874		OASL	1.012876877	5.376393508	10.13975184	3.02E-17	1.64E-15	28.7378724
C3orf14	0.546551768	5.030062249	3.468701295	0.001150413	0.011333464	-1.001949697		CXorf69	-0.80788522	5.939689556	-10.1388406	3.03E-17	1.64E-15	28.73323069
HBB	1.807112254	9.819741273	3.466906909	0.001156498	0.011357415	-1.006743175		ABTB2	-0.640795046	5.986498145	-10.13006864	3.17E-17	1.71E-15	28.68854738
HIST1H3H	0.567432404	5.523091941	3.466102319	0.001159237	0.0113613	-1.008892083		CEBPA	1.626700209	6.704611411	10.12304968	3.29E-17	1.77E-15	28.65279358
GPRC5A	-0.597889921	6.050679927	-3.460047672	0.001180045	0.01147272	-1.025053865		MYO5A	0.857675581	7.077990688	10.12053837	3.33E-17	1.79E-15	28.64000121
ZNF675	0.509120013	5.350403333	3.459319523	0.001182571	0.011486108	-1.026996453		GPR65	1.251593096	5.900640633	10.11936969	3.35E-17	1.79E-15	28.63404807

CXCL9	*0.644013611*	*5.450443908*	*3.456982981*	*0.001190712*	*0.011525981*	*-1.033228415*		*ARHGEF9*	*-0.728214481*	*6.115463642*	*-10.11032951*	*3.51E-17*	*1.87E-15*	*28.58799828*
ZNF845	*0.631364997*	*7.3505281*	*3.448180519*	*0.001221867*	*0.011669018*	*-1.056684679*		*COX6A2*	*-0.54999343*	*4.424983155*	*-10.10857911*	*3.54E-17*	*1.88E-15*	*28.57908189*
EIF1AY	*2.053999572*	*6.593324427*	*3.447483862*	*0.001224365*	*0.011669115*	*-1.058539641*		*ZNF671*	*0.644860535*	*5.173051751*	*10.0994906*	*3.71E-17*	*1.97E-15*	*28.53278583*
SLPI	*-0.971647162*	*6.5798397*	*-3.445073981*	*0.001233046*	*0.011725667*	*-1.064954688*		*C11orf75*	*0.728061168*	*7.810825756*	*10.09509199*	*3.80E-17*	*2.01E-15*	*28.51037969*
PLK3	*-0.563571155*	*7.184391233*	*-3.440146214*	*0.001250979*	*0.011798804*	*-1.078064354*		*PRCP*	*0.579252635*	*10.02709453*	*10.09405528*	*3.82E-17*	*2.02E-15*	*28.50509876*
NFKBIZ	*-1.202204102*	*7.294612*	*-3.43608268*	*0.001265955*	*0.011880359*	*-1.088866804*		*HCLS1*	*1.093900855*	*9.077471183*	*10.08853273*	*3.93E-17*	*2.07E-15*	*28.47696736*
LBP	*0.855779481*	*7.931255648*	*3.432897768*	*0.001277811*	*0.011930896*	*-1.097328448*		*ID2*	*-0.838181382*	*8.639521356*	*-10.08450507*	*4.01E-17*	*2.11E-15*	*28.45645084*
SIM1	*0.99377249*	*6.236565911*	*3.426045073*	*0.001303682*	*0.012042186*	*-1.11551942*		*LOC100131096*	*0.566942569*	*4.637537314*	*10.08367816*	*4.03E-17*	*2.12E-15*	*28.45223859*
LFNG	*0.531285265*	*7.36921661*	*3.418634978*	*0.001332218*	*0.012172824*	*-1.135166634*		*PLEKHO2*	*0.863576027*	*8.157241561*	*10.08252152*	*4.05E-17*	*2.13E-15*	*28.44634677*
EVI2A	*0.522956196*	*5.734884318*	*3.414505268*	*0.001348379*	*0.012256069*	*-1.146105607*		*C13orf33*	*-1.258252633*	*9.090629128*	*-10.08104654*	*4.08E-17*	*2.14E-15*	*28.43883335*
KRT19	*-0.70276931*	*7.689752312*	*-3.409639856*	*0.001367658*	*0.01237019*	*-1.158983592*		*XLOC_l2_015764*	*-0.51815395*	*4.066807567*	*-10.07973861*	*4.11E-17*	*2.15E-15*	*28.43217089*
INSIG1	*0.590761184*	*8.391332453*	*3.407709291*	*0.001375381*	*0.012411957*	*-1.164090567*		*SCO2*	*0.751364886*	*7.211327674*	*10.0781238*	*4.15E-17*	*2.16E-15*	*28.42394522*
TM4SF18	*0.597504929*	*8.715210509*	*3.403580707*	*0.001392035*	*0.012462299*	*-1.175006428*		*TIMELESS*	*0.660768654*	*6.385444529*	*10.07536677*	*4.21E-17*	*2.18E-15*	*28.40990117*
COPS2	*0.547227337*	*9.50509968*	*3.402431367*	*0.001396706*	*0.012474865*	*-1.178043892*		*ATP11A*	*0.562871597*	*6.260315135*	*10.07477954*	*4.22E-17*	*2.18E-15*	*28.40690984*
PFKFB3	*-0.594822641*	*10.22118071*	*-3.398858327*	*0.00141132*	*0.012571694*	*-1.18748291*		*CNNM4*	*0.574761219*	*6.843534041*	*10.07330365*	*4.25E-17*	*2.19E-15*	*28.39939184*
KLF3-AS1	*0.60909645*	*6.794221726*	*3.397462562*	*0.001417069*	*0.012600634*	*-1.191168589*		*FABP5*	*1.472384378*	*7.627146444*	*10.07050545*	*4.31E-17*	*2.22E-15*	*28.3851381*
IL6	*-1.298094535*	*5.91249107*	*-3.38602246*	*0.001465033*	*0.012838468*	*-1.221344498*		*C11orf52*	*-0.509741242*	*4.798371055*	*-10.06866181*	*4.35E-17*	*2.24E-15*	*28.37574675*
C1orf132	*0.573409883*	*6.519807095*	*3.383736129*	*0.001474802*	*0.012884537*	*-1.227368148*		*WNT11*	*-1.386999226*	*5.021512373*	*-10.06751189*	*4.38E-17*	*2.25E-15*	*28.36988918*
ACTA2	*-0.823551525*	*9.339277069*	*-3.378577063*	*0.001497074*	*0.013027872*	*-1.240951721*		*RANGAP1*	*0.543355854*	*8.545692744*	*10.06349554*	*4.47E-17*	*2.29E-15*	*28.34943032*
ZNF814	*0.684981723*	*5.970842373*	*3.376733565*	*0.00150511*	*0.013047843*	*-1.245802641*		*LINC00086*	*-0.548026874*	*5.407147295*	*-10.05720289*	*4.62E-17*	*2.35E-15*	*28.3173763*
CCND2	*0.67177763*	*10.37634215*	*3.373777729*	*0.001518081*	*0.013096641*	*-1.253577311*		*AOX1*	*-1.513873644*	*5.942645763*	*-10.05516216*	*4.67E-17*	*2.37E-15*	*28.30698108*
MCTP2	*-0.507679273*	*6.059306904*	*-3.369435746*	*0.001537327*	*0.01317161*	*-1.264990736*		*CDC42EP4*	*-0.670960198*	*6.123332641*	*-10.05499022*	*4.67E-17*	*2.37E-15*	*28.30610524*

PCDHB4	0.603692511	6.279530759	3.367594676	0.001545557	0.013200381	-1.269827621		LYL1	0.548419263	5.24225551	10.05070678	4.78E-17	2.42E-15	28.28428598
CXCL10	1.090022721	5.560432179	3.355069147	0.001602674	0.013510108	-1.302693818		RAB37	0.750488475	5.167516127	10.03600907	5.16E-17	2.60E-15	28.20941817
ALCAM	0.85259143	7.771567182	3.346311788	0.001643794	0.013753786	-1.325629905		HHEX	0.638577679	5.155353634	10.03376782	5.22E-17	2.63E-15	28.19800165
MCL1	-0.738373014	10.11316025	-3.344609983	0.0016519	0.013795639	-1.330082955		APBB1IP	1.256728427	6.930785212	10.02996247	5.32E-17	2.66E-15	28.17861789
CD55	-0.577131637	8.755673286	-3.343723305	0.001656139	0.013806717	-1.332402561		C4orf48	1.027113467	7.862262739	10.02988747	5.32E-17	2.66E-15	28.17823589
RASGRP3	0.70535605	7.388902138	3.339800601	0.001675015	0.013881371	-1.342660267		PTPRC	1.198545056	7.633588523	10.01923087	5.62E-17	2.79E-15	28.12395347
RGS18	1.043130182	5.269405935	3.337800237	0.001684719	0.01392319	-1.347888401		MAP3K14	0.613526475	6.502076321	10.01756447	5.67E-17	2.81E-15	28.1154652
MSTO2P	0.538960276	8.086793212	3.337703332	0.00168519	0.01392319	-1.348141623		FASLG	0.735071138	4.599524883	10.01239379	5.82E-17	2.88E-15	28.0891271
GLIPR1	0.60491272	8.234687681	3.318536685	0.001780944	0.01447714	-1.398140628		CARD9	0.854136111	5.317850268	10.01119837	5.86E-17	2.89E-15	28.08303794
TAS2R43	0.763461778	4.562518112	3.317910812	0.001784156	0.014485579	-1.39977043		VIPR2	-0.783837276	4.971242253	-10.00878621	5.93E-17	2.92E-15	28.07075107
OR7E13P	0.651548064	5.870338165	3.315664438	0.001795731	0.014550004	-1.405618593		CHODL	-1.107537646	5.213837685	-10.00693922	5.99E-17	2.95E-15	28.06134302
TNMD	0.842903143	8.267368779	3.312938827	0.00180987	0.014614206	-1.412711229		IGLL1	1.390457665	6.468974744	10.0060496	6.02E-17	2.96E-15	28.05681157
LOC102725104	0.50841978	5.852751733	3.309866339	0.001825936	0.014711261	-1.420702355		CCR2	1.11093446	5.642369835	10.00578395	6.03E-17	2.96E-15	28.05545842
COL14A1	-0.627973355	8.282826273	-3.295946386	0.001900433	0.015104475	-1.456850857		IRF8	1.43640058	6.403495844	9.999052812	6.24E-17	3.05E-15	28.0211721
JUNB	-0.944163274	8.65457201	-3.291352517	0.001925645	0.015238208	-1.468760611		PITPNB	-0.530983308	8.198786479	-9.996826833	6.31E-17	3.08E-15	28.0098337
FAM231B	-0.603740501	6.190983193	-3.289899222	0.001933686	0.015258708	-1.472526254		SLC22A18	1.00394094	7.074615251	9.992303259	6.46E-17	3.15E-15	27.9867922
GALNT13	0.551287957	6.479298663	3.287968653	0.001944418	0.01530196	-1.477527023		SASH3	1.247112602	6.125390712	9.990292946	6.53E-17	3.18E-15	27.97655241
PTPRC	0.527843399	7.699559227	3.285897597	0.001955994	0.015338707	-1.482889738		MRAP2	-1.336162876	7.069142165	-9.98857154	6.59E-17	3.20E-15	27.96778422
USP9Y	1.51515791	5.844745176	3.283495611	0.001969501	0.015420431	-1.489106811		RAI2	-0.924413508	6.608577034	-9.986606829	6.65E-17	3.23E-15	27.95777674
AREG	-0.83802522	5.275118114	-3.278014087	0.002000657	0.015597169	-1.503284434		ELL2	-0.951763183	8.65399351	-9.986176022	6.67E-17	3.23E-15	27.95558238
SNORD45C	0.557427481	4.93631713	3.273439991	0.002027013	0.015729022	-1.515104135		IMPDH1	0.649768395	9.23350546	9.985058561	6.71E-17	3.24E-15	27.94989048
SNORD45A	1.001526022	7.918158842	3.271596127	0.00203773	0.015775439	-1.519865964		TCIRG1	0.924825447	8.562578039	9.980364907	6.87E-17	3.30E-15	27.92598297

SNORD52	*0.503409297*	*4.945011469*	*3.270807683*	*0.002042329*	*0.015792693*	*-1.521901646*		*BTK*	*1.175261553*	*5.902412159*	*9.980198687*	*6.88E-17*	*3.30E-15*	*27.92513632*
SLC28A3	*-0.68314739*	*5.633107093*	*-3.253261174*	*0.002147258*	*0.016287149*	*-1.567128202*		*DOCK8*	*0.80238442*	*5.441592553*	*9.977164732*	*6.99E-17*	*3.35E-15*	*27.90968269*
CYCS	*0.509227917*	*8.310594188*	*3.245584084*	*0.002194755*	*0.01651509*	*-1.586869693*		*MAP3K1*	*0.612559162*	*7.271137306*	*9.976554269*	*7.01E-17*	*3.36E-15*	*27.90657327*
NRCAM	*0.800972576*	*6.977177219*	*3.243080483*	*0.002210458*	*0.016592131*	*-1.593301522*		*HLA-C*	*0.674907913*	*11.14961226*	*9.972404092*	*7.16E-17*	*3.42E-15*	*27.88543421*
NPR3	*0.772140539*	*9.327096925*	*3.232787409*	*0.002276143*	*0.016945186*	*-1.619712966*		*SBDS*	*-0.638441821*	*7.543402747*	*-9.965828664*	*7.41E-17*	*3.54E-15*	*27.85194228*
WEE1	*-0.594608588*	*5.94372068*	*-3.231284204*	*0.002285889*	*0.016992426*	*-1.623565818*		*BAG2*	*-0.927670805*	*7.138941577*	*-9.965235452*	*7.43E-17*	*3.54E-15*	*27.84892077*
PPBP	*1.858629793*	*7.16520697*	*3.229554439*	*0.002297152*	*0.017050791*	*-1.627998007*		*AKAP2*	*-0.557638955*	*5.354549701*	*-9.9616779*	*7.57E-17*	*3.60E-15*	*27.83080052*
FCGR1CP	*0.514498141*	*6.234154703*	*3.22759679*	*0.002309963*	*0.017101427*	*-1.633012353*		*LOC388242*	*0.635921433*	*5.947796872*	*9.959256551*	*7.66E-17*	*3.64E-15*	*27.81846753*
HSP90B3P	*0.50383995*	*5.694617746*	*3.225050935*	*0.002326724*	*0.017174518*	*-1.639530556*		*EFHD2*	*0.750213917*	*7.703317209*	*9.951010029*	*8.00E-17*	*3.78E-15*	*27.7764647*
TSPAN13	*0.521251989*	*8.204776745*	*3.208481949*	*0.002438651*	*0.017629725*	*-1.681875341*		*NOX4*	*-0.897912259*	*5.867488154*	*-9.950442284*	*8.02E-17*	*3.78E-15*	*27.77357296*
IL1RL1	*-0.615889875*	*5.333747789*	*-3.203662941*	*0.002472147*	*0.017794654*	*-1.694165929*		*GADD45A*	*-0.912834032*	*8.71924878*	*-9.947504649*	*8.14E-17*	*3.83E-15*	*27.75861055*
SLC7A10	*-0.539215082*	*8.69342224*	*-3.201903557*	*0.002484485*	*0.017844904*	*-1.698650293*		*APOOL*	*-0.507520343*	*6.055926504*	*-9.945025325*	*8.25E-17*	*3.88E-15*	*27.74598253*
ZFY	*1.035040889*	*5.951663975*	*3.199607608*	*0.002500672*	*0.017899014*	*-1.704499982*		*C20orf72*	*0.65582448*	*7.299577837*	*9.939519026*	*8.49E-17*	*3.98E-15*	*27.71793729*
NDUFA1	*-0.544471279*	*9.118871709*	*-3.193539806*	*0.002543934*	*0.018115674*	*-1.719947236*		*GNG12*	*-0.704062796*	*7.958424736*	*-9.937977406*	*8.56E-17*	*4.01E-15*	*27.7100854*
LRRN4	*-0.615053813*	*6.410255394*	*-3.193367134*	*0.002545175*	*0.018118059*	*-1.720386556*		*SMARCA1*	*-0.628620729*	*6.997134393*	*-9.935848831*	*8.65E-17*	*4.05E-15*	*27.69924402*
CYP1A1	*-0.549042218*	*5.189324417*	*-3.190196192*	*0.002568074*	*0.018184116*	*-1.728451575*		*C1QC*	*1.361086348*	*7.224827578*	*9.935198292*	*8.68E-17*	*4.05E-15*	*27.69593067*
PDGFRL	*-0.511051047*	*8.527808089*	*-3.186024542*	*0.002598496*	*0.018309664*	*-1.739054248*		*SOLH*	*0.559561744*	*7.340963992*	*9.927723782*	*9.02E-17*	*4.20E-15*	*27.65786142*
BNC1	*-0.649861716*	*5.48212336*	*-3.182925504*	*0.002621315*	*0.018410919*	*-1.746925199*		*ADAMTS14*	*1.127536165*	*5.262191701*	*9.919742387*	*9.40E-17*	*4.36E-15*	*27.61721104*
CD69	*-1.174633613*	*6.332630255*	*-3.182072265*	*0.002627631*	*0.018433911*	*-1.749091426*		*KCNMA1*	*-0.77055019*	*6.025337092*	*-9.919477945*	*9.41E-17*	*4.36E-15*	*27.61586421*
MIRLET7C	*0.538144238*	*5.049834674*	*3.180581867*	*0.002638698*	*0.018481058*	*-1.752874423*		*LIMK1*	*0.952744182*	*6.234350784*	*9.911053226*	*9.83E-17*	*4.54E-15*	*27.57295659*
UTY	*1.646814319*	*6.265326058*	*3.167570641*	*0.002737198*	*0.018896117*	*-1.78585329*		*ARRB2*	*1.064919615*	*7.978290926*	*9.903726118*	*1.02E-16*	*4.71E-15*	*27.53563972*
ANGPT1	*0.510519501*	*8.261614425*	*3.163141806*	*0.00277151*	*0.019024629*	*-1.797059602*		*XLOC_l2_003156*	*0.644711853*	*7.462268399*	*9.900122949*	*1.04E-16*	*4.79E-15*	*27.51728903*

OR52K3P	*-0.607078412*	*5.404696259*	*-3.157566317*	*0.002815283*	*0.019199828*	*-1.811153371*		*FBXO41*	*1.123556799*	*6.074619674*	*9.899918346*	*1.04E-16*	*4.79E-15*	*27.516247*
COL8A1	*-0.584381501*	*6.392642528*	*-3.156108485*	*0.002826836*	*0.019226137*	*-1.814835926*		*LSP1*	*0.959561461*	*7.925362715*	*9.898519161*	*1.05E-16*	*4.81E-15*	*27.50912109*
CSTA	*0.640689859*	*7.221098483*	*3.152355752*	*0.00285678*	*0.019361883*	*-1.824310614*		*SNORA11*	*-1.071820172*	*6.376919723*	*-9.897599368*	*1.05E-16*	*4.81E-15*	*27.50443668*
BTN3A2	*0.582523482*	*7.814060863*	*3.143264039*	*0.002930569*	*0.019629551*	*-1.847235545*		*STAT1*	*0.655390804*	*6.24860478*	*9.896949411*	*1.06E-16*	*4.82E-15*	*27.50112653*
STAG3L4	*0.613362227*	*6.495398694*	*3.143172343*	*0.002931323*	*0.019629551*	*-1.847466549*		*HDGFRP3*	*-0.562503729*	*6.844522214*	*-9.896139064*	*1.06E-16*	*4.84E-15*	*27.49699953*
OR14C36	*-0.511181215*	*6.187189389*	*-3.138879145*	*0.002966797*	*0.01982888*	*-1.858277271*		*OSTF1*	*0.632327027*	*7.818848117*	*9.892189666*	*1.08E-16*	*4.92E-15*	*27.47688584*
SULT1A2	*0.550593411*	*7.186052004*	*3.134545579*	*0.003003014*	*0.019955866*	*-1.869180214*		*BID*	*0.979353679*	*7.154042525*	*9.88045862*	*1.15E-16*	*5.20E-15*	*27.41714238*
NSA2	*0.547194819*	*8.960420581*	*3.1337631*	*0.003009598*	*0.01997969*	*-1.871147862*		*MS4A7*	*1.362088007*	*8.019215397*	*9.880265921*	*1.15E-16*	*5.20E-15*	*27.41616102*
LYRM9	*0.530738188*	*5.776680881*	*3.132215958*	*0.003022655*	*0.020019833*	*-1.87503745*		*CPNE7*	*1.07136872*	*5.75339156*	*9.879391163*	*1.16E-16*	*5.22E-15*	*27.41170615*
ANK2	*-0.605478738*	*7.426752917*	*-3.129704654*	*0.003043964*	*0.020114312*	*-1.881348405*		*XLOC_l2_013193*	*-1.039526505*	*5.797040035*	*-9.87701116*	*1.17E-16*	*5.28E-15*	*27.39958557*
PAMR1	*-0.712104673*	*7.113398769*	*-3.126171005*	*0.003074187*	*0.020293898*	*-1.890223125*		*SVIL*	*-0.655279141*	*6.163734874*	*-9.876210078*	*1.18E-16*	*5.29E-15*	*27.39550593*
IGBP1P1	*0.589919963*	*5.065871847*	*3.122412516*	*0.003106643*	*0.020407061*	*-1.899655573*		*NFKBIE*	*0.954684234*	*7.47332993*	*9.875325119*	*1.18E-16*	*5.31E-15*	*27.39099915*
NR4A2	*-1.045564701*	*6.411719364*	*-3.118411358*	*0.003141549*	*0.020521707*	*-1.909689144*		*EIF5A2*	*-0.526525016*	*5.49696516*	*-9.874240358*	*1.19E-16*	*5.33E-15*	*27.38547486*
SUCNR1	*0.593216967*	*5.471866297*	*3.118031035*	*0.003144886*	*0.020536795*	*-1.910642444*		*ATP6V0D1*	*0.570393124*	*6.271612966*	*9.872405584*	*1.20E-16*	*5.37E-15*	*27.37613106*
ERRFI1	*-0.649599926*	*8.349925094*	*-3.115271359*	*0.003169201*	*0.020629553*	*-1.917557508*		*ITGAM*	*1.451367238*	*6.581282138*	*9.870332258*	*1.21E-16*	*5.42E-15*	*27.36557245*
FCGR3B	*0.688152471*	*7.428382308*	*3.083284958*	*0.003464236*	*0.021844355*	*-1.997423083*		*HLA-DMA*	*1.365124087*	*8.046518971*	*9.868514918*	*1.22E-16*	*5.46E-15*	*27.35631753*
GJA1	*-0.542961785*	*7.746827771*	*-3.081473787*	*0.003481691*	*0.021899156*	*-2.001929576*		*SULT1A4*	*0.609410822*	*7.444314592*	*9.864785801*	*1.25E-16*	*5.56E-15*	*27.33732686*
ZNF98	*-0.641790275*	*4.568054331*	*-3.053114738*	*0.003766012*	*0.023184113*	*-2.072269566*		*BEX2*	*-0.876163406*	*5.762830228*	*-9.860497901*	*1.28E-16*	*5.66E-15*	*27.31549078*
MSL3P1	*0.67063736*	*6.62003714*	*3.052434076*	*0.003773097*	*0.023204335*	*-2.073952686*		*NCKAP1*	*-0.785911168*	*7.175011621*	*-9.858557039*	*1.29E-16*	*5.70E-15*	*27.30560705*
FGF9	*-0.616860097*	*4.694368145*	*-3.050688436*	*0.003791324*	*0.023268275*	*-2.078268142*		*IL2RG*	*1.056348028*	*5.465890275*	*9.845876867*	*1.38E-16*	*6.08E-15*	*27.24103511*
LOC100996634	*-0.528495199*	*7.648309125*	*-3.046614352*	*0.003834185*	*0.023472294*	*-2.088333609*		*POLD1*	*0.562936662*	*6.347284655*	*9.845261967*	*1.38E-16*	*6.09E-15*	*27.23790387*
TNFRSF11B	*0.58395471*	*6.296449374*	*3.042538352*	*0.003877521*	*0.023666497*	*-2.098395095*		*PLA2G15*	*1.189090041*	*8.894379936*	*9.843426982*	*1.39E-16*	*6.14E-15*	*27.22855967*

MBNL3	*0.519630787*	*8.29747788*	*3.041900563*	*0.003884344*	*0.023699623*	*-2.099968668*		*CDKN2A*	*0.951992483*	*6.170318981*	*9.842147813*	*1.40E-16*	*6.16E-15*	*27.22204584*
RGS1	*-0.906707871*	*5.521897661*	*-3.039030896*	*0.00391518*	*0.023838117*	*-2.107046157*		*TMEM106A*	*0.561880294*	*4.929473477*	*9.823610768*	*1.54E-16*	*6.77E-15*	*27.12765348*
CD226	*0.535095149*	*5.620754735*	*3.032558707*	*0.003985573*	*0.024176184*	*-2.122992665*		*PRKAA2*	*-0.690103763*	*4.87104294*	*-9.822583148*	*1.55E-16*	*6.78E-15*	*27.12242088*
SCFV	*-0.697804676*	*8.67209068*	*-3.028024543*	*0.004035593*	*0.024348765*	*-2.134151004*		*STAC*	*-0.695029378*	*4.685059837*	*-9.822116127*	*1.56E-16*	*6.78E-15*	*27.12004284*
ADAMTS1	*-0.919582545*	*8.612245223*	*-3.021178063*	*0.004112237*	*0.024626869*	*-2.150979224*		*KCNJ2*	*0.949900368*	*5.7819776*	*9.816296297*	*1.60E-16*	*6.95E-15*	*27.09040886*
GFPT2	*-0.642030953*	*7.627865288*	*-3.017522122*	*0.004153721*	*0.024732171*	*-2.159955124*		*EFHA2*	*-0.810555678*	*5.698511621*	*-9.808265843*	*1.67E-16*	*7.24E-15*	*27.04951945*
INPP4B	*-0.601471123*	*7.146558365*	*-3.003358618*	*0.004318165*	*0.02529376*	*-2.194661607*		*NTF3*	*-0.981487086*	*6.558997287*	*-9.803093968*	*1.72E-16*	*7.41E-15*	*27.02318585*
MT1A	*-0.50336924*	*7.834602585*	*-2.975672386*	*0.004657361*	*0.026757298*	*-2.262194406*		*UNC93B1*	*0.961561854*	*6.083152314*	*9.7960417*	*1.78E-16*	*7.67E-15*	*26.98727855*
DEFB132	*-0.571807775*	*7.675541147*	*-2.973527329*	*0.004684656*	*0.026863637*	*-2.267409449*		*RCBTB2*	*0.523277754*	*6.932877193*	*9.792456187*	*1.81E-16*	*7.80E-15*	*26.96902287*
SNORD104	*-0.680133069*	*7.190686847*	*-2.971664367*	*0.004708482*	*0.02693699*	*-2.271936645*		*CCR1*	*1.562048485*	*7.169348664*	*9.784586098*	*1.89E-16*	*8.11E-15*	*26.92895296*
LIPA	*0.717560021*	*10.23191917*	*2.971029305*	*0.00471663*	*0.026966731*	*-2.273479484*		*LOC648149*	*-0.789234344*	*5.106625638*	*-9.781184135*	*1.92E-16*	*8.24E-15*	*26.91163247*
FLJ36840	*0.551473339*	*6.675166189*	*2.96759087*	*0.004760974*	*0.027136333*	*-2.281829133*		*ARHGAP5*	*-0.6361365*	*6.372349407*	*-9.776929386*	*1.97E-16*	*8.41E-15*	*26.88997044*
SC5D	*0.625367384*	*7.735634922*	*2.957878781*	*0.004888343*	*0.027649431*	*-2.305378641*		*SHROOM3*	*-0.747100217*	*5.176329806*	*-9.769250505*	*2.05E-16*	*8.71E-15*	*26.85087606*
CCL13	*-0.620800456*	*6.506631636*	*-2.95664436*	*0.004904758*	*0.027707432*	*-2.308368147*		*CD84*	*1.529156556*	*6.04449772*	*9.759883904*	*2.15E-16*	*9.11E-15*	*26.80319064*
FGFR2	*0.531483359*	*7.290872663*	*2.954050476*	*0.004939418*	*0.027865264*	*-2.31464728*		*NAGA*	*0.656564195*	*6.355560577*	*9.748329076*	*2.28E-16*	*9.66E-15*	*26.74436714*
PERP	*0.587524233*	*8.540007785*	*2.950809494*	*0.004983047*	*0.028010722*	*-2.322487719*		*PGR*	*-0.91520145*	*5.502355101*	*-9.746834916*	*2.30E-16*	*9.72E-15*	*26.73676082*
CLIC6	*0.577714973*	*6.636306394*	*2.947392216*	*0.005029438*	*0.028200125*	*-2.330748439*		*MAN2B1*	*0.949666908*	*6.683426836*	*9.745625537*	*2.31E-16*	*9.77E-15*	*26.73060427*
PEMT	*0.615080423*	*9.568716327*	*2.941627343*	*0.005108615*	*0.028460453*	*-2.344669628*		*LOC100507165*	*-0.82438024*	*6.713137555*	*-9.739076204*	*2.39E-16*	*1.00E-14*	*26.69726426*
PKHD1L1	*-1.012185375*	*4.497093286*	*-2.940971517*	*0.005117696*	*0.028463581*	*-2.346252186*		*TNFRSF25*	*0.779007802*	*5.885829505*	*9.732987743*	*2.47E-16*	*1.03E-14*	*26.66627111*
NPY1R	*0.56519889*	*9.782163082*	*2.930146665*	*0.005269772*	*0.028994623*	*-2.372339262*		*PTPRJ*	*0.640553911*	*5.0892873*	*9.730804089*	*2.50E-16*	*1.04E-14*	*26.65515546*
IER2	*-0.53851529*	*8.200901207*	*-2.9284456*	*0.005294049*	*0.029072185*	*-2.376432847*		*STAC3*	*1.323513128*	*6.761772075*	*9.723382782*	*2.59E-16*	*1.08E-14*	*26.6173788*
HBA2	*1.092378995*	*11.45919961*	*2.923583634*	*0.005364016*	*0.029331879*	*-2.388124302*		*PPARGC1B*	*0.73828937*	*5.138254101*	*9.717178289*	*2.68E-16*	*1.11E-14*	*26.58579693*

NLGN4Y	0.532618152	6.325771247	2.9123426	0.005529097	0.029927193	-2.415105382		KANK1	-0.750778674	6.450249024	-9.711950221	2.75E-16	1.14E-14	26.55918584
SCUBE2	0.513204874	7.835710756	2.900828161	0.005703093	0.03062846	-2.442670111		NLRC3	0.578633097	6.935312777	9.70468365	2.86E-16	1.18E-14	26.52219963
MNDA	0.525036434	5.54532876	2.876751314	0.006083526	0.031850078	-2.500069471		SLCO2B1	1.51999774	7.521264055	9.68764507	3.12E-16	1.28E-14	26.43547919
LYVE1	-0.630253993	9.594318086	-2.865034759	0.006277077	0.032494369	-2.52788419		FIBIN	-1.132995823	6.394094867	-9.68296785	3.19E-16	1.31E-14	26.41167489
ID4	-0.525198414	8.993809424	-2.85182254	0.006502196	0.033181618	-2.559156597		DEF6	0.588485115	5.246360514	9.682638587	3.20E-16	1.31E-14	26.40999915
SEMA3G	0.500512195	8.969082237	2.850721242	0.006521295	0.033219718	-2.56175883		CCRL1	-1.056149415	6.361201927	-9.680652482	3.23E-16	1.32E-14	26.3998912
PLA2G7	1.135815103	8.229559424	2.850294507	0.00652871	0.033249016	-2.562766968		NLRC4	0.675632418	4.923823436	9.675615194	3.32E-16	1.36E-14	26.37425518
ATP1B3	0.519648944	7.020592834	2.843370433	0.006650119	0.033570629	-2.579110243		FOLR2	1.369872776	7.516685568	9.667309615	3.46E-16	1.41E-14	26.33198728
CCL18	-1.076137931	7.405868219	-2.832515559	0.006844677	0.034196012	-2.604676651		STRA6	0.809995868	4.746139921	9.665457746	3.50E-16	1.42E-14	26.32256315
CHI3L1	1.058315977	8.296542745	2.824133827	0.006998507	0.034764702	-2.624372025		KLHL17	0.51507275	6.741910179	9.66103501	3.58E-16	1.45E-14	26.30005627
HLA-DQA1	0.81074661	8.531375855	2.823589887	0.0070086	0.034797543	-2.625648783		MBOAT7	0.575439631	7.187539191	9.657662519	3.64E-16	1.48E-14	26.28289429
FABP5P3	0.533553914	10.10633244	2.821972112	0.007038698	0.03489497	-2.629445088		IGF2R	0.617286932	6.754524609	9.655790899	3.68E-16	1.49E-14	26.27337008
IGHM	-0.622559719	7.567306361	-2.818176818	0.007109779	0.035160163	-2.638345307		RECQL4	0.688657861	5.996901266	9.64962912	3.79E-16	1.53E-14	26.24201492
SNORD116-15	-0.899921258	9.445614519	-2.814532756	0.007178654	0.035366079	-2.646883088		CYBB	1.116054937	7.776684391	9.648090063	3.83E-16	1.54E-14	26.23418334
CD300LB	0.502227635	6.370147838	2.802734229	0.007405917	0.036124177	-2.674473725		LOC401093	-0.93539946	5.038142289	-9.647445122	3.84E-16	1.55E-14	26.23090154
MMP7	0.728693451	5.502067869	2.794847767	0.007561524	0.036632887	-2.69287122		AKAP12	-1.108058346	6.807720406	-9.647329846	3.84E-16	1.55E-14	26.23031495
SNORD15B	-0.698322321	7.711787138	-2.79129451	0.007632617	0.036850737	-2.701148459		PLA2G2A	-3.24052547	6.387660754	-9.644394503	3.90E-16	1.57E-14	26.2153785
CADM3	-0.536488078	6.898385497	-2.78185766	0.007824443	0.037470724	-2.723095797		NAIP	0.730748379	5.958447556	9.639799816	3.99E-16	1.59E-14	26.19199893
SFR1	-0.512219663	4.837967335	-2.775773216	0.007950479	0.03785663	-2.73721892		GAS2L3	0.874036346	5.150161397	9.635284997	4.09E-16	1.63E-14	26.16902628
SNORD54	0.608738983	5.840247399	2.774473974	0.007977635	0.037949772	-2.740231902		S100A10	0.68728808	9.578449623	9.632063269	4.16E-16	1.65E-14	26.15263354
PROX1	-0.526692588	4.983804342	-2.772253877	0.008024235	0.038126085	-2.745378094		PCDH20	-1.244535778	5.059736267	-9.631145893	4.17E-16	1.66E-14	26.14796582
TM4SF19-TCTEX1D2	1.138775087	8.505033278	2.76885956	0.00809597	0.038339337	-2.753240556		PFKFB4	0.803568246	5.608511976	9.623789128	4.34E-16	1.72E-14	26.11053443

RASGEF1B	-0.634367374	7.218596122	-2.768503791	0.008103523	0.038365104	-2.754064254		*CD300A*	1.273745661	5.852943768	9.623632035	4.34E-16	1.72E-14	26.10973516
RGS2	-0.803410433	7.344938192	-2.768114776	0.008111789	0.038386967	-2.754964842		*CYP4B1*	-0.569573318	4.0499644	-9.610748717	4.64E-16	1.82E-14	26.044188
MTND2P28	-0.849902029	10.20931443	-2.760636694	0.008272215	0.038879214	-2.772259696		*GSTA2*	-0.772652339	4.934180389	-9.606482308	4.74E-16	1.86E-14	26.02248253
SCARNA5	-0.685849687	8.309986411	-2.753493359	0.008428192	0.039426897	-2.788749696		*HS3ST1*	1.017942259	6.277027844	9.606135069	4.75E-16	1.86E-14	26.02071596
ZNF737	0.575421823	7.695928121	2.748617616	0.008536211	0.03973558	-2.799987832		*RASGRP4*	0.664508111	5.308277703	9.604946599	4.78E-16	1.87E-14	26.01466969
ZNF117	0.630393879	9.398714457	2.737620193	0.008784561	0.040480558	-2.82528434		*CD68*	1.573211121	8.317372025	9.602739925	4.83E-16	1.88E-14	26.00344346
EVI2B	0.50463607	7.5088266	2.732723283	0.008897277	0.040828209	-2.836525282		*SELPLG*	0.909202273	5.678272483	9.59943254	4.92E-16	1.91E-14	25.98661772
OCLN	0.537079864	5.433267447	2.725235325	0.009072216	0.041469135	-2.853686493		*GINS3*	0.54366347	5.781642052	9.598448624	4.94E-16	1.92E-14	25.98161228
FCER1G	0.669217496	8.353998521	2.717824149	0.009248479	0.04199633	-2.870638875		*LILRB2*	1.323200995	7.482893299	9.596258771	5.00E-16	1.94E-14	25.97047202
LAPTM5	0.742128366	10.14103984	2.716308536	0.009284912	0.042133054	-2.874101665		*XLOC_l2_014602*	-0.724151247	5.883770606	-9.583335741	5.34E-16	2.06E-14	25.90473242
HCST	0.532331692	7.667465284	2.690709705	0.009920589	0.044018348	-2.932380675		*SUSD5*	-1.32006102	7.016919987	-9.583226668	5.35E-16	2.06E-14	25.90417759
CCL21	-0.504377028	6.765223237	-2.685201975	0.010062491	0.044401576	-2.944868224		*HVCN1*	0.793468406	5.83703924	9.583222182	5.35E-16	2.06E-14	25.90415477
HLA-DRB1	0.907732309	7.527531125	2.683929737	0.010095534	0.044478648	-2.947750137		*TASP1*	-0.664985106	5.823154511	-9.583043087	5.35E-16	2.06E-14	25.90324374
CACNA2D1	0.509946706	9.413580294	2.65873446	0.010770803	0.04659299	-3.004621223		*DHX58*	0.622055359	7.577070695	9.582554649	5.37E-16	2.06E-14	25.90075915
SNX10	0.628670012	8.469158845	2.655760864	0.010853179	0.046767735	-3.011307795		*HEXIM1*	-0.679731108	6.488311527	-9.58068219	5.42E-16	2.08E-14	25.89123436
PSMB8	0.522033115	7.747670111	2.643645513	0.011194817	0.047827244	-3.038495105		*NIPSNAP3B*	-0.515136489	4.75920621	-9.579124612	5.46E-16	2.10E-14	25.88331138
CLDN1	-0.905985982	5.158068161	-2.62547592	0.011725697	0.049375385	-3.079099588		*DENND2D*	1.486896134	7.003527114	9.571777235	5.67E-16	2.16E-14	25.84593818
SDS	0.56186908	6.172688	2.622745566	0.011807437	0.049604635	-3.085183673		*SLC37A2*	1.273089417	5.823099692	9.570698205	5.70E-16	2.17E-14	25.84044972
								CECR1	1.680833838	7.815533975	9.562498808	5.95E-16	2.26E-14	25.79874474
								TBC1D2	1.313528866	7.92807929	9.551850693	6.29E-16	2.37E-14	25.74458771
								P2RX7	1.046313269	5.485694589	9.549182803	6.37E-16	2.40E-14	25.73101918
								NRXN3	-0.677254316	4.51313203	-9.545734719	6.49E-16	2.44E-14	25.713483

								LOC100507239	0.67605741	4.857375157	9.541989244	6.62E-16	2.49E-14	25.69443477
								NPAS2	-0.527053624	4.621972705	-9.537568403	6.77E-16	2.54E-14	25.67195241
								MKNK1	0.563127153	6.45709622	9.535985238	6.82E-16	2.56E-14	25.66390131
								IPO5	-0.518036949	6.768182307	-9.533422412	6.91E-16	2.59E-14	25.65086837
								PTPN7	0.925123104	5.196925489	9.526626832	7.16E-16	2.68E-14	25.61631126
								AIF1	0.543396536	6.890786258	9.524367941	7.24E-16	2.70E-14	25.60482459
								GIMAP2	0.802608078	6.051167114	9.520172628	7.40E-16	2.76E-14	25.58349148
								SETDB2	0.501836507	6.826068129	9.510631508	7.78E-16	2.88E-14	25.53497711
								NPR1	-1.132578435	6.60319081	-9.497349441	8.33E-16	3.07E-14	25.46744587
								CASQ1	-0.614786198	4.304086135	-9.493792484	8.48E-16	3.12E-14	25.44936191
								ADCY7	0.935621654	5.89890123	9.487449658	8.77E-16	3.22E-14	25.41711533
								DPEP2	1.363524124	5.707113043	9.486177757	8.82E-16	3.24E-14	25.41064922
								ZNF469	0.856277963	6.214366393	9.47648607	9.28E-16	3.38E-14	25.3613803
								RGS10	0.910284582	9.348703287	9.468793627	9.65E-16	3.51E-14	25.32227711
								SNORD116-19	-0.947978805	6.963069372	-9.465171668	9.83E-16	3.57E-14	25.30386624
								FCGR2A	1.218822793	7.971086802	9.459210123	1.01E-15	3.68E-14	25.27356397
								LINC00112	-0.60787485	4.381626626	-9.453093783	1.05E-15	3.79E-14	25.2424762
								CD2	0.560272707	4.973424052	9.451296585	1.06E-15	3.82E-14	25.23334177
								FOXN3	-0.556468434	7.149492406	-9.450981725	1.06E-15	3.83E-14	25.23174147
								LOC729970	-0.525583043	5.223338149	-9.449193515	1.07E-15	3.85E-14	25.22265286
								TMEM136	-0.525006165	5.928342459	-9.447030357	1.08E-15	3.89E-14	25.21165872
								CTSB	1.530168405	9.247942613	9.446371071	1.08E-15	3.90E-14	25.20830797

								VLDLR	*-0.527130059*	*5.058998624*	*-9.419322164*	*1.25E-15*	*4.45E-14*	*25.07084863*
								DENND1C	*0.934814639*	*5.528348066*	*9.416125464*	*1.27E-15*	*4.52E-14*	*25.0546052*
								NT5DC3	*-0.872142546*	*5.47105328*	*-9.414504726*	*1.28E-15*	*4.55E-14*	*25.04636988*
								RASGRP3	*0.861529615*	*5.446261935*	*9.412832474*	*1.29E-15*	*4.59E-14*	*25.03787291*
								C2orf84	*-0.510196032*	*5.018002515*	*-9.402389943*	*1.36E-15*	*4.82E-14*	*24.98481526*
								HMOX1	*1.838644304*	*9.410675512*	*9.395123513*	*1.41E-15*	*5.00E-14*	*24.94789766*
								RSL24D1	*-0.571249432*	*7.303010916*	*-9.393880108*	*1.42E-15*	*5.02E-14*	*24.94158066*
								REEP1	*-1.230648114*	*5.874626932*	*-9.38914158*	*1.46E-15*	*5.14E-14*	*24.9175076*
								CD40LG	*0.920409846*	*5.141403899*	*9.382576798*	*1.51E-15*	*5.30E-14*	*24.88415814*
								CD14	*1.312378971*	*10.57228729*	*9.381719595*	*1.51E-15*	*5.32E-14*	*24.87980363*
								LILRB3	*1.39594185*	*7.442597104*	*9.377557863*	*1.54E-15*	*5.43E-14*	*24.85866286*
								RNASE6	*1.197304905*	*6.83127802*	*9.376320137*	*1.55E-15*	*5.45E-14*	*24.85237559*
								IFI30	*1.781075603*	*9.867828129*	*9.374724331*	*1.57E-15*	*5.49E-14*	*24.84426948*
								TACC3	*0.803524471*	*5.210254832*	*9.373896615*	*1.57E-15*	*5.51E-14*	*24.84006502*
								TMC6	*1.001880904*	*6.000868431*	*9.367560782*	*1.63E-15*	*5.68E-14*	*24.80788256*
								CHKB	*0.515332874*	*7.123141185*	*9.366769445*	*1.63E-15*	*5.70E-14*	*24.80386313*
								SEPT10	*-0.527781353*	*8.131933045*	*-9.365417593*	*1.64E-15*	*5.73E-14*	*24.79699674*
								VENTX	*0.857389168*	*5.432506809*	*9.364885173*	*1.65E-15*	*5.74E-14*	*24.79429248*
								MPP6	*-0.636596704*	*4.916701995*	*-9.364052011*	*1.66E-15*	*5.76E-14*	*24.7900607*
								SLC36A1	*0.921022099*	*7.032707491*	*9.362122901*	*1.67E-15*	*5.80E-14*	*24.78026252*
								JAK3	*1.144407866*	*7.837887707*	*9.356104325*	*1.73E-15*	*5.96E-14*	*24.74969445*
								HLA-DPB2	*1.233249544*	*7.605957872*	*9.353310434*	*1.75E-15*	*6.03E-14*	*24.73550494*

								FRY	*-0.649559371*	*5.660441527*	*-9.350935518*	*1.77E-15*	*6.10E-14*	*24.72344355*
								PRR11	*0.53729058*	*4.593300953*	*9.346681246*	*1.81E-15*	*6.22E-14*	*24.70183816*
								PDLIM1	*-0.624031502*	*6.69086957*	*-9.346591091*	*1.81E-15*	*6.22E-14*	*24.70138032*
								TPST2	*0.828539383*	*7.153203094*	*9.341368529*	*1.86E-15*	*6.38E-14*	*24.67485854*
								CYB561	*0.597074137*	*7.021952363*	*9.338842476*	*1.89E-15*	*6.45E-14*	*24.66203088*
								HECW2	*0.622188186*	*5.823373008*	*9.333032987*	*1.94E-15*	*6.64E-14*	*24.63253052*
								GPR162	*0.679527894*	*6.388306893*	*9.325360527*	*2.02E-15*	*6.88E-14*	*24.59357234*
								LOC100509553	*-0.816300033*	*6.259395967*	*-9.321694543*	*2.06E-15*	*6.98E-14*	*24.57495861*
								HLA-B	*0.919372911*	*9.628863012*	*9.321573091*	*2.06E-15*	*6.98E-14*	*24.57434196*
								S100Z	*0.860018072*	*4.984714964*	*9.321097089*	*2.07E-15*	*6.99E-14*	*24.57192514*
								ZFAND1	*-0.580020951*	*6.524382795*	*-9.31702423*	*2.11E-15*	*7.11E-14*	*24.55124635*
								C2	*1.455779245*	*6.5903253*	*9.312020676*	*2.17E-15*	*7.27E-14*	*24.52584324*
								DPRXP4	*0.907358398*	*6.128309738*	*9.311152774*	*2.18E-15*	*7.29E-14*	*24.521437*
								UCN	*0.539655896*	*6.125541188*	*9.308606869*	*2.20E-15*	*7.37E-14*	*24.50851194*
								MAP2	*-0.521640121*	*5.516060876*	*-9.303524947*	*2.26E-15*	*7.55E-14*	*24.48271289*
								ICA1	*0.538209834*	*5.76937651*	*9.301211144*	*2.29E-15*	*7.62E-14*	*24.47096695*
								GULP1	*-0.826707256*	*6.185404686*	*-9.301128348*	*2.29E-15*	*7.62E-14*	*24.47054665*
								RIMKLB	*-0.630536124*	*5.690165606*	*-9.297735*	*2.33E-15*	*7.74E-14*	*24.4533209*
								CYBA	*1.168077832*	*8.251352498*	*9.297199731*	*2.34E-15*	*7.76E-14*	*24.45060374*
								MYOT	*-0.570320135*	*4.033079496*	*-9.296096897*	*2.35E-15*	*7.79E-14*	*24.44500553*
								CAP2	*-0.733809425*	*5.309196368*	*-9.2914134*	*2.41E-15*	*7.96E-14*	*24.42123178*
								EMR2	*1.297096695*	*6.008557769*	*9.289453557*	*2.43E-15*	*8.03E-14*	*24.41128379*

								MOAP1	*-0.518583411*	*6.190769849*	*-9.28506718*	*2.49E-15*	*8.19E-14*	*24.38901956*
								C6orf145	*-0.647095935*	*6.956622378*	*-9.273516193*	*2.64E-15*	*8.66E-14*	*24.33039377*
								PILRA	*0.772449302*	*6.75961173*	*9.265125534*	*2.76E-15*	*8.99E-14*	*24.28781185*
								CYB561D2	*0.688120721*	*6.593838832*	*9.259350605*	*2.84E-15*	*9.23E-14*	*24.25850651*
								ANKRD13D	*0.512634984*	*7.730978307*	*9.255392338*	*2.90E-15*	*9.39E-14*	*24.2384209*
								BATF2	*0.633793136*	*5.60811816*	*9.249015702*	*3.00E-15*	*9.67E-14*	*24.20606527*
								HLA-DPB1	*1.006266498*	*8.363833374*	*9.246105751*	*3.04E-15*	*9.80E-14*	*24.19130057*
								LIPA	*1.49305546*	*9.031872363*	*9.243450788*	*3.08E-15*	*9.93E-14*	*24.17783001*
								PCDH12	*0.7746087*	*5.183800976*	*9.24019989*	*3.14E-15*	*1.01E-13*	*24.16133631*
								MYL2	*-0.935208651*	*5.547595657*	*-9.236633774*	*3.19E-15*	*1.03E-13*	*24.14324394*
								MAPKAPK3	*0.881808679*	*7.187525145*	*9.232988402*	*3.25E-15*	*1.04E-13*	*24.12475012*
								TMEM86A	*0.952079964*	*4.9509102*	*9.223924436*	*3.41E-15*	*1.09E-13*	*24.07876942*
								SPRY2	*-0.545829679*	*6.821091266*	*-9.223512536*	*3.42E-15*	*1.09E-13*	*24.07667998*
								LILRB1	*1.394237575*	*6.509709022*	*9.22208347*	*3.44E-15*	*1.10E-13*	*24.06943087*
								LOC729680	*-0.602403019*	*4.868569108*	*-9.221107293*	*3.46E-15*	*1.10E-13*	*24.06447914*
								CEBPE	*1.06175093*	*5.317398023*	*9.219282922*	*3.49E-15*	*1.11E-13*	*24.05522503*
								OBFC2A	*0.859250932*	*6.997295404*	*9.213000015*	*3.61E-15*	*1.14E-13*	*24.02335633*
								MEI1	*1.158765622*	*6.483525419*	*9.212384867*	*3.62E-15*	*1.15E-13*	*24.02023624*
								FGFRL1	*-0.689490241*	*5.619499248*	*-9.209352744*	*3.68E-15*	*1.16E-13*	*24.00485726*
								WDFY4	*0.683264437*	*5.213060235*	*9.206006677*	*3.74E-15*	*1.18E-13*	*23.98788651*
								ATP6AP1	*0.684664105*	*8.935358729*	*9.205961595*	*3.74E-15*	*1.18E-13*	*23.98765786*
								EPSTI1	*0.699265672*	*5.146311655*	*9.205109351*	*3.76E-15*	*1.18E-13*	*23.98333551*

								ATG7	*0.526775242*	*6.087821209*	*9.198172202*	*3.89E-15*	*1.22E-13*	*23.9481535*
								MYOZ1	*-0.978189556*	*5.488059448*	*-9.196038316*	*3.94E-15*	*1.23E-13*	*23.93733193*
								CA2	*1.400981127*	*6.068030692*	*9.193712442*	*3.98E-15*	*1.25E-13*	*23.925537*
								LAPTM5	*1.631006313*	*8.072224992*	*9.191436642*	*4.03E-15*	*1.26E-13*	*23.91399628*
								ROCK2	*-0.607141931*	*6.549584682*	*-9.189492511*	*4.07E-15*	*1.27E-13*	*23.90413769*
								FBXO25	*-0.637003905*	*7.455110641*	*-9.18619385*	*4.14E-15*	*1.29E-13*	*23.88741081*
								FMNL1	*0.783391553*	*5.192703246*	*9.183242457*	*4.20E-15*	*1.31E-13*	*23.87244535*
								NFIL3	*-1.239320428*	*7.929447158*	*-9.180446401*	*4.27E-15*	*1.32E-13*	*23.85826797*
								PTBP2	*-0.574147047*	*6.505052612*	*-9.178622677*	*4.31E-15*	*1.33E-13*	*23.84902102*
								RINL	*0.678029605*	*6.590980568*	*9.178501951*	*4.31E-15*	*1.33E-13*	*23.8484089*
								CDCA8	*0.740553269*	*5.376375033*	*9.1776798*	*4.33E-15*	*1.34E-13*	*23.84424036*
								ATP6V1A	*0.713748325*	*8.025535888*	*9.177131966*	*4.34E-15*	*1.34E-13*	*23.84146271*
								LOC153684	*0.694444106*	*6.783261138*	*9.177045239*	*4.34E-15*	*1.34E-13*	*23.84102298*
								XLOC_011184	*-0.51990674*	*5.151093631*	*-9.177038045*	*4.34E-15*	*1.34E-13*	*23.8409865*
								PIK3CG	*0.891994532*	*5.837043385*	*9.170926416*	*4.48E-15*	*1.38E-13*	*23.81000013*
								LOC100505592	*1.273116969*	*5.751855819*	*9.16915282*	*4.52E-15*	*1.39E-13*	*23.80100826*
								SLC16A5	*0.843338393*	*7.40202506*	*9.16623335*	*4.59E-15*	*1.41E-13*	*23.78620735*
								BTN3A2	*0.614490156*	*5.867481536*	*9.165386617*	*4.61E-15*	*1.41E-13*	*23.78191473*
								SLC4A11	*0.609568356*	*4.877599267*	*9.164835739*	*4.62E-15*	*1.41E-13*	*23.77912201*
								TMEM47	*-1.140817263*	*6.53720879*	*-9.162050293*	*4.69E-15*	*1.43E-13*	*23.76500121*
								LOC643988	*0.917164611*	*5.33143547*	*9.160668335*	*4.72E-15*	*1.44E-13*	*23.75799553*
								SIRPA	*0.984765531*	*7.592565685*	*9.157356077*	*4.80E-15*	*1.46E-13*	*23.74120487*

								LDOC1	*-1.154037729*	*8.947951235*	*-9.154774117*	*4.87E-15*	*1.48E-13*	*23.72811668*
								HRH1	*-0.634727059*	*5.639074366*	*-9.153764592*	*4.89E-15*	*1.49E-13*	*23.72299941*
								MBOAT1	*0.633532909*	*6.109383407*	*9.149896485*	*4.99E-15*	*1.51E-13*	*23.70339254*
								PSTPIP1	*0.92077419*	*6.797342561*	*9.147790991*	*5.05E-15*	*1.53E-13*	*23.69272045*
								EID1	*-0.513480075*	*4.95801418*	*-9.146408862*	*5.08E-15*	*1.54E-13*	*23.685715*
								PLD2	*0.591468189*	*7.82163595*	*9.143983296*	*5.15E-15*	*1.56E-13*	*23.67342106*
								LOC100132909	*-0.616073093*	*5.076655962*	*-9.143570273*	*5.16E-15*	*1.56E-13*	*23.67132769*
								ACAP1	*0.598163159*	*4.64174494*	*9.143448729*	*5.16E-15*	*1.56E-13*	*23.67071166*
								FAM19A5	*-0.591865774*	*4.425261891*	*-9.142195328*	*5.19E-15*	*1.57E-13*	*23.66435898*
								AP1B1	*0.754630826*	*7.752305365*	*9.140521917*	*5.24E-15*	*1.58E-13*	*23.65587768*
								PTPRCAP	*0.852114796*	*5.460782602*	*9.139156247*	*5.28E-15*	*1.58E-13*	*23.64895622*
								STK32A	*-0.521534092*	*4.19126051*	*-9.133980106*	*5.42E-15*	*1.62E-13*	*23.62272354*
								CCDC107	*-0.514505308*	*8.629310848*	*-9.131068859*	*5.50E-15*	*1.64E-13*	*23.60797001*
								TAP1	*0.525147887*	*8.179907996*	*9.122082489*	*5.76E-15*	*1.71E-13*	*23.5624322*
								ADRB2	*0.856229384*	*6.294870807*	*9.114732973*	*5.98E-15*	*1.77E-13*	*23.52519248*
								NFYB	*-0.694420645*	*6.4219695*	*-9.110591814*	*6.11E-15*	*1.81E-13*	*23.50421076*
								LCK	*0.898544556*	*5.345087338*	*9.096599481*	*6.57E-15*	*1.94E-13*	*23.43332422*
								SH3RF1	*-0.500234825*	*6.569683593*	*-9.096438635*	*6.57E-15*	*1.94E-13*	*23.43250943*
								CD33	*0.840734526*	*5.721999524*	*9.088465884*	*6.85E-15*	*2.02E-13*	*23.39212393*
								FERMT3	*1.164388805*	*6.308226499*	*9.088293799*	*6.85E-15*	*2.02E-13*	*23.39125228*
								CHMP4C	*-0.525087655*	*4.228379704*	*-9.087431996*	*6.88E-15*	*2.02E-13*	*23.38688711*
								LY86	*1.380807478*	*7.678262024*	*9.086795266*	*6.91E-15*	*2.02E-13*	*23.38366199*

								RPS6KA1	*0.745396255*	*5.277263613*	*9.086615635*	*6.91E-15*	*2.02E-13*	*23.38275215*
								TNFRSF21	*1.273751508*	*7.176851871*	*9.08482987*	*6.98E-15*	*2.04E-13*	*23.37370718*
								PLP1	*-0.917082682*	*4.369290502*	*-9.083939005*	*7.01E-15*	*2.05E-13*	*23.36919498*
								LOC283070	*0.545331599*	*7.059195223*	*9.080070267*	*7.15E-15*	*2.08E-13*	*23.34960054*
								XLOC_l2_006404	*0.578804004*	*6.600618577*	*9.073439797*	*7.40E-15*	*2.15E-13*	*23.31602053*
								TUFT1	*-0.542859459*	*6.722685906*	*-9.070967529*	*7.49E-15*	*2.17E-13*	*23.30350042*
								SPHAR	*-0.511399492*	*6.581318842*	*-9.070756413*	*7.50E-15*	*2.17E-13*	*23.3024313*
								PPP1CA	*0.546927789*	*7.08191948*	*9.069258019*	*7.56E-15*	*2.19E-13*	*23.2948433*
								GPRC5A	*-0.984009568*	*4.62907129*	*-9.068080622*	*7.60E-15*	*2.20E-13*	*23.28888095*
								PHYHIP	*-1.15159995*	*5.086105147*	*-9.055464997*	*8.11E-15*	*2.34E-13*	*23.22500068*
								PTGER3	*-0.697904851*	*5.462921506*	*-9.054524401*	*8.15E-15*	*2.35E-13*	*23.22023829*
								CMTM7	*0.892472681*	*6.546205694*	*9.047155905*	*8.47E-15*	*2.43E-13*	*23.18293229*
								LRRN4CL	*-1.253068144*	*5.289455504*	*-9.041494371*	*8.72E-15*	*2.49E-13*	*23.15427078*
								LHFPL2	*0.942225262*	*8.134388759*	*9.035360575*	*9.00E-15*	*2.57E-13*	*23.12322072*
								MTHFD2	*-0.562009902*	*7.421928821*	*-9.034734358*	*9.03E-15*	*2.57E-13*	*23.12005086*
								MATK	*1.131778641*	*5.119440655*	*9.031653778*	*9.17E-15*	*2.61E-13*	*23.10445759*
								LAIR1	*1.27072634*	*7.19852375*	*9.028233655*	*9.33E-15*	*2.65E-13*	*23.08714633*
								LYRM1	*-0.574760579*	*5.797368067*	*-9.027981669*	*9.34E-15*	*2.65E-13*	*23.08587091*
								DOK6	*-0.602080386*	*4.709289096*	*-9.026770027*	*9.40E-15*	*2.67E-13*	*23.07973828*
								KCNMB1	*-0.685849495*	*6.970028292*	*-9.026550295*	*9.41E-15*	*2.67E-13*	*23.07862613*
								FLNC	*-1.349067478*	*6.259069603*	*-9.019112103*	*9.78E-15*	*2.76E-13*	*23.04098045*
								LOC374443	*0.508884441*	*5.492245557*	*9.016546863*	*9.91E-15*	*2.80E-13*	*23.02799825*

								RASSF2	0.562043571	6.474507053	9.009743866	1.03E-14	2.89E-13	22.99357162
								ARHGAP10	-0.532954777	6.740119463	-9.007958528	1.04E-14	2.91E-13	22.9845374
								OXCT1	-0.602200739	6.335060797	-9.003121177	1.06E-14	2.97E-13	22.96006034
								PTGER4	0.804340663	7.59291483	8.994656262	1.11E-14	3.09E-13	22.91723146
								XLOC_000101	-0.530321075	5.594435371	-8.985840539	1.16E-14	3.23E-13	22.87263268
								ADAM8	1.994255296	8.51964657	8.985495514	1.16E-14	3.23E-13	22.8708873
								C17orf56	0.626206231	8.133685658	8.981115598	1.19E-14	3.30E-13	22.84873132
								OSR1	-0.729820443	4.577213927	-8.974552533	1.23E-14	3.41E-13	22.8155342
								CALM1	-0.52630923	7.017781074	-8.972499994	1.24E-14	3.44E-13	22.8051527
								C6orf115	0.884015067	7.502595157	8.968632887	1.27E-14	3.50E-13	22.7855941
								LOC100506553	-0.612364708	4.907472294	-8.966513346	1.28E-14	3.54E-13	22.77487456
								MPP1	0.900451443	7.064627587	8.9631686	1.30E-14	3.59E-13	22.75795921
								UST	-0.541899761	5.196705541	-8.961466673	1.32E-14	3.62E-13	22.74935236
								NGRN	-0.586711117	6.820183547	-8.959050578	1.33E-14	3.66E-13	22.73713421
								KCNE3	0.953138687	5.612141925	8.957792485	1.34E-14	3.68E-13	22.73077222
								CD74	1.201187119	7.511538156	8.957089568	1.34E-14	3.69E-13	22.72721772
								TTC21A	0.602736441	6.5247222	8.956293583	1.35E-14	3.70E-13	22.72319264
								TNNI2	0.971406402	5.434107647	8.953001963	1.37E-14	3.76E-13	22.70654825
								GRAMD4	0.861177855	6.226326734	8.947629916	1.41E-14	3.86E-13	22.67938561
								CCL5	1.441908144	6.823305595	8.944230005	1.44E-14	3.92E-13	22.66219569
								TLR1	1.091820921	6.588434577	8.935436162	1.50E-14	4.09E-13	22.61773787
								NXPH3	-0.902093231	6.092868098	-8.932982371	1.52E-14	4.14E-13	22.60533354

								DIXDC1	*-0.672020683*	*7.321921863*	*-8.927167935*	*1.57E-14*	*4.26E-13*	*22.57594228*
								FAM49B	*0.888441932*	*7.33620676*	*8.923662242*	*1.60E-14*	*4.33E-13*	*22.55822259*
								BVES	*-0.714313264*	*5.215744562*	*-8.912705524*	*1.69E-14*	*4.56E-13*	*22.502847*
								NLRP12	*1.022809806*	*5.06612965*	*8.912251682*	*1.69E-14*	*4.57E-13*	*22.50055346*
								GLB1	*0.688348206*	*6.87077883*	*8.911009428*	*1.70E-14*	*4.59E-13*	*22.49427565*
								CSF3R	*1.385876161*	*7.094837745*	*8.910177333*	*1.71E-14*	*4.61E-13*	*22.49007067*
								ETNK2	*-0.590346924*	*5.260047993*	*-8.90143113*	*1.79E-14*	*4.81E-13*	*22.44587489*
								GAL3ST1	*-0.502764183*	*4.75433734*	*-8.900966548*	*1.79E-14*	*4.81E-13*	*22.44352744*
								FCGR1B	*1.336403249*	*7.291644276*	*8.897090132*	*1.83E-14*	*4.90E-13*	*22.42394128*
								PPP2R2B	*-1.117603549*	*6.678482317*	*-8.896310701*	*1.84E-14*	*4.91E-13*	*22.42000322*
								CD27	*0.778765341*	*5.345834532*	*8.893099291*	*1.87E-14*	*4.98E-13*	*22.40377811*
								SNORA42	*-0.720262386*	*6.465346606*	*-8.888832087*	*1.91E-14*	*5.09E-13*	*22.38221995*
								ACRBP	*0.863022715*	*5.397575579*	*8.882287767*	*1.97E-14*	*5.26E-13*	*22.34916028*
								MIR100HG	*-0.674080656*	*6.746295662*	*-8.877034302*	*2.03E-14*	*5.38E-13*	*22.32262386*
								LOC100652912	*0.651378673*	*5.999484727*	*8.875181086*	*2.05E-14*	*5.42E-13*	*22.31326335*
								ZNF683	*1.038252163*	*5.325413846*	*8.874924289*	*2.05E-14*	*5.42E-13*	*22.3119663*
								ECM1	*0.769557576*	*7.060096167*	*8.874352611*	*2.06E-14*	*5.43E-13*	*22.30907883*
								TFEC	*1.081586514*	*5.395687245*	*8.874297612*	*2.06E-14*	*5.43E-13*	*22.30880104*
								HLA-DMB	*1.425915221*	*9.248003557*	*8.869290177*	*2.11E-14*	*5.56E-13*	*22.28351032*
								C1QA	*1.387417947*	*8.522368592*	*8.868359216*	*2.12E-14*	*5.59E-13*	*22.27880858*
								REC8	*0.666482817*	*6.486807635*	*8.86739438*	*2.13E-14*	*5.61E-13*	*22.27393583*
								GCNT1	*0.687558703*	*5.26630033*	*8.867275022*	*2.13E-14*	*5.61E-13*	*22.27333303*

								GPX3	-0.789535338	7.646130615	-8.865230859	2.16E-14	5.66E-13	22.26300956
								LOC100506585	1.306777589	5.680341855	8.861394088	2.20E-14	5.76E-13	22.24363387
								ZNF831	0.662176858	4.582278392	8.860084984	2.21E-14	5.79E-13	22.23702315
								SCHIP1	-0.888312996	7.823703432	-8.859371897	2.22E-14	5.81E-13	22.23342226
								SNORD109B	-0.50896936	5.149089196	-8.857438372	2.24E-14	5.86E-13	22.22365868
								CTSD	1.412069485	8.983312094	8.852873755	2.30E-14	5.99E-13	22.20061019
								HN1	1.026243736	7.522263811	8.847672307	2.36E-14	6.13E-13	22.17434804
								KLRG1	0.521348947	4.833693448	8.846240608	2.38E-14	6.17E-13	22.16711974
								PLD5	-0.612597433	4.209530946	-8.845752112	2.38E-14	6.18E-13	22.16465348
								XLOC_l2_006398	-0.543720594	4.322687178	-8.842724964	2.42E-14	6.27E-13	22.14937078
								SEL1L3	0.875645225	6.238326884	8.84171142	2.43E-14	6.30E-13	22.14425402
								PNMAL1	-0.987345389	6.978900654	-8.839979854	2.45E-14	6.35E-13	22.13551258
								CRYAB	-1.117453349	9.208898849	-8.832100347	2.55E-14	6.59E-13	22.09573751
								NEXN	-1.066758088	7.948655918	-8.825791145	2.64E-14	6.77E-13	22.06389268
								OTUD7B	-0.60399602	5.566233303	-8.820259729	2.71E-14	6.96E-13	22.03597618
								RNF149	0.584289853	8.273767628	8.816920538	2.76E-14	7.07E-13	22.01912478
								HOXB6	0.849370652	6.303365033	8.816060629	2.77E-14	7.09E-13	22.01478535
								CD6	1.103061199	5.555446858	8.815765208	2.78E-14	7.09E-13	22.01329455
								GBP5	1.1152232	6.279916365	8.811370704	2.84E-14	7.24E-13	21.9911192
								SLC6A6	0.589028343	6.213113811	8.808659134	2.88E-14	7.33E-13	21.97743696
								SLC7A7	1.218300499	7.120007161	8.797199887	3.05E-14	7.74E-13	21.91962151
								ASPHD1	1.176544443	5.491678075	8.784931965	3.25E-14	8.23E-13	21.85773772

								IL4I1	*2.021675362*	*7.605939947*	*8.783956522*	*3.27E-14*	*8.25E-13*	*21.85281775*
								TIAM1	*0.959008403*	*6.296944116*	*8.783381129*	*3.28E-14*	*8.27E-13*	*21.84991561*
								LAG3	*0.694564117*	*5.553450273*	*8.781518241*	*3.31E-14*	*8.34E-13*	*21.84051984*
								TNFRSF10C	*0.901043379*	*5.951810913*	*8.779784882*	*3.34E-14*	*8.41E-13*	*21.83177762*
								PATL2	*0.651147008*	*4.943280819*	*8.777704477*	*3.38E-14*	*8.49E-13*	*21.82128539*
								XLOC_l2_004318	*-0.580190193*	*4.338570073*	*-8.777468883*	*3.38E-14*	*8.49E-13*	*21.82009723*
								IL17B	*-1.140405924*	*5.399285922*	*-8.777224157*	*3.38E-14*	*8.49E-13*	*21.81886301*
								TMEM37	*0.655200081*	*5.552995757*	*8.775579307*	*3.41E-14*	*8.56E-13*	*21.81056776*
								IL2RB	*0.96694504*	*5.673242995*	*8.773821043*	*3.44E-14*	*8.62E-13*	*21.80170078*
								HLA-DQB1	*1.303002865*	*8.245704562*	*8.766005339*	*3.58E-14*	*8.95E-13*	*21.76228904*
								TDO2	*0.98492685*	*4.642639182*	*8.765725508*	*3.59E-14*	*8.96E-13*	*21.76087805*
								APOBEC3G	*0.717244251*	*6.066043165*	*8.764928394*	*3.60E-14*	*8.99E-13*	*21.75685879*
								PVRIG	*1.002784955*	*5.80978451*	*8.762219995*	*3.65E-14*	*9.10E-13*	*21.74320274*
								NCF2	*1.303741169*	*6.810124653*	*8.755646239*	*3.78E-14*	*9.39E-13*	*21.71005965*
								SMAD9	*-0.922067058*	*6.189545133*	*-8.751873191*	*3.85E-14*	*9.56E-13*	*21.6910386*
								SH2D3A	*0.614877521*	*4.869754618*	*8.748165434*	*3.93E-14*	*9.73E-13*	*21.67234785*
								IGLL5	*2.692043268*	*9.059863264*	*8.747014719*	*3.95E-14*	*9.78E-13*	*21.66654735*
								PAM	*-0.56925734*	*7.639536444*	*-8.746793128*	*3.95E-14*	*9.78E-13*	*21.66543037*
								MSX1	*-0.779052863*	*5.194416996*	*-8.74298598*	*4.03E-14*	*9.96E-13*	*21.64624024*
								ADAMTSL3	*-0.719231913*	*5.510861526*	*-8.736993221*	*4.16E-14*	*1.03E-12*	*21.61603588*
								AGRN	*0.503716013*	*6.266849562*	*8.736157064*	*4.18E-14*	*1.03E-12*	*21.61182177*
								AQP7P3	*-0.672433032*	*4.779870288*	*-8.736071487*	*4.18E-14*	*1.03E-12*	*21.61139048*

								GNS	0.630303798	9.414030821	8.733087472	4.24E-14	1.04E-12	21.59635199
								ALOX5	0.738053749	5.984833129	8.730739169	4.29E-14	1.05E-12	21.58451782
								RGS18	0.952868788	6.005718572	8.726998483	4.38E-14	1.07E-12	21.56566776
								KLHDC7B	0.645524654	4.89648547	8.725731123	4.40E-14	1.08E-12	21.55928155
								IQCJ-SCHIP1	-0.810845235	6.455391509	-8.723433493	4.46E-14	1.09E-12	21.54770418
								SLC15A3	1.331896804	7.857857455	8.720850714	4.52E-14	1.10E-12	21.53469054
								ATP6V0C	0.673084303	10.65291162	8.714311273	4.67E-14	1.14E-12	21.50174332
								HLA-DPA1	1.228179205	9.811932849	8.713127948	4.70E-14	1.14E-12	21.49578185
								FKBP15	0.784012133	7.319182529	8.705939638	4.87E-14	1.18E-12	21.45957049
								XLOC_l2_015033	0.625002812	4.853424416	8.704524323	4.91E-14	1.19E-12	21.45244132
								BEND7	-0.545702848	4.877658026	-8.702890331	4.95E-14	1.20E-12	21.44421085
								FCGR2C	1.148859819	7.172478254	8.698835374	5.05E-14	1.22E-12	21.42378692
								SIT1	0.532186315	4.472923939	8.698798252	5.06E-14	1.22E-12	21.42359996
								IL11RA	-0.660732249	6.946098871	-8.693922836	5.18E-14	1.25E-12	21.39904548
								TRIM36	-0.651579972	4.844101311	-8.693123393	5.20E-14	1.25E-12	21.39501937
								GPR132	0.761361117	5.326351733	8.691197414	5.26E-14	1.26E-12	21.38532011
								ACADL	-1.208479844	5.639329097	-8.690928761	5.26E-14	1.26E-12	21.38396719
								NRK	-0.740454174	5.126649501	-8.688112648	5.34E-14	1.28E-12	21.36978585
								GLA	0.932720001	8.088456604	8.687751951	5.35E-14	1.28E-12	21.36796951
								PTK2B	0.746355273	6.745467174	8.679219662	5.59E-14	1.33E-12	21.32500729
								RASL10A	1.34515168	6.522411672	8.679161674	5.59E-14	1.33E-12	21.32471533
								NPL	1.007042351	6.318897221	8.673712679	5.75E-14	1.36E-12	21.29728168

								ATP6V0B	0.648146203	8.628657468	8.670707471	5.84E-14	1.38E-12	21.28215273
								UCP2	1.431521561	6.702280049	8.668816552	5.89E-14	1.39E-12	21.27263379
								SECTM1	0.896515602	7.113238477	8.666796836	5.95E-14	1.41E-12	21.26246684
								GPR34	0.996613294	6.275437509	8.659870493	6.17E-14	1.45E-12	21.22760344
								RAC3	0.576733703	5.436467817	8.659311416	6.19E-14	1.46E-12	21.22478954
								FGL2	-0.809351285	6.103625277	-8.657596361	6.24E-14	1.47E-12	21.21615766
								C11orf35	0.730947395	5.565370498	8.657537689	6.24E-14	1.47E-12	21.21586236
								SNX20	0.501450571	4.360260159	8.657428952	6.25E-14	1.47E-12	21.2153151
								PYDC1	0.749021782	5.360243224	8.654045201	6.36E-14	1.49E-12	21.19828548
								DAB2	0.726043756	8.562333002	8.652082259	6.42E-14	1.50E-12	21.18840694
								PKD2	-0.71286788	7.588032169	-8.6511588	6.45E-14	1.51E-12	21.18375974
								CCRL2	0.941205554	5.174728738	8.650863551	6.46E-14	1.51E-12	21.18227394
								CLCN7	0.531870468	5.994931579	8.648917497	6.52E-14	1.52E-12	21.17248095
								NLGN1	-0.636046993	4.741670917	-8.647530697	6.57E-14	1.53E-12	21.16550247
								ARHGAP22	0.768588093	7.28928921	8.645410139	6.64E-14	1.55E-12	21.15483199
								CHI3L1	2.295229148	7.474474417	8.643945506	6.69E-14	1.56E-12	21.14746232
								GBA	0.555333143	6.875694971	8.643883271	6.69E-14	1.56E-12	21.14714917
								FNDC1	1.126381805	6.544819692	8.643518582	6.71E-14	1.56E-12	21.14531418
								SSTR1	-0.693163741	4.343028165	-8.643151464	6.72E-14	1.56E-12	21.14346697
								LGALS2	1.563521992	6.294572787	8.639662773	6.84E-14	1.58E-12	21.12591379
								TCEAL1	-0.639106178	7.831094078	-8.638419512	6.88E-14	1.59E-12	21.11965864
								FBLN1	-1.500694444	8.458674263	-8.633173444	7.07E-14	1.63E-12	21.093266

								CD4	*0.546824891*	*6.618303971*	*8.631813641*	*7.12E-14*	*1.64E-12*	*21.08642533*
								GINS1	*0.662403437*	*5.172996272*	*8.63051671*	*7.17E-14*	*1.65E-12*	*21.07990111*
								SIDT1	*0.644931779*	*4.815723102*	*8.627533271*	*7.28E-14*	*1.67E-12*	*21.06489348*
								CD37	*1.303686977*	*6.19068512*	*8.626600004*	*7.31E-14*	*1.68E-12*	*21.06019903*
								PRKCD	*0.622647616*	*6.697295977*	*8.622179322*	*7.48E-14*	*1.72E-12*	*21.03796353*
								GPSM3	*0.800371266*	*6.759762299*	*8.617276159*	*7.67E-14*	*1.76E-12*	*21.01330333*
								C6orf168	*-0.573758504*	*4.337667504*	*-8.611735877*	*7.89E-14*	*1.81E-12*	*20.9854415*
								SKAP1	*0.896293571*	*5.839746893*	*8.611326969*	*7.91E-14*	*1.81E-12*	*20.98338523*
								NAV2	*-0.578001125*	*6.152818699*	*-8.609995367*	*7.96E-14*	*1.82E-12*	*20.97668914*
								PAQR4	*0.553702621*	*4.605133186*	*8.605331474*	*8.15E-14*	*1.86E-12*	*20.95323763*
								SAP18	*-0.596391133*	*5.678147161*	*-8.604550067*	*8.19E-14*	*1.86E-12*	*20.94930868*
								CARD16	*0.778130491*	*6.280251421*	*8.604031478*	*8.21E-14*	*1.87E-12*	*20.94670121*
								CCDC136	*-0.509483326*	*5.477213172*	*-8.60376152*	*8.22E-14*	*1.87E-12*	*20.94534387*
								LOC100507150	*-1.358242023*	*5.487932472*	*-8.599677644*	*8.39E-14*	*1.90E-12*	*20.92481117*
								SLC35C1	*0.504320836*	*6.478056176*	*8.599221856*	*8.41E-14*	*1.91E-12*	*20.92251968*
								FGR	*1.449126256*	*8.222699681*	*8.598396669*	*8.45E-14*	*1.91E-12*	*20.91837107*
								YAP1	*-0.901195303*	*8.025695732*	*-8.597543957*	*8.48E-14*	*1.92E-12*	*20.91408415*
								WFDC3	*-0.667586944*	*5.914853854*	*-8.597529976*	*8.48E-14*	*1.92E-12*	*20.91401387*
								CARD6	*0.621897069*	*6.946616562*	*8.593705512*	*8.65E-14*	*1.96E-12*	*20.89478763*
								FAM129A	*-0.643517793*	*9.465530516*	*-8.590693091*	*8.79E-14*	*1.98E-12*	*20.87964466*
								TCEAL5	*-0.624149321*	*8.457726599*	*-8.588614768*	*8.88E-14*	*2.00E-12*	*20.86919777*
								NPC2	*0.842621576*	*10.40621536*	*8.583997369*	*9.09E-14*	*2.04E-12*	*20.84598943*

								SPP1	*2.790325767*	*9.969399803*	*8.583202984*	*9.13E-14*	*2.05E-12*	*20.84199684*
								EXT1	*-0.59121604*	*6.545756008*	*-8.582022651*	*9.18E-14*	*2.06E-12*	*20.83606458*
								LRRC8A	*-0.51066776*	*8.844998471*	*-8.581779916*	*9.19E-14*	*2.06E-12*	*20.83484462*
								FRMD4B	*0.747007579*	*6.442477759*	*8.568089437*	*9.86E-14*	*2.20E-12*	*20.76604748*
								BRI3BP	*0.529146833*	*5.061450172*	*8.565549212*	*9.99E-14*	*2.22E-12*	*20.75328439*
								MSR1	*1.113305549*	*6.18042267*	*8.562952098*	*1.01E-13*	*2.25E-12*	*20.74023612*
								MS4A6A	*1.14288914*	*6.908795707*	*8.561893752*	*1.02E-13*	*2.26E-12*	*20.73491904*
								ARRDC4	*0.545650948*	*6.77457795*	*8.560934632*	*1.02E-13*	*2.27E-12*	*20.73010055*
								RCAN3	*0.672560921*	*5.158495162*	*8.559436883*	*1.03E-13*	*2.29E-12*	*20.72257624*
								HSPB2	*-0.701988985*	*8.427440107*	*-8.55343403*	*1.06E-13*	*2.35E-12*	*20.69242165*
								LDB3	*-0.912470815*	*5.573690099*	*-8.550112014*	*1.08E-13*	*2.39E-12*	*20.67573544*
								KCNB2	*-0.523993284*	*4.217798251*	*-8.54781111*	*1.09E-13*	*2.41E-12*	*20.66417883*
								TIFAB	*1.205203071*	*4.828740815*	*8.547385636*	*1.10E-13*	*2.42E-12*	*20.66204188*
								LOC100506897	*1.044598891*	*5.516809835*	*8.546979251*	*1.10E-13*	*2.42E-12*	*20.66000083*
								XLOC_013436	*-0.793699225*	*8.138375053*	*-8.543352422*	*1.12E-13*	*2.46E-12*	*20.64178595*
								CTSA	*0.707818757*	*8.398067366*	*8.540901091*	*1.13E-13*	*2.49E-12*	*20.62947546*
								LGR6	*-0.632698328*	*5.557054092*	*-8.538536651*	*1.15E-13*	*2.52E-12*	*20.6176019*
								UNC5B	*0.796055483*	*7.352436621*	*8.538114096*	*1.15E-13*	*2.52E-12*	*20.61548*
								FBXO2	*-0.781865852*	*5.125891009*	*-8.535458861*	*1.16E-13*	*2.55E-12*	*20.60214693*
								MXI1	*-0.582978801*	*6.696424137*	*-8.525908639*	*1.22E-13*	*2.67E-12*	*20.554197*
								XLOC_014161	*0.812438482*	*5.326404764*	*8.524208504*	*1.23E-13*	*2.68E-12*	*20.5456619*
								PTCRA	*1.376597131*	*5.458173205*	*8.520406822*	*1.26E-13*	*2.73E-12*	*20.52657755*

								APOD	-1.824374808	5.644553505	-8.5180152	1.27E-13	2.76E-12	20.51457242
								RHPN1	0.754740935	5.60348641	8.505262998	1.36E-13	2.93E-12	20.45057049
								VIT	-1.072193246	4.706481832	-8.504539323	1.36E-13	2.94E-12	20.44693894
								CSTB	1.205202362	9.465449637	8.50313003	1.37E-13	2.96E-12	20.43986698
								MYOCD	-1.041608295	5.179963914	-8.498433029	1.41E-13	3.02E-12	20.41629845
								MTSS1	0.599161026	5.513660641	8.497138762	1.42E-13	3.04E-12	20.40980449
								FRK	-0.71331765	4.900523714	-8.496631746	1.42E-13	3.04E-12	20.4072606
								PCDH11X	-0.667421042	4.266228361	-8.494594176	1.43E-13	3.07E-12	20.3970376
								LOC100652987	-0.501804345	6.931515398	-8.491480224	1.46E-13	3.12E-12	20.38141495
								CD109	0.682428985	7.577214899	8.487251611	1.49E-13	3.18E-12	20.36020166
								LIMCH1	-0.723471616	7.397123706	-8.485060415	1.51E-13	3.21E-12	20.34921003
								LOC100506965	-0.576275664	6.147195488	-8.481448695	1.53E-13	3.27E-12	20.33109375
								FUT4	0.53883303	6.100435184	8.475958122	1.58E-13	3.35E-12	20.30355579
								TCEAL6	-0.592468221	8.63334721	-8.473134025	1.60E-13	3.40E-12	20.28939278
								TFPI2	-1.340830793	6.647563933	-8.465660546	1.66E-13	3.52E-12	20.25191686
								TEAD3	-0.800183062	6.759681431	-8.465034447	1.67E-13	3.53E-12	20.24877753
								TST	0.508223444	7.776777103	8.463785267	1.68E-13	3.54E-12	20.24251415
								XLOC_008700	-0.827981382	5.190834128	-8.463452422	1.68E-13	3.55E-12	20.24084529
								HLA-DRB4	1.372368095	8.526928681	8.463294886	1.68E-13	3.55E-12	20.24005543
								PRUNE2	-0.958001315	7.677589995	-8.46289344	1.69E-13	3.55E-12	20.23804264
								SDC4	-0.66573331	7.315271373	-8.459382878	1.72E-13	3.61E-12	20.22044191
								TNFRSF11A	0.524902836	4.523723585	8.452529974	1.78E-13	3.73E-12	20.1860876

								LOC100507319	-0.612321865	6.364755753	-8.445850845	1.84E-13	3.85E-12	20.15260927
								RNASE1	1.260303345	10.45672008	8.445262734	1.84E-13	3.86E-12	20.14966166
								C20orf103	0.784713387	4.973032385	8.444119211	1.86E-13	3.87E-12	20.14393044
								KRTAP6-3	-0.647570069	4.717349781	-8.442042283	1.88E-13	3.91E-12	20.13352144
								ST3GAL6	0.662268808	5.891008396	8.440767004	1.89E-13	3.93E-12	20.12713032
								IRS2	-0.655045039	6.949020061	-8.437406234	1.92E-13	3.99E-12	20.1102885
								SLC39A14	-0.677236921	6.463526666	-8.435848929	1.94E-13	4.02E-12	20.10248479
								RBM47	0.812538851	5.495870777	8.431634573	1.98E-13	4.11E-12	20.08136782
								RRM2	0.502604847	4.372635364	8.430135887	1.99E-13	4.13E-12	20.07385879
								WBSCR17	-0.815080548	4.97304587	-8.428250788	2.01E-13	4.16E-12	20.06441401
								C13orf15	1.068058937	8.01012021	8.428053836	2.01E-13	4.16E-12	20.06342726
								TCEAL7	-0.576517554	4.900290341	-8.427917487	2.02E-13	4.16E-12	20.06274414
								LEFTY2	-0.996041968	4.837383256	-8.425355065	2.04E-13	4.21E-12	20.04990652
								BATF	1.109429991	7.030908692	8.421917394	2.08E-13	4.28E-12	20.03268507
								TGFBI	0.753823628	10.56032751	8.421289303	2.08E-13	4.29E-12	20.02953871
								SNORD108	-0.577577263	5.479162666	-8.417333869	2.13E-13	4.38E-12	20.00972532
								PDZD2	-0.531244606	4.661367598	-8.415541695	2.15E-13	4.41E-12	20.00074861
								TRGV7	0.765588792	4.746590388	8.413613097	2.17E-13	4.45E-12	19.99108896
								ADAP1	0.607181268	5.811081915	8.412227851	2.18E-13	4.48E-12	19.98415101
								FAM59A	-0.964861371	6.507504221	-8.409184561	2.22E-13	4.55E-12	19.96890957
								GNPTAB	0.504217017	6.746588258	8.403661084	2.28E-13	4.67E-12	19.94124942
								PARVG	0.687676678	5.037225757	8.402474626	2.29E-13	4.69E-12	19.93530838

								IDH1	*0.786991981*	*7.276115914*	*8.394188331*	*2.39E-13*	*4.88E-12*	*19.89382016*
								DOCK2	*0.749349186*	*4.996588444*	*8.392014207*	*2.42E-13*	*4.92E-12*	*19.88293591*
								PNMT	*-0.772186848*	*4.690285721*	*-8.391391831*	*2.43E-13*	*4.94E-12*	*19.87982023*
								PTPN21	*-0.775107009*	*6.499510177*	*-8.385153337*	*2.51E-13*	*5.08E-12*	*19.84859202*
								ATP8B4	*0.574161821*	*5.371088756*	*8.382990698*	*2.53E-13*	*5.13E-12*	*19.83776744*
								SLC45A3	*0.6252673*	*6.17348079*	*8.380160414*	*2.57E-13*	*5.20E-12*	*19.82360193*
								C3AR1	*0.757452835*	*7.809457924*	*8.373042194*	*2.66E-13*	*5.38E-12*	*19.78797936*
								EMILIN2	*1.180224508*	*8.588131103*	*8.372796009*	*2.67E-13*	*5.38E-12*	*19.78674744*
								HLA-DRA	*1.291430871*	*9.589292408*	*8.371031731*	*2.69E-13*	*5.43E-12*	*19.77791919*
								PLS3	*-0.764730604*	*8.687597316*	*-8.37075014*	*2.70E-13*	*5.43E-12*	*19.77651017*
								ADA	*0.683032518*	*7.747910672*	*8.370534736*	*2.70E-13*	*5.44E-12*	*19.77543234*
								ADRA1B	*-1.035784465*	*6.513602054*	*-8.369788654*	*2.71E-13*	*5.45E-12*	*19.77169918*
								FOXC2	*-0.535732335*	*4.771205208*	*-8.36863828*	*2.72E-13*	*5.48E-12*	*19.76594318*
								FOXC1	*-0.839601615*	*8.013810564*	*-8.3674783*	*2.74E-13*	*5.51E-12*	*19.76013926*
								VASN	*-0.770799727*	*8.401173345*	*-8.363153019*	*2.80E-13*	*5.62E-12*	*19.73849925*
								BHMT2	*-0.530582934*	*5.488704936*	*-8.362955646*	*2.80E-13*	*5.62E-12*	*19.73751182*
								SNX10	*0.993341075*	*5.407625619*	*8.362403594*	*2.81E-13*	*5.63E-12*	*19.73474999*
								HIST1H1C	*-0.78783689*	*8.473836062*	*-8.359610717*	*2.85E-13*	*5.70E-12*	*19.72077818*
								IQGAP3	*0.779533044*	*5.432083423*	*8.357373223*	*2.89E-13*	*5.76E-12*	*19.70958541*
								HEXA	*0.696714855*	*7.961047046*	*8.356729516*	*2.89E-13*	*5.77E-12*	*19.70636546*
								NUP210	*0.59591448*	*4.411450001*	*8.352167254*	*2.96E-13*	*5.90E-12*	*19.68354544*
								ANKRD58	*1.269582061*	*5.928308497*	*8.351398251*	*2.97E-13*	*5.92E-12*	*19.67969919*

								LPP	-0.94920554	6.922321839	-8.350983785	2.98E-13	5.93E-12	19.67762623
								NNMT	-0.657888146	10.48268677	-8.344220654	3.08E-13	6.12E-12	19.64380298
								XLOC_l2_002659	0.68143051	4.649988486	8.34326878	3.10E-13	6.15E-12	19.63904296
								PNPLA7	-0.503326657	4.525035469	-8.337767077	3.19E-13	6.30E-12	19.61153276
								PSMB9	0.54608033	7.695619441	8.335253983	3.23E-13	6.37E-12	19.59896768
								DDAH1	-0.566981755	6.159695519	-8.334780796	3.24E-13	6.38E-12	19.5966019
								TM4SF19	1.490119889	5.099543482	8.331643261	3.29E-13	6.48E-12	19.58091592
								TMEM51	1.044219223	7.704668449	8.322071286	3.45E-13	6.80E-12	19.53306828
								PART1	-0.753433969	4.831117494	-8.322069686	3.45E-13	6.80E-12	19.53306028
								PTPN22	0.777221499	5.228169365	8.314686637	3.58E-13	7.04E-12	19.4961618
								MT1E	-0.844616035	9.413071124	-8.314597656	3.59E-13	7.04E-12	19.49571714
								SEPT1	0.658055841	4.668403319	8.310352306	3.66E-13	7.19E-12	19.47450297
								LOC100653058	0.716540921	6.362896269	8.309019804	3.69E-13	7.23E-12	19.46784485
								LOC100508196	0.7401616	4.751106778	8.307175991	3.72E-13	7.28E-12	19.4586322
								GPR160	0.821039046	5.344956886	8.306105261	3.74E-13	7.31E-12	19.45328246
								LOC100170939	0.814430243	7.002402317	8.30538438	3.76E-13	7.34E-12	19.44968076
								SLC24A3	-0.831155341	7.601420902	-8.305120453	3.76E-13	7.34E-12	19.44836213
								PAG1	0.667481893	5.831268587	8.303116249	3.80E-13	7.40E-12	19.43834904
								UGDH	-0.5680689	7.330559546	-8.302320718	3.82E-13	7.42E-12	19.43437465
								TSPAN14	0.537199511	5.874357799	8.29950775	3.87E-13	7.52E-12	19.420322
								SLC40A1	0.998698037	8.200448994	8.294686345	3.97E-13	7.69E-12	19.39623804
								ZDHHC12	0.536270338	6.327015653	8.294485539	3.97E-13	7.69E-12	19.39523503

								C9orf139	0.735759373	4.87925771	8.29441015	3.97E-13	7.69E-12	19.39485847
								PREX1	0.780121575	8.33950529	8.292890894	4.00E-13	7.74E-12	19.38727009
								SH3BGR	-0.936744243	6.342510061	-8.291287214	4.04E-13	7.79E-12	19.37926031
								LILRA4	1.243947731	6.112749547	8.285839669	4.15E-13	7.98E-12	19.35205421
								PRKX	0.623615928	5.551830602	8.285649553	4.15E-13	7.99E-12	19.3511048
								CAPG	1.490679751	8.11082074	8.280203137	4.27E-13	8.20E-12	19.32390801
								IGSF6	1.284228207	5.787740955	8.265323325	4.61E-13	8.80E-12	19.24962363
								CXCR7	-0.926509654	8.6451234	-8.260331777	4.72E-13	9.01E-12	19.22471038
								KALRN	-0.5128629	5.773871419	-8.257146041	4.80E-13	9.15E-12	19.20881168
								REEP4	0.891597895	6.156150522	8.255651021	4.84E-13	9.21E-12	19.20135108
								IFIT3	0.747151308	6.6570081	8.255511591	4.84E-13	9.21E-12	19.2006553
								IFIT1	0.88785884	7.606502818	8.253491623	4.89E-13	9.29E-12	19.19057552
								CACNB2	-0.722439874	5.001898157	-8.252219828	4.92E-13	9.33E-12	19.18422943
								NEDD9	-0.670627301	4.841078235	-8.250844422	4.96E-13	9.39E-12	19.17736656
								RHBDF2	1.054623102	7.684773978	8.249437739	4.99E-13	9.44E-12	19.17034786
								IL1RN	1.455546574	6.480044519	8.248801377	5.01E-13	9.47E-12	19.16717279
								CRTAM	0.784370581	4.611917753	8.245591864	5.09E-13	9.61E-12	19.15115999
								SP140	0.529765467	5.407360491	8.242906568	5.16E-13	9.74E-12	19.13776356
								NT5C3L	-0.529295784	5.921698922	-8.241052354	5.21E-13	9.82E-12	19.12851376
								TMEM180	0.549630508	5.107432984	8.237796895	5.30E-13	9.97E-12	19.11227484
								MSRB3	-0.814441228	8.127274473	-8.237214713	5.31E-13	9.99E-12	19.10937093
								ARHGEF4	-0.520115729	4.811913266	-8.232811347	5.43E-13	1.02E-11	19.08740842

								TWF2	*0.533841518*	*6.696393017*	*8.231570366*	*5.47E-13*	*1.03E-11*	*19.08121926*
								LGALS9	*0.819652536*	*5.745998112*	*8.230475544*	*5.50E-13*	*1.03E-11*	*19.0757592*
								P2RX4	*0.861439065*	*8.552605471*	*8.227738257*	*5.57E-13*	*1.04E-11*	*19.06210854*
								KCNIP3	*-0.93678449*	*5.72469138*	*-8.224778707*	*5.66E-13*	*1.06E-11*	*19.04735052*
								BST2	*0.803975232*	*8.066803328*	*8.222085982*	*5.73E-13*	*1.07E-11*	*19.033924*
								SOCS2	*-0.877699917*	*7.272393684*	*-8.221258209*	*5.76E-13*	*1.07E-11*	*19.02979673*
								EFNB1	*0.565549782*	*7.517177418*	*8.22084332*	*5.77E-13*	*1.08E-11*	*19.02772812*
								PRDM1	*0.721119642*	*5.708489228*	*8.21932405*	*5.82E-13*	*1.08E-11*	*19.02015335*
								SNORD21	*-0.583234756*	*5.849174048*	*-8.218283506*	*5.85E-13*	*1.09E-11*	*19.01496557*
								TACC2	*-0.715867424*	*6.54184259*	*-8.218262783*	*5.85E-13*	*1.09E-11*	*19.01486225*
								TOM1	*0.505199051*	*8.197879631*	*8.218003866*	*5.85E-13*	*1.09E-11*	*19.0135714*
								LOC440104	*0.588900924*	*6.174764937*	*8.21618278*	*5.91E-13*	*1.10E-11*	*19.00449252*
								TMEM176B	*1.274725007*	*7.352666454*	*8.214588583*	*5.96E-13*	*1.10E-11*	*18.99654511*
								NKG7	*0.97780448*	*6.253326006*	*8.214455013*	*5.96E-13*	*1.10E-11*	*18.99587925*
								RHOB	*-0.991473108*	*7.984019798*	*-8.206947395*	*6.19E-13*	*1.15E-11*	*18.95845662*
								BEND5	*-0.669009025*	*5.800281697*	*-8.20598522*	*6.22E-13*	*1.15E-11*	*18.95366106*
								ATP6V0A1	*0.561122428*	*9.013516022*	*8.204836373*	*6.26E-13*	*1.16E-11*	*18.94793526*
								SNORD114-15	*-0.758431221*	*5.20863367*	*-8.203785341*	*6.29E-13*	*1.16E-11*	*18.94269712*
								RBPMS	*-0.876609624*	*6.567107478*	*-8.198828109*	*6.45E-13*	*1.19E-11*	*18.9179931*
								XLOC_011815	*-0.712377837*	*6.194211802*	*-8.198701924*	*6.46E-13*	*1.19E-11*	*18.9173643*
								CD3G	*0.962585526*	*5.742978476*	*8.19506214*	*6.58E-13*	*1.21E-11*	*18.89922774*
								SNORA63	*-0.646806655*	*6.60730146*	*-8.194212021*	*6.61E-13*	*1.21E-11*	*18.89499195*

								RGS5	-1.406007499	9.667042576	-8.192776952	6.65E-13	1.22E-11	18.88784181
								SPINT1	0.862663625	4.854887967	8.192006377	6.68E-13	1.22E-11	18.88400258
								RNF180	-0.559051184	6.435503448	-8.191774155	6.69E-13	1.22E-11	18.8828456
								3-Mar	-0.518292749	4.615513549	-8.19036218	6.74E-13	1.23E-11	18.87581096
								FDXR	0.542527338	7.882633178	8.189274971	6.77E-13	1.24E-11	18.87039452
								XLOC_012724	-0.614589027	4.835158936	-8.185219952	6.91E-13	1.26E-11	18.85019389
								RAB38	0.618674519	5.292238123	8.184972204	6.92E-13	1.26E-11	18.84895977
								LPAR6	0.584962773	7.013710341	8.178261795	7.16E-13	1.30E-11	18.8155358
								PODXL	0.834385804	8.519803648	8.175618055	7.26E-13	1.32E-11	18.80236916
								CLN6	0.710512751	5.526089087	8.166767357	7.59E-13	1.37E-11	18.75829651
								MIPOL1	-0.566055991	5.087360232	-8.165625546	7.63E-13	1.38E-11	18.75261153
								XLOC_l2_012081	0.507619498	6.236446189	8.165061416	7.66E-13	1.38E-11	18.74980284
								NSUN7	-0.659575809	4.777797068	-8.156746402	7.99E-13	1.44E-11	18.70840879
								PLA2G7	1.871742154	6.250542655	8.156323225	8.00E-13	1.44E-11	18.70630236
								AURKA	0.516409894	4.693410298	8.153077075	8.13E-13	1.46E-11	18.69014491
								RNASET2	0.699357341	6.251170044	8.151403175	8.20E-13	1.47E-11	18.68181375
								HLA-DQB2	1.172108324	6.986083746	8.151078885	8.22E-13	1.47E-11	18.68019977
								HAVCR2	1.222293474	5.783662519	8.144777103	8.48E-13	1.51E-11	18.64883876
								LILRB4	1.186339622	5.976662439	8.144431456	8.50E-13	1.52E-11	18.64711879
								DARC	-1.315817484	5.840979766	-8.143554545	8.54E-13	1.52E-11	18.64275527
								OSMR	-0.611073723	5.662957596	-8.141156952	8.64E-13	1.54E-11	18.63082534
								SPAG5	0.593759433	5.547400267	8.141093176	8.64E-13	1.54E-11	18.63050801

								CD3D	*1.092588807*	*6.551193469*	*8.139360276*	*8.72E-13*	*1.55E-11*	*18.62188594*
								SNORD71	*-0.72876703*	*6.2528338*	*-8.137945039*	*8.78E-13*	*1.56E-11*	*18.61484471*
								RPGRIP1	*0.577049848*	*4.52332057*	*8.136781053*	*8.83E-13*	*1.57E-11*	*18.60905372*
								SNORA57	*-0.659146388*	*7.478709006*	*-8.135607338*	*8.89E-13*	*1.58E-11*	*18.60321451*
								LPAR5	*0.771785555*	*4.765890953*	*8.134152416*	*8.95E-13*	*1.59E-11*	*18.59597655*
								CD83	*1.544483202*	*7.628642077*	*8.130920702*	*9.10E-13*	*1.61E-11*	*18.57990041*
								ASXL1	*-0.567385936*	*6.42704372*	*-8.127993058*	*9.24E-13*	*1.63E-11*	*18.56533806*
								GPX1	*0.817935867*	*10.52811368*	*8.126696969*	*9.30E-13*	*1.64E-11*	*18.55889157*
								AOAH	*0.955650046*	*5.274896102*	*8.125649482*	*9.35E-13*	*1.65E-11*	*18.55368173*
								TMSB15B	*-0.690315801*	*6.073185446*	*-8.122520711*	*9.49E-13*	*1.67E-11*	*18.5381212*
								SH2D3C	*0.876869585*	*7.366769438*	*8.116082276*	*9.81E-13*	*1.73E-11*	*18.50610461*
								HABP4	*-0.596654857*	*8.349630799*	*-8.107966406*	*1.02E-12*	*1.79E-11*	*18.46575452*
								ZAP70	*0.627302127*	*4.848444918*	*8.106309005*	*1.03E-12*	*1.81E-11*	*18.45751543*
								ASPM	*0.802675258*	*5.009245331*	*8.102572897*	*1.05E-12*	*1.84E-11*	*18.43894425*
								NDST1	*0.623015579*	*8.345011871*	*8.09844701*	*1.07E-12*	*1.88E-11*	*18.41843778*
								CIITA	*0.637088266*	*5.3909692*	*8.094910672*	*1.09E-12*	*1.91E-11*	*18.40086333*
								COL1A2	*0.783465823*	*10.72921964*	*8.09383274*	*1.10E-12*	*1.92E-11*	*18.39550669*
								MYBPH	*0.675627297*	*4.93882033*	*8.091854325*	*1.11E-12*	*1.93E-11*	*18.38567564*
								MX1	*0.806853128*	*8.255188489*	*8.089497634*	*1.12E-12*	*1.96E-11*	*18.37396558*
								CCL18	*2.996146159*	*8.819578567*	*8.08840947*	*1.13E-12*	*1.96E-11*	*18.3685589*
								GGA2	*0.528623992*	*6.713285083*	*8.088369277*	*1.13E-12*	*1.96E-11*	*18.3683592*
								C14orf132	*-0.784046438*	*6.412900749*	*-8.084467706*	*1.15E-12*	*2.00E-11*	*18.34897512*

								LOC100129781	-0.609112626	5.307732847	-8.083820688	1.15E-12	2.01E-11	18.34576075
								ZNF295	-0.573762604	6.355367133	-8.082099525	1.16E-12	2.02E-11	18.33721036
								OAS1	0.908081169	5.294820507	8.081038253	1.17E-12	2.03E-11	18.33193837
								PIF1	0.614818935	5.208098028	8.071128728	1.23E-12	2.13E-11	18.28271917
								TTYH3	0.83119686	6.535949766	8.066639218	1.26E-12	2.17E-11	18.26042488
								PCP4	-1.48124637	4.985127758	-8.064718379	1.27E-12	2.19E-11	18.2508871
								NUAK2	0.769490467	5.931961633	8.063302167	1.28E-12	2.20E-11	18.24385535
								C3	-1.441322075	6.884767434	-8.062662658	1.28E-12	2.21E-11	18.24068015
								JPH2	-0.67618524	5.559404798	-8.061028973	1.30E-12	2.22E-11	18.23256909
								CPVL	1.181455309	6.592532031	8.059973808	1.30E-12	2.23E-11	18.22733051
								TRPC1	-0.578582081	6.303077608	-8.059290302	1.31E-12	2.24E-11	18.22393719
								PLXNB1	-0.764450431	5.694758104	-8.055843906	1.33E-12	2.27E-11	18.20682827
								SYNPO	-0.591652608	6.915421038	-8.055307645	1.33E-12	2.28E-11	18.20416626
								LOC100128420	0.517375706	4.469185429	8.047186154	1.39E-12	2.37E-11	18.16385597
								KCNA3	0.546019135	4.652723033	8.046498488	1.39E-12	2.37E-11	18.16044323
								MFSD7	0.849732781	6.664986074	8.041834335	1.43E-12	2.43E-11	18.13729775
								CLDN23	0.894235094	5.581529307	8.035680569	1.47E-12	2.50E-11	18.10676488
								FAM150B	-1.388709844	6.266598733	-8.035084049	1.48E-12	2.50E-11	18.10380543
								TMEM100	-0.857611291	4.75516233	-8.033852873	1.49E-12	2.52E-11	18.09769751
								CAMK2G	-0.676032443	6.9353945	-8.029307272	1.52E-12	2.57E-11	18.0751484
								MYO1G	1.152296313	5.768992824	8.028299547	1.53E-12	2.58E-11	18.07014984
								IL7R	1.29196359	6.805692248	8.023502938	1.57E-12	2.64E-11	18.04635944

								ZNRF3	-0.51508887	4.850554162	-8.021887679	1.58E-12	2.66E-11	18.03834876
								ITPR3	0.59787042	6.149029777	8.01986177	1.59E-12	2.68E-11	18.02830203
								PBX4	0.858870557	5.389467737	8.016086457	1.63E-12	2.73E-11	18.00958135
								RUNX3	0.869028978	5.707097275	8.012760041	1.65E-12	2.77E-11	17.99308831
								SNAI3	0.690506628	4.723133398	8.010024795	1.68E-12	2.80E-11	17.9795276
								TRPV2	0.73293281	8.482289317	8.004083248	1.73E-12	2.88E-11	17.9500745
								PION	0.696858293	5.529756749	8.002990784	1.74E-12	2.90E-11	17.94465956
								OGFRL1	0.671434541	6.54257066	8.000210342	1.76E-12	2.93E-11	17.93087869
								NXF3	0.809098005	5.517584958	8.000123883	1.76E-12	2.93E-11	17.93045018
								TAGAP	0.764937497	5.017265694	7.998489334	1.78E-12	2.95E-11	17.92234931
								MFSD1	0.88520047	8.459009614	7.997149961	1.79E-12	2.97E-11	17.91571164
								TMEM206	0.665388602	5.355250393	7.99584237	1.80E-12	2.99E-11	17.90923171
								LOC283352	0.604852544	5.271180823	7.987089799	1.88E-12	3.11E-11	17.86586367
								DSEL	-0.506131917	5.839298146	-7.986354168	1.89E-12	3.12E-11	17.8622192
								POU2AF1	0.626255145	4.681441029	7.982636607	1.92E-12	3.17E-11	17.84380286
								SLC24A6	0.617330133	6.826235545	7.980946042	1.94E-12	3.19E-11	17.83542867
								TSKU	-0.661620806	6.432374282	-7.97542137	2.00E-12	3.27E-11	17.80806521
								LOC100653060	0.652671043	4.800705767	7.973543869	2.01E-12	3.30E-11	17.79876705
								SGCE	-0.734423904	7.731321938	-7.973493713	2.01E-12	3.30E-11	17.79851866
								LRRC8D	0.660126501	5.828745017	7.97331821	2.02E-12	3.30E-11	17.79764953
								SRMS	0.756259402	5.14050607	7.967158051	2.08E-12	3.39E-11	17.7671457
								ADPGK	0.716179519	6.456454349	7.964779172	2.11E-12	3.43E-11	17.75536749

								MS4A4E	*0.620335998*	*4.360678447*	*7.963306917*	*2.12E-12*	*3.45E-11*	*17.74807853*
								CD300C	*0.845398121*	*5.145813987*	*7.963284828*	*2.12E-12*	*3.45E-11*	*17.74796917*
								RGL4	*0.768761396*	*4.834566729*	*7.962808175*	*2.13E-12*	*3.46E-11*	*17.74560939*
								ZFPM2	*-0.554560236*	*5.581106029*	*-7.955337062*	*2.21E-12*	*3.58E-11*	*17.7086263*
								GIMAP6	*0.683355937*	*6.596063082*	*7.954194992*	*2.22E-12*	*3.60E-11*	*17.70297362*
								EFHD1	*-0.936634155*	*6.574183712*	*-7.95334288*	*2.23E-12*	*3.61E-11*	*17.69875621*
								DOCK4	*0.554467547*	*5.900891949*	*7.951152176*	*2.25E-12*	*3.64E-11*	*17.68791412*
								XLOC_007710	*0.6183652*	*4.513678981*	*7.950764052*	*2.26E-12*	*3.65E-11*	*17.68599332*
								CMIP	*0.605660043*	*5.903089881*	*7.943441385*	*2.34E-12*	*3.77E-11*	*17.64975808*
								C5orf20	*1.007279761*	*5.06916443*	*7.941060194*	*2.37E-12*	*3.81E-11*	*17.6379768*
								SYNDIG1	*-0.911138983*	*6.302742694*	*-7.938603249*	*2.40E-12*	*3.85E-11*	*17.62582161*
								ANXA8L2	*1.228602877*	*5.156434929*	*7.937634202*	*2.41E-12*	*3.86E-11*	*17.62102771*
								FOXL1	*-0.680587665*	*6.759342679*	*-7.934988622*	*2.45E-12*	*3.91E-11*	*17.60794068*
								CNTN1	*-0.565289816*	*4.094998529*	*-7.933847397*	*2.46E-12*	*3.94E-11*	*17.60229564*
								C1orf186	*0.780015931*	*5.21953972*	*7.933171494*	*2.47E-12*	*3.94E-11*	*17.5989524*
								SNORD114-23	*-0.662304759*	*4.928543987*	*-7.932995228*	*2.47E-12*	*3.95E-11*	*17.59808054*
								OPLAH	*0.644198741*	*5.798861627*	*7.929226022*	*2.52E-12*	*4.01E-11*	*17.57943816*
								SRPK3	*-0.684220811*	*5.037774283*	*-7.923808683*	*2.59E-12*	*4.11E-11*	*17.5526479*
								ZWINT	*0.720571385*	*5.257214947*	*7.922754279*	*2.60E-12*	*4.13E-11*	*17.54743409*
								CCNB2	*0.860355458*	*5.157821351*	*7.922678658*	*2.60E-12*	*4.13E-11*	*17.54706017*
								CDT1	*0.743167972*	*4.928138737*	*7.920947245*	*2.62E-12*	*4.16E-11*	*17.53849908*
								LOC100507505	*-0.601297556*	*4.426465411*	*-7.918814643*	*2.65E-12*	*4.20E-11*	*17.52795491*

								SNORD114-14	-0.556336447	4.704411185	-7.917482603	2.67E-12	4.22E-11	17.52136928
								SLC2A4	-0.584702883	4.213378117	-7.916780844	2.68E-12	4.24E-11	17.51789988
								KIAA0226L	0.801611884	5.699240726	7.915036728	2.70E-12	4.27E-11	17.50927754
								RAB23	-0.668454211	6.67860994	-7.913697296	2.72E-12	4.30E-11	17.50265614
								CHRDL1	-1.39643833	5.273284636	-7.909567137	2.78E-12	4.38E-11	17.48224067
								PCDH11Y	-0.584739455	4.276423874	-7.906212726	2.83E-12	4.45E-11	17.46566165
								PLEK2	0.98986472	5.333783494	7.899829121	2.92E-12	4.59E-11	17.43411573
								CBLN3	0.554632307	5.842660088	7.899561289	2.92E-12	4.59E-11	17.43279232
								DLG3	-0.654845643	6.611037714	-7.899479399	2.92E-12	4.59E-11	17.43238769
								CRISPLD2	-1.181808944	5.181762778	-7.889435223	3.08E-12	4.81E-11	17.38276549
								KCNA5	-1.219829561	6.420953616	-7.887997357	3.10E-12	4.84E-11	17.37566314
								GIMAP8	0.65346578	6.098252499	7.886196463	3.13E-12	4.88E-11	17.36676805
								MAP1B	-0.853487285	7.114767231	-7.883997094	3.16E-12	4.92E-11	17.35590547
								SNORD114-3	-0.794538096	5.612991912	-7.879042792	3.24E-12	5.03E-11	17.33143915
								TCEAL2	-1.095886996	7.191595189	-7.87751666	3.27E-12	5.06E-11	17.32390327
								CNDP2	0.52300923	7.303873884	7.8774691	3.27E-12	5.06E-11	17.32366843
								LOC645954	-0.796307353	5.846118577	-7.873612541	3.33E-12	5.15E-11	17.30462679
								SELM	-0.684080326	8.901960114	-7.870442844	3.38E-12	5.22E-11	17.28897824
								PNOC	1.006894254	4.750573786	7.870317243	3.39E-12	5.22E-11	17.28835818
								TGM2	-0.658830139	6.833369026	-7.86239332	3.52E-12	5.41E-11	17.2492454
								SNORD114-20	-0.64121592	4.687551329	-7.862237738	3.53E-12	5.41E-11	17.24847754
								RALA	0.630975979	6.155765802	7.860643193	3.55E-12	5.45E-11	17.24060803

								NCS1	*-0.649402238*	*5.45525824*	*-7.858885755*	*3.59E-12*	*5.50E-11*	*17.23193506*
								XLOC_002133	*0.664720568*	*4.93502546*	*7.857149683*	*3.62E-12*	*5.54E-11*	*17.22336801*
								TCEAL4	*-0.578960681*	*9.642037376*	*-7.855573338*	*3.65E-12*	*5.58E-11*	*17.21558958*
								OSCAR	*0.527184065*	*6.937616386*	*7.855145038*	*3.65E-12*	*5.58E-11*	*17.21347622*
								MS4A4A	*1.450804015*	*8.352484031*	*7.850066356*	*3.75E-12*	*5.71E-11*	*17.18841861*
								SERPINA3	*-1.14833732*	*6.69510251*	*-7.846796023*	*3.81E-12*	*5.80E-11*	*17.17228533*
								CCR7	*1.07584034*	*6.146414945*	*7.846748932*	*3.81E-12*	*5.80E-11*	*17.17205303*
								MAPK13	*1.03487831*	*5.573894802*	*7.845840817*	*3.83E-12*	*5.82E-11*	*17.1675734*
								ZBTB47	*-0.508868443*	*8.058741877*	*-7.842654578*	*3.89E-12*	*5.91E-11*	*17.15185709*
								SRF	*-0.500978534*	*5.194486531*	*-7.840494391*	*3.93E-12*	*5.97E-11*	*17.14120275*
								CNTN3	*-0.837110184*	*4.533732924*	*-7.83788358*	*3.98E-12*	*6.04E-11*	*17.12832686*
								ELN	*-1.022072572*	*6.625889948*	*-7.836700994*	*4.01E-12*	*6.07E-11*	*17.12249499*
								GALNT5	*0.64786236*	*5.019639909*	*7.833190593*	*4.08E-12*	*6.17E-11*	*17.10518491*
								DSC2	*0.784436873*	*5.247857902*	*7.832507978*	*4.09E-12*	*6.19E-11*	*17.10181911*
								ITGAX	*1.30188815*	*6.076748117*	*7.827285277*	*4.20E-12*	*6.33E-11*	*17.07606974*
								RAB42	*1.586522024*	*5.995916105*	*7.822437479*	*4.31E-12*	*6.47E-11*	*17.05217265*
								PLXNC1	*0.850773069*	*5.447476889*	*7.81958997*	*4.37E-12*	*6.55E-11*	*17.03813768*
								SAMD3	*0.50746092*	*4.488574089*	*7.819269671*	*4.37E-12*	*6.56E-11*	*17.03655906*
								PAR-SN	*-0.52491498*	*6.959290874*	*-7.818997841*	*4.38E-12*	*6.57E-11*	*17.03521933*
								AIM2	*0.792027372*	*5.152721133*	*7.815824372*	*4.45E-12*	*6.66E-11*	*17.01957959*
								MZB1	*0.795199817*	*4.805543462*	*7.812551851*	*4.52E-12*	*6.76E-11*	*17.00345339*
								HPCAL1	*0.509429568*	*6.54108386*	*7.811676205*	*4.54E-12*	*6.79E-11*	*16.99913871*

								HIST2H2AA4	*-0.706067191*	*6.029226093*	*-7.810576074*	*4.57E-12*	*6.82E-11*	*16.99371808*
								MT1X	*-1.028379103*	*8.364106223*	*-7.807158452*	*4.65E-12*	*6.94E-11*	*16.97687979*
								LGMN	*1.089168533*	*7.133477478*	*7.805480931*	*4.69E-12*	*6.99E-11*	*16.9686155*
								MYOM1	*-1.144740411*	*6.557475264*	*-7.805139725*	*4.70E-12*	*7.00E-11*	*16.96693461*
								KRBOX1	*-0.527962375*	*5.020066411*	*-7.799590484*	*4.83E-12*	*7.18E-11*	*16.93959989*
								CTSS	*1.075668242*	*5.582551117*	*7.799278979*	*4.84E-12*	*7.18E-11*	*16.93806561*
								SACS	*-0.627207915*	*6.651679612*	*-7.79927398*	*4.84E-12*	*7.18E-11*	*16.93804099*
								EVI2A	*0.918979404*	*6.993365496*	*7.798797933*	*4.85E-12*	*7.20E-11*	*16.93569631*
								BMP2K	*0.644444155*	*5.640531727*	*7.796541045*	*4.90E-12*	*7.27E-11*	*16.92458094*
								GM2A	*1.043775926*	*7.636926963*	*7.796175757*	*4.91E-12*	*7.28E-11*	*16.92278195*
								LAMP3	*0.925016687*	*4.816914184*	*7.795646325*	*4.92E-12*	*7.30E-11*	*16.9201746*
								CACNA2D4	*0.879121375*	*5.711420222*	*7.789929154*	*5.07E-12*	*7.50E-11*	*16.89202158*
								LIFR	*-0.672697103*	*5.614628278*	*-7.764210447*	*5.77E-12*	*8.46E-11*	*16.76544145*
								HK3	*1.030629132*	*5.217344616*	*7.763851119*	*5.78E-12*	*8.47E-11*	*16.76367372*
								PARM1	*-1.074855785*	*6.434694447*	*-7.762162308*	*5.82E-12*	*8.54E-11*	*16.75536579*
								NCKAP1L	*0.826703427*	*5.667343738*	*7.75984659*	*5.89E-12*	*8.63E-11*	*16.74397463*
								CXCL14	*-2.92648777*	*7.439607516*	*-7.757761132*	*5.95E-12*	*8.72E-11*	*16.73371689*
								OSBPL7	*0.530416036*	*7.353193537*	*7.752196033*	*6.12E-12*	*8.95E-11*	*16.7063474*
								LOC439949	*0.624712832*	*5.029267039*	*7.751069367*	*6.16E-12*	*9.00E-11*	*16.70080702*
								GMPR	*-0.57162324*	*5.617501544*	*-7.747134107*	*6.28E-12*	*9.16E-11*	*16.68145703*
								LOC100505702	*0.548479671*	*4.730878786*	*7.738904743*	*6.54E-12*	*9.53E-11*	*16.64100097*
								XLOC_l2_004840	*0.993549465*	*5.315532991*	*7.738194023*	*6.57E-12*	*9.56E-11*	*16.63750756*

								CTSK	*0.947545847*	*9.860760552*	*7.73638499*	*6.63E-12*	*9.64E-11*	*16.62861599*
								SFRP1	*-1.371157398*	*5.863216496*	*-7.732130747*	*6.77E-12*	*9.83E-11*	*16.60770813*
								PIM2	*0.781893144*	*5.939608622*	*7.730370298*	*6.83E-12*	*9.90E-11*	*16.59905714*
								TNFSF13B	*0.7747237*	*7.0601215*	*7.729166973*	*6.87E-12*	*9.96E-11*	*16.5931442*
								RORC	*0.539664034*	*4.424882758*	*7.726110282*	*6.98E-12*	*1.01E-10*	*16.57812524*
								ABI3	*0.594500056*	*6.413284419*	*7.723845426*	*7.06E-12*	*1.02E-10*	*16.56699795*
								LOC100653210	*1.727970254*	*6.529043614*	*7.723062833*	*7.08E-12*	*1.02E-10*	*16.56315326*
								IL27RA	*0.603084329*	*5.952031028*	*7.719289796*	*7.22E-12*	*1.04E-10*	*16.54461868*
								HSPA12A	*-0.575869909*	*6.604026244*	*-7.718940733*	*7.23E-12*	*1.04E-10*	*16.54290407*
								HCK	*0.966797303*	*6.201536105*	*7.718578476*	*7.24E-12*	*1.05E-10*	*16.54112468*
								LOC100129846	*-1.099798419*	*6.973966559*	*-7.716346597*	*7.33E-12*	*1.06E-10*	*16.53016225*
								BAG3	*-0.602171723*	*6.305890521*	*-7.715237161*	*7.37E-12*	*1.06E-10*	*16.5247133*
								C6orf192	*0.890460461*	*6.36656677*	*7.715070685*	*7.37E-12*	*1.06E-10*	*16.52389568*
								ARID5B	*-0.534860127*	*6.177009938*	*-7.712514723*	*7.47E-12*	*1.07E-10*	*16.51134304*
								STXBP2	*0.63643481*	*4.63401771*	*7.711892372*	*7.49E-12*	*1.08E-10*	*16.50828677*
								GKAP1	*-0.552029593*	*5.660392576*	*-7.711857935*	*7.49E-12*	*1.08E-10*	*16.50811765*
								LOC100289026	*0.547417552*	*4.614822743*	*7.709805554*	*7.57E-12*	*1.09E-10*	*16.4980392*
								COMMD9	*0.55303785*	*6.024743345*	*7.709160945*	*7.59E-12*	*1.09E-10*	*16.49487392*
								RNF130	*0.568273993*	*8.261857435*	*7.703682288*	*7.80E-12*	*1.12E-10*	*16.46797446*
								LOC100133190	*-0.525755892*	*5.00718198*	*-7.695005199*	*8.15E-12*	*1.16E-10*	*16.42538168*
								VNN1	*1.110200918*	*5.542702469*	*7.693554464*	*8.21E-12*	*1.17E-10*	*16.41826179*
								SGCB	*-0.591605611*	*7.086122215*	*-7.692985643*	*8.23E-12*	*1.17E-10*	*16.41547024*

								COX7A1	*-0.550974322*	*9.899060555*	*-7.691857774*	*8.28E-12*	*1.18E-10*	*16.40993527*
								GIMAP4	*0.701358731*	*7.212721958*	*7.689770269*	*8.37E-12*	*1.19E-10*	*16.39969151*
								RYR2	*-0.975398109*	*5.343906106*	*-7.688136887*	*8.43E-12*	*1.20E-10*	*16.39167673*
								FUCA1	*1.281815514*	*7.972903226*	*7.68694011*	*8.48E-12*	*1.21E-10*	*16.38580461*
								RCSD1	*0.645639389*	*6.328777744*	*7.677106482*	*8.91E-12*	*1.26E-10*	*16.33756417*
								NTN4	*-0.611346596*	*6.734527178*	*-7.667564683*	*9.35E-12*	*1.32E-10*	*16.29077142*
								IGJ	*1.562658476*	*6.567942168*	*7.667306284*	*9.36E-12*	*1.32E-10*	*16.28950446*
								SNORA15	*-0.759664805*	*6.190937401*	*-7.665811716*	*9.43E-12*	*1.33E-10*	*16.28217664*
								RASSF4	*0.922747381*	*6.304990628*	*7.664761468*	*9.48E-12*	*1.34E-10*	*16.27702754*
								PLSCR4	*-0.559278739*	*6.99202031*	*-7.663981987*	*9.52E-12*	*1.34E-10*	*16.27320607*
								FLJ26332	*-0.585601152*	*4.96090688*	*-7.662598393*	*9.58E-12*	*1.35E-10*	*16.26642315*
								XLOC_002872	*-0.526332005*	*5.964938615*	*-7.658392155*	*9.78E-12*	*1.37E-10*	*16.24580456*
								SLC29A3	*1.081189104*	*5.895642193*	*7.65708884*	*9.85E-12*	*1.38E-10*	*16.23941646*
								TBX21	*0.654933924*	*4.92181014*	*7.656648773*	*9.87E-12*	*1.38E-10*	*16.23725958*
								XLOC_004144	*-0.513655644*	*5.218354071*	*-7.653240515*	*1.00E-11*	*1.41E-10*	*16.22055594*
								B2M	*0.556264931*	*11.00735397*	*7.652985907*	*1.01E-11*	*1.41E-10*	*16.21930821*
								BLM	*0.523969693*	*4.701504226*	*7.650874621*	*1.02E-11*	*1.42E-10*	*16.20896206*
								TDP1	*0.52362723*	*6.098953172*	*7.650528928*	*1.02E-11*	*1.42E-10*	*16.20726811*
								IFIT2	*0.69050054*	*6.202638789*	*7.648541411*	*1.03E-11*	*1.44E-10*	*16.19752931*
								CD96	*0.763990072*	*5.189393884*	*7.643425676*	*1.05E-11*	*1.47E-10*	*16.17246554*
								GAL3ST4	*0.62676514*	*4.919121739*	*7.642267645*	*1.06E-11*	*1.48E-10*	*16.16679258*
								PCOLCE2	*-0.954001167*	*8.571820162*	*-7.627125751*	*1.14E-11*	*1.59E-10*	*16.09263747*

								MYBPC3	*0.641322137*	*5.157751683*	*7.624867337*	*1.16E-11*	*1.61E-10*	*16.08158073*
								NAP1L3	*-0.602211456*	*5.789581252*	*-7.622377463*	*1.17E-11*	*1.63E-10*	*16.06939187*
								TLR4	*0.598823164*	*6.88938857*	*7.60850635*	*1.25E-11*	*1.73E-10*	*16.00150796*
								NUSAP1	*0.774367171*	*5.094612512*	*7.608010549*	*1.26E-11*	*1.74E-10*	*15.9990822*
								ALDH1B1	*-0.849957527*	*7.426058074*	*-7.605383247*	*1.27E-11*	*1.76E-10*	*15.98622857*
								CXCL12	*0.55404244*	*6.833510496*	*7.600212549*	*1.31E-11*	*1.80E-10*	*15.96093545*
								TREM1	*1.008019559*	*5.247049244*	*7.600080121*	*1.31E-11*	*1.80E-10*	*15.96028773*
								ARHGEF26	*-0.747370793*	*5.879626932*	*-7.599489895*	*1.31E-11*	*1.81E-10*	*15.95740089*
								MT1L	*-0.818897149*	*9.856987131*	*-7.594911647*	*1.34E-11*	*1.84E-10*	*15.93501046*
								PLD3	*1.01164158*	*7.933345477*	*7.594709954*	*1.34E-11*	*1.84E-10*	*15.93402414*
								PP12719	*0.577397104*	*5.945768509*	*7.590515836*	*1.37E-11*	*1.88E-10*	*15.91351583*
								RAB9B	*-0.711634061*	*5.449137083*	*-7.589591595*	*1.38E-11*	*1.88E-10*	*15.90899693*
								AFAP1L1	*0.71064383*	*6.374361983*	*7.582087683*	*1.43E-11*	*1.95E-10*	*15.87231374*
								MT1B	*-0.818444567*	*9.853520765*	*-7.57878908*	*1.46E-11*	*1.98E-10*	*15.85619163*
								ATF7IP2	*0.504537722*	*6.034471414*	*7.576336788*	*1.47E-11*	*2.00E-10*	*15.84420721*
								HCAR3	*1.357164577*	*5.805343021*	*7.572677451*	*1.50E-11*	*2.03E-10*	*15.82632598*
								AASS	*-0.620130626*	*5.831817926*	*-7.572244378*	*1.50E-11*	*2.04E-10*	*15.82420994*
								SNORD114-9	*-0.705104093*	*5.176470526*	*-7.569349915*	*1.53E-11*	*2.06E-10*	*15.81006821*
								IGFLR1	*0.981889604*	*7.79292196*	*7.567209591*	*1.54E-11*	*2.08E-10*	*15.79961204*
								ARL4C	*1.117573219*	*7.908106405*	*7.566325695*	*1.55E-11*	*2.09E-10*	*15.79529417*
								LOC100507233	*-0.585276491*	*4.804300406*	*-7.563589522*	*1.57E-11*	*2.12E-10*	*15.78192876*
								ISG15	*0.534249659*	*9.006097699*	*7.562587046*	*1.58E-11*	*2.13E-10*	*15.7770323*

								MMP7	2.46896161	6.010118146	7.560700264	1.59E-11	2.14E-10	15.76781707
								ROM1	-0.568632899	7.207194629	-7.56018049	1.60E-11	2.15E-10	15.76527856
								PRKCH	0.588623062	6.965162863	7.557094501	1.62E-11	2.18E-10	15.750208
								IGF2BP3	0.676071004	5.598078341	7.553262495	1.65E-11	2.22E-10	15.7314967
								STK17B	0.527909624	5.971028421	7.550570647	1.67E-11	2.25E-10	15.7183543
								MT1A	-0.772185905	10.01330117	-7.549379182	1.68E-11	2.26E-10	15.71253765
								C10orf35	-0.544371487	5.88615468	-7.548947283	1.69E-11	2.26E-10	15.71042922
								TMEM35	-1.036603734	5.525340689	-7.54829976	1.69E-11	2.27E-10	15.70726821
								SORBS1	-1.216633122	7.4674508	-7.546408998	1.71E-11	2.29E-10	15.69803856
								PLA2G4C	0.606761346	7.478727555	7.543213131	1.74E-11	2.32E-10	15.68243963
								TRIM14	0.655847324	4.962540956	7.543158226	1.74E-11	2.32E-10	15.68217165
								FILIP1	-0.812206528	5.712154248	-7.53993837	1.77E-11	2.36E-10	15.66645759
								NOD2	0.844271373	5.845382355	7.539931867	1.77E-11	2.36E-10	15.66642585
								IMMP2L	-0.514562847	6.458528973	-7.528382408	1.87E-11	2.49E-10	15.61007629
								HIST1H1E	-0.708233828	7.767887907	-7.52672142	1.89E-11	2.51E-10	15.60197443
								ADAMTS1	-1.078799277	7.49826482	-7.524804149	1.90E-11	2.53E-10	15.59262313
								FAM20A	0.908011146	6.333274512	7.518971605	1.96E-11	2.59E-10	15.56417974
								THEMIS	0.584853982	4.540541819	7.518677261	1.96E-11	2.60E-10	15.56274449
								CATSPERB	-0.578825189	4.222360216	-7.517169539	1.98E-11	2.62E-10	15.55539296
								GATA6	-0.540989373	6.934496081	-7.511390942	2.03E-11	2.69E-10	15.52722096
								XLOC_003228	0.719566002	4.554888093	7.503699166	2.11E-11	2.78E-10	15.48973161
								ATP6V1B2	0.667523723	7.695079818	7.503205874	2.12E-11	2.79E-10	15.48732771

								TTLL7	-0.830399475	5.905912857	-7.501851274	2.13E-11	2.80E-10	15.48072675
								C1QB	1.341990738	9.655175769	7.488092448	2.28E-11	2.99E-10	15.4136999
								LGALSL	-0.511575552	7.701861169	-7.484286889	2.33E-11	3.04E-10	15.39516733
								VOPP1	0.540440752	7.794679555	7.482962372	2.34E-11	3.06E-10	15.38871776
								ADRA2C	-1.554941138	7.778398073	-7.481048677	2.36E-11	3.08E-10	15.37939985
								LOC728392	0.500166642	7.396146795	7.475593717	2.43E-11	3.16E-10	15.35284318
								TREML1	0.553965831	4.368329156	7.475536746	2.43E-11	3.16E-10	15.35256586
								TYMS	0.66786915	6.397950508	7.470207698	2.50E-11	3.24E-10	15.32662778
								CENPM	0.670920682	5.033784998	7.470086632	2.50E-11	3.24E-10	15.32603858
								GPRIN3	0.572903405	4.731150682	7.469601132	2.50E-11	3.25E-10	15.32367579
								CRNDE	-0.520199783	6.757964076	-7.466959972	2.54E-11	3.29E-10	15.31082283
								IRF5	1.372282102	7.175730056	7.46191073	2.60E-11	3.36E-10	15.28625491
								GRB14	-0.798323253	5.508593014	-7.460969668	2.61E-11	3.38E-10	15.28167657
								SLC22A18AS	0.924506565	5.201610933	7.460078814	2.62E-11	3.39E-10	15.27734266
								CAV2	-0.500912273	6.039347725	-7.456245556	2.67E-11	3.45E-10	15.258696
								ATP9A	-0.566575419	6.70383482	-7.453873129	2.71E-11	3.48E-10	15.2471569
								XLOC_002035	-0.61651496	4.679670834	-7.451854409	2.73E-11	3.51E-10	15.23733904
								LOC100506299	-0.531487508	4.671510029	-7.447893838	2.79E-11	3.57E-10	15.21807949
								ACBD7	-0.562963451	5.84308164	-7.445274445	2.82E-11	3.61E-10	15.20534354
								C1orf162	0.875127871	6.953060777	7.442037025	2.87E-11	3.67E-10	15.18960449
								XLOC_003123	-0.595960492	5.044180729	-7.441511901	2.88E-11	3.67E-10	15.18705174
								XLOC_l2_006821	1.348930054	6.937057781	7.435965634	2.96E-11	3.77E-10	15.16009336

								MYLK	-0.712148001	7.938797843	-7.434896974	2.97E-11	3.78E-10	15.1548997
								AOC4	-0.866991808	5.251857384	-7.433236425	3.00E-11	3.81E-10	15.14682991
								ARAP3	0.629243725	7.534970095	7.432360586	3.01E-11	3.83E-10	15.1425738
								CTNNAL1	-0.624110621	7.628505279	-7.430504771	3.04E-11	3.86E-10	15.13355603
								ACCN4	0.926565372	7.067804513	7.426483917	3.10E-11	3.94E-10	15.11402026
								GABARAPL1	-0.513725116	6.466731075	-7.425850987	3.11E-11	3.95E-10	15.11094539
								FAM149A	-0.57663999	5.504670501	-7.42537363	3.12E-11	3.96E-10	15.10862637
								SNORD114-21	-0.705571366	5.294502364	-7.424966869	3.12E-11	3.96E-10	15.10665035
								PDE6G	1.280978106	5.892685612	7.424443527	3.13E-11	3.97E-10	15.10410803
								SCXA	-0.702314826	5.284736789	-7.421986852	3.17E-11	4.01E-10	15.09217457
								MME	0.64534461	4.929338588	7.421090297	3.18E-11	4.03E-10	15.08781979
								KCNN4	1.088719517	6.84956174	7.418148997	3.23E-11	4.08E-10	15.07353433
								SEPP1	0.878831806	9.212318744	7.411947563	3.33E-11	4.21E-10	15.04342054
								RASL12	-1.09943307	6.178034503	-7.411703705	3.33E-11	4.21E-10	15.04223654
								NETO2	0.608915323	5.115975535	7.407899728	3.40E-11	4.28E-10	15.02376863
								SUSD1	0.81273506	6.58187608	7.407302365	3.41E-11	4.29E-10	15.02086876
								CXorf65	0.689653752	5.08297293	7.404852746	3.45E-11	4.34E-10	15.00897794
								NLRP3	0.745688376	5.822604626	7.403644682	3.47E-11	4.37E-10	15.00311426
								LILRA6	0.888500297	6.231369234	7.402049592	3.50E-11	4.40E-10	14.99537249
								CXCL16	0.908808445	6.790696245	7.401523849	3.51E-11	4.41E-10	14.99282091
								TREM2	1.501119236	6.564479991	7.399030215	3.55E-11	4.46E-10	14.98071934
								SNORA3	-0.552020574	5.446014674	-7.393616384	3.65E-11	4.57E-10	14.95445039

								SPARCL1	-1.032591554	10.00796009	-7.376206116	3.97E-11	4.97E-10	14.87001255
								SLC25A23	-0.513718034	5.78623274	-7.375702391	3.98E-11	4.98E-10	14.86757046
								PPT1	0.536775456	5.989129767	7.374223457	4.01E-11	5.02E-10	14.86040078
								SNCA	0.769290719	6.206764279	7.372081702	4.06E-11	5.07E-10	14.85001861
								SEMA3D	-0.982097203	5.253824832	-7.367278326	4.15E-11	5.18E-10	14.82673763
								B3GNT8	0.549658739	6.332236119	7.36399856	4.22E-11	5.26E-10	14.81084398
								GADD45B	-0.896912922	9.768168471	-7.361293612	4.28E-11	5.32E-10	14.79773754
								C7orf58	-0.629001928	5.571652633	-7.358848922	4.33E-11	5.37E-10	14.78589343
								NFIA	-0.590372228	6.263316053	-7.356871004	4.37E-11	5.42E-10	14.77631164
								LOXL2	0.845271262	7.361910971	7.353588351	4.44E-11	5.51E-10	14.760411
								VAC14	0.550111283	5.086088436	7.35076642	4.51E-11	5.58E-10	14.74674379
								GPR137B	0.889669122	6.45720913	7.342669048	4.69E-11	5.78E-10	14.7075356
								CD300LF	0.984914283	4.969822675	7.341121564	4.73E-11	5.82E-10	14.70004409
								HPR	-1.370035584	5.830225403	-7.339913935	4.75E-11	5.85E-10	14.69419819
								LOC401317	-0.692323491	4.646091136	-7.334049199	4.89E-11	6.01E-10	14.66581243
								LY96	0.840787794	7.884671268	7.323704865	5.15E-11	6.31E-10	14.61576248
								GRB2	0.541422072	7.082912801	7.317532657	5.31E-11	6.49E-10	14.58590953
								ITM2C	-0.72530018	6.592891223	-7.317034638	5.32E-11	6.50E-10	14.58350112
								ERRFI1	-0.715371255	8.654875569	-7.316599325	5.33E-11	6.51E-10	14.581396
								FHOD3	-0.651090312	6.790996561	-7.310870085	5.49E-11	6.68E-10	14.55369379
								PMAIP1	0.798045553	5.482359043	7.310378082	5.50E-11	6.69E-10	14.55131517
								PLTP	1.101709509	9.144636312	7.310119029	5.51E-11	6.69E-10	14.55006277

								BTC	-1.020690285	5.639077506	-7.302983145	5.70E-11	6.91E-10	14.51556988
								CKS2	0.64352805	6.537501821	7.299778712	5.79E-11	7.01E-10	14.50008402
								NFE2L3	0.596872404	5.864870411	7.29762185	5.86E-11	7.08E-10	14.48966191
								PDE8B	-1.05683875	6.509826581	-7.296348427	5.89E-11	7.12E-10	14.4835091
								RASSF3	-0.678236631	5.424182097	-7.295902777	5.91E-11	7.13E-10	14.48135593
								FBP1	1.624203373	6.912325275	7.293415398	5.98E-11	7.21E-10	14.46933887
								TES	-0.770978453	6.258614106	-7.292408493	6.01E-11	7.24E-10	14.46447466
								XLOC_005815	-0.773706962	4.630223799	-7.284262047	6.25E-11	7.51E-10	14.42512833
								QPRT	0.560283263	5.402168934	7.280307835	6.38E-11	7.64E-10	14.40603505
								SMAP2	0.597945301	6.374492512	7.280223858	6.38E-11	7.64E-10	14.40562959
								C10orf105	0.726038659	4.730738457	7.280054961	6.39E-11	7.64E-10	14.40481413
								PCOLCE	0.653345988	8.172262242	7.279354794	6.41E-11	7.67E-10	14.4014337
								APOE	1.931924327	10.72202268	7.267525856	6.79E-11	8.10E-10	14.34433889
								XLOC_011052	-0.518851685	4.501439686	-7.26715691	6.80E-11	8.11E-10	14.34255858
								SLC2A6	0.668744038	6.124229058	7.263629426	6.92E-11	8.25E-10	14.32553852
								XLOC_001215	0.817739335	4.589574213	7.262266741	6.97E-11	8.30E-10	14.3189643
								TCEA3	-0.562505199	6.991136302	-7.260016785	7.05E-11	8.38E-10	14.30811036
								HP	-1.802516288	6.50657385	-7.258663508	7.09E-11	8.43E-10	14.30158257
								PDGFD	-0.651082997	5.540247971	-7.251998693	7.33E-11	8.68E-10	14.26943936
								XLOC_l2_013301	0.978648858	4.741240279	7.25094769	7.37E-11	8.72E-10	14.26437144
								ARHGAP24	0.508839255	4.778679821	7.238887842	7.82E-11	9.22E-10	14.20623609
								SNORA68	-0.562601066	6.167080606	-7.236569989	7.91E-11	9.31E-10	14.19506633

								TSPAN2	*-0.703158952*	*5.241898879*	*-7.235031901*	*7.97E-11*	*9.37E-10*	*14.1876549*
								SIGLEC15	*0.880747504*	*6.364323509*	*7.234786689*	*7.98E-11*	*9.38E-10*	*14.18647338*
								PALLD	*-0.524002855*	*7.350266147*	*-7.23427893*	*8.00E-11*	*9.40E-10*	*14.18402683*
								THSD4	*-0.547708438*	*5.015390306*	*-7.232579703*	*8.06E-11*	*9.47E-10*	*14.17583983*
								MLF1IP	*0.586395074*	*5.121819715*	*7.229719921*	*8.18E-11*	*9.59E-10*	*14.1620626*
								AQP9	*1.63487498*	*6.288592703*	*7.221243721*	*8.53E-11*	*9.97E-10*	*14.12123832*
								LOC283335	*-0.513706006*	*5.201365265*	*-7.220216251*	*8.57E-11*	*1.00E-09*	*14.11629074*
								TNS1	*-0.732654668*	*8.176774585*	*-7.216739707*	*8.72E-11*	*1.02E-09*	*14.09955185*
								IGFBPL1	*-0.726418918*	*5.558479488*	*-7.212773491*	*8.89E-11*	*1.03E-09*	*14.08045851*
								C9orf150	*-0.566524906*	*5.603139015*	*-7.212571091*	*8.90E-11*	*1.04E-09*	*14.07948425*
								C7orf41	*-0.828351404*	*8.087308932*	*-7.211786467*	*8.93E-11*	*1.04E-09*	*14.07570752*
								SLC19A2	*-0.791477509*	*6.365711081*	*-7.209761027*	*9.02E-11*	*1.05E-09*	*14.06595884*
								HEXDC	*0.530278108*	*8.07519503*	*7.208087997*	*9.10E-11*	*1.06E-09*	*14.05790704*
								SLA	*1.104612347*	*8.079089778*	*7.206906264*	*9.15E-11*	*1.06E-09*	*14.05222008*
								SNORA11C	*-0.641934762*	*5.849271704*	*-7.204582699*	*9.25E-11*	*1.07E-09*	*14.04103906*
								SNORD114-12	*-0.578328288*	*5.063391511*	*-7.198832824*	*9.52E-11*	*1.10E-09*	*14.01337574*
								SLC43A2	*0.68810025*	*5.869206236*	*7.19802511*	*9.56E-11*	*1.10E-09*	*14.00949032*
								WEE1	*-0.628841117*	*5.810643829*	*-7.195449904*	*9.68E-11*	*1.12E-09*	*13.99710354*
								TLR2	*1.001274129*	*7.469422121*	*7.194780727*	*9.71E-11*	*1.12E-09*	*13.99388503*
								IL1B	*1.446722649*	*7.011350812*	*7.191126256*	*9.88E-11*	*1.14E-09*	*13.97631002*
								SNTA1	*-0.634282158*	*6.004264171*	*-7.186875281*	*1.01E-10*	*1.16E-09*	*13.95587006*
								CYTIP	*0.91588937*	*7.047533316*	*7.186052429*	*1.01E-10*	*1.16E-09*	*13.95191401*

								MMP1	*1.90647174*	*5.967470426*	*7.185537228*	*1.02E-10*	*1.17E-09*	*13.94943714*
								KHDRBS3	*-0.514782546*	*6.057502918*	*-7.182376414*	*1.03E-10*	*1.18E-09*	*13.93424255*
								SCARF1	*0.595267793*	*6.246225244*	*7.182097619*	*1.03E-10*	*1.18E-09*	*13.93290244*
								LOC100507254	*0.542878896*	*4.462696182*	*7.18075994*	*1.04E-10*	*1.19E-09*	*13.92647273*
								ASS1	*-0.510679624*	*6.600310404*	*-7.173070204*	*1.08E-10*	*1.23E-09*	*13.88951891*
								TPM1	*-0.809798498*	*9.206687162*	*-7.164629698*	*1.13E-10*	*1.28E-09*	*13.84897244*
								DAAM2	*-0.629201731*	*6.22386446*	*-7.160521986*	*1.15E-10*	*1.30E-09*	*13.82924561*
								RHOG	*0.534536747*	*6.463654358*	*7.158955251*	*1.16E-10*	*1.31E-09*	*13.82172255*
								TUBB3	*0.890768363*	*6.277705327*	*7.158372317*	*1.16E-10*	*1.32E-09*	*13.81892359*
								BIRC5	*0.955108829*	*5.805795066*	*7.154812978*	*1.18E-10*	*1.34E-09*	*13.80183507*
								NAGK	*0.606529755*	*7.527983751*	*7.153545769*	*1.19E-10*	*1.34E-09*	*13.79575184*
								EPB49	*-0.685460997*	*7.549023458*	*-7.153161112*	*1.19E-10*	*1.35E-09*	*13.79390537*
								KCNJ5	*1.125338093*	*4.997804547*	*7.151077898*	*1.20E-10*	*1.36E-09*	*13.78390589*
								CD180	*0.797222137*	*4.571527202*	*7.150158747*	*1.21E-10*	*1.36E-09*	*13.77949425*
								PVRL4	*0.987433303*	*5.033655387*	*7.149372753*	*1.21E-10*	*1.37E-09*	*13.77572188*
								HJURP	*0.730291446*	*4.93953553*	*7.144819245*	*1.24E-10*	*1.40E-09*	*13.7538701*
								TRIM2	*-0.508746769*	*5.598714888*	*-7.139169651*	*1.27E-10*	*1.43E-09*	*13.72676487*
								C21orf7	*-0.597646512*	*5.029711216*	*-7.133362753*	*1.31E-10*	*1.47E-09*	*13.69891251*
								ADORA2B	*0.807183136*	*4.976480887*	*7.132297205*	*1.32E-10*	*1.48E-09*	*13.69380252*
								FCN1	*1.116782981*	*6.990740484*	*7.12720862*	*1.35E-10*	*1.52E-09*	*13.66940306*
								LOC100653004	*0.521074059*	*6.134391206*	*7.123584964*	*1.38E-10*	*1.54E-09*	*13.65203146*
								GZMA	*1.103520213*	*6.703331314*	*7.11876669*	*1.41E-10*	*1.57E-09*	*13.62893759*

								HRC	*-0.781630293*	*5.328871767*	*-7.110262856*	*1.47E-10*	*1.64E-09*	*13.58819194*
								FTL	*0.945113466*	*10.52973835*	*7.108203728*	*1.48E-10*	*1.65E-09*	*13.57832825*
								CTSH	*0.969677447*	*8.734905725*	*7.099413414*	*1.55E-10*	*1.72E-09*	*13.53623167*
								PIK3AP1	*0.706541316*	*4.675678395*	*7.09649357*	*1.57E-10*	*1.75E-09*	*13.52225257*
								TM7SF4	*1.305408286*	*5.140562016*	*7.095078464*	*1.58E-10*	*1.76E-09*	*13.5154783*
								GIMAP7	*0.697293876*	*7.366111059*	*7.092592816*	*1.60E-10*	*1.78E-09*	*13.50358034*
								AKAP1	*-0.537660806*	*6.82522149*	*-7.090416172*	*1.62E-10*	*1.79E-09*	*13.49316267*
								ST14	*0.70160072*	*6.405824714*	*7.089214948*	*1.63E-10*	*1.80E-09*	*13.48741393*
								RHOH	*0.697286755*	*5.417011885*	*7.083257208*	*1.68E-10*	*1.85E-09*	*13.45890679*
								NFKBID	*0.660000097*	*5.066976949*	*7.080819814*	*1.70E-10*	*1.87E-09*	*13.44724651*
								FAM180B	*-1.271366827*	*6.170991461*	*-7.075503403*	*1.74E-10*	*1.92E-09*	*13.42181808*
								IFI44L	*0.797312583*	*6.825708953*	*7.074582821*	*1.75E-10*	*1.92E-09*	*13.41741561*
								ETS2	*-0.53774211*	*6.677334136*	*-7.073936383*	*1.75E-10*	*1.93E-09*	*13.41432428*
								CD28	*0.652995726*	*5.167130826*	*7.068385045*	*1.80E-10*	*1.98E-09*	*13.38778131*
								SNORD114-26	*-0.531453081*	*4.746801631*	*-7.065080152*	*1.83E-10*	*2.01E-09*	*13.37198285*
								GPD1L	*-0.579528196*	*6.741535564*	*-7.054606621*	*1.93E-10*	*2.10E-09*	*13.32193298*
								XLOC_007433	*-0.693573658*	*6.340380208*	*-7.049560919*	*1.97E-10*	*2.15E-09*	*13.29783034*
								PHGDH	*-0.968688443*	*6.591898216*	*-7.049261794*	*1.98E-10*	*2.15E-09*	*13.29640165*
								CPNE5	*0.86252363*	*6.628281465*	*7.041849898*	*2.05E-10*	*2.23E-09*	*13.2610075*
								LOC389493	*-0.55463567*	*5.21320029*	*-7.035199001*	*2.12E-10*	*2.30E-09*	*13.22925848*
								CALD1	*-0.65876291*	*10.31102327*	*-7.03262678*	*2.14E-10*	*2.32E-09*	*13.21698243*
								LAMA2	*-0.71819455*	*6.360448745*	*-7.031882422*	*2.15E-10*	*2.33E-09*	*13.21343025*

								SNORA34	-0.743323579	8.370312985	-7.031502705	2.16E-10	2.33E-09	13.21161823
								WAS	0.607994782	5.45824373	7.025666291	2.22E-10	2.40E-09	13.18377112
								KLHL6	1.096293045	5.912079188	7.019200463	2.29E-10	2.47E-09	13.15293044
								ACOT13	0.519615571	7.542695573	7.014833637	2.34E-10	2.52E-09	13.13210725
								SCRG1	-1.348766245	7.849986023	-7.011140645	2.38E-10	2.56E-09	13.11450082
								HBA2	1.890065193	9.797845028	7.008783527	2.41E-10	2.59E-09	13.10326493
								SMTN	-1.103893906	7.765521069	-7.008452037	2.41E-10	2.59E-09	13.10168489
								DACT3	-1.134768943	8.671530241	-6.996547088	2.56E-10	2.74E-09	13.04495793
								IL10RB	0.534779186	6.319000984	6.996155391	2.56E-10	2.74E-09	13.04309208
								TNNT3	-0.795973694	4.939036925	-6.995291729	2.57E-10	2.75E-09	13.03897816
								SNORD90	-0.584903505	5.421650148	-6.992475433	2.61E-10	2.79E-09	13.02556442
								ST6GAL2	-0.822528216	5.623242147	-6.991260743	2.62E-10	2.80E-09	13.01977957
								PAQR5	1.159310181	5.217767502	6.989946128	2.64E-10	2.82E-09	13.01351924
								TMEM200A	0.687343599	6.387076504	6.98090168	2.76E-10	2.93E-09	12.97046007
								SYNPO2	-0.926083149	6.799143369	-6.976804557	2.81E-10	2.99E-09	12.95096091
								TMSB15A	-0.641342263	4.668235439	-6.97663598	2.82E-10	2.99E-09	12.9501587
								SNORD113-9	-0.56532736	4.721585034	-6.976296089	2.82E-10	2.99E-09	12.94854128
								KCNAB2	0.643038498	4.802847521	6.973448785	2.86E-10	3.03E-09	12.93499307
								PPFIBP2	0.582567666	5.591748015	6.972053889	2.88E-10	3.05E-09	12.92835652
								MYO7A	0.776640198	5.683337644	6.971934278	2.88E-10	3.05E-09	12.92778746
								LILRA3	0.777902313	5.114428407	6.971587326	2.89E-10	3.06E-09	12.92613684
								F11R	0.642870752	6.937191052	6.971064231	2.89E-10	3.06E-09	12.92364827

								COL15A1	*0.683500967*	*7.880751029*	*6.967893708*	*2.94E-10*	*3.11E-09*	*12.9085663*
								DDR2	*-0.516209757*	*6.459816972*	*-6.966957082*	*2.95E-10*	*3.12E-09*	*12.90411131*
								ACTN1	*-0.521268392*	*9.118660647*	*-6.965002742*	*2.98E-10*	*3.14E-09*	*12.89481632*
								DPP7	*0.581186317*	*7.801733229*	*6.955925051*	*3.11E-10*	*3.27E-09*	*12.85165449*
								XLOC_002623	*0.675054434*	*4.717741567*	*6.952162527*	*3.17E-10*	*3.33E-09*	*12.83377073*
								KIAA1199	*0.960906193*	*5.130166448*	*6.951573813*	*3.18E-10*	*3.33E-09*	*12.83097281*
								ATP1A2	*-0.561541086*	*4.471916121*	*-6.948004011*	*3.24E-10*	*3.38E-09*	*12.81400885*
								DBN1	*-0.557114967*	*8.023000032*	*-6.947068131*	*3.25E-10*	*3.40E-09*	*12.809562*
								MC1R	*0.547312738*	*6.479326414*	*6.946436294*	*3.26E-10*	*3.41E-09*	*12.80655994*
								MMP12	*2.315056771*	*5.945250486*	*6.940355221*	*3.36E-10*	*3.50E-09*	*12.77767187*
								FAM96A	*0.759254665*	*7.513090557*	*6.940045887*	*3.36E-10*	*3.51E-09*	*12.77620263*
								LOC283663	*0.508071208*	*5.977298452*	*6.937565584*	*3.40E-10*	*3.55E-09*	*12.76442281*
								VMO1	*1.275671445*	*7.208430199*	*6.937410325*	*3.41E-10*	*3.55E-09*	*12.76368549*
								CORO2A	*0.693480598*	*5.348053863*	*6.935009048*	*3.45E-10*	*3.58E-09*	*12.75228255*
								LOC100652766	*-0.500836281*	*6.626756549*	*-6.932678024*	*3.49E-10*	*3.62E-09*	*12.74121459*
								VSIG4	*1.044423364*	*9.130489719*	*6.927845627*	*3.57E-10*	*3.70E-09*	*12.71827418*
								SNORA80B	*-0.5426275*	*5.684312602*	*-6.927213139*	*3.58E-10*	*3.70E-09*	*12.71527205*
								PCSK5	*-0.528607167*	*5.567697788*	*-6.925324905*	*3.61E-10*	*3.73E-09*	*12.70631009*
								TRPV4	*0.57188819*	*4.526803047*	*6.923013398*	*3.65E-10*	*3.77E-09*	*12.69534039*
								RN7SL1	*-0.612468243*	*10.79290288*	*-6.921650626*	*3.68E-10*	*3.80E-09*	*12.68887373*
								PSAP	*0.860037646*	*9.270364695*	*6.920877687*	*3.69E-10*	*3.81E-09*	*12.68520617*
								PGD	*0.753587084*	*6.626376142*	*6.918121442*	*3.74E-10*	*3.86E-09*	*12.67212912*

								PDLIM3	*-0.798628746*	*7.830528665*	*-6.916729712*	*3.77E-10*	*3.88E-09*	*12.66552676*
								MYL9	*-0.973338583*	*9.850378125*	*-6.913199615*	*3.83E-10*	*3.94E-09*	*12.64878218*
								MS4A2	*0.522672826*	*4.630953988*	*6.908690321*	*3.92E-10*	*4.03E-09*	*12.62739747*
								RAP2B	*0.590093517*	*5.845829986*	*6.902284506*	*4.04E-10*	*4.15E-09*	*12.59702759*
								AKT3	*-0.536481255*	*7.06730265*	*-6.901641093*	*4.05E-10*	*4.16E-09*	*12.59397775*
								C16orf89	*-1.141879661*	*6.295864034*	*-6.899095962*	*4.10E-10*	*4.21E-09*	*12.58191462*
								SOBP	*-0.585732399*	*5.536359638*	*-6.898577017*	*4.11E-10*	*4.21E-09*	*12.57945518*
								SDSL	*0.775762089*	*7.662119372*	*6.89614049*	*4.16E-10*	*4.26E-09*	*12.56790866*
								MRC1	*0.906625355*	*6.680778479*	*6.89365698*	*4.21E-10*	*4.31E-09*	*12.55614104*
								SNORD114-28	*-0.577150177*	*4.918936942*	*-6.892499292*	*4.23E-10*	*4.33E-09*	*12.5506561*
								NEURL3	*0.794827929*	*4.659472652*	*6.892437097*	*4.24E-10*	*4.33E-09*	*12.55036144*
								UBE2T	*0.597074622*	*5.639584812*	*6.87840026*	*4.53E-10*	*4.61E-09*	*12.48388451*
								THY1	*0.811233276*	*8.615148576*	*6.877886852*	*4.54E-10*	*4.62E-09*	*12.48145402*
								HLA-DQA1	*1.506021103*	*7.957719953*	*6.870012012*	*4.72E-10*	*4.79E-09*	*12.44418272*
								LOC100129662	*-0.561030202*	*5.071117078*	*-6.869572383*	*4.73E-10*	*4.80E-09*	*12.44210244*
								SCARB1	*0.965122844*	*8.661440582*	*6.863640352*	*4.87E-10*	*4.92E-09*	*12.41403756*
								PMFBP1	*0.561062396*	*4.584451718*	*6.861076476*	*4.93E-10*	*4.98E-09*	*12.40191047*
								SYNM	*-1.13196365*	*7.352566961*	*-6.860390967*	*4.95E-10*	*4.99E-09*	*12.39866831*
								ANGPTL7	*-1.152743283*	*4.77084037*	*-6.857642511*	*5.01E-10*	*5.05E-09*	*12.38567053*
								SCARA3	*-0.788641523*	*7.670137198*	*-6.854433787*	*5.09E-10*	*5.12E-09*	*12.37049855*
								HEXB	*0.655858039*	*8.308148437*	*6.854030023*	*5.10E-10*	*5.13E-09*	*12.3685896*
								TM6SF1	*0.638561136*	*5.306932476*	*6.851003838*	*5.17E-10*	*5.20E-09*	*12.35428348*

								TCAP	*-0.635158651*	*6.000456197*	*-6.845315086*	*5.32E-10*	*5.34E-09*	*12.32739664*
								ID3	*-0.521950283*	*8.179659166*	*-6.842445486*	*5.39E-10*	*5.41E-09*	*12.3138372*
								TNNC2	*-0.557823598*	*5.404120043*	*-6.838994877*	*5.48E-10*	*5.49E-09*	*12.29753519*
								KCNAB1	*-1.011131676*	*6.03418477*	*-6.833633689*	*5.63E-10*	*5.63E-09*	*12.27221302*
								BCAT1	*0.837345752*	*6.341121346*	*6.833170765*	*5.64E-10*	*5.63E-09*	*12.27002687*
								PLAU	*0.848765702*	*7.804469589*	*6.830940685*	*5.70E-10*	*5.68E-09*	*12.25949614*
								SMPX	*-0.673707599*	*4.318252923*	*-6.830866123*	*5.70E-10*	*5.68E-09*	*12.25914407*
								TMEM30B	*-0.686325449*	*6.695156678*	*-6.822649621*	*5.93E-10*	*5.91E-09*	*12.22035606*
								CDC45	*0.624190925*	*5.215686813*	*6.820199324*	*6.00E-10*	*5.97E-09*	*12.20879226*
								XLOC_008000	*-0.593187288*	*5.793524128*	*-6.820157968*	*6.00E-10*	*5.97E-09*	*12.2085971*
								LOC100288432	*0.704373721*	*4.759935559*	*6.819318204*	*6.03E-10*	*5.99E-09*	*12.20463433*
								SHC4	*-0.856390201*	*5.858073247*	*-6.813355039*	*6.21E-10*	*6.15E-09*	*12.17650003*
								CLEC7A	*0.546174583*	*4.709424494*	*6.811748002*	*6.25E-10*	*6.20E-09*	*12.16891961*
								XLOC_008644	*0.802088554*	*4.704880485*	*6.811024729*	*6.28E-10*	*6.21E-09*	*12.16550813*
								PMEPA1	*-0.537222037*	*7.731274915*	*-6.803697514*	*6.50E-10*	*6.42E-09*	*12.13095554*
								TPM2	*-0.853037557*	*11.31881016*	*-6.800811833*	*6.59E-10*	*6.51E-09*	*12.11735155*
								TSPYL2	*-0.528691961*	*5.716850566*	*-6.799567709*	*6.63E-10*	*6.54E-09*	*12.11148705*
								CATSPER1	*0.655504299*	*4.912617672*	*6.797787567*	*6.69E-10*	*6.59E-09*	*12.1030966*
								FXYD1	*-0.873711347*	*7.342619262*	*-6.796169596*	*6.74E-10*	*6.63E-09*	*12.09547125*
								PID1	*-0.656528205*	*7.186691086*	*-6.791823854*	*6.88E-10*	*6.76E-09*	*12.0749936*
								COL6A3	*0.565419808*	*5.788428031*	*6.79017932*	*6.94E-10*	*6.81E-09*	*12.06724566*
								MMD	*0.64743147*	*6.804377731*	*6.784717197*	*7.12E-10*	*6.97E-09*	*12.04151698*

								DDIT4L	*0.705876975*	*6.236164614*	*6.779974295*	*7.29E-10*	*7.12E-09*	*12.01918254*
								SORL1	*0.610017131*	*8.343140634*	*6.779199318*	*7.31E-10*	*7.14E-09*	*12.01553373*
								ANGPTL1	*-0.879892807*	*5.513621205*	*-6.773376092*	*7.52E-10*	*7.31E-09*	*11.98812143*
								BDNF	*-0.682976204*	*4.590244688*	*-6.768117612*	*7.71E-10*	*7.48E-09*	*11.96337542*
								NR1D1	*-0.77915215*	*5.627765491*	*-6.766314108*	*7.78E-10*	*7.54E-09*	*11.95488997*
								LOC283174	*-0.936935432*	*6.190053778*	*-6.765948319*	*7.80E-10*	*7.55E-09*	*11.95316905*
								PTGS1	*0.763494062*	*5.718913067*	*6.762738391*	*7.92E-10*	*7.66E-09*	*11.93806888*
								SLC12A8	*0.522554139*	*4.428440237*	*6.760926016*	*7.99E-10*	*7.71E-09*	*11.92954431*
								HLA-DRB5	*1.14037928*	*9.288567736*	*6.757999035*	*8.10E-10*	*7.81E-09*	*11.91577902*
								XLOC_003146	*0.561474889*	*4.875067678*	*6.75776789*	*8.11E-10*	*7.81E-09*	*11.91469206*
								PPAPDC1A	*0.891119685*	*4.757360778*	*6.756169103*	*8.17E-10*	*7.87E-09*	*11.90717421*
								KIF2C	*0.521613818*	*5.300709788*	*6.755020648*	*8.22E-10*	*7.91E-09*	*11.90177434*
								TRIM58	*0.794201108*	*5.128058869*	*6.749962927*	*8.42E-10*	*8.09E-09*	*11.87799789*
								RAB11FIP1	*0.530304772*	*6.328553387*	*6.747226741*	*8.53E-10*	*8.19E-09*	*11.86513791*
								HILPDA	*-1.172350875*	*7.440516925*	*-6.744616447*	*8.64E-10*	*8.29E-09*	*11.8528715*
								NBLA00301	*-0.963985285*	*4.525124531*	*-6.743421038*	*8.69E-10*	*8.33E-09*	*11.84725459*
								LOC730020	*0.54783327*	*4.480911569*	*6.739992592*	*8.83E-10*	*8.46E-09*	*11.8311474*
								PLCE1	*-0.74410753*	*6.0625407*	*-6.733080701*	*9.13E-10*	*8.73E-09*	*11.79868432*
								PPP1R12B	*-0.642250822*	*6.727238501*	*-6.730573717*	*9.24E-10*	*8.83E-09*	*11.78691298*
								MCOLN1	*0.632747606*	*5.507325278*	*6.724223487*	*9.52E-10*	*9.09E-09*	*11.75710364*
								SLC31A2	*1.05322771*	*7.377116092*	*6.723043302*	*9.58E-10*	*9.13E-09*	*11.75156481*
								PTTG1	*0.617734398*	*6.487307897*	*6.720126362*	*9.71E-10*	*9.25E-09*	*11.7378767*

								CTSZ	*0.9024<br>9084*	*6.4200<br>56412*	*6.7160<br>59789*	*9.90E-<br>10*	*9.42E-<br>09*	*11.718<br>79768*
								DRAM1	*0.7252<br>43887*	*7.9025<br>74835*	*6.7152<br>3933*	*9.94E-<br>10*	*9.45E-<br>09*	*11.714<br>9489*
								SGK1	*1.0334<br>84792*	*8.5806<br>71647*	*6.7147<br>73614*	*9.97E-<br>10*	*9.47E-<br>09*	*11.712<br>7643*
								PODNL1	*0.5226<br>75132*	*5.3044<br>1766*	*6.7138<br>578*	*1.00E-<br>09*	*9.50E-<br>09*	*11.708<br>46856*
								SCARNA5	*-0.7675<br>12067*	*6.7664<br>57303*	*-6.7052<br>48083*	*1.04E-<br>09*	*9.87E-<br>09*	*11.668<br>09474*
								LPXN	*0.7092<br>28248*	*6.2020<br>67587*	*6.6981<br>10952*	*1.08E-<br>09*	*1.02E-<br>08*	*11.634<br>64184*
								SLC25A19	*0.6052<br>09167*	*6.3821<br>18425*	*6.6977<br>3516*	*1.08E-<br>09*	*1.02E-<br>08*	*11.632<br>88083*
								GPR116	*0.6198<br>72549*	*5.6543<br>97446*	*6.6962<br>41011*	*1.09E-<br>09*	*1.03E-<br>08*	*11.625<br>87944*
								SNORA28	*-0.7993<br>81888*	*8.0765<br>95639*	*-6.6926<br>31133*	*1.11E-<br>09*	*1.04E-<br>08*	*11.608<br>96656*
								LILRA2	*0.7450<br>88327*	*4.9902<br>10316*	*6.6913<br>98352*	*1.11E-<br>09*	*1.05E-<br>08*	*11.603<br>1916*
								LOC28305<br>0	*0.8681<br>12676*	*5.5155<br>00901*	*6.6862<br>55801*	*1.14E-<br>09*	*1.07E-<br>08*	*11.579<br>10588*
								ABI3BP	*-0.5142<br>09004*	*5.6977<br>59409*	*-6.6857<br>80772*	*1.15E-<br>09*	*1.08E-<br>08*	*11.576<br>8814*
								GPC3	*-0.6823<br>54415*	*6.0740<br>71802*	*-6.6827<br>61552*	*1.16E-<br>09*	*1.09E-<br>08*	*11.562<br>74435*
								PI4K2A	*0.5002<br>6463*	*6.2243<br>61188*	*6.6790<br>24847*	*1.18E-<br>09*	*1.11E-<br>08*	*11.545<br>25129*
								APOBEC<br>3A	*0.8044<br>39208*	*4.6996<br>59196*	*6.6729<br>22564*	*1.22E-<br>09*	*1.14E-<br>08*	*11.516<br>69234*
								PLXNA1	*0.5873<br>58175*	*8.3060<br>73603*	*6.6687<br>21135*	*1.24E-<br>09*	*1.16E-<br>08*	*11.497<br>0355*
								SH3D19	*-0.6129<br>92558*	*8.0492<br>13753*	*-6.6656<br>214*	*1.26E-<br>09*	*1.18E-<br>08*	*11.482<br>53622*
								CXCR4	*1.2288<br>68087*	*8.9738<br>89874*	*6.6605<br>48529*	*1.29E-<br>09*	*1.20E-<br>08*	*11.458<br>81322*
								XLOC_l2<br>_000342	*0.7606<br>39571*	*4.6840<br>50458*	*6.6592<br>05389*	*1.30E-<br>09*	*1.21E-<br>08*	*11.452<br>5333*
								QPCT	*0.5489<br>36202*	*5.3145<br>88202*	*6.6558<br>35284*	*1.32E-<br>09*	*1.23E-<br>08*	*11.436<br>77845*
								SNORD6	*-0.5442<br>6922*	*5.6149<br>99758*	*-6.6531<br>62088*	*1.34E-<br>09*	*1.24E-<br>08*	*11.424<br>28383*
								RBM24	*-0.5896<br>79205*	*4.3704<br>29036*	*-6.6529<br>56071*	*1.34E-<br>09*	*1.24E-<br>08*	*11.423<br>32098*

								TSPAN8	*-1.090738331*	*5.323771745*	*-6.652937445*	*1.34E-09*	*1.24E-08*	*11.42323393*
								LPL	*1.241682033*	*6.77938935*	*6.651089106*	*1.35E-09*	*1.25E-08*	*11.41459602*
								RNF152	*-0.645322274*	*5.626174341*	*-6.650577964*	*1.36E-09*	*1.25E-08*	*11.41220745*
								MREG	*0.889986353*	*4.832487322*	*6.646671003*	*1.38E-09*	*1.28E-08*	*11.39395262*
								LOC100507008	*-0.878145037*	*4.946091406*	*-6.646637436*	*1.38E-09*	*1.28E-08*	*11.3937958*
								SUN2	*-0.548812256*	*6.740496579*	*-6.639466707*	*1.43E-09*	*1.32E-08*	*11.36030266*
								SNORD114-11	*-0.526765888*	*4.699973327*	*-6.639355372*	*1.43E-09*	*1.32E-08*	*11.35978276*
								GIMAP5	*0.522509955*	*6.402076099*	*6.639231903*	*1.43E-09*	*1.32E-08*	*11.35920618*
								PPARG	*0.675265689*	*5.572291738*	*6.636161513*	*1.45E-09*	*1.33E-08*	*11.34486961*
								C20orf46	*0.579780581*	*5.061063796*	*6.635489402*	*1.46E-09*	*1.34E-08*	*11.34173168*
								SCARNA11	*-0.705227194*	*5.470987569*	*-6.628522543*	*1.51E-09*	*1.38E-08*	*11.30921255*
								RYR3	*-0.788820791*	*5.135340376*	*-6.628345021*	*1.51E-09*	*1.38E-08*	*11.30838411*
								LY6H	*0.938324272*	*4.71362591*	*6.625023246*	*1.53E-09*	*1.40E-08*	*11.29288411*
								RASGEF1B	*0.786098064*	*5.63647307*	*6.611881909*	*1.63E-09*	*1.48E-08*	*11.23159509*
								PODN	*-0.661120751*	*4.988924712*	*-6.610356174*	*1.64E-09*	*1.49E-08*	*11.22448251*
								DCN	*-0.888374043*	*9.459011908*	*-6.608938429*	*1.65E-09*	*1.50E-08*	*11.21787394*
								XLOC_012031	*-0.549422433*	*4.919533651*	*-6.608222767*	*1.66E-09*	*1.50E-08*	*11.21453823*
								FILIP1L	*-0.500799794*	*5.874774698*	*-6.60670209*	*1.67E-09*	*1.51E-08*	*11.20745082*
								VCAM1	*0.741853948*	*6.592880959*	*6.605137921*	*1.68E-09*	*1.52E-08*	*11.20016139*
								ACTN2	*-0.533286635*	*4.46320118*	*-6.604190633*	*1.69E-09*	*1.53E-08*	*11.19574713*
								CAND2	*-0.682623025*	*6.045026109*	*-6.599725645*	*1.73E-09*	*1.56E-08*	*11.17494423*
								ITPKC	*-0.556879639*	*7.342912679*	*-6.592566163*	*1.79E-09*	*1.61E-08*	*11.14159932*

								SERPINA1	*0.588292244*	*5.510026987*	*6.592448643*	*1.79E-09*	*1.61E-08*	*11.1410521*
								TMEM176A	*0.913489148*	*6.911781025*	*6.592407655*	*1.79E-09*	*1.61E-08*	*11.14086124*
								RSAD2	*0.712944928*	*6.06912439*	*6.587363593*	*1.83E-09*	*1.65E-08*	*11.11737788*
								RASGRP1	*0.600466216*	*5.159889789*	*6.585056093*	*1.85E-09*	*1.66E-08*	*11.10663743*
								PAPPA	*-0.53582738*	*6.0016307*	*-6.584182253*	*1.86E-09*	*1.67E-08*	*11.10257047*
								ITM2A	*-0.891835205*	*7.462018468*	*-6.582563248*	*1.87E-09*	*1.68E-08*	*11.09503601*
								S1PR3	*-0.921380782*	*8.038326223*	*-6.57939881*	*1.90E-09*	*1.70E-08*	*11.08031165*
								HEYL	*-0.669706231*	*7.256695279*	*-6.574090646*	*1.95E-09*	*1.74E-08*	*11.05561889*
								ELOVL7	*-0.591350545*	*4.555801279*	*-6.569443379*	*1.99E-09*	*1.78E-08*	*11.03400724*
								HLA-DQA2	*1.41916562*	*7.387835852*	*6.567693191*	*2.01E-09*	*1.79E-08*	*11.0258698*
								SYPL2	*-0.547787596*	*5.28556172*	*-6.564915928*	*2.04E-09*	*1.81E-08*	*11.01295883*
								CD5	*0.690461331*	*5.071756051*	*6.562481047*	*2.06E-09*	*1.83E-08*	*11.00164137*
								TFCP2L1	*0.786175391*	*4.868483073*	*6.561280104*	*2.07E-09*	*1.84E-08*	*10.99605996*
								SNAR-A3	*0.632131187*	*8.811283936*	*6.559669894*	*2.09E-09*	*1.85E-08*	*10.98857714*
								LUM	*0.750175835*	*9.153138115*	*6.556465281*	*2.12E-09*	*1.88E-08*	*10.97368719*
								PLBD1	*1.06197075*	*7.714002282*	*6.551786485*	*2.17E-09*	*1.92E-08*	*10.95195298*
								CD82	*0.513290815*	*7.681272659*	*6.546141165*	*2.23E-09*	*1.97E-08*	*10.92573755*
								DRAM2	*0.54241963*	*6.674138556*	*6.54492786*	*2.24E-09*	*1.98E-08*	*10.92010449*
								CXCR2	*0.902527333*	*4.634359405*	*6.544597155*	*2.24E-09*	*1.98E-08*	*10.91856919*
								TGFBR3	*-0.661005557*	*5.63616619*	*-6.535618373*	*2.34E-09*	*2.06E-08*	*10.87689737*
								ICAM4	*0.64513705*	*4.911801382*	*6.535362084*	*2.35E-09*	*2.06E-08*	*10.87570824*
								ATF5	*0.558645132*	*5.876149291*	*6.535069647*	*2.35E-09*	*2.06E-08*	*10.87435142*

								SNORA27	-0.72109902	8.380732238	-6.533561316	2.37E-09	2.08E-08	10.86735361
								HMMR	0.624770874	4.759669901	6.533197552	2.37E-09	2.08E-08	10.86566605
								COBLL1	-0.567802052	7.817618675	-6.52937215	2.41E-09	2.11E-08	10.84792176
								B3GNT5	0.80142703	5.960277976	6.528405056	2.42E-09	2.12E-08	10.84343653
								PPIF	0.755072633	7.332633585	6.527355538	2.44E-09	2.13E-08	10.83856935
								NFIX	-0.603833448	6.23418618	-6.523020576	2.49E-09	2.17E-08	10.81846925
								NRIP3	0.575919932	4.738608016	6.521771706	2.50E-09	2.18E-08	10.81267959
								LOC100131733	0.729477808	5.320871135	6.509220142	2.66E-09	2.30E-08	10.75451731
								TCN2	0.664772591	5.708294793	6.505614259	2.70E-09	2.34E-08	10.73781679
								GZMK	0.987450065	5.810325426	6.505395358	2.70E-09	2.34E-08	10.73680308
								FCER1G	0.729341399	6.664180997	6.50263961	2.74E-09	2.37E-08	10.72404272
								PEBP4	-0.598698562	5.607032037	-6.491824949	2.88E-09	2.48E-08	10.6739879
								MTUS2	-0.721262891	4.344894299	-6.488976163	2.92E-09	2.51E-08	10.66080835
								PAWR	-0.649882431	7.952801222	-6.479932875	3.05E-09	2.62E-08	10.61898685
								NOV	-0.943763527	9.69204698	-6.478918596	3.06E-09	2.63E-08	10.61429776
								C12orf49	0.539105256	5.168891372	6.478843009	3.07E-09	2.63E-08	10.61394833
								RIN3	0.601961384	6.404337877	6.476662254	3.10E-09	2.65E-08	10.60386763
								CXCL10	0.826075836	5.085335801	6.472948924	3.15E-09	2.69E-08	10.5867058
								ITGA5	-0.851066884	7.720276487	-6.472919515	3.15E-09	2.69E-08	10.5865699
								PTGIS	-0.533700766	8.892589149	-6.465902386	3.26E-09	2.77E-08	10.55415039
								CKMT2	-0.813895956	6.116726472	-6.46396165	3.29E-09	2.80E-08	10.54518672
								PLEKHB2	0.516673758	6.607139954	6.46110579	3.33E-09	2.83E-08	10.53199845

								GGT8P	0.562820771	4.494931882	6.460044916	3.35E-09	2.85E-08	10.52709999
								ATP6V0D2	0.825631314	4.557091865	6.450673684	3.50E-09	2.97E-08	10.48384436
								HAND2	-1.169355295	4.968013617	-6.439736375	3.69E-09	3.11E-08	10.43339389
								LOC100270746	-0.646366088	5.836710154	-6.436880897	3.74E-09	3.15E-08	10.42022846
								SNORD76	-0.505671272	5.871101322	-6.434467688	3.78E-09	3.18E-08	10.4091041
								SIGLEC14	0.544496884	5.597145987	6.431484214	3.83E-09	3.23E-08	10.39535341
								THRA	-0.572227022	7.420895378	-6.427186871	3.91E-09	3.29E-08	10.37555196
								SPON1	-1.43247642	6.194478248	-6.424194649	3.97E-09	3.33E-08	10.36176765
								AMIGO2	-0.665334961	7.379849089	-6.421712215	4.02E-09	3.36E-08	10.35033388
								TNFAIP2	0.869671753	9.365280062	6.415734343	4.13E-09	3.45E-08	10.32280837
								TAGLN	-0.799449872	10.69799498	-6.409673696	4.25E-09	3.55E-08	10.29491299
								XLOC_005811	0.523811434	4.393489746	6.398252833	4.48E-09	3.73E-08	10.24237702
								KLK1	0.726229218	4.550094734	6.397831023	4.49E-09	3.73E-08	10.24043747
								MT2A	-0.627938559	11.41860227	-6.395213554	4.55E-09	3.78E-08	10.22840317
								C11orf96	-1.128555895	11.25391919	-6.389942001	4.66E-09	3.86E-08	10.20417269
								PGM5	-0.913412578	6.440067868	-6.387970134	4.71E-09	3.89E-08	10.19511131
								GNG8	-0.568599922	5.160220199	-6.374891109	5.01E-09	4.12E-08	10.13503964
								CYP2S1	0.555027001	4.589445258	6.374351046	5.02E-09	4.13E-08	10.1325603
								IL17RD	-0.558695514	5.046044957	-6.36917256	5.14E-09	4.22E-08	10.10879133
								C15orf48	1.536084576	6.564530889	6.36911246	5.14E-09	4.22E-08	10.10851552
								TMOD1	-0.812307383	5.873351918	-6.359789875	5.37E-09	4.40E-08	10.0657468
								KLRB1	0.800155906	5.858864611	6.359464196	5.38E-09	4.41E-08	10.06425319

								KIAA0125	*0.554567398*	*4.350275709*	*6.358848269*	*5.40E-09*	*4.42E-08*	*10.06142857*
								POLR2J2	*-0.575575373*	*6.269138565*	*-6.357675157*	*5.43E-09*	*4.44E-08*	*10.05604903*
								COL10A1	*0.911164116*	*5.950072316*	*6.354448435*	*5.51E-09*	*4.50E-08*	*10.04125452*
								ICOS	*0.513847438*	*4.726925172*	*6.350568938*	*5.61E-09*	*4.57E-08*	*10.02347138*
								TNNC1	*-0.549305626*	*4.743545375*	*-6.346338804*	*5.72E-09*	*4.66E-08*	*10.00408641*
								XLOC_l2_001986	*0.507309164*	*4.417534153*	*6.34013262*	*5.89E-09*	*4.79E-08*	*9.975656275*
								FLJ42875	*-0.793193816*	*6.418549535*	*-6.336812995*	*5.98E-09*	*4.86E-08*	*9.960454314*
								ALDH1L1	*-0.866755319*	*5.19997202*	*-6.322175223*	*6.41E-09*	*5.17E-08*	*9.893463678*
								HSPA6	*0.592248006*	*5.443597403*	*6.321969787*	*6.42E-09*	*5.17E-08*	*9.892523975*
								FMO3	*0.791246031*	*5.808014213*	*6.313673824*	*6.67E-09*	*5.35E-08*	*9.854587981*
								SLC17A9	*0.778068957*	*6.505121958*	*6.312568301*	*6.70E-09*	*5.38E-08*	*9.849534282*
								FGF13	*-0.658865335*	*6.953187091*	*-6.308906837*	*6.82E-09*	*5.46E-08*	*9.832799375*
								XLOC_014336	*0.548292893*	*4.510966879*	*6.305327175*	*6.94E-09*	*5.54E-08*	*9.816442515*
								PTGER2	*0.522141556*	*4.999179157*	*6.303823011*	*6.99E-09*	*5.58E-08*	*9.809570639*
								FBLIM1	*-0.551320749*	*7.378433853*	*-6.292075007*	*7.38E-09*	*5.87E-08*	*9.75592421*
								CFP	*0.794129419*	*6.729850417*	*6.288145257*	*7.52E-09*	*5.97E-08*	*9.737989246*
								LOC729468	*-0.894181033*	*5.935824387*	*-6.278962929*	*7.85E-09*	*6.22E-08*	*9.696101609*
								SLMAP	*-0.596216725*	*6.832216605*	*-6.276699267*	*7.93E-09*	*6.28E-08*	*9.685779523*
								RBPMS2	*-1.273857115*	*5.451948339*	*-6.267617721*	*8.27E-09*	*6.53E-08*	*9.644385312*
								MPDZ	*-0.51577937*	*6.890330698*	*-6.261749062*	*8.50E-09*	*6.69E-08*	*9.61764996*
								HPSE2	*-0.838354176*	*4.736481795*	*-6.249526374*	*9.00E-09*	*7.06E-08*	*9.562004321*
								ECEL1P2	*0.513849485*	*4.471805464*	*6.247464095*	*9.09E-09*	*7.12E-08*	*9.552620325*

								MOXD1	*0.809407759*	*5.354984956*	*6.244225232*	*9.23E-09*	*7.22E-08*	*9.537885335*
								APOC1	*2.277102696*	*8.04424735*	*6.24178147*	*9.33E-09*	*7.29E-08*	*9.526769896*
								CERS1	*-0.602126008*	*4.765211994*	*-6.23608881*	*9.58E-09*	*7.47E-08*	*9.5008845*
								FAM26F	*0.734458372*	*6.405614844*	*6.233018114*	*9.72E-09*	*7.58E-08*	*9.486926018*
								BCAM	*-0.504494647*	*4.716866908*	*-6.230978576*	*9.81E-09*	*7.64E-08*	*9.477656606*
								MAOB	*-0.721296006*	*6.433235311*	*-6.230611828*	*9.83E-09*	*7.65E-08*	*9.475989934*
								MYO18B	*-0.6006786*	*4.453387377*	*-6.229935918*	*9.86E-09*	*7.67E-08*	*9.472918404*
								GFRA1	*-0.734270481*	*5.258985984*	*-6.229419779*	*9.89E-09*	*7.69E-08*	*9.470573022*
								CRIP2	*-0.524431002*	*8.188126196*	*-6.228613821*	*9.92E-09*	*7.71E-08*	*9.466910852*
								NCEH1	*0.790352347*	*7.127821487*	*6.225458372*	*1.01E-08*	*7.81E-08*	*9.452574971*
								CSRP2	*-0.888445912*	*9.438939716*	*-6.223777944*	*1.01E-08*	*7.87E-08*	*9.444941775*
								KMO	*0.805740257*	*5.09922904*	*6.216750454*	*1.05E-08*	*8.11E-08*	*9.413030185*
								IL6R	*0.541917873*	*5.868306775*	*6.211781939*	*1.07E-08*	*8.28E-08*	*9.39047825*
								DES	*-2.114279871*	*9.29726157*	*-6.211534763*	*1.07E-08*	*8.29E-08*	*9.389356542*
								LIMS2	*-0.597372594*	*8.246003362*	*-6.208713265*	*1.09E-08*	*8.39E-08*	*9.376553751*
								DOPEY2	*0.509234036*	*4.795330289*	*6.205407652*	*1.11E-08*	*8.51E-08*	*9.361557624*
								PDE4D	*-0.652787924*	*5.203347596*	*-6.201195236*	*1.13E-08*	*8.67E-08*	*9.342453025*
								HTRA3	*-0.699436724*	*4.834639086*	*-6.198211386*	*1.14E-08*	*8.78E-08*	*9.328923944*
								LOC100505504	*-0.504563566*	*4.583738915*	*-6.19795429*	*1.14E-08*	*8.79E-08*	*9.327758383*
								TIMP4	*-0.866007012*	*6.187519249*	*-6.195614072*	*1.16E-08*	*8.87E-08*	*9.317149887*
								LOC644189	*0.648675478*	*5.287780106*	*6.193304231*	*1.17E-08*	*8.96E-08*	*9.306680895*
								MGST1	*-0.963233891*	*6.868192817*	*-6.191620768*	*1.18E-08*	*9.02E-08*	*9.299051992*

								NOXA1	*0.591038135*	*6.466117125*	*6.187435073*	*1.20E-08*	*9.17E-08*	*9.28008792*
								PTGIR	*-0.558173347*	*7.846299356*	*-6.181602541*	*1.23E-08*	*9.41E-08*	*9.25367238*
								SIGLEC9	*0.671394474*	*4.915826753*	*6.174028584*	*1.28E-08*	*9.73E-08*	*9.219387061*
								JAM3	*-0.515298258*	*5.940936633*	*-6.173138826*	*1.28E-08*	*9.77E-08*	*9.215360629*
								NET1	*-0.709232347*	*8.151235996*	*-6.170054796*	*1.30E-08*	*9.89E-08*	*9.20140652*
								SLPI	*-1.049442416*	*6.202698433*	*-6.166770491*	*1.32E-08*	*1.00E-07*	*9.186549775*
								DBP	*0.665566636*	*5.600324665*	*6.165264036*	*1.33E-08*	*1.01E-07*	*9.179736465*
								CST6	*-0.837084144*	*6.7405236*	*-6.160755132*	*1.36E-08*	*1.03E-07*	*9.159348444*
								SNORD27	*-0.543040668*	*5.846126974*	*-6.16067586*	*1.36E-08*	*1.03E-07*	*9.15899006*
								HS3ST2	*1.507257468*	*6.187200564*	*6.158271708*	*1.38E-08*	*1.04E-07*	*9.14812204*
								SOX8	*-0.604799264*	*5.647696443*	*-6.157539797*	*1.38E-08*	*1.04E-07*	*9.144813808*
								NAP1L2	*-0.516728781*	*4.960132905*	*-6.155236959*	*1.40E-08*	*1.05E-07*	*9.134406193*
								IL18	*0.872695691*	*5.600354048*	*6.153564923*	*1.41E-08*	*1.06E-07*	*9.126850606*
								NLN	*0.506378369*	*5.266612562*	*6.14915941*	*1.44E-08*	*1.08E-07*	*9.106947553*
								ANGPTL5	*-0.77326639*	*4.720632289*	*-6.142321052*	*1.48E-08*	*1.11E-07*	*9.076066618*
								XLOC_011331	*0.637204659*	*4.454845417*	*6.136312495*	*1.52E-08*	*1.14E-07*	*9.04894614*
								HES4	*-0.784150425*	*9.315311891*	*-6.133812665*	*1.54E-08*	*1.15E-07*	*9.037666445*
								GGTLC1	*0.675570859*	*5.703016169*	*6.131856945*	*1.55E-08*	*1.16E-07*	*9.028843366*
								TGFB3	*-0.701602156*	*6.567491341*	*-6.107007459*	*1.74E-08*	*1.29E-07*	*8.916851602*
								CD163	*1.081511168*	*9.376809412*	*6.105436007*	*1.76E-08*	*1.30E-07*	*8.909776552*
								NINJ1	*0.534185555*	*9.689642113*	*6.099540584*	*1.81E-08*	*1.33E-07*	*8.883241589*
								CD8B	*0.551736568*	*5.075167809*	*6.098598561*	*1.81E-08*	*1.34E-07*	*8.879002715*

								CYP27A1	*0.885880571*	*8.869685183*	*6.09564741*	*1.84E-08*	*1.35E-07*	*8.865725251*
								ID1	*-0.753928541*	*8.684847236*	*-6.09422093*	*1.85E-08*	*1.36E-07*	*8.859308492*
								CDH3	*0.546409053*	*4.620584688*	*6.092839644*	*1.86E-08*	*1.37E-07*	*8.853095705*
								TK1	*0.811264865*	*7.675211925*	*6.09155303*	*1.87E-08*	*1.38E-07*	*8.847309331*
								MYH10	*-0.940262995*	*7.802087283*	*-6.090533435*	*1.88E-08*	*1.38E-07*	*8.84272425*
								ADORA3	*0.858161344*	*5.328132436*	*6.072831421*	*2.04E-08*	*1.49E-07*	*8.763176845*
								GZMH	*0.884870302*	*6.509073223*	*6.066754725*	*2.10E-08*	*1.53E-07*	*8.735895355*
								ASGR2	*0.55705545*	*5.432300259*	*6.060064151*	*2.17E-08*	*1.58E-07*	*8.705872861*
								SLC39A11	*0.565526388*	*6.687260658*	*6.059623328*	*2.17E-08*	*1.58E-07*	*8.703895314*
								ABCC3	*0.649213391*	*6.400058442*	*6.048893013*	*2.28E-08*	*1.66E-07*	*8.65577999*
								GNGT2	*0.526723261*	*4.768008952*	*6.047734496*	*2.29E-08*	*1.66E-07*	*8.650587571*
								C8orf84	*-1.242048906*	*6.837868367*	*-6.047336745*	*2.30E-08*	*1.67E-07*	*8.648804977*
								MRVI1	*-0.716306577*	*9.364121197*	*-6.046622998*	*2.30E-08*	*1.67E-07*	*8.645606331*
								GZMB	*0.895914574*	*6.682838308*	*6.043779555*	*2.33E-08*	*1.69E-07*	*8.632865285*
								XLOC_l2_007424	*-0.687246027*	*5.020839604*	*-6.040883151*	*2.37E-08*	*1.71E-07*	*8.619889876*
								FAM43A	*0.713828543*	*6.125919635*	*6.037078092*	*2.41E-08*	*1.74E-07*	*8.602848366*
								RARRES3	*0.505258306*	*8.435715698*	*6.035561654*	*2.42E-08*	*1.75E-07*	*8.596058208*
								MGAT4A	*0.589137394*	*6.654997204*	*6.032677189*	*2.46E-08*	*1.77E-07*	*8.583144697*
								CAMK2N1	*-0.644771181*	*6.292589462*	*-6.031583659*	*2.47E-08*	*1.78E-07*	*8.578249826*
								ADAMTS8	*-1.073353632*	*6.949881598*	*-6.029820004*	*2.49E-08*	*1.79E-07*	*8.570356234*
								COL4A3	*-0.510912105*	*4.273513663*	*-6.028997835*	*2.50E-08*	*1.80E-07*	*8.566676828*
								PYGM	*-0.609218877*	*5.286536229*	*-6.019353443*	*2.61E-08*	*1.88E-07*	*8.523533854*

								VNN2	*0.862940632*	*6.458059397*	*6.017895513*	*2.63E-08*	*1.89E-07*	*8.51701488*
								LOC158402	*0.524041508*	*4.542255162*	*6.01597944*	*2.65E-08*	*1.90E-07*	*8.508448527*
								JAKMIP2	*0.779859877*	*4.911924686*	*6.008286863*	*2.75E-08*	*1.97E-07*	*8.474069888*
								SLC2A9	*0.583997585*	*5.284505745*	*6.004309105*	*2.80E-08*	*2.00E-07*	*8.456301351*
								XLOC_001788	*0.594756544*	*5.481433604*	*5.997926613*	*2.88E-08*	*2.05E-07*	*8.42780282*
								JMJD7-PLA2G4B	*0.505559621*	*7.547453397*	*5.997634505*	*2.88E-08*	*2.06E-07*	*8.426498877*
								CRYBB1	*0.586444997*	*4.717015179*	*5.996109001*	*2.90E-08*	*2.07E-07*	*8.419689666*
								NAPSA	*0.509132437*	*5.09979986*	*5.990639961*	*2.98E-08*	*2.12E-07*	*8.39528506*
								PRDM16	*-0.515319578*	*5.943718723*	*-5.987565606*	*3.02E-08*	*2.15E-07*	*8.381571049*
								PPP1R14A	*-0.536386181*	*9.721802562*	*-5.984998575*	*3.06E-08*	*2.17E-07*	*8.370122711*
								LARGE	*-0.57539219*	*6.357047579*	*-5.98493593*	*3.06E-08*	*2.17E-07*	*8.369843359*
								SLC6A12	*0.565321466*	*4.349838538*	*5.984507918*	*3.06E-08*	*2.17E-07*	*8.367934775*
								RNU105C	*0.648710396*	*4.45281528*	*5.983900899*	*3.07E-08*	*2.18E-07*	*8.36522807*
								ENTPD2	*-0.553243267*	*4.684236161*	*-5.979284451*	*3.14E-08*	*2.22E-07*	*8.344647673*
								DLX5	*0.894692596*	*5.206353128*	*5.972231439*	*3.24E-08*	*2.29E-07*	*8.313219853*
								LMOD1	*-0.927655941*	*7.011825952*	*-5.968431156*	*3.30E-08*	*2.32E-07*	*8.296293503*
								RAD51	*0.516466966*	*4.849301176*	*5.964912014*	*3.35E-08*	*2.36E-07*	*8.280624036*
								GNA15	*0.52885236*	*5.266719972*	*5.96409133*	*3.36E-08*	*2.37E-07*	*8.276970476*
								P2RY8	*0.569505698*	*5.737635688*	*5.964055965*	*3.36E-08*	*2.37E-07*	*8.276813038*
								PACSIN1	*0.614059332*	*5.161132762*	*5.963739158*	*3.37E-08*	*2.37E-07*	*8.275402733*
								TNNT1	*0.836675635*	*5.129259594*	*5.958685899*	*3.45E-08*	*2.41E-07*	*8.252912453*
								NDC80	*0.529601147*	*5.814023676*	*5.957319974*	*3.47E-08*	*2.43E-07*	*8.246834802*

								PTP4A3	*-0.704874733*	*6.759573124*	*-5.955851393*	*3.49E-08*	*2.44E-07*	*8.240301148*
								TMEM61	*-1.0369093*	*5.57059365*	*-5.948944036*	*3.60E-08*	*2.51E-07*	*8.209581184*
								DGKD	*-0.623736169*	*7.63524426*	*-5.946863475*	*3.64E-08*	*2.53E-07*	*8.200331466*
								CILP	*-1.31720519*	*5.364588518*	*-5.94292181*	*3.70E-08*	*2.58E-07*	*8.182812045*
								PABPC4L	*-0.526664852*	*5.041188922*	*-5.938227328*	*3.78E-08*	*2.63E-07*	*8.16195404*
								COCH	*-0.553708562*	*4.302107894*	*-5.937091339*	*3.80E-08*	*2.64E-07*	*8.156907957*
								XLOC_l2_010897	*0.57743852*	*4.862844347*	*5.93605886*	*3.82E-08*	*2.65E-07*	*8.152322078*
								STMN2	*1.268494728*	*5.300148491*	*5.933869056*	*3.86E-08*	*2.67E-07*	*8.142597102*
								CLEC5A	*0.78252723*	*4.920121961*	*5.921794878*	*4.08E-08*	*2.82E-07*	*8.089007134*
								CTSL1	*0.625449922*	*8.031904451*	*5.913416*	*4.24E-08*	*2.92E-07*	*8.051850052*
								CSF2RA	*0.591826925*	*5.091380219*	*5.895010635*	*4.61E-08*	*3.16E-07*	*7.970320943*
								TIMD4	*0.931537456*	*5.061060841*	*5.890421775*	*4.70E-08*	*3.22E-07*	*7.950013622*
								NFIB	*-0.566783524*	*7.205799362*	*-5.880785226*	*4.92E-08*	*3.36E-07*	*7.907394149*
								CRTAC1	*0.987680685*	*7.1947989*	*5.879528949*	*4.94E-08*	*3.37E-07*	*7.901840587*
								SELL	*0.705995403*	*5.684228473*	*5.877828053*	*4.98E-08*	*3.40E-07*	*7.894322472*
								LOC100288911	*-0.577063755*	*7.076796531*	*-5.872678943*	*5.10E-08*	*3.47E-07*	*7.871569546*
								MAFF	*-0.782644728*	*6.990213524*	*-5.871332961*	*5.13E-08*	*3.49E-07*	*7.865623556*
								SNORA81	*-0.573655397*	*6.816144433*	*-5.869690341*	*5.17E-08*	*3.51E-07*	*7.858368065*
								HBD	*1.576993142*	*10.22764651*	*5.868213246*	*5.20E-08*	*3.53E-07*	*7.851844571*
								ADAMDEC1	*0.846350448*	*4.67634213*	*5.853172385*	*5.57E-08*	*3.77E-07*	*7.785464467*
								ASAH1	*0.646280749*	*9.187889776*	*5.851594604*	*5.61E-08*	*3.79E-07*	*7.778506177*
								CNIH3	*0.728428862*	*4.994558829*	*5.845632352*	*5.76E-08*	*3.88E-07*	*7.752220119*

								GPR84	*0.676277296*	*5.228359286*	*5.844733961*	*5.79E-08*	*3.90E-07*	*7.748260514*
								KIAA0101	*0.694429268*	*5.18194652*	*5.841197495*	*5.88E-08*	*3.95E-07*	*7.732676717*
								HBB	*0.663501134*	*7.734896999*	*5.839635212*	*5.92E-08*	*3.98E-07*	*7.725793874*
								HDC	*0.81546334*	*6.187764051*	*5.835514743*	*6.04E-08*	*4.05E-07*	*7.707645059*
								CARTPT	*-1.034215672*	*4.59436846*	*-5.832364641*	*6.12E-08*	*4.10E-07*	*7.69377464*
								TMEM132A	*0.598079249*	*6.490312578*	*5.831342034*	*6.15E-08*	*4.12E-07*	*7.689272745*
								ALOX5AP	*0.827043897*	*6.656748296*	*5.830333908*	*6.18E-08*	*4.14E-07*	*7.684834993*
								MEIS2	*-0.597813146*	*5.676833309*	*-5.829031831*	*6.21E-08*	*4.16E-07*	*7.679103846*
								CASQ2	*-1.301435649*	*6.064107892*	*-5.823400062*	*6.38E-08*	*4.26E-07*	*7.654322843*
								XLOC_011608	*0.910578241*	*4.642616248*	*5.819686949*	*6.48E-08*	*4.32E-07*	*7.637990978*
								ADAMTS4	*-0.995561832*	*6.244953548*	*-5.817890928*	*6.54E-08*	*4.35E-07*	*7.630093198*
								IL32	*0.509388872*	*6.471663088*	*5.81413291*	*6.65E-08*	*4.42E-07*	*7.613571791*
								HIST1H1A	*-0.604687101*	*6.00943217*	*-5.808405146*	*6.82E-08*	*4.53E-07*	*7.588401196*
								DIO3	*-0.784191772*	*4.505413195*	*-5.805745192*	*6.90E-08*	*4.58E-07*	*7.576716347*
								UNC13C	*-0.692560551*	*4.735723047*	*-5.79771891*	*7.16E-08*	*4.73E-07*	*7.5414744*
								IFI6	*0.626968606*	*7.889873245*	*5.795803431*	*7.22E-08*	*4.77E-07*	*7.53306755*
								AGTR1	*-0.522364021*	*4.335526526*	*-5.792288905*	*7.34E-08*	*4.84E-07*	*7.517646329*
								VSTM1	*0.746830374*	*4.676971418*	*5.790073414*	*7.41E-08*	*4.88E-07*	*7.507927539*
								ZNF385A	*0.606836338*	*7.17774162*	*5.788250837*	*7.47E-08*	*4.92E-07*	*7.49993378*
								RDH10	*-0.510335294*	*5.45524761*	*-5.78104027*	*7.72E-08*	*5.06E-07*	*7.468321113*
								ASPN	*-0.68784056*	*7.660218944*	*-5.780506523*	*7.74E-08*	*5.08E-07*	*7.465981853*
								SYNE2	*-0.518283047*	*7.173865526*	*-5.78008969*	*7.75E-08*	*5.08E-07*	*7.464155072*

								SLC28A3	0.577691811	4.103265	5.779707191	7.76E-08	5.09E-07	7.462478818
								BAMBI	-0.762201793	7.682383726	-5.775729421	7.91E-08	5.17E-07	7.44505012
								BCL2A1	0.84948464	6.662470284	5.771673228	8.05E-08	5.27E-07	7.427284141
								C14orf34	0.654290585	4.27007298	5.765757186	8.27E-08	5.39E-07	7.401383582
								SCIN	0.644492364	4.721665831	5.764666639	8.31E-08	5.42E-07	7.396610632
								XK	-0.702013716	5.126587394	-5.76119961	8.44E-08	5.50E-07	7.381439725
								CCL13	0.985385553	5.870150777	5.757209792	8.59E-08	5.59E-07	7.363987026
								C1orf38	0.743833098	6.536536379	5.750215092	8.87E-08	5.75E-07	7.33340508
								GALNTL1	-0.725525811	6.136310585	-5.746905023	9.00E-08	5.83E-07	7.318939615
								ACOT4	0.67514914	5.225419265	5.746048221	9.03E-08	5.85E-07	7.315195968
								POTEF	-0.589252748	10.30468189	-5.738300039	9.35E-08	6.04E-07	7.281354762
								TNN	0.512468966	4.264082409	5.733217627	9.57E-08	6.16E-07	7.259169491
								LOC100506304	-0.804699651	4.942705636	-5.731224904	9.66E-08	6.21E-07	7.25047382
								CD8A	0.555551354	5.573159107	5.73096022	9.67E-08	6.22E-07	7.249318936
								LOC100506115	0.682128639	4.573742555	5.723496282	1.00E-07	6.41E-07	7.216763175
								DMPK	-0.62255118	7.981990661	-5.718193877	1.02E-07	6.55E-07	7.193648847
								XLOC_000978	0.584754881	4.810270672	5.715088573	1.04E-07	6.64E-07	7.180117329
								SIGLEC7	0.718922442	4.924749667	5.710142514	1.06E-07	6.77E-07	7.158572534
								XLOC_001855	0.682746325	4.300113569	5.703468304	1.09E-07	6.96E-07	7.129515405
								XLOC_001228	0.594568666	4.358178291	5.701136935	1.11E-07	7.03E-07	7.119369632
								ADH1B	-0.658164779	5.957055964	-5.699969731	1.11E-07	7.06E-07	7.114290951
								LOC283454	0.757762514	4.62608584	5.688816269	1.17E-07	7.39E-07	7.06578795

								CYP27B1	*0.648340111*	*4.480871592*	*5.685310372*	*1.19E-07*	*7.49E-07*	*7.050552149*
								KRT78	*-0.50577555*	*5.071061707*	*-5.680683839*	*1.21E-07*	*7.63E-07*	*7.03045387*
								LOC400456	*-0.574598161*	*5.262255619*	*-5.67754914*	*1.23E-07*	*7.73E-07*	*7.016841198*
								APCDD1	*-0.65582397*	*5.904019658*	*-5.672876168*	*1.25E-07*	*7.88E-07*	*6.996555782*
								POSTN	*0.74270916*	*8.853618784*	*5.668347984*	*1.28E-07*	*8.03E-07*	*6.976907275*
								CXCR2P1	*0.535105178*	*4.394043224*	*5.666626522*	*1.29E-07*	*8.08E-07*	*6.969439746*
								CAPN3	*0.520275037*	*6.161417629*	*5.662402359*	*1.31E-07*	*8.22E-07*	*6.951120813*
								IGANRP	*0.599290487*	*5.287018537*	*5.658089581*	*1.34E-07*	*8.36E-07*	*6.932425017*
								VCL	*-0.540992381*	*8.747417861*	*-5.653262246*	*1.37E-07*	*8.53E-07*	*6.911507543*
								C1QTNF7	*-0.696933332*	*5.435340414*	*-5.650885279*	*1.38E-07*	*8.61E-07*	*6.901211298*
								C17orf87	*0.515590499*	*4.829117772*	*5.637743392*	*1.47E-07*	*9.09E-07*	*6.844326316*
								HRK	*0.95789743*	*5.190592404*	*5.633167139*	*1.50E-07*	*9.27E-07*	*6.824534375*
								CTSL2	*0.526506819*	*4.391849329*	*5.627350902*	*1.54E-07*	*9.50E-07*	*6.799391916*
								CNN1	*-1.207258501*	*10.6818366*	*-5.619554474*	*1.59E-07*	*9.82E-07*	*6.765711148*
								COL3A1	*0.614489017*	*8.002461728*	*5.618614093*	*1.60E-07*	*9.85E-07*	*6.761650358*
								KAT2A	*0.542005361*	*7.380576452*	*5.616601559*	*1.61E-07*	*9.93E-07*	*6.752960975*
								AR	*-0.59144296*	*6.66688895*	*-5.615091946*	*1.62E-07*	*9.99E-07*	*6.746444109*
								MMP19	*0.800507327*	*7.129905672*	*5.612161949*	*1.64E-07*	*1.01E-06*	*6.733798234*
								LAMB3	*0.535673774*	*5.967776063*	*5.600098129*	*1.73E-07*	*1.06E-06*	*6.681767904*
								SBSN	*0.700546635*	*4.507550878*	*5.593841752*	*1.78E-07*	*1.09E-06*	*6.654808224*
								NGFR	*-0.851594794*	*5.301074234*	*-5.58982882*	*1.81E-07*	*1.11E-06*	*6.637524389*
								NPNT	*-0.856712777*	*7.675217121*	*-5.585823863*	*1.85E-07*	*1.13E-06*	*6.620281544*

								BCL6B	*0.759903893*	*6.080836875*	*5.581559991*	*1.88E-07*	*1.14E-06*	*6.601931264*
								ADM	*-0.822529112*	*8.419628836*	*-5.562611257*	*2.05E-07*	*1.24E-06*	*6.520473523*
								TPSAB1	*0.828330029*	*6.120664049*	*5.558539898*	*2.08E-07*	*1.26E-06*	*6.502990873*
								DIRAS1	*0.738338408*	*5.663806416*	*5.556701872*	*2.10E-07*	*1.27E-06*	*6.49510055*
								FAM107A	*-0.739739471*	*6.23837943*	*-5.548332803*	*2.18E-07*	*1.31E-06*	*6.459191458*
								GPNMB	*0.689305545*	*6.949240693*	*5.548072038*	*2.18E-07*	*1.31E-06*	*6.458073067*
								SNHG5	*-0.529573852*	*8.963279111*	*-5.542249615*	*2.24E-07*	*1.35E-06*	*6.433108784*
								OGN	*-0.935220855*	*8.523744147*	*-5.525689108*	*2.41E-07*	*1.44E-06*	*6.362181635*
								SNORA54	*-0.649913468*	*8.973035582*	*-5.517141812*	*2.50E-07*	*1.49E-06*	*6.325619568*
								SEMA3B	*-0.670995224*	*7.386528641*	*-5.511936874*	*2.56E-07*	*1.52E-06*	*6.303369966*
								HSD11B1	*-0.85636489*	*6.248394819*	*-5.51144266*	*2.57E-07*	*1.52E-06*	*6.301257939*
								XLOC_011344	*-0.711205412*	*5.262259039*	*-5.509978945*	*2.58E-07*	*1.53E-06*	*6.295003355*
								ID4	*-0.616289692*	*8.239970393*	*-5.506653465*	*2.62E-07*	*1.55E-06*	*6.28079666*
								HPGDS	*0.68189295*	*5.947294588*	*5.496016482*	*2.75E-07*	*1.62E-06*	*6.235386224*
								XLOC_000578	*-0.516135525*	*4.383752124*	*-5.488624178*	*2.84E-07*	*1.67E-06*	*6.203856044*
								C5AR1	*0.870072994*	*6.984614075*	*5.483684862*	*2.90E-07*	*1.70E-06*	*6.182801518*
								SFN	*-0.508299042*	*5.099911835*	*-5.481603477*	*2.93E-07*	*1.72E-06*	*6.173932447*
								CYP4X1	*-0.562481836*	*6.355117382*	*-5.477465878*	*2.98E-07*	*1.75E-06*	*6.156307063*
								XLOC_010931	*-0.521839636*	*4.390357223*	*-5.471376246*	*3.06E-07*	*1.79E-06*	*6.130379731*
								ACTBL2	*-0.808408535*	*8.683312788*	*-5.469241048*	*3.09E-07*	*1.80E-06*	*6.121292635*
								XLOC_009685	*-0.677085326*	*5.447757585*	*-5.46883979*	*3.09E-07*	*1.81E-06*	*6.119585161*
								LOC100130811	*0.532610436*	*4.426236677*	*5.462296395*	*3.18E-07*	*1.85E-06*	*6.091750747*

								RTN1	-0.572217192	5.849024944	-5.454762278	3.29E-07	1.91E-06	6.05972481
								XLOC_l2_007731	0.549655718	4.365039506	5.453302386	3.31E-07	1.92E-06	6.053521944
								MDK	0.540513758	6.773794343	5.451095752	3.34E-07	1.94E-06	6.044148036
								GPR171	0.636780104	5.56503431	5.44938388	3.37E-07	1.96E-06	6.03687735
								SLC16A6	0.691113046	5.107904762	5.449106018	3.37E-07	1.96E-06	6.035697332
								RN5-8S1	-0.943619332	11.01595741	-5.448865533	3.38E-07	1.96E-06	6.034676073
								OMD	-0.726795311	6.257658688	-5.448243044	3.39E-07	1.96E-06	6.032032682
								FOXS1	-0.683108219	8.109218883	-5.448017712	3.39E-07	1.97E-06	6.031075855
								HTRA4	1.208323497	5.437678882	5.444293289	3.45E-07	2.00E-06	6.015264011
								CDCP1	0.551320381	4.852787376	5.44363709	3.46E-07	2.00E-06	6.012478774
								FHL5	-1.315719519	6.503546605	-5.442893583	3.47E-07	2.01E-06	6.009323186
								TM4SF18	0.549185088	5.082828927	5.437884705	3.54E-07	2.05E-06	5.988070763
								MYOZ2	-0.550438212	4.386822851	-5.436934709	3.56E-07	2.05E-06	5.984041207
								GPRASP1	-0.5775647	6.945025603	-5.436757043	3.56E-07	2.06E-06	5.983287649
								IL10	0.602491531	4.892520529	5.436601382	3.56E-07	2.06E-06	5.982627437
								ME2	0.51723469	6.68942976	5.436127543	3.57E-07	2.06E-06	5.980617791
								PCDH7	-0.572310097	6.267364872	-5.430535536	3.66E-07	2.11E-06	5.956908302
								GJA4	-0.717013774	6.998279254	-5.427274	3.71E-07	2.14E-06	5.943086024
								HSPB6	-0.672241289	6.088803144	-5.427071603	3.72E-07	2.14E-06	5.942228424
								RELT	0.631915596	7.221709817	5.417858548	3.87E-07	2.21E-06	5.903209645
								HRCT1	-0.787072661	6.340170763	-5.417774194	3.87E-07	2.22E-06	5.902852563
								ASB16	0.585994908	5.382315048	5.411283733	3.98E-07	2.27E-06	5.87538688

								BMP2	*-0.572479206*	*5.624667547*	*-5.411163463*	*3.98E-07*	*2.27E-06*	*5.874878108*
								PKIB	*0.502709802*	*4.671119908*	*5.398115913*	*4.22E-07*	*2.40E-06*	*5.819721251*
								HIST1H1D	*-0.665172895*	*7.206437101*	*-5.396386368*	*4.25E-07*	*2.42E-06*	*5.812415421*
								CA12	*0.598278306*	*5.389783616*	*5.392835797*	*4.31E-07*	*2.45E-06*	*5.797421457*
								FLNA	*-0.602506283*	*7.354215332*	*-5.390045908*	*4.37E-07*	*2.48E-06*	*5.78564372*
								LOC100652988	*-0.530413806*	*5.247326391*	*-5.369517938*	*4.77E-07*	*2.70E-06*	*5.699088804*
								CLC	*0.819340084*	*4.445769187*	*5.367284829*	*4.82E-07*	*2.72E-06*	*5.689684277*
								CPE	*-0.658742336*	*9.528945167*	*-5.362336764*	*4.93E-07*	*2.78E-06*	*5.668853837*
								GAS1	*-0.684188434*	*6.586511553*	*-5.362005441*	*4.93E-07*	*2.78E-06*	*5.667459417*
								GRP	*0.596064188*	*4.435075174*	*5.359511642*	*4.99E-07*	*2.81E-06*	*5.656965468*
								ANXA3	*-0.57616552*	*5.170542386*	*-5.357970168*	*5.02E-07*	*2.82E-06*	*5.650480303*
								FTHL17	*0.509267214*	*9.656879432*	*5.351271226*	*5.17E-07*	*2.90E-06*	*5.622309321*
								IFI27	*0.719039316*	*8.511465357*	*5.348405517*	*5.23E-07*	*2.93E-06*	*5.61026429*
								CXCR6	*0.59836283*	*4.836269964*	*5.344113508*	*5.33E-07*	*2.98E-06*	*5.592231142*
								GNAI1	*-0.654020336*	*5.559441718*	*-5.334691527*	*5.55E-07*	*3.10E-06*	*5.552672946*
								KRT18	*0.733393447*	*7.073239377*	*5.330128887*	*5.66E-07*	*3.15E-06*	*5.533530973*
								XLOC_010933	*-0.504954096*	*5.375477412*	*-5.322681549*	*5.85E-07*	*3.25E-06*	*5.502306671*
								MCOLN2	*0.569605624*	*5.077700465*	*5.321505384*	*5.88E-07*	*3.26E-06*	*5.497377667*
								GCHFR	*0.786196105*	*6.617148994*	*5.320340999*	*5.91E-07*	*3.28E-06*	*5.492498639*
								XLOC_009932	*-0.58190771*	*4.423906774*	*-5.316662263*	*6.00E-07*	*3.33E-06*	*5.477087942*
								DNAJB4	*-0.51793294*	*6.462650988*	*-5.311466998*	*6.14E-07*	*3.40E-06*	*5.455334688*
								MYOM2	*-0.54030023*	*4.841440808*	*-5.31107398*	*6.15E-07*	*3.40E-06*	*5.453689564*

								SLC11A1	*0.622776274*	*5.224897276*	*5.306931158*	*6.26E-07*	*3.46E-06*	*5.436352477*
								GPR68	*0.75016288*	*6.680948184*	*5.304661385*	*6.32E-07*	*3.49E-06*	*5.426857108*
								FLVCR2	*0.524057408*	*4.49366989*	*5.30460281*	*6.33E-07*	*3.49E-06*	*5.426612096*
								MARCO	*1.612444431*	*7.885609*	*5.297216507*	*6.53E-07*	*3.59E-06*	*5.395728533*
								AKR1B1	*0.556155386*	*7.591396657*	*5.286874162*	*6.83E-07*	*3.74E-06*	*5.352526713*
								NPY1R	*-0.86039615*	*5.564728559*	*-5.285950109*	*6.86E-07*	*3.75E-06*	*5.348669143*
								GADD45G	*-0.717116212*	*6.930629918*	*-5.278379558*	*7.08E-07*	*3.86E-06*	*5.317079569*
								EPB41L3	*0.540709731*	*6.771510975*	*5.277292141*	*7.12E-07*	*3.88E-06*	*5.312544254*
								ATP1B1	*0.580099729*	*7.782158715*	*5.275258963*	*7.18E-07*	*3.91E-06*	*5.304065882*
								SMPDL3A	*0.828077095*	*5.993323916*	*5.273650325*	*7.23E-07*	*3.94E-06*	*5.29735918*
								COLEC12	*0.576777796*	*7.112779753*	*5.270834277*	*7.32E-07*	*3.98E-06*	*5.285621406*
								NTRK3	*-0.67391102*	*6.588406032*	*-5.267102375*	*7.44E-07*	*4.04E-06*	*5.270071775*
								RTN4R	*0.699130401*	*5.215283658*	*5.264829068*	*7.51E-07*	*4.08E-06*	*5.260602756*
								PALM	*-0.837686772*	*7.547680235*	*-5.264218483*	*7.53E-07*	*4.08E-06*	*5.258059885*
								HMGA1	*0.608382582*	*6.25921508*	*5.260108319*	*7.66E-07*	*4.15E-06*	*5.240946944*
								GNG13	*-0.600695055*	*4.752304231*	*-5.258694519*	*7.71E-07*	*4.17E-06*	*5.235062279*
								MLC1	*0.59782263*	*4.800916045*	*5.246915782*	*8.11E-07*	*4.37E-06*	*5.186071237*
								XLOC_004924	*0.543716133*	*4.560064236*	*5.239290669*	*8.38E-07*	*4.51E-06*	*5.154390263*
								CTSL1P8	*0.817492441*	*7.708117926*	*5.234899607*	*8.54E-07*	*4.58E-06*	*5.136158339*
								NPC1	*0.513894381*	*6.246055778*	*5.234126017*	*8.57E-07*	*4.60E-06*	*5.132947275*
								RCAN2	*-0.770281563*	*9.158731498*	*-5.233180133*	*8.60E-07*	*4.61E-06*	*5.129021417*
								C15orf27	*0.599090848*	*4.37315377*	*5.230724332*	*8.69E-07*	*4.66E-06*	*5.118830626*

								PDE1C	-0.629976711	4.699154394	-5.227287378	8.82E-07	4.72E-06	5.104573043
								PRICKLE1	-0.617330071	5.782143668	-5.222712676	9.00E-07	4.81E-06	5.085604192
								GSTM5	-0.80682511	6.354768256	-5.215264842	9.29E-07	4.95E-06	5.054742733
								SAA1	-1.407464782	6.298814066	-5.21498768	9.30E-07	4.96E-06	5.053594757
								LRRC55	-0.607531622	4.484412267	-5.211558056	9.44E-07	5.03E-06	5.039392573
								METTL7B	0.567128073	4.52233315	5.208573391	9.56E-07	5.08E-06	5.027037428
								HEY2	-0.741864158	6.197029586	-5.204091949	9.74E-07	5.18E-06	5.008494093
								CLEC10A	0.576367696	6.222484307	5.202558568	9.81E-07	5.21E-06	5.002151405
								IGFBP1	0.777983723	4.495459191	5.202179328	9.82E-07	5.21E-06	5.000582884
								PDE5A	-0.660702921	9.226872503	-5.20129844	9.86E-07	5.23E-06	4.996939823
								XLOC_009549	-0.593343542	5.156423358	-5.19988368	9.92E-07	5.26E-06	4.991089604
								BHLHE40	-0.563117964	7.27775813	-5.19697687	1.00E-06	5.32E-06	4.979072489
								LOC645638	0.856658789	5.382953314	5.184118453	1.06E-06	5.59E-06	4.925961521
								STEAP3	0.530316012	5.648580198	5.182252941	1.07E-06	5.64E-06	4.918262554
								LOC286058	-0.633101971	4.481619219	-5.176325893	1.10E-06	5.77E-06	4.893812456
								HIST1H2AD	-0.512932167	7.650384215	-5.174868171	1.10E-06	5.80E-06	4.887801624
								ITGB2	0.963794422	7.23266121	5.173246147	1.11E-06	5.84E-06	4.881114476
								NTN1	-0.852667728	7.543808996	-5.170370563	1.13E-06	5.91E-06	4.869262294
								ALK	0.567002507	4.163905788	5.151334575	1.22E-06	6.36E-06	4.790900627
								SNAP25	-0.6062769	5.839227534	-5.149806938	1.23E-06	6.40E-06	4.78461952
								F13A1	0.623822237	7.151463236	5.12591985	1.36E-06	7.03E-06	4.686548139
								RERG	-0.727958747	6.838110729	-5.117119051	1.41E-06	7.28E-06	4.650483797

								SAMSN1	*0.645488387*	*6.071360097*	*5.105969867*	*1.48E-06*	*7.61E-06*	*4.604849258*
								BCHE	*-0.629948329*	*4.62359315*	*-5.092319912*	*1.57E-06*	*8.03E-06*	*4.54905997*
								NFATC4	*-0.59806727*	*6.148378683*	*-5.083525265*	*1.63E-06*	*8.31E-06*	*4.513162462*
								DNAJC5B	*0.57870248*	*4.419277496*	*5.082952627*	*1.63E-06*	*8.33E-06*	*4.510826394*
								CSRNP1	*-0.729967148*	*5.779969454*	*-5.073556179*	*1.70E-06*	*8.63E-06*	*4.472516306*
								IGF1	*-0.84240453*	*6.620056363*	*-5.073008318*	*1.70E-06*	*8.65E-06*	*4.470283952*
								LINC00303	*-0.513149346*	*4.290319846*	*-5.071739305*	*1.71E-06*	*8.69E-06*	*4.465113688*
								KRT16	*-0.672237203*	*5.271787701*	*-5.070446822*	*1.72E-06*	*8.73E-06*	*4.459848602*
								PTGFR	*-0.751697349*	*5.381590121*	*-5.068658808*	*1.73E-06*	*8.79E-06*	*4.452566248*
								GALNTL2	*-0.857766638*	*6.374307117*	*-5.068637041*	*1.73E-06*	*8.79E-06*	*4.452477601*
								LOC100505976	*-0.559344*	*5.370725606*	*-5.0537055*	*1.84E-06*	*9.31E-06*	*4.39172401*
								MCAM	*-0.856623784*	*9.543119368*	*-5.05332319*	*1.85E-06*	*9.32E-06*	*4.390169884*
								LOC645722	*-0.61748802*	*6.860532926*	*-5.048704275*	*1.88E-06*	*9.49E-06*	*4.3713992*
								RASD1	*-1.078650291*	*7.139060828*	*-5.043993035*	*1.92E-06*	*9.68E-06*	*4.352264025*
								CMTM2	*0.508585434*	*4.272388943*	*5.037982033*	*1.97E-06*	*9.90E-06*	*4.327865456*
								SGCA	*-0.652587775*	*8.741995227*	*-5.025418881*	*2.08E-06*	*1.04E-05*	*4.276928855*
								MT1F	*0.583659292*	*6.717636686*	*5.023347062*	*2.10E-06*	*1.05E-05*	*4.268536208*
								ADARB1	*-0.522390673*	*6.966581726*	*-5.013915015*	*2.18E-06*	*1.09E-05*	*4.230354942*
								KANK2	*-0.527996732*	*9.768053982*	*-5.013583665*	*2.18E-06*	*1.09E-05*	*4.229014421*
								CPA3	*0.688531985*	*5.906516512*	*5.011317373*	*2.20E-06*	*1.10E-05*	*4.219847277*
								CR1	*0.503661857*	*4.696506856*	*5.008845653*	*2.23E-06*	*1.11E-05*	*4.209852061*
								THBS2	*0.507961817*	*8.684729329*	*5.006762612*	*2.25E-06*	*1.12E-05*	*4.201430935*

								GAL	*0.508786269*	*4.71169044*	*4.996802689*	*2.34E-06*	*1.16E-05*	*4.161195457*
								TNFSF13	*0.563642964*	*6.388400298*	*4.989909861*	*2.41E-06*	*1.19E-05*	*4.133378945*
								FCER1A	*0.55097933*	*5.713131692*	*4.951703226*	*2.83E-06*	*1.38E-05*	*3.979621294*
								GDF10	*-0.913705936*	*6.230792322*	*-4.951619521*	*2.83E-06*	*1.38E-05*	*3.979285234*
								PDZRN4	*-0.989662698*	*5.891116383*	*-4.948785998*	*2.86E-06*	*1.40E-05*	*3.967911224*
								ADAM28	*0.555578259*	*5.331981084*	*4.945246962*	*2.90E-06*	*1.42E-05*	*3.95371087*
								CSF3	*-0.972433636*	*4.450128317*	*-4.944568612*	*2.91E-06*	*1.42E-05*	*3.950989714*
								RARRES1	*-0.880676988*	*7.263689107*	*-4.939117556*	*2.98E-06*	*1.45E-05*	*3.92913155*
								ACTG2	*-1.044742892*	*8.285448811*	*-4.937803344*	*3.00E-06*	*1.46E-05*	*3.923863927*
								PLEKHG3	*-0.511123864*	*8.369724258*	*-4.933395182*	*3.05E-06*	*1.48E-05*	*3.906201491*
								HPSE	*0.601851493*	*4.892637639*	*4.918959373*	*3.24E-06*	*1.56E-05*	*3.84842921*
								AGRP	*0.575428709*	*4.649533834*	*4.9132151*	*3.32E-06*	*1.60E-05*	*3.825469825*
								CCL7	*0.722194431*	*4.761489517*	*4.903836957*	*3.45E-06*	*1.65E-05*	*3.788022061*
								KCNK5	*-0.587669747*	*5.641235536*	*-4.897888842*	*3.53E-06*	*1.69E-05*	*3.764293828*
								EGFL6	*0.649014577*	*4.560687081*	*4.893579359*	*3.60E-06*	*1.72E-05*	*3.747113662*
								WFDC1	*-0.799789496*	*7.031979403*	*-4.89349579*	*3.60E-06*	*1.72E-05*	*3.746780601*
								CLDN11	*-0.642997273*	*5.185306781*	*-4.893070851*	*3.61E-06*	*1.72E-05*	*3.745087071*
								MFGE8	*-0.529181471*	*8.339113316*	*-4.877860662*	*3.84E-06*	*1.83E-05*	*3.684529758*
								MYH11	*-0.889696199*	*11.19224787*	*-4.864222227*	*4.06E-06*	*1.92E-05*	*3.630330697*
								VAT1L	*-0.557397842*	*5.164158042*	*-4.860305361*	*4.13E-06*	*1.95E-05*	*3.614782717*
								TIMP1	*0.541752215*	*9.932717933*	*4.852019055*	*4.27E-06*	*2.01E-05*	*3.581916244*
								SRPX	*-0.65144489*	*7.124798452*	*-4.847022338*	*4.36E-06*	*2.05E-05*	*3.562114544*

								JUNB	-0.644935374	7.008494123	-4.844022042	4.41E-06	2.07E-05	3.550230726
								COL4A4	-0.623454551	6.392356366	-4.836052676	4.56E-06	2.14E-05	3.518687564
								LOC401847	0.584093776	4.560816738	4.834170951	4.60E-06	2.15E-05	3.511244391
								DDIT4	-0.679099697	8.266176242	-4.811600242	5.04E-06	2.34E-05	3.42210882
								PNPLA3	0.591116499	4.808460413	4.798097942	5.33E-06	2.47E-05	3.368912581
								LOC100506393	-0.541548106	5.257630829	-4.791749373	5.47E-06	2.53E-05	3.343933441
								CXCL9	0.799383087	6.245282798	4.791503228	5.47E-06	2.53E-05	3.342965381
								PKD2L1	0.951652478	5.278895635	4.786485493	5.59E-06	2.58E-05	3.323238121
								CHIT1	0.70559925	4.757767795	4.782977906	5.67E-06	2.61E-05	3.309455849
								EBI3	0.553339439	5.397429284	4.779725019	5.74E-06	2.64E-05	3.296680134
								MAOA	-0.627227631	7.603165896	-4.774137934	5.88E-06	2.70E-05	3.274749818
								ABRA	-0.795153177	4.783352199	-4.749803682	6.49E-06	2.95E-05	3.179425221
								CLMP	-0.568377975	6.909879672	-4.74144136	6.71E-06	3.05E-05	3.14673973
								SRGN	0.545100333	8.798830806	4.736316054	6.85E-06	3.11E-05	3.126724966
								PTPRO	0.561141555	5.453969291	4.73616409	6.86E-06	3.11E-05	3.126131749
								KCNJ3	-0.609659326	4.985037737	-4.732723182	6.96E-06	3.15E-05	3.112702811
								ACTA1	-1.186375928	5.316599705	-4.712937606	7.54E-06	3.39E-05	3.035607363
								SNORD66	-0.610200146	6.375937891	-4.703506777	7.83E-06	3.51E-05	2.998933247
								KRT31	0.570803482	4.915116288	4.702904902	7.85E-06	3.52E-05	2.996594322
								NAMPT	-0.60168712	7.840942252	-4.695437498	8.09E-06	3.62E-05	2.967591678
								ITGA8	-0.752473491	8.738607192	-4.678110085	8.67E-06	3.86E-05	2.900409198
								HBQ1	0.835986066	5.964377001	4.676713807	8.72E-06	3.88E-05	2.895002537

								PPP1R3C	-0.649094775	8.443248303	-4.63776691	1.02E-05	4.48E-05	2.744617915
								APOC2	0.959629702	5.029831559	4.633965724	1.04E-05	4.54E-05	2.729984652
								PER1	-0.527484917	7.483610282	-4.62563483	1.07E-05	4.68E-05	2.697941153
								MIR143HG	-0.676311119	7.492436782	-4.617324651	1.11E-05	4.82E-05	2.666015145
								FOSL2	-0.581699761	7.357731861	-4.616161102	1.11E-05	4.84E-05	2.661548046
								DNASE2B	0.619586472	4.395114721	4.613121562	1.13E-05	4.90E-05	2.649882145
								FST	0.511241222	6.825934252	4.605880412	1.16E-05	5.03E-05	2.622110686
								ADH1A	-0.599427373	7.243552692	-4.595500579	1.21E-05	5.23E-05	2.582351952
								ZBTB16	-0.763435481	7.483681862	-4.594262949	1.21E-05	5.25E-05	2.577615315
								WTIP	-0.539635772	8.364395818	-4.590006246	1.23E-05	5.34E-05	2.561330581
								HLF	-0.701150546	6.142156686	-4.539678054	1.51E-05	6.41E-05	2.369552761
								GPR20	-0.791774566	6.558380662	-4.532958219	1.55E-05	6.58E-05	2.344053306
								XLOC_004049	-0.673277691	5.041427755	-4.502144962	1.75E-05	7.38E-05	2.227452198
								C12orf75	-0.617526237	8.247754621	-4.489242786	1.84E-05	7.72E-05	2.178787759
								SHANK3	0.652489803	6.962404669	4.480523856	1.90E-05	7.97E-05	2.145955059
								DIO3OS	-0.529693847	4.381593339	-4.476363748	1.94E-05	8.09E-05	2.130304622
								LOC100509105	-0.60000192	7.040792431	-4.468471854	2.00E-05	8.31E-05	2.100642136
								ITGA7	-0.675413036	9.67859014	-4.467598557	2.00E-05	8.34E-05	2.09736194
								GFPT2	-0.583182149	6.852132273	-4.466856021	2.01E-05	8.36E-05	2.094573237
								NFE2	0.674085345	5.269122121	4.459039706	2.07E-05	8.59E-05	2.065236975
								C10orf10	-0.689677638	8.947488377	-4.458557429	2.08E-05	8.60E-05	2.063428032
								SNORD38A	-0.690801066	7.060261029	-4.457575715	2.08E-05	8.64E-05	2.059746187

								MGP	*-0.549729807*	*11.56468109*	*-4.456061468*	*2.10E-05*	*8.68E-05*	*2.054068198*
								SPOCD1	*0.787357909*	*6.729869996*	*4.44856158*	*2.16E-05*	*8.92E-05*	*2.025965095*
								MFSD2A	*0.527249051*	*4.754972787*	*4.422620992*	*2.39E-05*	*9.78E-05*	*1.929010741*
								KRT18P55	*0.608077788*	*6.614024164*	*4.420015368*	*2.41E-05*	*9.87E-05*	*1.919293459*
								SCD	*0.76379103*	*6.211512965*	*4.418408909*	*2.43E-05*	*9.93E-05*	*1.913304359*
								SULT1C2	*0.514816356*	*4.376954062*	*4.416248766*	*2.45E-05*	*1.00E-04*	*1.905253396*
								CXCL5	*0.759895429*	*5.659274708*	*4.416036337*	*2.45E-05*	*0.000100054*	*1.904461808*
								C4B	*0.762723895*	*8.00232617*	*4.409836533*	*2.51E-05*	*0.000102239*	*1.881370542*
								C19orf59	*0.788978007*	*5.264023725*	*4.398842483*	*2.62E-05*	*0.000106239*	*1.840477655*
								COL4A5	*-0.578075735*	*6.876661737*	*-4.361345983*	*3.03E-05*	*0.000121427*	*1.70153549*
								CXCL1	*0.771261693*	*5.922282064*	*4.352521027*	*3.14E-05*	*0.000125311*	*1.66895411*
								SLC16A10	*0.517126641*	*4.914131817*	*4.347532413*	*3.20E-05*	*0.000127514*	*1.650556532*
								SDS	*1.084263877*	*6.238942895*	*4.342246182*	*3.26E-05*	*0.000129972*	*1.631077302*
								IL2RA	*0.557915237*	*4.964117546*	*4.314861486*	*3.63E-05*	*0.000143112*	*1.530430719*
								P2RY6	*0.504225808*	*6.605521295*	*4.314817337*	*3.63E-05*	*0.000143118*	*1.530268815*
								RNF122	*-0.566996386*	*7.249021549*	*-4.312018162*	*3.67E-05*	*0.000144553*	*1.520006054*
								KRT1	*0.508514338*	*4.579082939*	*4.278988437*	*4.16E-05*	*0.00016225*	*1.399258473*
								SHISA3	*0.746694088*	*6.545343369*	*4.278880596*	*4.16E-05*	*0.000162296*	*1.398865299*
								HAMP	*0.947186791*	*6.24703254*	*4.265897087*	*4.37E-05*	*0.000170023*	*1.351579904*
								SAA2	*-0.655827166*	*4.7104979*	*-4.228854886*	*5.04E-05*	*0.000193682*	*1.217229286*
								MYRIP	*-0.650806992*	*5.578295667*	*-4.22322695*	*5.14E-05*	*0.000197339*	*1.196889294*
								XLOC_009994	*-0.505558644*	*4.752874079*	*-4.215110644*	*5.30E-05*	*0.000202966*	*1.167589778*

								FAM46B	*-0.610074801*	*6.071769305*	*-4.197816899*	*5.66E-05*	*0.000215629*	*1.105293289*
								MUSTN1	*-1.077703097*	*7.974346266*	*-4.160145295*	*6.53E-05*	*0.000245439*	*0.970221225*
								SNORD67	*-0.533680691*	*6.096322333*	*-4.139544356*	*7.05E-05*	*0.000263689*	*0.896724089*
								HSPB3	*-0.748067933*	*5.612577568*	*-4.127417827*	*7.38E-05*	*0.000274914*	*0.853583031*
								CKB	*-0.824119444*	*6.915293197*	*-4.094813401*	*8.33E-05*	*0.000307107*	*0.738041871*
								DNASE1L3	*0.646024043*	*6.222914992*	*4.078051923*	*8.87E-05*	*0.000325065*	*0.678901211*
								NDUFA4L2	*0.557971091*	*7.222337104*	*4.076235891*	*8.93E-05*	*0.000327134*	*0.672504119*
								PF4	*0.817355435*	*5.445226276*	*4.075639468*	*8.95E-05*	*0.000327729*	*0.670403633*
								PITX1	*-0.661604698*	*5.151220573*	*-4.053114225*	*9.72E-05*	*0.000353666*	*0.59123721*
								TACSTD2	*-0.501672587*	*5.382527861*	*-4.034636008*	*0.000104099*	*0.000376539*	*0.526532526*
								CYTL1	*-0.533601649*	*6.405065638*	*-4.022466832*	*0.000108866*	*0.000391928*	*0.48403777*
								PTX3	*-0.818711744*	*6.423547213*	*-4.012772235*	*0.000112813*	*0.000404517*	*0.450251352*
								XLOC_009167	*-0.508928041*	*5.402507374*	*-4.002448624*	*0.000117167*	*0.000418703*	*0.414338365*
								TPPP3	*0.606312457*	*6.627216013*	*3.996565519*	*0.000119719*	*0.000427124*	*0.393902991*
								ITLN1	*-1.439620649*	*7.867261214*	*-3.993377841*	*0.000121124*	*0.000431432*	*0.382839579*
								C1QTNF4	*0.584839386*	*5.242306103*	*3.98132274*	*0.000126581*	*0.000448882*	*0.341058781*
								GPIHBP1	*0.688000329*	*6.782414042*	*3.97009282*	*0.000131876*	*0.00046636*	*0.302221488*
								LOC100652913	*-0.537517038*	*7.701636463*	*-3.945815545*	*0.000144055*	*0.000506556*	*0.218538012*
								SRL	*-0.818327986*	*5.288241173*	*-3.940268149*	*0.000146985*	*0.000516206*	*0.199469434*
								ADH1C	*-0.580718199*	*8.778730577*	*-3.917985407*	*0.000159338*	*0.000555741*	*0.123075173*
								FPR1	*0.561378956*	*7.816434789*	*3.911317552*	*0.000163223*	*0.000568125*	*0.100277571*
								ADSSL1	*-0.530010668*	*7.740141035*	*-3.908631602*	*0.000164814*	*0.000572892*	*0.091102377*

								PLIN4	-0.883898293	5.574089952	-3.886458515	0.00017852	0.000616536	0.015538514
								PROK2	0.519601315	4.40854025	3.877529286	0.000184341	0.000634487	-0.014800926
								AVPR1A	-0.959693015	5.316518465	-3.86677706	0.00019159	0.000657366	-0.051265265
								WISP2	-0.685856528	10.21471762	-3.849021763	0.00020416	0.000697127	-0.111313314
								ABCG1	0.574052683	6.078930212	3.848806705	0.000204317	0.000697507	-0.112039367
								KCNJ8	-0.63945185	6.037490603	-3.845979679	0.000206391	0.000704116	-0.121580784
								GREM2	-0.719980002	5.02119291	-3.84115397	0.000209977	0.000715393	-0.137855735
								CFD	-0.578331437	10.27208443	-3.833875909	0.000215499	0.000732654	-0.162372328
								PPBP	0.777046814	5.109287726	3.82682363	0.000220982	0.000749625	-0.186094996
								C7	-0.90943514	6.961193479	-3.757397683	0.000282528	0.000939525	-0.417868576
								SLC1A3	0.552580499	5.627527902	3.742595433	0.000297609	0.000985279	-0.466868042
								IL8	0.618029496	4.808139753	3.729058131	0.000312068	0.001028473	-0.511551048
								HSPA1B	-0.540065323	8.056663021	-3.685889284	0.000362762	0.001179258	-0.653211704
								ADAMTS9	-0.587696936	6.996785617	-3.68396925	0.000365189	0.001185815	-0.659482983
								OLR1	0.602893148	5.089177414	3.657363327	0.000400446	0.001292819	-0.74612502
								XLOC_014002	0.593011939	4.644623771	3.620207323	0.000455114	0.001453946	-0.866310922
								LOC100509100	0.672602989	6.250205304	3.6199855	0.00045546	0.001454749	-0.867025581
								SNAR-B2	0.542653418	8.187584641	3.595331978	0.000495582	0.00157189	-0.946241091
								MFAP4	-0.552776906	10.68785803	-3.594237777	0.000497438	0.001577122	-0.949747162
								CNR1	-0.557716727	4.996316767	-3.580533847	0.000521247	0.001645096	-0.993587264
								KCNK17	-0.562220633	4.973100511	-3.56921009	0.000541729	0.001702879	-1.029714344
								BGLAP	0.560781837	6.040796127	3.558961599	0.00056092	0.00175832	-1.062333796

								HBG1	*0.857957072*	*5.919559639*	*3.526831528*	*0.000625332*	*0.001939736*	*-1.164122422*
								SOST	*-0.936485905*	*7.129300513*	*-3.517478411*	*0.000645357*	*0.001995886*	*-1.193616953*
								FAM70A	*0.507832369*	*4.570044364*	*3.501264574*	*0.00068151*	*0.002098603*	*-1.244600194*
								NR4A3	*-0.636621875*	*5.963111399*	*-3.487338716*	*0.000714077*	*0.002188356*	*-1.28824065*
								LTF	*0.527505568*	*4.886721636*	*3.447545805*	*0.000815408*	*0.002471135*	*-1.412183273*
								HLA-DRB6	*0.615381253*	*4.330726379*	*3.42182295*	*0.000887954*	*0.002669278*	*-1.491700324*
								RBP4	*-0.623770519*	*5.847286151*	*-3.395611565*	*0.00096809*	*0.002889704*	*-1.572238663*
								LYVE1	*-0.514428829*	*6.068984926*	*-3.372304862*	*0.001045004*	*0.003099307*	*-1.643435435*
								PGAM2	*-0.596478154*	*5.826595009*	*-3.350637112*	*0.001121622*	*0.003306322*	*-1.709272292*
								PDPN	*0.520529909*	*6.39514798*	*3.334942864*	*0.001180373*	*0.003464732*	*-1.756745436*
								DKFZP547L112	*-0.532471883*	*7.643448628*	*-3.31034473*	*0.001278288*	*0.003725823*	*-1.830789635*
								TNC	*0.501017263*	*8.11613366*	*3.30680917*	*0.001292971*	*0.003765987*	*-1.841395778*
								FOSB	*0.994801908*	*8.084836427*	*3.30347389*	*0.001306968*	*0.003800617*	*-1.851392703*
								SNAR-D	*0.514628543*	*7.816237914*	*3.270298758*	*0.001454116*	*0.004185105*	*-1.950383702*
								CD24	*0.50152684*	*5.856854636*	*3.268038146*	*0.001464687*	*0.004213544*	*-1.957099575*
								DPT	*-0.5951697*	*8.788958699*	*-3.25949297*	*0.001505297*	*0.004320199*	*-1.982451614*
								XLOC_013368	*-0.59151001*	*4.912471359*	*-3.216061453*	*0.001728463*	*0.004899022*	*-2.110467601*
								HBM	*0.690564867*	*5.334639158*	*3.193118236*	*0.001858458*	*0.005239281*	*-2.177525887*
								FRZB	*-0.519670949*	*8.454553335*	*-3.157282619*	*0.002079849*	*0.00579128*	*-2.28147592*
								MAPK4	*-0.609263609*	*5.206019635*	*-3.115493418*	*0.002368926*	*0.006522498*	*-2.401471733*
								XLOC_004956	*-0.84563387*	*5.977191614*	*-3.111642891*	*0.002397359*	*0.006590689*	*-2.412461715*
								G0S2	*-0.653224258*	*7.383776214*	*-3.097813577*	*0.002502113*	*0.006855234*	*-2.451839623*

								XLOC_011134	*-0.503633435*	*4.733129283*	*-3.037732409*	*0.003008403*	*0.008099995*	*-2.621218822*
								RPA4	*-0.783449595*	*7.180486756*	*-2.983820709*	*0.003541784*	*0.009384868*	*-2.770838907*
								ALOX15B	*0.548173563*	*5.589408912*	*2.977629475*	*0.00360833*	*0.009541231*	*-2.787877057*
								REP15	*-0.659526943*	*6.155196654*	*-2.968553535*	*0.00370797*	*0.009782479*	*-2.812799925*
								SELE	*-0.586662607*	*5.303565332*	*-2.936419982*	*0.004081489*	*0.01066044*	*-2.9005231*
								XLOC_004187	*-0.7029332*	*5.583372179*	*-2.927835031*	*0.004186985*	*0.010902432*	*-2.923822844*
								XLOC_012294	*-0.81326881*	*6.185157751*	*-2.878665223*	*0.00484108*	*0.01241413*	*-3.056153983*
								CACNA1H	*-0.562470535*	*7.054766618*	*-2.868909618*	*0.004981517*	*0.012738976*	*-3.08218242*
								ISM1	*-0.583949378*	*6.098318786*	*-2.856457081*	*0.005166207*	*0.013171585*	*-3.115296823*
								XLOC_l2_007770	*-0.787321917*	*6.312252411*	*-2.831894877*	*0.00554902*	*0.014063101*	*-3.18025268*
								XLOC_009764	*-0.741863124*	*5.652847482*	*-2.827068906*	*0.005627221*	*0.014242387*	*-3.19295871*
								FOS	*0.58824364*	*9.83360518*	*2.806322602*	*0.005975014*	*0.015028035*	*-3.247368699*
								GPR182	*-0.780266574*	*6.19682795*	*-2.780336736*	*0.006438326*	*0.016053258*	*-3.315034021*
								XLOC_010855	*-0.804323103*	*6.096859162*	*-2.778143827*	*0.006478887*	*0.016143853*	*-3.320719395*
								XLOC_002486	*-0.538353089*	*4.872817575*	*-2.776084807*	*0.006517184*	*0.016230014*	*-3.326054129*
								CCDC66	*-0.640728661*	*5.940613279*	*-2.771153126*	*0.006609752*	*0.016432413*	*-3.338817803*
								XLOC_001339	*-0.589791056*	*5.177124378*	*-2.76495982*	*0.006727698*	*0.016699821*	*-3.354818952*
								XLOC_007020	*-0.807139538*	*6.174887399*	*-2.75098942*	*0.00700083*	*0.017294904*	*-3.390799644*
								KRT79	*-0.74354322*	*5.760993933*	*-2.745354774*	*0.007113822*	*0.017537174*	*-3.405267066*
								XLOC_009191	*-0.719972278*	*6.753711136*	*-2.719988934*	*0.007643331*	*0.018687232*	*-3.470077922*
								GPR179	*-0.790896465*	*6.570636902*	*-2.715591044*	*0.0077387*	*0.01889017*	*-3.481261688*
								S100A8	*0.571414258*	*8.639305194*	*2.644969545*	*0.009425667*	*0.022511673*	*-3.658693211*

								ALAS2	*0.608454556*	*5.878739358*	*2.633328056*	*0.009733716*	*0.023174937*	*-3.687549863*
								CRLF1	*-0.568773928*	*8.989238028*	*-2.615877497*	*0.010212547*	*0.024177448*	*-3.730597181*
								XLOC_004525	*-0.680139627*	*5.69458585*	*-2.611587583*	*0.01033347*	*0.024420205*	*-3.741141202*
								STAC2	*-0.597088428*	*6.105103767*	*-2.605946089*	*0.010494458*	*0.024770001*	*-3.754984117*
								PPP6R1	*-0.764315841*	*7.273295797*	*-2.595262753*	*0.010805536*	*0.025431548*	*-3.781126549*
								XLOC_l2_012388	*-0.755898658*	*7.712729228*	*-2.564738347*	*0.01174068*	*0.027414959*	*-3.855300224*
								LDLR	*-0.52608864*	*7.22290058*	*-2.53035103*	*0.012880877*	*0.029743223*	*-3.937933757*
								P2RX1	*-0.571436881*	*5.940305049*	*-2.522955381*	*0.013138717*	*0.030279104*	*-3.955576904*
								VPS18	*-0.612296797*	*9.606874567*	*-2.483722097*	*0.014585502*	*0.033237278*	*-4.048406979*
								XLOC_008559	*-0.697564037*	*8.632389033*	*-2.428660321*	*0.016856096*	*0.037813593*	*-4.176506452*
								CD86	*-0.707094167*	*9.008068621*	*-2.420053479*	*0.01723814*	*0.038579997*	*-4.196298545*
								XLOC_001699	*-0.697213359*	*8.976957999*	*-2.376575644*	*0.019288208*	*0.042547968*	*-4.295317956*
								LOC100509175	*0.52206097*	*4.787339675*	*2.34961255*	*0.020665585*	*0.045223914*	*-4.355916496*

Supplementary Table S2

KEGG analysis results of 566 DEGs in GSE135917 dataset												
Category	*Term*	*Count*	*%*	*PValue*	*Genes*	*List Total*	*Pop Hits*	*Pop Total*	*Fold Enrichment*	*Bonferroni*	*Benjamini*	*FDR*
KEGG_PATHWAY	*hsa04010: MAPK signaling pathway*	*17*	*3.250478011*	*0.021461088*	*ENSG00000175197, ENSG00000130522, ENSG00000154188, ENSG00000050748, ENSG00000198001, ENSG00000153956, ENSG00000066468, ENSG00000152689, ENSG00000177606, ENSG00000170345, ENSG00000136997,*	*257*	*294*	*8205*	*1.846065274*	*0.99784423*	*0.401298982*	*0.401298982*

					ENSG00000116473, ENSG00000120129, ENSG00000102678, ENSG00000109321, ENSG00000123358, ENSG00000127314							
KEGG_PATHWAY	hsa05171:Coronavirus disease - COVID-19	15	2.868068834	0.013697627	ENSG00000050748, ENSG00000198001, ENSG00000118181, ENSG00000134419, ENSG00000177606, ENSG00000136244, ENSG00000265681, ENSG00000170345, ENSG00000177954, ENSG00000170889, ENSG00000113070, ENSG00000169245, ENSG00000109475, ENSG00000162711, ENSG00000164587	257	232	8205	2.064185563	0.97982321	0.401298982	0.401298982
KEGG_PATHWAY	hsa05166:Human T-cell leukemia virus 1 infection	15	2.868068834	0.009552422	ENSG00000050748, ENSG00000146592, ENSG00000120738, ENSG00000177606, ENSG00000136244, ENSG00000196126, ENSG00000170345, ENSG00000137673, ENSG00000136997, ENSG00000139687, ENSG00000196735, ENSG00000118971, ENSG00000124762, ENSG00000128016, ENSG00000053900	257	222	8205	2.157166895	0.933883039	0.38619078	0.38619078
KEGG_PATHWAY	hsa05417:Lipid and atherosclerosis	14	2.676864245	0.016946588	ENSG00000175197, ENSG00000050748, ENSG00000198001, ENSG00000177606, ENSG00000136244, ENSG00000170345, ENSG00000172115, ENSG00000116473,	257	215	8205	2.078906886	0.992069082	0.401298982	0.401298982

					ENSG00000084674, ENSG00000162711, ENSG00000129988, ENSG00000081041, ENSG00000127314, ENSG00000140465							
KEGG_PATHWAY	hsa05167:Kaposi sarcoma-associated herpesvirus infection	14	2.676864245	0.007602451	ENSG00000073756, ENSG00000050748, ENSG00000110852, ENSG00000159200, ENSG00000177606, ENSG00000136244, ENSG00000170345, ENSG00000136997, ENSG00000139687, ENSG00000172115, ENSG00000140941, ENSG00000081041, ENSG00000124762, ENSG00000128016	257	194	8205	2.303943199	0.884641846	0.38619078	0.38619078
KEGG_PATHWAY	hsa04024:cAMP signaling pathway	13	2.485659656	0.043659801	ENSG00000106018, ENSG00000050748, ENSG00000146592, ENSG00000177606, ENSG00000170345, ENSG00000131771, ENSG00000112541, ENSG00000069849, ENSG00000116473, ENSG00000198829, ENSG00000125675, ENSG00000127314, ENSG00000164128	257	221	8205	1.87800412	0.999996739	0.588367797	0.588367797
KEGG_PATHWAY	hsa05169:Epstein-Barr virus infection	13	2.485659656	0.024106299	ENSG00000050748, ENSG00000198001, ENSG00000116815, ENSG00000177606, ENSG00000136244, ENSG00000196126, ENSG00000136997, ENSG00000139687, ENSG00000196735, ENSG00000172115, ENSG00000118971,	257	202	8205	2.054648072	0.998997899	0.401298982	0.401298982

					ENSG00000169245, ENSG00000124762							
KEGG_PATHWAY	hsa05130:Pathogenic Escherichia coli infection	13	2.485659656	0.020263414	ENSG00000101162, ENSG00000050748, ENSG00000198001, ENSG00000104833, ENSG00000177606, ENSG00000136244, ENSG00000170345, ENSG00000197822, ENSG00000172115, ENSG00000133392, ENSG00000162711, ENSG00000167306, ENSG00000163347	257	197	8205	2.1067965	0.996952531	0.401298982	0.401298982
KEGG_PATHWAY	hsa04514:Cell adhesion molecules	13	2.485659656	0.003627	ENSG00000116815, ENSG00000162706, ENSG00000196126, ENSG00000081237, ENSG00000261371, ENSG00000197822, ENSG00000196735, ENSG00000170017, ENSG00000165246, ENSG00000150637, ENSG00000091129, ENSG00000163531, ENSG00000163347	257	157	8205	2.64355994	0.642388096	0.38619078	0.38619078
KEGG_PATHWAY	hsa05161: Hepatitis B	11	2.103250478	0.030088642	ENSG00000050748, ENSG00000198001, ENSG00000179388, ENSG00000146592, ENSG00000136244, ENSG00000177606, ENSG00000170345, ENSG00000136997, ENSG00000139687, ENSG00000172115, ENSG00000124762	257	162	8205	2.167819571	0.999824139	0.473060321	0.473060321
KEGG_PATHWAY	hsa04932: Non-alcoholic	11	2.103250478	0.023026383	ENSG00000175197, ENSG00000065518, ENSG00000186010, ENSG00000050748, ENSG00000165264,	257	155	8205	2.2657211	0.998629615	0.401298982	0.401298982

	fatty liver disease				*ENSG00000136244, ENSG00000177606, ENSG00000170345, ENSG00000184557, ENSG00000125356, ENSG00000172115*							
KEGG_PATHWAY	*hsa04932: Non-alcoholic fatty liver disease*	*11*	*2.103250478*	*0.023026383*	*ENSG00000175197, ENSG00000065518, ENSG00000186010, ENSG00000050748, ENSG00000165264, ENSG00000136244, ENSG00000177606, ENSG00000170345, ENSG00000184557, ENSG00000125356, ENSG00000172115*	*257*	*155*	*8205*	*2.2657211*	*0.998629615*	*0.401298982*	*0.401298982*
KEGG_PATHWAY	*hsa04668:TNF signaling pathway*	*10*	*1.912045889*	*0.008158019*	*ENSG00000073756, ENSG00000050748, ENSG00000169245, ENSG00000146592, ENSG00000136244, ENSG00000177606, ENSG00000171223, ENSG00000170345, ENSG00000184557, ENSG00000081041*	*257*	*112*	*8205*	*2.850541968*	*0.901548206*	*0.38619078*	*0.38619078*
KEGG_PATHWAY	*hsa04620:Toll-like receptor signaling pathway*	*9*	*1.72084130*	*0.015751839*	*ENSG00000050748, ENSG00000198001, ENSG00000169245, ENSG00000138755, ENSG00000136244, ENSG00000129988, ENSG00000177606, ENSG00000174125, ENSG00000170345*	*257*	*104*	*8205*	*2.762832984*	*0.988815794*	*0.401298982*	*0.401298982*
KEGG_PATHWAY	*hsa04061: Viral protein interactio*	*9*	*1.72084130*	*0.012648173*	*ENSG00000163737, ENSG00000169245, ENSG00000137077, ENSG00000181374, ENSG00000138755, ENSG00000136244, ENSG00000163736,*	*257*	*100*	*8205*	*2.873346304*	*0.972738023*	*0.401298982*	*0.401298982*

	n with cytokine and cytokine receptor				ENSG00000275385, ENSG00000081041							
KEGG_PATHWAY	hsa04657:IL-17 signaling pathway	9	1.720841 3	0.008868356	ENSG00000073756, ENSG00000130522, ENSG00000050748, ENSG00000169245, ENSG00000136244, ENSG00000177606, ENSG00000170345, ENSG00000081041, ENSG00000125740	257	94	8205	3.056751387	0.919615971	0.38619078	0.38619078
KEGG_PATHWAY	hsa04625:C-type lectin receptor signaling pathway	8	1.529636711	0.043573898	ENSG00000158869, ENSG00000173846, ENSG00000073756, ENSG00000050748, ENSG00000179388, ENSG00000162711, ENSG00000136244, ENSG00000177606	257	104	8205	2.455851541	0.999996655	0.588367797	0.588367797
KEGG_PATHWAY	hsa05210:Colorectal cancer	8	1.529636711	0.017563278	ENSG00000006451, ENSG00000050748, ENSG00000109321, ENSG00000177606, ENSG00000170345, ENSG00000136997, ENSG00000172115, ENSG00000124762	257	86	8205	2.96986698	0.993359538	0.401298982	0.401298982
KEGG_PATHWAY	hsa05140:Leishmaniasis	7	1.338432122	0.032642342	ENSG00000073756, ENSG00000162747, ENSG00000198001, ENSG00000196126, ENSG00000177606, ENSG00000170345, ENSG00000196735	257	77	8205	2.902370004	0.999916605	0.486199096	0.486199096
KEGG_PATHWAY	hsa00	7	1.3	0.003193	ENSG00000141429,	257	47	8205	4.754946602	0.5954873	0.386190	0.38619

ATHWAY	514: Other types of O-glycan biosynthesis		38432122	027	ENSG00000171155, ENSG00000106392, ENSG00000144278, ENSG00000163389, ENSG00000151233, ENSG00000106003					87	78	078
KEGG_PATHWAY	hsa00563: Glycosylphosphatidylinositol (GPI)-anchor biosynthesis	5	0.956022945	0.008055207	ENSG00000112293, ENSG00000142892, ENSG00000069943, ENSG00000100564, ENSG00000151665	257	26	8205	6.139628854	0.898617474	0.38619078	0.38619078
KEGG analysis results of 566 DEGs in GSE100927 dataset												
Category	Term	Count	%	PValue	Genes	List Total	Pop Hits	Pop Total	Fold Enrichment	Bonferroni	Benjamini	FDR
KEGG_PATHWAY	hsa04062:Chemokine signaling pathway	26	4.601769912	3.11E-09	ENSG00000121281, ENSG00000173020, ENSG00000276085, ENSG00000141968, ENSG00000115415, ENSG00000137486, ENSG00000134215, ENSG00000137841, ENSG00000160791, ENSG00000163823, ENSG00000101333, ENSG00000277632, ENSG00000172380, ENSG00000158517, ENSG00000128340, ENSG00000170581, ENSG00000121807,	279	192	8465	4.108609618	9.05E-07	9.05E-07	7.18E-07

					ENSG00000197943, ENSG00000114353, ENSG00000275302, ENSG00000141480, ENSG00000171608, ENSG00000186810, ENSG00000254087, ENSG00000100077, ENSG00000168329							
KEGG_PATHWAY	*hsa05169:Epstein-Barr virus infection*	*25*	*4.424778761*	*4.02E-08*	*ENSG00000185507, ENSG00000095585, ENSG00000115415, ENSG00000111335, ENSG00000005844, ENSG00000196126, ENSG00000213928, ENSG00000111331, ENSG00000204592, ENSG00000165025, ENSG00000204257, ENSG00000204632, ENSG00000015475, ENSG00000116717, ENSG00000170581, ENSG00000197943, ENSG00000204525, ENSG00000172936, ENSG00000010671, ENSG00000204642, ENSG00000006062, ENSG00000232810, ENSG00000171608, ENSG00000146232, ENSG00000254087*	*279*	*202*	*8465*	*3.755012598*	*1.17E-05*	*3.90E-06*	*3.10E-06*
KEGG_PATHWAY	*hsa04060:Cytokine-cytokine receptor interactio*	*21*	*3.716814159*	*0.001811065*	*ENSG00000129048, ENSG00000177663, ENSG00000110324, ENSG00000107779, ENSG00000276085, ENSG00000215788, ENSG00000121807, ENSG00000172458, ENSG00000275302, ENSG00000117560,*	*279*	*297*	*8465*	*2.145288006*	*0.40992038*	*0.014243784*	*0.011306921*

	n				ENSG00000160791, ENSG00000232810, ENSG00000147168, ENSG00000186810, ENSG00000163823, ENSG00000227507, ENSG00000103522, ENSG00000164620, ENSG00000168329, ENSG00000182578, ENSG00000277632							
KEGG_PATHWAY	hsa04380:Osteoclast differentiation	20	3.539823009	3.07E-08	ENSG00000158517, ENSG00000095585, ENSG00000170581, ENSG00000043462, ENSG00000066336, ENSG00000115415, ENSG00000131042, ENSG00000213928, ENSG00000197943, ENSG00000143226, ENSG00000011600, ENSG00000165025, ENSG00000010671, ENSG00000006062, ENSG00000171608, ENSG00000232810, ENSG00000100365, ENSG00000203747, ENSG00000102575, ENSG00000182578	279	128	8465	4.740703405	8.94E-06	3.90E-06	3.10E-06
KEGG_PATHWAY	hsa04611:Platelet activation	17	3.008849558	2.66E-06	ENSG00000108821, ENSG00000121281, ENSG00000043462, ENSG00000213639, ENSG00000077420, ENSG00000197943, ENSG00000118640, ENSG00000058272, ENSG00000114353, ENSG00000137841, ENSG00000143226, ENSG00000165025, ENSG00000010671,	279	124	8465	4.159584923	7.73E-04	1.29E-04	1.02E-04

					ENSG00000171608, ENSG00000185532, ENSG00000254087, ENSG00000101333							
KEGG_PATHWAY	hsa04145:Phagosome	17	3.008849558	3.66E-05	ENSG00000135218, ENSG00000158517, ENSG00000196126, ENSG00000159720, ENSG00000204592, ENSG00000204525, ENSG00000276600, ENSG00000143226, ENSG00000165168, ENSG00000169896, ENSG00000204257, ENSG00000204642, ENSG00000102879, ENSG00000204632, ENSG00000100365, ENSG00000203747, ENSG00000110719	279	152	8465	3.393345595	0.010592398	6.66E-04	5.28E-04
KEGG_PATHWAY	hsa04514:Cell adhesion molecules	16	2.831858407	1.93E-04	ENSG00000110876, ENSG00000116824, ENSG00000076662, ENSG00000005844, ENSG00000139626, ENSG00000196126, ENSG00000081237, ENSG00000021645, ENSG00000088827, ENSG00000204592, ENSG00000204525, ENSG00000115232, ENSG00000169896, ENSG00000204257, ENSG00000204642, ENSG00000204632	279	157	8465	3.09202566	0.054494322	0.002436087	0.001933801
KEGG_PATHWAY	hsa05152:Tuberculosis	16	2.831858407	8.34E-04	ENSG00000110324, ENSG00000115415, ENSG00000196126, ENSG00000159720, ENSG00000130592, ENSG00000143226, ENSG00000172936,	279	180	8465	2.696933493	0.215458202	0.007824332	0.006211067

					ENSG00000165025, ENSG00000169896, ENSG00000204257, ENSG00000187796, ENSG00000102879, ENSG00000232810, ENSG00000015475, ENSG00000203747, ENSG00000110719							
KEGG_PATHWAY	hsa04662:B cell receptor signaling pathway	15	2.654867257	4.93E-07	ENSG00000095585, ENSG00000128340, ENSG00000168918, ENSG00000141968, ENSG00000111679, ENSG00000152689, ENSG00000131042, ENSG00000197943, ENSG00000134215, ENSG00000070190, ENSG00000165025, ENSG00000010671, ENSG00000171608, ENSG00000146232, ENSG00000254087	279	84	8465	5.417946749	1.44E-04	2.87E-05	2.28E-05
KEGG_PATHWAY	hsa04670:Leukocyte transendothelial migration	15	2.654867257	2.01E-05	ENSG00000158517, ENSG00000128340, ENSG00000213445, ENSG00000141968, ENSG00000005844, ENSG00000197943, ENSG00000134215, ENSG00000114353, ENSG00000115232, ENSG00000165168, ENSG00000169896, ENSG00000100985, ENSG00000171608, ENSG00000100852, ENSG00000100365	279	114	8465	3.992171288	0.005824934	3.89E-04	3.09E-04
KEGG_PATHWAY	hsa04142:Lysosome	14	2.477876106	3.81E-04	ENSG00000103066, ENSG00000213983, ENSG00000103174, ENSG00000104774, ENSG00000169919,	279	132	8465	3.217932008	0.105043968	0.004300122	0.003413499

					ENSG00000171298, ENSG00000198951, ENSG00000159720, ENSG00000102575, ENSG00000197081, ENSG00000110719, ENSG00000109861, ENSG00000164733, ENSG00000129226							
KEGG_PATHWAY	hsa05162: Measles	14	2.477876106	6.28E-04	ENSG00000185507, ENSG00000105327, ENSG00000170581, ENSG00000115415, ENSG00000196664, ENSG00000111335, ENSG00000213928, ENSG00000111331, ENSG00000115267, ENSG00000172936, ENSG00000117560, ENSG00000171608, ENSG00000147168, ENSG00000015475	279	139	8465	3.055877878	0.16706473	0.006091398	0.00483544
KEGG_PATHWAY	hsa05140:Leishmaniasis	13	2.300884956	6.78E-06	ENSG00000158517, ENSG00000111679, ENSG00000115415, ENSG00000196126, ENSG00000143226, ENSG00000172936, ENSG00000115232, ENSG00000165168, ENSG00000169896, ENSG00000204257, ENSG00000232810, ENSG00000100365, ENSG00000203747	279	77	8465	5.12242238	0.001971207	2.34E-04	1.86E-04
KEGG_PATHWAY	hsa04061: Viral protein interaction	13	2.300884956	9.89E-05	ENSG00000129048, ENSG00000110324, ENSG00000276085, ENSG00000121807, ENSG00000275302, ENSG00000160791, ENSG00000232810, ENSG00000147168,	279	100	8465	3.944265233	0.028372084	0.001439046	0.001142336

	with cytokine and cytokine receptor				ENSG00000186810, ENSG00000163823, ENSG00000168329, ENSG00000182578, ENSG00000277632							
KEGG_PATHWAY	hsa05416: Viral myocarditis	10	1.769911504	1.33E-04	ENSG00000204525, ENSG00000170624, ENSG00000204257, ENSG00000204642, ENSG00000128340, ENSG00000204632, ENSG00000196126, ENSG00000005844, ENSG00000015475, ENSG00000204592	279	60	8465	5.056750299	0.037866001	0.001838047	0.001459068
KEGG_PATHWAY	hsa05323:Rheumatoid arthritis	10	1.769911504	0.00336253	ENSG00000204257, ENSG00000232810, ENSG00000276085, ENSG00000227507, ENSG00000196126, ENSG00000005844, ENSG00000159720, ENSG00000102575, ENSG00000110719, ENSG00000277632	279	93	8465	3.262419548	0.624743364	0.024462408	0.019418612
KEGG_PATHWAY	hsa05133:Pertussis	9	1.592920354	0.003331771	ENSG00000172936, ENSG00000165410, ENSG00000169896, ENSG00000103490, ENSG00000140968, ENSG00000232810, ENSG00000159189, ENSG00000137752, ENSG00000114353	279	76	8465	3.59295416	0.621358021	0.024462408	0.019418612
KEGG_PATHWAY	hsa05150:Staphylococcus aureus	9	1.592920354	0.013379807	ENSG00000143226, ENSG00000169403, ENSG00000169896, ENSG00000204257, ENSG00000110876, ENSG00000159189, ENSG00000196126,	279	96	8465	2.844422043	0.980155054	0.080520905	0.063918657

	infection				*ENSG00000005844, ENSG00000203747*							
KEGG_PATHWAY	*hsa04640: Hematopoietic cell lineage*	*9*	*1.592920354*	*0.015889394*	*ENSG00000115232, ENSG00000135218, ENSG00000169896, ENSG00000204257, ENSG00000232810, ENSG00000166825, ENSG00000116824, ENSG00000196126, ENSG00000182578*	*279*	*99*	*8465*	*2.758227436*	*0.990542453*	*0.090663011*	*0.071969607*
KEGG_PATHWAY	*hsa04933: Antigen processing and presentation*	*9*	*1.592920354*	*0.016796971*	*ENSG00000165168, ENSG00000108821, ENSG00000232810, ENSG00000171608, ENSG00000115415, ENSG00000101333, ENSG00000086991, ENSG00000197943, ENSG00000137841*	*279*	*100*	*8465*	*2.730645161*	*0.992769414*	*0.092469662*	*0.073403752*
KEGG_PATHWAY	*hsa04660:Th17 cell differentiation*	*9*	*1.592920354*	*0.020802927*	*ENSG00000006062, ENSG00000141968, ENSG00000111679, ENSG00000043462, ENSG00000232810, ENSG00000171608, ENSG00000146232, ENSG00000081237, ENSG00000134215*	*279*	*104*	*8465*	*2.625620347*	*0.997796054*	*0.102604268*	*0.081448749*
KEGG_PATHWAY	*hsa05330: Allograft rejection*	*8*	*1.415929204*	*2.01E-04*	*ENSG00000204525, ENSG00000204257, ENSG00000117560, ENSG00000204642, ENSG00000232810, ENSG00000204632, ENSG00000196126, ENSG00000204592*	*279*	*38*	*8465*	*6.387474061*	*0.056840913*	*0.002438101*	*0.0019354*
KEGG_PATHWAY	*hsa05332: Graft-versus-host*	*8*	*1.415929204*	*3.84E-04*	*ENSG00000204525, ENSG00000204257, ENSG00000117560, ENSG00000204642, ENSG00000232810, ENSG00000204632,*	*279*	*42*	*8465*	*5.779143198*	*0.105798962*	*0.004300122*	*0.003413499*

	disease				ENSG00000196126, ENSG00000204592							
KEGG_PATHWAY	hsa04940:Type I diabetes mellitus	8	1.415929204	4.46E-04	ENSG00000204525, ENSG00000204257, ENSG00000117560, ENSG00000204642, ENSG00000232810, ENSG00000204632, ENSG00000196126, ENSG00000204592	279	43	8465	5.644744519	0.121799104	0.004809293	0.003817686
KEGG_PATHWAY	hsa05145:Toxoplasmosis	8	1.415929204	0.075190536	ENSG00000172936, ENSG00000160791, ENSG00000110324, ENSG00000204257, ENSG00000232810, ENSG00000115415, ENSG00000196126, ENSG00000114353	279	112	8465	2.167178699	1	0.265769922	0.210972
KEGG_PATHWAY	hsa05320: Autoimmune thyroid disease	7	1.238938053	0.007470255	ENSG00000204525, ENSG00000204257, ENSG00000117560, ENSG00000204642, ENSG00000204632, ENSG00000196126, ENSG00000204592	279	53	8465	4.007236086	0.88718484	0.048784408	0.038725767
KEGG_PATHWAY	hsa05321:Inflammatory bowel disease	7	1.238938053	0.01942022	ENSG00000204257, ENSG00000178573, ENSG00000232810, ENSG00000115415, ENSG00000147168, ENSG00000196126, ENSG00000103522	279	65	8465	3.267438655	0.996676974	0.099145333	0.078702997
KEGG_PATHWAY	hsa05340:Primary immunodeficiency	5	0.884955752	0.035006715	ENSG00000128322, ENSG00000095585, ENSG00000010671, ENSG00000147168, ENSG00000081237	279	38	8465	3.992171288	0.999968626	0.145527915	0.115522159
KEGG_P	hsa04	5	0.8	0.076272	ENSG00000115232,	279	49	8465	3.095969571	1	0.265769	0.21097

ATHWAY	*672:Intestinal immune network for IgA production*		*84955752*	*42*	*ENSG00000204257, ENSG00000006062, ENSG00000196126, ENSG00000139626*						*922*	*2*
KEGG_PATHWAY	*hsa01523: Asthma*	*4*	*0.707964602*	*0.074162683*	*ENSG00000114770, ENSG00000232810, ENSG00000076351, ENSG00000165457*	*279*	*30*	*8465*	*4.045400239*	*1*	*0.265769922*	*0.210972*

Supplementary Table S3

Common differential genes between GSE135917 and GSE135917

PRCP
RALA
PTPN22
WNT11
CSRNP1
TPPP3
PDLIM3
PARP12
FAM96A
COMMD9
MYH11
BHMT2
P2RY6
CNTN4
VIPR2
NIPSNAP3B
EGFL6
SNORA15
GLB1
TMOD1
NNMT
PI16

NLRP3
C12orf75
ADAMTSL3
OGN
PF4
ID2
FOS
FNDC1
ACTG2
HIGD1A
PDPN
BAMBI
FOSB
RRM2
KRT18P55
LAIR1
HSD11B1
HLA-J
SLC6A6
PTGIS
CLEC4A
MARCO
ACOT4
CD163L1
GPR160
SLAMF7
DPP4
TLR1
PARM1
HBB
GPRC5A
CXCL9
SLPI
EVI2A
TM4SF18
CXCL10
RASGRP3
RGS18
JUNB
PTPRC
SLC28A3
NPR3
WEE1

PPBP
BTN3A2
ERRFI1
ADAMTS1
GFPT2
MT1A
LIPA
CCL13
PERP
NPY1R
HBA2
MNDA
LYVE1
ID4
PLA2G7
CCL18
CHI3L1
HLA-DQA1
MMP7
RASGEF1B
SCARNA5
EVI2B
FCER1G
LAPTM5
HCST
HLA-DRB1
SNX10
SDS

Supplementary Table S4

The GO analysis of 93 genes									
ONTOLOGY	*ID*	*Description*	*GeneRatio*	*BgRatio*	*pvalue*	*p.adjust*	*qvalue*	*geneID*	*Count*
BP	*GO:0030593*	*neutrophil chemotaxis*	*8/86*	*106/18800*	*2.88E-08*	*5.48E-05*	*4.50E-05*	*PF4/DPP4/CXCL9/CXCL10/PPBP/CCL13/CCL18/FCER1G*	*8*
BP	*GO:0071621*	*granulocyte chemotaxis*	*8/86*	*128/18800*	*1.26E-07*	*7.99E-05*	*6.55E-05*	*PF4/DPP4/C*	*8*

								XCL9/CXCL10/PPBP/CCL13/CCL18/FCER1G	
BP	GO:1990266	neutrophil migration	8/86	128/18800	1.26E-07	7.99E-05	6.55E-05	PF4/DPP4/CXCL9/CXCL10/PPBP/CCL13/CCL18/FCER1G	8
BP	GO:0097530	granulocyte migration	8/86	154/18800	5.22E-07	0.000248212	0.000203581	PF4/DPP4/CXCL9/CXCL10/PPBP/CCL13/CCL18/FCER1G	8
BP	GO:0097529	myeloid leukocyte migration	9/86	229/18800	1.03E-06	0.000391108	0.000320782	PF4/DPP4/CXCL9/CXCL10/PPBP/CCL13/PLA2G7/CCL18/FCER1G	9
BP	GO:0030595	leukocyte chemotaxis	9/86	236/18800	1.32E-06	0.000418435	0.000343195	PF4/DPP4/CXCL9/CXCL10/PPBP/CCL13/PLA2G7/CCL18/FCER1G	9
BP	GO:0070098	chemokine-mediated signaling pathway	6/86	89/18800	3.29E-06	0.000894857	0.000733952	PF4/CXCL9/CXCL10/PPBP/CCL13/CCL18	6
BP	GO:0002763	positive regulation of myeloid leukocyte differentiation	5/86	55/18800	5.18E-06	0.001034581	0.000848553	PF4/ID2/FOS/EVI2B/HLA-DRB1	5
BP	GO:1990868	response to chemokine	6/86	97/18800	5.44E-06	0.001034581	0.000848553	PF4/CXCL9/CXCL10/PPBP/CCL13/CCL18	6
BP	GO:1990869	cellular response to chemokine	6/86	97/18800	5.44E-06	0.001034581	0.000848553	PF4/CXCL9/CXCL10/PPBP/CCL13/CCL18	6
CC	GO:0030139	endocytic vesicle	8/90	342/19594	0.000177435	0.01351283	0.010391209	RALA/MARCO/DPP4/TLR1/HBB/H	8

								BA2/HLA-DQA1/HLA-DRB1	
CC	GO:0101002	ficolin-1-rich granule	6/90	185/19594	0.000210829	0.01351283	0.010391209	PRCP/COMMD9/GLB1/HBB/MNDA/FCER1G	6
CC	GO:0009897	external side of plasma membrane	9/90	455/19594	0.000242745	0.01351283	0.010391209	CLEC4A/CD163L1/SLAMF7/CXCL9/CXCL10/PTPRC/BTN3A2/FCER1G/HLA-DRB1	9
CC	GO:0034774	secretory granule lumen	7/90	322/19594	0.000706032	0.021511066	0.016541759	COMMD9/GLB1/PF4/SLPI/PPBP/MNDA/CHI3L1	7
CC	GO:0060205	cytoplasmic vesicle lumen	7/90	325/19594	0.000745548	0.021511066	0.016541759	COMMD9/GLB1/PF4/SLPI/PPBP/MNDA/CHI3L1	7
CC	GO:0031983	vesicle lumen	7/90	327/19594	0.000772853	0.021511066	0.016541759	COMMD9/GLB1/PF4/SLPI/PPBP/MNDA/CHI3L1	7
CC	GO:0070820	tertiary granule	5/90	164/19594	0.00095762	0.02284608	0.017568369	PRCP/LAIR1/HBB/PPBP/FCER1G	5
CC	GO:0031838	haptoglobin-hemoglobin complex	2/90	11/19594	0.001117031	0.023318028	0.017931291	HBB/HBA2	2
CC	GO:0005833	hemoglobin complex	2/90	12/19594	0.001336436	0.024798311	0.019069612	HBB/HBA2	2
CC	GO:1904813	ficolin-1-rich granule lumen	4/90	124/19594	0.002566765	0.041189011	0.031673869	COMMD9/GLB1/HBB/MNDA	4
MF	GO:0008009	chemokine activity	6/84	49/18410	9.00E-08	2.04E-05	1.71E-05	PF4/CXCL9/CXCL10/PPBP/CCL13/CCL18	6
MF	GO:0001664	G protein-coupled receptor binding	10/84	288/18410	7.47E-07	6.44E-05	5.40E-05	RALA/WNT11/PF4/BAMBI/MARCO/CXCL9/CXCL10/PPBP/C	10

								CL13/CCL18	
MF	GO:0042379	chemokine receptor binding	6/84	71/18410	8.51E-07	6.44E-05	5.40E-05	PF4/CXCL9/CXCL10/PPBP/CCL13/CCL18	6
MF	GO:0045236	CXCR chemokine receptor binding	4/84	18/18410	1.18E-06	6.67E-05	5.60E-05	PF4/CXCL9/CXCL10/PPBP	4
MF	GO:0005125	cytokine activity	7/84	235/18410	9.93E-05	0.004507152	0.003782956	WNT11/PF4/CXCL9/CXCL10/PPBP/CCL13/CCL18	7
MF	GO:0005126	cytokine receptor binding	7/84	272/18410	0.000244357	0.00924484	0.007759406	PF4/BAMBI/CXCL9/CXCL10/PPBP/CCL13/CCL18	7
MF	GO:0048018	receptor ligand activity	9/84	489/18410	0.000385029	0.012112741	0.010166502	WNT11/OGN/PF4/DPP4/CXCL9/CXCL10/PPBP/CCL13/CCL18	9
MF	GO:0030546	signaling receptor activator activity	9/84	496/18410	0.000426881	0.012112741	0.010166502	WNT11/OGN/PF4/DPP4/CXCL9/CXCL10/PPBP/CCL13/CCL18	9
MF	GO:0042277	peptide binding	7/84	322/18410	0.000671549	0.015785096	0.013248793	VIPR2/MARCO/TLR1/NPR3/NPY1R/HLA-DQA1/HLA-DRB1	7
MF	GO:0005539	glycosaminoglycan binding	6/84	234/18410	0.000703591	0.015785096	0.013248793	NLRP3/PF4/CXCL10/PTPRC/ADAMTS1/LYVE1	6

The KEGG analysis of 93 genes											
Term	Count	%	PValue	Genes	List Total	Pop Hits	Pop Total	Fold Enrichment	Bonferroni	Benjamini	FDR

hsa04061:Viral protein interaction with cytokine and cytokine receptor	*6*	*6.451612903*	*2.15E-04*	*ENSG00000163737, ENSG00000169245, ENSG00000181374, ENSG00000138755, ENSG00000163736, ENSG00000275385*	*48*	*100*	*8465*	*10.58125*	*0.031378228*	*0.031877638*	*0.031877638*
hsa04062:Chemokine signaling pathway	*6*	*6.451612903*	*0.004036888*	*ENSG00000163737, ENSG00000169245, ENSG00000181374, ENSG00000138755, ENSG00000163736, ENSG00000275385*	*48*	*192*	*8465*	*5.511067708*	*0.450457192*	*0.298729705*	*0.298729705*
hsa05310:Asthma	*3*	*3.225806452*	*0.012665059*	*ENSG00000158869, ENSG00000196126, ENSG00000196735*	*48*	*31*	*8465*	*17.06653226*	*0.848383328*	*0.624809594*	*0.624809594*
hsa04620:Toll-like receptor signaling pathway	*4*	*4.301075269*	*0.01976027*	*ENSG00000169245, ENSG00000138755, ENSG00000174125, ENSG00000170345*	*48*	*104*	*8465*	*6.782852564*	*0.947858857*	*0.700352227*	*0.700352227*
hsa04060:Cytokine-cytokine receptor interaction	*6*	*6.451612903*	*0.023660548*	*ENSG00000163737, ENSG00000169245, ENSG00000181374, ENSG00000138755, ENSG00000163736, ENSG00000275385*	*48*	*297*	*8465*	*3.562710438*	*0.971098252*	*0.700352227*	*0.700352227*
hsa05140:Leishmaniasis	*3*	*3.225806452*	*0.067894868*	*ENSG00000196126, ENSG00000170345, ENSG00000196735*	*48*	*77*	*8465*	*6.870941558*	*0.999969744*	*1*	*1*
hsa05164:Influenza A	*4*	*4.301075269*	*0.068842048*	*ENSG00000169245, ENSG00000162711, ENSG00000196126, ENSG00000196735*	*48*	*171*	*8465*	*4.125243665*	*0.999973971*	*1*	*1*
hsa05152:Tuberculosis	*4*	*4.301075269*	*0.077680251*	*ENSG00000158869, ENSG00000196126, ENSG00000174125, ENSG00000196735*	*48*	*180*	*8465*	*3.918981481*	*0.999993655*	*1*	*1*
hsa04658:Th1 and Th2 cell differentiation	*3*	*3.225806452*	*0.092244879*	*ENSG00000196126, ENSG00000170345, ENSG00000196735*	*48*	*92*	*8465*	*5.750679348*	*0.999999398*	*1*	*1*
hsa05323:Rh	*3*	*3.225*	*0.0939494*	*ENSG00000196126,*	*48*	*93*	*8465*	*5.68884*	*0.99999954*	*1*	*1*

eumatoid arthritis		8064 52	16	ENSG00000170345, ENSG00000196735				4086	4		
hsa04657:IL-17 signaling pathway	3	3.225 8064 52	0.0956632 18	ENSG00000169245, ENSG00000170345, ENSG00000125740	48	94	8465	5.62832 4468	0.99999965 6	1	1

Supplementary Table S6

List of genes in the strongest positive and negative modules for OSA and AS

OSA_grey	*OSA_turquoise*	*AS_turquoise*
ANXA3	*AADAC*	*ABCA1*
AOX1	*ABCC3*	*ABCA6*
CASQ2	*ACTA2*	*ABCA7*
CDH6	*ADAMTS1*	*ABCA8*
CP	*ADAMTS18*	*ABCC3*
FMOD	*ADGRD1*	*ABCC5*
GPAT3	*ANO3*	*ABCC8*
IFI44L	*APOB*	*ABCC9*
LGI1	*AQP4*	*ABCG1*
LHCGR	*AREG*	*ABRA*
LIMD1-AS1	*ASPN*	*ACADL*
LOC101926933	*ATF3*	*ACAN*
LOC389834	*ATP1A2*	*ACCN4*
LTF	*AZGP1*	*ACOT4*
MAL2	*B4GALT6*	*ACP5*
MXRA5	*BCHE*	*ACRBP*
PDE11A	*BNC1*	*ACSL1*
PLAC8	*C1GALT1*	*ACTA1*
RNU5B-1	*C4B_2*	*ACTBL2*
SAA1	*C7*	*ACTG2*
SAA2	*CAB39L*	*ADA*
SFRP2	*CACNA2D1*	*ADAM28*
SNORD45A	*CADM3*	*ADAM8*
SNORD45B	*CCDC3*	*ADAMDEC1*
STOX1	*CCL13*	*ADAMTS1*
TNC	*CCL18*	*ADAMTS14*
USP32P1	*CCL2*	*ADAMTS5*
VNN2	*CCL5*	*ADAMTS8*
ZNF117	*CCL8*	*ADAMTS9*
ZNF91	*CCND2*	*ADAMTSL3*
	CD226	*ADAP2*
	CD24	*ADARB1*
	CD69	*ADCY1*

	CDR1	*ADCY7*
	CES1P1	*ADH1A*
	CFB	*ADH1B*
	CFD	*ADH1C*
	CHI3L2	*ADH5*
	CIDEA	*ADORA2B*
	CLDN1	*ADORA3*
	CLIC6	*ADPGK*
	COL14A1	*ADRA1B*
	COL1A1	*ADRA2C*
	COL6A6	*ADRB2*
	COL8A1	*ADRBK2*
	CPA3	*ADSSL1*
	CR1	*AFAP1L1*
	CRHBP	*AGPAT4-IT1*
	CSF3R	*AGRP*
	CSN1S1	*AGT*
	CSRNP1	*AGTR1*
	CSTA	*AHSP*
	CTGF	*AIM1*
	CXCL10	*AIM2*
	CXCL14	*AKAP12*
	CXCL2	*AKR1B1*
	CXCL9	*ALAS2*
	CXCR1	*ALDH1A3*
	CXCR2	*ALDH1B1*
	CYP1B1	*ALDH1L1*
	CYR61	*ALK*
	DDIT3	*ALOX15B*
	DDX3Y	*ALOX5*
	DEFB132	*ALOX5AP*
	DLEU2	*AMICA1*
	DPP4	*AMIGO2*
	DPT	*AMPH*
	DSC3	*ANGPT1*
	DUSP1	*ANGPTL1*
	DUSP4	*ANGPTL5*
	EGFL6	*ANGPTL7*
	EGR1	*ANKLE1*
	EIF1AY	*ANKRD1*
	ENPEP	*ANKRD37*
	ERRFI1	*ANKRD58*

	FAM177A1	*ANPEP*
	FAM200B	*ANXA3*
	FAM96A	*ANXA8L2*
	FAP	*AOAH*
	FGF10	*AOC4*
	FGF9	*AOX1*
	FGFBP2	*AP1B1*
	FLJ36840	*APBB1IP*
	FMO3	*APCDD1*
	FNDC1	*APCDD1L*
	FOS	*APOBEC3A*
	FOSB	*APOBEC3G*
	FRZB	*APOBR*
	GCA	*APOC1*
	GFPT2	*APOC2*
	GLB1	*APOD*
	GLDN	*APOE*
	GPLD1	*AQP9*
	GPR183	*AR*
	GPR34	*ARC*
	GREM1	*ARHGAP22*
	GSTM1	*ARHGAP25*
	GSTM3	*ARHGAP27*
	GSTM5	*ARHGAP30*
	GZMK	*ARHGAP9*
	HAS2	*ARHGDIB*
	HBA2	*ARHGEF17*
	HBB	*ARHGEF25*
	HIST1H2BC	*ARHGEF26*
	HIST1H4C	*ARID3A*
	HLA-DQA1	*ARID5A*
	HLA-DQB1	*ARL4C*
	HLA-DRB1	*ARRB2*
	HLA-DRB5	*ARSA*
	HP	*ASAH1*
	HPR	*ASB16*
	HSD11B1	*ASCL2*
	HSD17B13	*ASGR2*
	HSD17B6	*ASPA*
	HSP90AA6P	*ASPHD1*
	HSPB7	*ASPM*
	ID4	*ASPN*

	IDO1	*ATG16L2*
	IGBP1P1	*ATP1B1*
	IGHM	*ATP1B2*
	IGKC	*ATP6V0D2*
	IGLJ3	*ATP6V1A*
	IL1RL1	*ATP6V1B2*
	IL6	*AVPR1A*
	IL7R	*AZU1*
	IMPAD1	*B3GNT5*
	INPP4B	*BAG2*
	IQCF2	*BAMBI*
	IRF8	*BATF*
	ITLN1	*BCAT1*
	JCHAIN	*BCHE*
	JUN	*BCL2A1*
	JUNB	*BDKRB1*
	KDM5D	*BDKRB2*
	KLF4	*BDNF*
	KLHL31	*BEND5*
	KLHL4	*BEX2*
	KRT18P55	*BEX5*
	KRT19	*BGLAP*
	LBP	*BHLHE41*
	LINC00917	*BID*
	LOC100128775	*BIN2*
	LOC101060256	*BIRC5*
	LOC102723407	*BLNK*
	LOC1720	*BMP2*
	LOC728323	*BMP2K*
	LOX	*BMPR1A*
	LRRC70	*BMX*
	LRRTM4	*BST2*
	LYPLAL1	*BTC*
	LYVE1	*BTK*
	MAMDC2	*BVES*
	MCL1	*C10orf10*
	MEGF9	*C10orf105*
	MIR10A	*C10orf116*
	MIR21	*C11orf35*
	MIR224	*C11orf96*
	MIR24-2	*C12orf53*
	MMRN1	*C12orf75*

	MOP-1	*C13orf15*
	MSC	*C13orf33*
	MSL3P1	*C14orf132*
	MSMO1	*C14orf34*
	MTND2P28	*C15orf27*
	MYC	*C15orf48*
	MYOC	*C16orf54*
	NAMPT	*C16orf89*
	NECAB1	*C17orf87*
	NFIL3	*C19orf59*
	NFKBIZ	*C1QA*
	NIPSNAP3B	*C1QB*
	NNAT	*C1QC*
	NPR3	*C1QTNF4*
	NPY1R	*C1QTNF7*
	NPY5R	*C1QTNF8*
	NR4A1	*C1orf133*
	NR4A2	*C1orf140*
	NRCAM	*C1orf162*
	OCLN	*C1orf186*
	OGN	*C1orf38*
	OR2A9P	*C1orf93*
	OR52K3P	*C2*
	OR7E13P	*C20orf103*
	OSR2	*C20orf46*
	OVOS	*C21orf96*
	PAMR1	*C2orf40*
	PAX8-AS1	*C3*
	PCLO	*C3AR1*
	PDE8B	*C3orf70*
	PDGFD	*C4orf48*
	PDLIM3	*C5AR1*
	PEG10	*C5orf20*
	PEMT	*C5orf4*
	PERP	*C6*
	PF4	*C6orf115*
	PFKFB3	*C6orf192*
	PHGDH	*C7*
	PI16	*C7orf41*
	PKHD1L1	*C7orf58*
	PKP2	*C8orf4*
	PLA2G2A	*C8orf84*

	PLA2G4A	*C9orf139*
	PNPLA3	*C9orf167*
	POPDC3	*C9orf47*
	PPBP	*CA1*
	PRG4	*CA12*
	PSMB8	*CA2*
	PTGIS	*CACNA1H*
	PTGS2	*CACNA2D4*
	PTPN22	*CACNB2*
	RANBP3L	*CADM3*
	RASGEF1B	*CALD1*
	RBP4	*CAMK2G*
	RGS1	*CAMK2N1*
	RGS18	*CAMP*
	RGS2	*CAND2*
	RGS7BP	*CAP2*
	RPS4Y1	*CAPG*
	RPS4Y2	*CAPN1*
	S100A4	*CAPN3*
	S100A8	*CARD16*
	SAA2-SAA4	*CARD9*
	SCFV	*CARTPT*
	SCIN	*CASP1*
	SELE	*CASQ2*
	SELL	*CATSPER1*
	SERPINB9P1	*CAV1*
	SERPINE1	*CBS*
	SFRP4	*CCDC146*
	SIM1	*CCDC3*
	SLC28A3	*CCL13*
	SLC2A14	*CCL15*
	SLC2A3	*CCL18*
	SLPI	*CCL19*
	SMOC2	*CCL2*
	SNORA28	*CCL20*
	SNORD113-4	*CCL21*
	SNORD116-21	*CCL26*
	SNORD14E	*CCL3*
	SNORD44	*CCL3L3*
	SNORD49A	*CCL4*
	SNORD54	*CCL5*
	SNORD78	*CCL7*

	SNORD82	*CCNB2*
	SNX10	*CCR1*
	SOCS3	*CCR2*
	SPAG17	*CCR5*
	STAG3L4	*CCR7*
	STMN2	*CCRL1*
	SULF1	*CCRL2*
	SULT1A2	*CD109*
	TAS2R43	*CD14*
	TAS2R45	*CD163*
	TBL1Y	*CD163L1*
	TC2N	*CD180*
	TFPI2	*CD209*
	TIPARP	*CD24*
	TNFRSF11B	*CD248*
	TNMD	*CD27*
	TPRG1	*CD28*
	TRDN	*CD300A*
	TRIM48	*CD300C*
	TTTY10	*CD300LF*
	TTTY9B	*CD33*
	TUBB1	*CD36*
	TUBB4A	*CD37*
	TXLNGY	*CD38*
	USP9Y	*CD3D*
	UTY	*CD3G*
	VCAM1	*CD40LG*
	VCAN	*CD5*
	WT1	*CD52*
	ZBED8	*CD53*
	ZFP36	*CD6*
	ZFY	*CD68*
	ZNF204P	*CD69*
	ZNF718	*CD74*
	ZNF814	*CD83*
	ZNF845	*CD84*
	ZNF98	*CD8A*
		CD8B
		CD96
		CDC45
		CDCA8
		CDCP1

		CDH15
		CDH19
		CDH23
		CDH3
		CDKN1A
		CDKN2A
		CDO1
		CDON
		CDT1
		CEBPA
		CEBPE
		CECR1
		CENPM
		CERS1
		CES1
		CFHR3
		CFL2
		CFP
		CHGA
		CHI3L1
		CHI3L2
		CHIT1
		CHODL
		CHRDL1
		CHRDL2
		CHRM4
		CHRNA1
		CHURC1
		CILP
		CILP2
		CKB
		CKMT2
		CKS2
		CLC
		CLDN11
		CLDN23
		CLEC10A
		CLEC11A
		CLEC18B
		CLEC2B
		CLEC3B
		CLEC4A

		CLEC4G
		CLEC4GP1
		CLEC5A
		CLECL1
		CLIC3
		CLMP
		CLN6
		CLSTN2
		CLU
		CMTM2
		CMTM3
		CMTM7
		CNIH3
		CNN1
		CNR1
		CNTN3
		CNTN4
		COBLL1
		COCH
		COL10A1
		COL12A1
		COL14A1
		COL15A1
		COL18A1
		COL1A1
		COL1A2
		COL21A1
		COL3A1
		COL4A1
		COL4A4
		COL4A5
		COL4A6
		COL8A1
		COLEC11
		COLEC12
		COMP
		COPG2IT1
		CORO1A
		CORO2A
		CORO7
		COTL1
		CP

		CPA3
		CPE
		CPNE7
		CPVL
		CR1
		CRISPLD1
		CRISPLD2
		CRLF1
		CRTAC1
		CRTAM
		CRYAB
		CRYBB1
		CSDC2
		CSF1R
		CSF2RA
		CSF3
		CSF3R
		CSK
		CSRP1
		CSRP2
		CST6
		CSTB
		CTAG1A
		CTHRC1
		CTNNAL1
		CTSA
		CTSB
		CTSD
		CTSH
		CTSK
		CTSL1
		CTSL1P8
		CTSL2
		CTSS
		CTSZ
		CX3CR1
		CXCL1
		CXCL10
		CXCL14
		CXCL16
		CXCL5
		CXCL9

		CXCR2
		CXCR2P1
		CXCR3
		CXCR4
		CXCR6
		CXCR7
		CXorf65
		CXorf69
		CYBA
		CYBB
		CYP1A1
		CYP1B1
		CYP27A1
		CYP27B1
		CYP4X1
		CYTH4
		CYTIP
		CYTL1
		DAAM2
		DAB2
		DACT3
		DAPL1
		DAPP1
		DARC
		DBP
		DCDC2C
		DCLK1
		DCN
		DDIT4
		DDIT4L
		DDX3Y
		DDX43
		DDX60L
		DEFA3
		DEFA4
		DENND1C
		DENND2D
		DENND4B
		DES
		DFNA5
		DGKD
		DHDH

		DHRS9
		DIO3
		DIO3OS
		DIRAS1
		DKFZp451A211
		DKK3
		DLG3
		DLX5
		DMP1
		DMPK
		DNAJA4
		DNAJB4
		DNAJB5
		DNAJC5B
		DNASE2
		DNASE2B
		DOCK2
		DOCK8
		DOK1
		DOK3
		DPEP2
		DPEP3
		DPP4
		DPRXP4
		DPT
		DRAM1
		DSC2
		DSP
		DUSP10
		DUSP2
		DUSP26
		DUSP6
		EBF1
		EBI3
		ECM1
		EFHA2
		EFHD1
		EFHD2
		EFNB2
		EGFL6
		EGR2
		ELL2

		ELN
		ELOVL7
		EMB
		EMILIN2
		EMR2
		EMX2
		EMX2OS
		ENPP1
		ENTPD2
		EPB41L3
		EPB42
		EPB49
		EPHX2
		ERAP2
		ERP27
		ERRFI1
		ESAM
		EVI2A
		EVI2B
		EVX1
		EYA2
		F11R
		F13A1
		F3
		F5
		FABP3
		FABP4
		FABP5
		FAIM2
		FAM101A
		FAM107A
		FAM110A
		FAM134B
		FAM150B
		FAM162B
		FAM180B
		FAM196A
		FAM19A2
		FAM20A
		FAM26F
		FAM43B
		FAM46B

		FAM46C
		FAM49A
		FAM49B
		FAM59A
		FAM70A
		FAM71E1
		FAM78A
		FAM83D
		FAM96A
		FBLN1
		FBLN2
		FBLN5
		FBP1
		FBXO16
		FBXO2
		FBXO32
		FBXO41
		FCAR
		FCER1A
		FCER1G
		FCGBP
		FCGR1B
		FCGR2A
		FCGR2C
		FCGR3A
		FCGRT
		FCHO1
		FCN1
		FERMT2
		FERMT3
		FGD3
		FGF13
		FGF7
		FGFR3
		FGL2
		FGR
		FHL5
		FHOD3
		FIBIN
		FILIP1
		FKBP15
		FLJ30901

		FLJ42875
		FLNA
		FLNC
		FLVCR2
		FMNL1
		FMO2
		FMO3
		FN1
		FNDC1
		FOLR2
		FOSL2
		FOXC1
		FOXL1
		FOXS1
		FPR1
		FPR2
		FRK
		FRMD4B
		FRZB
		FSCN1
		FST
		FSTL3
		FTHL17
		FTL
		FUCA1
		FUT8
		FXYD1
		FYB
		FZD10
		FZD4
		GAA
		GABRE
		GADD45A
		GADD45B
		GADD45G
		GAL
		GALNT14
		GALNT5
		GALNT6
		GALNTL1
		GALNTL2
		GAS1

		GAS2L3
		GBP5
		GCHFR
		GDF10
		GEM
		GFPT2
		GFRA1
		GFRA2
		GGT8P
		GGTLC1
		GGTLC2
		GIMAP1
		GIMAP2
		GIMAP4
		GIMAP6
		GIMAP7
		GIMAP8
		GJA4
		GJC1
		GK3P
		GLA
		GLDN
		GLIS1
		GLT1D1
		GLUL
		GM2A
		GMFG
		GMIP
		GNAI1
		GNG11
		GNG13
		GNG8
		GNLY
		GPC3
		GPNMB
		GPR132
		GPR137B
		GPR160
		GPR171
		GPR183
		GPR20
		GPR34

		GPR65
		GPR68
		GPR84
		GPRASP1
		GPRC5A
		GPSM3
		GPX1
		GPX3
		GRAMD4
		GRB14
		GREM1
		GREM2
		GRIA2
		GRP
		GSTA2
		GSTM5
		GUCA2B
		GULP1
		GUSB
		GZMA
		GZMB
		GZMH
		GZMK
		H19
		HAMP
		HAND2
		HAPLN2
		HAVCR2
		HBA2
		HBB
		HBD
		HBG1
		HBM
		HBQ1
		HCAR3
		HCK
		HCLS1
		HCST
		HDC
		HEMGN
		HES4
		HEXA

		HEXB
		HEY2
		HEYL
		HHIP
		HILPDA
		HIST1H1A
		HIST1H1B
		HIST1H1C
		HIST1H1D
		HIST1H1E
		HIST1H2AD
		HIST1H3B
		HIST2H2AA4
		HIST2H3A
		HJURP
		HK2
		HK3
		HLA-B
		HLA-DMA
		HLA-DMB
		HLA-DPA1
		HLA-DPB1
		HLA-DPB2
		HLA-DQA1
		HLA-DQA2
		HLA-DQB1
		HLA-DQB2
		HLA-DRA
		HLA-DRB1
		HLA-DRB4
		HLA-DRB5
		HLA-DRB6
		HLA-G
		HLA-J
		HLF
		HMCN1
		HMGA1
		HMGA1P4
		HMHA1
		HMMR
		HMOX1
		HN1

		HOTAIR
		HOXA11-AS1
		HOXA5
		HOXA6
		HOXA7
		HOXA9
		HOXB6
		HOXC10
		HOXC4
		HOXC6
		HOXC8
		HOXC9
		HOXD8
		HP
		HPGDS
		HPR
		HPSE
		HPSE2
		HRC
		HRCT1
		HRK
		HS3ST1
		HS3ST2
		HSD11B1
		HSD17B6
		HSP90AB1
		HSPA1A
		HSPA1B
		HSPA6
		HSPB2
		HSPB3
		HSPB6
		HSPB7
		HTR2B
		HTRA3
		HTRA4
		HVCN1
		IBSP
		ICAM1
		ICAM4
		ID1
		ID2

		ID4
		IDH1
		IFI27
		IFI30
		IFI44L
		IFI6
		IFIT1
		IFIT2
		IFIT3
		IGANRP
		IGF1
		IGF2
		IGF2BP3
		IGFBP1
		IGFBP2
		IGFBP3
		IGFBP4
		IGFBP5
		IGFBP6
		IGFBPL1
		IGFLR1
		IGJ
		IGLL1
		IGLL5
		IGSF1
		IGSF6
		IKZF1
		IL10
		IL10RA
		IL11
		IL17B
		IL17D
		IL17RA
		IL17RD
		IL18
		IL18RAP
		IL1B
		IL1RN
		IL21R
		IL2RA
		IL2RB
		IL2RG

		IL33
		IL4I1
		IL7R
		IL8
		INHBA
		INPP5D
		INSIG1
		IQCJ-SCHIP1
		IQGAP3
		IRF1
		IRF5
		IRF7
		IRF8
		IRX1
		ISM1
		ITGA1
		ITGA10
		ITGA11
		ITGA4
		ITGA5
		ITGA7
		ITGA8
		ITGAL
		ITGAM
		ITGAX
		ITGB1BP2
		ITGB2
		ITGB3
		ITGB7
		ITIH1
		ITIH3
		ITIH4
		ITLN1
		ITM2A
		ITM2C
		JAK3
		JAKMIP2
		JPH2
		JUNB
		KANK1
		KANK2
		KAT2A

		KCNA5
		KCNAB1
		KCNAB2
		KCNE3
		KCNIP3
		KCNJ18
		KCNJ2
		KCNJ3
		KCNJ5
		KCNJ8
		KCNK17
		KCNK2
		KCNK5
		KCNMA1
		KCNN4
		KIAA0101
		KIAA0226L
		KIAA1199
		KIR2DL4
		KLF9
		KLHL6
		KLK1
		KLRB1
		KMO
		KRT1
		KRT14
		KRT16
		KRT16P2
		KRT17
		KRT18
		KRT18P55
		KRT25
		KRT31
		KRT86
		LAG3
		LAIR1
		LAMA2
		LAMB3
		LAMP3
		LAPTM5
		LARGE
		LAT

		LAT2
		LAYN
		LBP
		LCK
		LCP2
		LDB3
		LDLR
		LDOC1
		LEF1
		LEFTY2
		LEP
		LGALS2
		LGALS9
		LGALS9C
		LGI4
		LGMN
		LHFP
		LHFPL2
		LIFR
		LILRA2
		LILRA3
		LILRA4
		LILRA6
		LILRB1
		LILRB2
		LILRB3
		LILRB4
		LIMCH1
		LIMK1
		LIMS2
		LIMS3L
		LINC00230A
		LINC00303
		LINC00312
		LINC00473
		LIPA
		LIPE
		LMCD1
		LMO3
		LMOD1
		LOC100127983
		LOC100129846

		LOC100130236
		LOC100130811
		LOC100130876
		LOC100131733
		LOC100170939
		LOC100270746
		LOC100288432
		LOC100288911
		LOC100292909
		LOC100505495
		LOC100505592
		LOC100505875
		LOC100505976
		LOC100505994
		LOC100506115
		LOC100506304
		LOC100506393
		LOC100506585
		LOC100506662
		LOC100506783
		LOC100506897
		LOC100507008
		LOC100507009
		LOC100507150
		LOC100507165
		LOC100507263
		LOC100507311
		LOC100507554
		LOC100508196
		LOC100509100
		LOC100509121
		LOC100509553
		LOC100652739
		LOC100652755
		LOC100652760
		LOC100652787
		LOC100652963
		LOC100652988
		LOC100653030
		LOC100653058
		LOC100653060
		LOC100653210

		LOC142937
		LOC145694
		LOC145820
		LOC254057
		LOC283050
		LOC283174
		LOC283454
		LOC286058
		LOC400043
		LOC400456
		LOC401093
		LOC401317
		LOC401847
		LOC441268
		LOC606724
		LOC643988
		LOC644189
		LOC644246
		LOC645638
		LOC645722
		LOC645954
		LOC648149
		LOC650226
		LOC654433
		LOC728052
		LOC728061
		LOC728868
		LOC729468
		LONRF2
		LOXL2
		LPAR1
		LPAR5
		LPHN3
		LPL
		LPP
		LPXN
		LRCH2
		LRIG3
		LRMP
		LRRC33
		LRRC55
		LRRC8D

		LRRN4CL
		LSP1
		LST1
		LTB
		LTF
		LUM
		LY6H
		LY86
		LY96
		LYN
		LYVE1
		MAF
		MAFB
		MAFF
		MAGED4B
		MAMDC2
		MAN2B1
		MANSC1
		MAOA
		MAOB
		MAP1B
		MAP4K1
		MAPK13
		MAPK4
		MAPKAPK3
		MARCKS
		MARCO
		MAT1A
		MATK
		MCAM
		MCOLN1
		MCOLN2
		ME2
		MEI1
		MEIS2
		MEOX2
		MET
		METTL7B
		MFAP4
		MFGE8
		MFNG
		MFSD1

		MFSD2A
		MFSD7
		MGAT4A
		MGAT4C
		MGP
		MGST1
		MILR1
		MIR143HG
		MLC1
		MMD
		MME
		MMP1
		MMP12
		MMP16
		MMP19
		MMP23B
		MMP7
		MMP9
		MNDA
		MOXD1
		MPEG1
		MPO
		MPP1
		MPP7
		MPZL2
		MRAP2
		MRC1
		MREG
		MRVI1
		MS4A14
		MS4A4A
		MS4A6A
		MS4A7
		MSR1
		MSRB3
		MSX1
		MT1A
		MT1B
		MT1E
		MT1F
		MT1G
		MT1L

		MT1M
		MT1X
		MT2A
		MTUS2
		MUSTN1
		MX1
		MXRA5
		MXRA7
		MYBL2
		MYBPC3
		MYBPH
		MYD88
		MYEF2
		MYH10
		MYH11
		MYL2
		MYL9
		MYLK
		MYLK2
		MYO18B
		MYO1F
		MYO1G
		MYO5A
		MYO7A
		MYOC
		MYOCD
		MYOM1
		MYOM2
		MYOZ1
		MYOZ2
		MYRIP
		MZB1
		NADKD1
		NAGK
		NAGPA
		NAIP
		NAP1L5
		NBLA00301
		NCEH1
		NCF1
		NCF2
		NCF4

		NCKAP1
		NCKAP1L
		NCRNA00185
		NCS1
		NDRG2
		NDUFA4L2
		NEDD9
		NET1
		NEURL2
		NEURL3
		NEXN
		NFAM1
		NFATC4
		NFE2
		NFIB
		NFIL3
		NFIX
		NFKBID
		NFKBIE
		NGFR
		NID1
		NIPAL4
		NKG7
		NLGN4X
		NLRP12
		NLRP3
		NOD2
		NOTCH3
		NOV
		NOX4
		NOXA1
		NPC1
		NPC2
		NPL
		NPNT
		NPR1
		NPR3
		NPTX2
		NPY1R
		NR1D1
		NR2F1
		NR2F2

		NRIP3
		NRK
		NRN1
		NSUN7
		NT5DC3
		NTF3
		NTN1
		NTRK3
		NUAK2
		NUSAP1
		NXF3
		NXPH3
		NXPH4
		OAS1
		OASL
		OBFC2A
		OGFRL1
		OGN
		OLFML3
		OLR1
		OMD
		OR10S1
		OR51E1
		OR51E2
		OSBPL3
		OSR1
		OSR2
		OXTR
		P2RX1
		P2RX4
		P2RX7
		P2RY13
		P2RY6
		P2RY8
		PACSIN1
		PACSIN3
		PAG1
		PALM
		PAQR5
		PARM1
		PARP12
		PART1

		PARVG
		PAWR
		PBX4
		PCDH10
		PCDH20
		PCDH7
		PCDHB9
		PCDHGB2
		PCOLCE
		PCOLCE2
		PCP4
		PCSK1
		PDE1C
		PDE4B
		PDE4D
		PDE5A
		PDE6G
		PDE8B
		PDGFB
		PDGFD
		PDGFRB
		PDK4
		PDLIM3
		PDPN
		PDZRN4
		PEBP4
		PEG10
		PER1
		PERP
		PF4
		PFKFB4
		PGAM2
		PGD
		PGF
		PGM5
		PGR
		PHGDH
		PHYHIP
		PI15
		PI16
		PI3
		PID1

		PIK3AP1
		PIK3CG
		PILRA
		PILRB
		PIM2
		PION
		PITX1
		PKD2
		PKD2L1
		PKIB
		PLA2G15
		PLA2G2A
		PLA2G7
		PLAT
		PLAU
		PLBD1
		PLCB2
		PLCB4
		PLCE1
		PLD3
		PLEK2
		PLEKHA6
		PLEKHG3
		PLEKHO2
		PLIN1
		PLIN2
		PLIN4
		PLN
		PLP1
		PLS3
		PLTP
		PLXNA1
		PLXNB1
		PLXNC1
		PLXND1
		PMAIP1
		PMF1-BGLAP
		PNMAL1
		PNMT
		PNOC
		PNPLA3
		PODN

		POLR2J2
		POSTN
		POTEF
		PPAP2A
		PPAPDC1A
		PPARG
		PPARGC1B
		PPBP
		PPIF
		PPL
		PPP1CB
		PPP1R12B
		PPP1R14A
		PPP1R3C
		PPP1R9A
		PPP2R2B
		PRDM1
		PRDM6
		PREX1
		PRICKLE1
		PROK2
		PRRT2
		PRRX1
		PRRX2
		PRSS35
		PRUNE2
		PSAP
		PSORS1C1
		PSPH
		PSPHP1
		PSTPIP1
		PTAFR
		PTCHD1
		PTCRA
		PTGER4
		PTGFR
		PTGIR
		PTGS1
		PTK2B
		PTN
		PTP4A3
		PTPLA

		PTPN21
		PTPN22
		PTPN6
		PTPN7
		PTPRC
		PTPRCAP
		PTPRD
		PTPRE
		PTPRO
		PTTG1
		PTX3
		PVRIG
		PVRL4
		PYCARD
		PYDC1
		PYGM
		Q6TXI9
		Q93YZ4
		RAB15
		RAB23
		RAB37
		RAB42
		RAB7B
		RAB9B
		RAC2
		RAI2
		RAMP1
		RAP2B
		RARRES1
		RASAL3
		RASD1
		RASGEF1B
		RASGRP1
		RASGRP3
		RASL10A
		RASL12
		RASSF3
		RASSF4
		RBFOX3
		RBM24
		RBM47
		RBP1

		RBP7
		RBPMS
		RBPMS2
		RCAN2
		RCSD1
		RDH5
		REEP1
		REEP4
		RELT
		RERG
		RERGL
		RETN
		RGL4
		RGMA
		RGN
		RGS1
		RGS10
		RGS18
		RGS19
		RGS5
		RGS7BP
		RHBDF2
		RHOB
		RHOH
		RHPN1
		RIMBP3
		RIMS4
		RIN3
		RMRP
		RN5-8S1
		RN7SL1
		RNASE1
		RNASE2
		RNASE3
		RNASE6
		RNASET2
		RNF112
		RNF125
		RNF135
		RNF152
		RNU105A
		RNU105C

		RNU2-2
		ROR1
		RORB
		RPS6KA1
		RRAGD
		RSAD2
		RSPO3
		RTN1
		RTN4R
		RTP4
		RUNX1T1
		RUNX3
		RYR2
		RYR3
		S100A12
		S100A8
		S100P
		S100Z
		S1PR3
		S1PR4
		SAA1
		SAA2
		SALL2
		SAMD14
		SAMD9L
		SAMSN1
		SAP25
		SASH3
		SAT1
		SBSN
		SCARA3
		SCARA5
		SCARB1
		SCARNA11
		SCARNA5
		SCD
		SCGB3A2
		SCHIP1
		SCIN
		SCN4B
		SCO2
		SCRG1

		SCXA
		SDIM1
		SDPR
		SDS
		SDSL
		SEC31B
		SECTM1
		SEL1L3
		SELENBP1
		SELL
		SELM
		SELP
		SELPLG
		SEMA3B
		SEMA3D
		SEPP1
		SERPINA1
		SERPINA3
		SERPINB9
		SERPINE1
		SERPINE2
		SEZ6L2
		SFN
		SFRP1
		SFRP4
		SGCA
		SGCD
		SGCE
		SGK1
		SGK223
		SH2B3
		SH3BGR
		SH3D19
		SHC4
		SHISA3
		SHOX2
		SHROOM3
		SIGLEC1
		SIGLEC15
		SIGLEC7
		SIGLEC9
		SIK1

		SIRPA
		SKAP1
		SLA
		SLAMF7
		SLAMF8
		SLC11A1
		SLC14A1
		SLC15A3
		SLC16A10
		SLC16A5
		SLC16A6
		SLC17A9
		SLC19A2
		SLC1A3
		SLC22A18
		SLC22A18AS
		SLC22A3
		SLC24A3
		SLC25A19
		SLC25A4
		SLC26A10
		SLC28A3
		SLC29A3
		SLC2A6
		SLC2A9
		SLC30A3
		SLC31A2
		SLC36A1
		SLC37A2
		SLC38A7
		SLC39A11
		SLC39A14
		SLC40A1
		SLC43A2
		SLC47A1
		SLC6A12
		SLC6A16
		SLC7A11
		SLC7A2
		SLC7A7
		SLCO2B1
		SLCO4A1

		SLIT3
		SLITRK4
		SLMAP
		SLN
		SLPI
		SMAD6
		SMAD9
		SMOC1
		SMOC2
		SMPD3
		SMPDL3A
		SMPX
		SMTN
		SNAI3
		SNAP25
		SNAR-A3
		SNAR-B2
		SNAR-D
		SNAR-F
		SNAR-G2
		SNAR-H
		SNCA
		SNCG
		SNHG5
		SNORA11
		SNORA11C
		SNORA15
		SNORA19
		SNORA21
		SNORA27
		SNORA28
		SNORA34
		SNORA42
		SNORA45
		SNORA54
		SNORA57
		SNORA81
		SNORD105B
		SNORD107
		SNORD114-15
		SNORD114-21
		SNORD114-23

		SNORD114-3
		SNORD114-31
		SNORD114-9
		SNORD116-19
		SNORD116-4
		SNORD116-6
		SNORD15A
		SNORD15B
		SNORD16
		SNORD17
		SNORD46
		SNORD66
		SNORD71
		SNORD89
		SNORD97
		SNTA1
		SNX10
		SOAT1
		SOCS2
		SOD2
		SORBS1
		SORBS2
		SORL1
		SOST
		SOX8
		SP5
		SPARCL1
		SPI1
		SPINK1
		SPINK13
		SPINK2
		SPINT1
		SPINT2
		SPOCD1
		SPON1
		SPP1
		SRGN
		SRL
		SRMS
		SRPK3
		SRPX
		SRRM4

		SSTR1
		ST14
		ST6GAL2
		STAB1
		STAC2
		STAC3
		STEAP1
		STEAP1B
		STEAP3
		STK10
		STK38L
		STMN2
		STRA6
		STXBP2
		SULT1C2
		SUSD1
		SUSD2
		SUSD5
		SVEP1
		SYBU
		SYK
		SYNC
		SYNDIG1
		SYNM
		SYNPO2
		SYTL2
		TACC2
		TACC3
		TACSTD2
		TAGAP
		TAGLN
		TBC1D10C
		TBC1D2
		TBX2
		TBX21
		TC2N
		TCAP
		TCEAL2
		TCIRG1
		TCN2
		TDO2
		TEAD3

		TES
		TFCP2L1
		TFEC
		TFPI2
		TFRC
		TGFB1I1
		TGFB3
		TGFBI
		TGFBR3
		TGM2
		THBS1
		THBS2
		THBS4
		THRA
		THY1
		TIAM1
		TIFAB
		TIMD4
		TIMP1
		TIMP3
		TIMP4
		TK1
		TLR1
		TLR2
		TLR7
		TM4SF19
		TM6SF1
		TM7SF4
		TMC6
		TMEM100
		TMEM114
		TMEM130
		TMEM132A
		TMEM158
		TMEM176A
		TMEM176B
		TMEM181
		TMEM200A
		TMEM206
		TMEM30B
		TMEM35
		TMEM47

		TMEM51
		TMEM61
		TMEM86A
		TMEM88
		TMOD1
		TMSB15A
		TMSB15B
		TNF
		TNFAIP2
		TNFAIP3
		TNFAIP8L2
		TNFRSF10C
		TNFRSF11B
		TNFRSF12A
		TNFRSF21
		TNFRSF25
		TNFRSF9
		TNFSF13
		TNFSF13B
		TNFSF14
		TNFSF9
		TNNI2
		TNNT1
		TNNT3
		TNS1
		TPM1
		TPM2
		TPPP
		TPSAB1
		TPST2
		TRAF3IP3
		TRDN
		TREM1
		TREM2
		TRGV7
		TRIB3
		TRIM14
		TRIM25
		TRIM54
		TRIM58
		TRIM63
		TRPM2

		TRPV2
		TSC22D1
		TSC22D3
		TSKU
		TSPAN10
		TSPAN12
		TSPAN15
		TSPAN2
		TSPAN33
		TSPAN7
		TSPAN8
		TTLL7
		TTYH3
		TUBB3
		TUSC3
		TXNDC3
		TXNIP
		TYMS
		TYROBP
		UAP1
		UAP1L1
		UBD
		UBE2T
		UCP2
		UNC13C
		UNC13D
		UNC5B
		UNC93B1
		UPP1
		USP53
		UTS2
		VAMP8
		VASN
		VAT1L
		VAV1
		VAV3
		VCAM1
		VCL
		VENTX
		VGLL3
		VIPR2
		VIT

		VMO1
		VNN1
		VNN2
		VSIG4
		VSTM1
		WAS
		WBSCR17
		WDR86
		WEE1
		WFDC1
		WFDC2
		WIF1
		WISP2
		WNK3
		WNT11
		WNT5A
		WTIP
		XAF1
		XK
		XLOC_000346
		XLOC_000578
		XLOC_000587
		XLOC_000845
		XLOC_000978
		XLOC_001215
		XLOC_001228
		XLOC_001678
		XLOC_001788
		XLOC_001855
		XLOC_001947
		XLOC_002133
		XLOC_002231
		XLOC_002623
		XLOC_003177
		XLOC_003181
		XLOC_003228
		XLOC_003776
		XLOC_003799
		XLOC_003919
		XLOC_004049
		XLOC_004351
		XLOC_004555

		XLOC_004598
		XLOC_004924
		XLOC_005062
		XLOC_005489
		XLOC_005566
		XLOC_005815
		XLOC_005936
		XLOC_006025
		XLOC_006751
		XLOC_007433
		XLOC_008000
		XLOC_008251
		XLOC_008378
		XLOC_008644
		XLOC_008700
		XLOC_009167
		XLOC_009549
		XLOC_009685
		XLOC_009932
		XLOC_009994
		XLOC_010385
		XLOC_010931
		XLOC_010933
		XLOC_011331
		XLOC_011344
		XLOC_011608
		XLOC_011815
		XLOC_012139
		XLOC_012252
		XLOC_013436
		XLOC_013929
		XLOC_013983
		XLOC_014002
		XLOC_014161
		XLOC_l2_000342
		XLOC_l2_002659
		XLOC_l2_002790
		XLOC_l2_004840
		XLOC_l2_005804
		XLOC_l2_006821
		XLOC_l2_007424
		XLOC_l2_007731

		XLOC_l2_008783
		XLOC_l2_008976
		XLOC_l2_009273
		XLOC_l2_010064
		XLOC_l2_010897
		XLOC_l2_011118
		XLOC_l2_012953
		XLOC_l2_013193
		XLOC_l2_013301
		XLOC_l2_013636
		XLOC_l2_014602
		XLOC_l2_015738
		XLOC_l2_015938
		YAP1
		ZBTB16
		ZEB1
		ZFHX4
		ZFP57
		ZNF385A
		ZNF469
		ZNF683
		ZWINT

Supplementary Table S7

Overlap of 109 genes in the strongest positive and negative modules of OSA and AS

ABCC3
ADAMTS1
ASPN
BCHE
C7
CADM3
CCDC3
CCL13
CCL18
CCL2
CCL5
CD24
CD69
CHI3L2
COL14A1
COL1A1
COL8A1
CPA3

CR1
CSF3R
CXCL10
CXCL14
CXCL9
CXCR2
CYP1B1
DDX3Y
DPP4
DPT
EGFL6
ERRFI1
FAM96A
FMO3
FNDC1
FRZB
GFPT2
GLDN
GPR183
GPR34
GREM1
GSTM5
GZMK
HBA2
HBB
HLA-DQA1
HLA-DQB1
HLA-DRB1
HLA-DRB5
HP
HPR
HSD11B1
HSD17B6
HSPB7
ID4
IL7R
IRF8
ITLN1
JUNB
KRT18P55
LBP
LYVE1

MAMDC2
MYOC
NFIL3
NPR3
NPY1R
OGN
OSR2
PDE8B
PDGFD
PDLIM3
PEG10
PERP
PF4
PHGDH
PI16
PLA2G2A
PNPLA3
PPBP
PTPN22
RASGEF1B
RGS1
RGS18
RGS7BP
S100A8
SCIN
SELL
SERPINE1
SFRP4
SLC28A3
SLPI
SMOC2
SNORA28
SNX10
STMN2
TC2N
TFPI2
TNFRSF11B
TRDN
VCAM1
ANXA3
AOX1
CASQ2

CP
IFI44L
LTF
MXRA5
SAA1
SAA2
VNN2

Supplementary Table S8

Enrichment analysis results of KEGG for 109 genes												
Category	*Term*	*Count*	*%*	*PValue*	*Genes*	*List Total*	*Pop Hits*	*Pop Total*	*Fold Enrichment*	*Bonferroni*	*Benjamini*	*FDR*
KEGG_PATHWAY	*hsa04061:Viral protein interaction with cytokine and cytokine receptor*	*10*	*9.174311927*	*3.64E-08*	*ENSG00000145824, ENSG00000180871, ENSG00000163737, ENSG00000169245, ENSG00000181374, ENSG00000138755, ENSG00000271503, ENSG00000108691, ENSG00000163736, ENSG00000275385*	*61*	*100*	*8205*	*13.45081967*	*5.28E-06*	*5.28E-06*	*4.59E-06*
KEGG_PATHWAY	*hsa04060:Cytokine-cytokine receptor interaction*	*13*	*11.926605 5*	*1.11E-06*	*ENSG00000180871, ENSG00000163737, ENSG00000181374, ENSG00000138755, ENSG00000119535, ENSG00000145824, ENSG00000164761, ENSG00000169245, ENSG00000168685, ENSG00000271503, ENSG00000108691, ENSG00000163736, ENSG00000275385*	*61*	*295*	*8205*	*5.927479856*	*1.61E-04*	*8.06E-05*	*7.00E-05*
KEGG_PATHWAY	*hsa04640:Hematopoietic cell lineage*	*8*	*7.339449541*	*6.88E-06*	*ENSG00000203710, ENSG00000119535, ENSG00000168685, ENSG00000179344, ENSG00000196126, ENSG00000198502, ENSG00000272398, ENSG00000196735*	*61*	*99*	*8205*	*10.86934923*	*9.98E-04*	*3.33E-04*	*2.89E-04*

KEGG_PATHWAY	*hsa04062:Chemokine signaling pathway*	*10*	*9.174311927*	*9.20E-06*	*ENSG00000145824, ENSG00000180871, ENSG00000163737, ENSG00000169245, ENSG00000181374, ENSG00000138755, ENSG00000271503, ENSG00000108691, ENSG00000163736, ENSG00000275385*	*61*	*192*	*8205*	*7.005635246*	*0.001332646*	*3.33E-04*	*2.90E-04*
KEGG_PATHWAY	*hsa05144:Malaria*	*5*	*4.587155963*	*4.63E-04*	*ENSG00000203710, ENSG00000162692, ENSG00000244734, ENSG00000188536, ENSG00000108691*	*61*	*50*	*8205*	*13.45081967*	*0.064933845*	*0.013424491*	*0.011665422*
KEGG_PATHWAY	*hsa05323:Rheumatoid arthritis*	*6*	*5.504587156*	*5.61E-04*	*ENSG00000179344, ENSG00000271503, ENSG00000196126, ENSG00000108691, ENSG00000198502, ENSG00000196735*	*61*	*93*	*8205*	*8.677948176*	*0.078112407*	*0.013551529*	*0.011775811*
KEGG_PATHWAY	*hsa04514:Cell adhesion molecules*	*7*	*6.422018349*	*9.52E-04*	*ENSG00000162692, ENSG00000179344, ENSG00000162706, ENSG00000188404, ENSG00000196126, ENSG00000198502, ENSG00000196735*	*61*	*157*	*8205*	*5.997180746*	*0.12897614*	*0.019717168*	*0.017133539*
KEGG_PATHWAY	*hsa05310:Asthma*	*4*	*3.669724771*	*0.001444635*	*ENSG00000179344, ENSG00000196126, ENSG00000198502, ENSG00000196735*	*61*	*31*	*8205*	*17.35589635*	*0.189110542*	*0.023849229*	*0.020724158*
KEGG_PATHWAY	*hsa05164:Influenza A*	*7*	*6.422018349*	*0.001480297*	*ENSG00000169245, ENSG00000179344, ENSG00000271503, ENSG00000196126, ENSG00000108691, ENSG00000198502, ENSG00000196735*	*61*	*171*	*8205*	*5.506183492*	*0.193298918*	*0.023849229*	*0.020724158*
KEGG_PATHWAY	*hsa05140:Leishmaniasis*	*5*	*4.587155963*	*0.00234902*	*ENSG00000203710, ENSG00000179344, ENSG00000196126, ENSG00000198502,*	*61*	*77*	*8205*	*8.734298488*	*0.288947223*	*0.031593204*	*0.02745 34*

					ENSG00000196735							05
KEGG_PATHWAY	hsa05143:African trypanosomiasis	4	3.669724771	0.002420698	ENSG00000162692, ENSG00000244734, ENSG00000261701, ENSG00000188536	61	37	8205	14.54142667	0.296316634	0.031593204	0.027453405
KEGG_PATHWAY	hsa05330:Allograft rejection	4	3.669724771	0.00261461	ENSG00000179344, ENSG00000196126, ENSG00000198502, ENSG00000196735	61	38	8205	14.15875755	0.31587521	0.031593204	0.027453405
KEGG_PATHWAY	hsa05332:Graft-versus-host disease	4	3.669724771	0.003485147	ENSG00000179344, ENSG00000196126, ENSG00000198502, ENSG00000196735	61	42	8205	12.81030445	0.397235683	0.038603203	0.033548853
KEGG_PATHWAY	hsa04940:Type I diabetes mellitus	4	3.669724771	0.003727206	ENSG00000179344, ENSG00000196126, ENSG00000198502, ENSG00000196735	61	43	8205	12.51239039	0.418098757	0.038603203	0.033548853
KEGG_PATHWAY	hsa04672:Intestinal immune network for IgA production	4	3.669724771	0.005394519	ENSG00000179344, ENSG00000196126, ENSG00000198502, ENSG00000196735	61	49	8205	10.98026096	0.543571276	0.05214702	0.045313962
KEGG_PATHWAY	hsa04974:Protein digestion and absorption	5	4.587155963	0.006667411	ENSG00000144810, ENSG00000108821, ENSG00000197635, ENSG00000163751, ENSG00000187955	61	103	8205	6.529524113	0.62092146	0.057308959	0.049799509
KEGG_PATHWAY	hsa05320:Autoimmune thyroid disease	4	3.669724771	0.006718981	ENSG00000179344, ENSG00000196126, ENSG00000198502, ENSG00000196735	61	53	8205	10.15156202	0.623764507	0.057308959	0.049799509
KEGG_PATHWAY	hsa04668:TNF signaling pathway	5	4.587155963	0.008922223	ENSG00000162692, ENSG00000169245, ENSG00000271503, ENSG00000108691, ENSG00000171223	61	112	8205	6.004830211	0.727339636	0.07185778	0.062441933
KEGG_PATHWAY	hsa05416:Viral myocarditis	4	3.669724771	0.009467246	ENSG00000179344, ENSG00000196126, ENSG00000198502,	61	60	8205	8.967213115	0.748242788	0.07185778	0.06244

					ENSG00000196735							1933
KEGG_PATHWAY	hsa05152:Tuberculosis	6	5.504587156	0.009911418	ENSG00000203710, ENSG00000179344, ENSG00000196126, ENSG00000129988, ENSG00000198502, ENSG00000196735	61	180	8205	4.483606557	0.764094773	0.07185778	0.062441933
KEGG_PATHWAY	hsa05321:Inflammatory bowel disease	4	3.669724771	0.011777653	ENSG00000179344, ENSG00000196126, ENSG00000198502, ENSG00000196735	61	65	8205	8.277427491	0.820556714	0.081321891	0.070665919
KEGG_PATHWAY	hsa05322:Systemic lupus erythematosus	5	4.587155963	0.017213767	ENSG00000112936, ENSG00000179344, ENSG00000196126, ENSG00000198502, ENSG00000196735	61	136	8205	4.945154291	0.91935744	0.113454374	0.098587939
KEGG_PATHWAY	hsa04612:Antigen processing and presentation	4	3.669724771	0.019189555	ENSG00000179344, ENSG00000196126, ENSG00000198502, ENSG00000196735	61	78	8205	6.897856242	0.939766664	0.120977629	0.105125388
KEGG_PATHWAY	hsa04658:Th1 and Th2 cell differentiation	4	3.669724771	0.029490204	ENSG00000179344, ENSG00000196126, ENSG00000198502, ENSG00000196735	61	92	8205	5.848182466	0.986968665	0.178169981	0.154823569
KEGG_PATHWAY	hsa05150:Staphylococcus aureus infection	4	3.669724771	0.032876007	ENSG00000179344, ENSG00000196126, ENSG00000198502, ENSG00000196735	61	96	8205	5.604508197	0.992149219	0.190680842	0.165695077
KEGG_PATHWAY	hsa04933:AGE-RAGE signaling pathway in diabetic complications	4	3.669724771	0.036456844	ENSG00000162692, ENSG00000108821, ENSG00000108691, ENSG00000106366	61	100	8205	5.380327869	0.995415206	0.203317014	0.176675474
KEGG_PATHWAY	hsa04620:Toll-like receptor signaling pathway	4	3.669724771	0.040231028	ENSG00000169245, ENSG00000138755, ENSG00000271503, ENSG00000129988	61	104	8205	5.173392182	0.997404791	0.216055521	0.187744798
KEGG_	hsa04659:Th17 cell	4	3.669	0.04419	ENSG00000179344,	61	108	8205	4.981785064	0.9985	0.228	0.1

PATHWAY	differentiation		724771	6477	ENSG00000196126, ENSG00000198502, ENSG00000196735					76194	874613	98884146
KEGG_PATHWAY	hsa05145:Toxoplasmosis	4	3.669724771	0.048350742	ENSG00000179344, ENSG00000196126, ENSG00000198502, ENSG00000196735	61	112	8205	4.803864169	0.999242901	0.24175371	0.210075638
KEGG_PATHWAY	hsa05169:Epstein-Barr virus infection	5	4.587155963	0.059787534	ENSG00000169245, ENSG00000179344, ENSG00000196126, ENSG00000198502, ENSG00000196735	61	202	8205	3.32941081	0.999868849	0.288973081	0.251107643
KEGG_PATHWAY	hsa00140:Steroid hormone biosynthesis	3	2.75229357 8	0.075222922	ENSG00000117594, ENSG00000138061, ENSG00000025423	61	62	8205	6.508461132	0.999988105	0.351849152	0.305744478
KEGG_PATHWAY	hsa05204:Chemical carcinogenesis - DNA adducts	3	2.752293578	0.09038557	ENSG00000117594, ENSG00000138061, ENSG00000134201	61	69	8205	5.848182466	0.999998918	0.409559614	0.355893182
KEGG_PATHWAY	hsa00982:Drug metabolism - cytochrome P450	3	2.752293578	0.097139781	ENSG00000007933, ENSG00000138356, ENSG00000134201	61	72	8205	5.604508197	0.999999633	0.424487845	0.368868653
KEGG_PATHWAY	hsa04145:Phagosome	4	3.669724771	0.099535081	ENSG00000179344, ENSG00000196126, ENSG00000198502, ENSG00000196735	61	152	8205	3.539689387	0.99999975	0.424487845	0.368868653

Supplementary Table S9

Enrichment analysis results of GO for 109 genes									
ONTOLOGY	ID	Description	GeneRatio	BgRatio	pvalue	p.adjust	qvalue	geneID	Count
BP	GO:0060425	lung morphogenesis	4/57	50/18800	1.58E-05	0.020047912	0.015707026	GRHL2/LIF/PLOD3/SHH	4
BP	GO:0042060	wound healing	8/57	429/18800	4.27E-05	0.020047912	0.015707026	CDH3/CLDN1/F5/FOXA2/OCLN/PLAU/SERPINA1/S	8

								HH	
BP	*GO:1905330*	*regulation of morphogenesis of an epithelium*	*4/57*	*65/18800*	*4.48E-05*	*0.020047912*	*0.015707026*	*HOXB7/LIF/MDK/SHH*	*4*
BP	*GO:0035270*	*endocrine system development*	*5/57*	*132/18800*	*4.94E-05*	*0.020047912*	*0.015707026*	*CITED2/FOXA2/LY6E/MDK/SHH*	*5*
BP	*GO:0030325*	*adrenal gland development*	*3/57*	*27/18800*	*7.34E-05*	*0.02382541*	*0.018666599*	*CITED2/LY6E/MDK*	*3*
BP	*GO:1903034*	*regulation of response to wounding*	*5/57*	*166/18800*	*0.000146013*	*0.03949662*	*0.030944591*	*CLDN1/FOXA2/MDK/OCLN/PLAU*	*5*
BP	*GO:1905332*	*positive regulation of morphogenesis of an epithelium*	*3/57*	*36/18800*	*0.000175735*	*0.040745374*	*0.031922957*	*HOXB7/LIF/MDK*	*3*
BP	*GO:0045687*	*positive regulation of glial cell differentiation*	*3/57*	*42/18800*	*0.000278934*	*0.056588761*	*0.044335846*	*LIF/MDK/SHH*	*3*
BP	*GO:0009612*	*response to mechanical stimulus*	*5/57*	*201/18800*	*0.00035443*	*0.060426202*	*0.047342382*	*BDKRB1/BTG2/CITED2/MDK/SLITRK6*	*5*
BP	*GO:0050679*	*positive regulation of epithelial cell proliferation*	*5/57*	*211/18800*	*0.000442616*	*0.060426202*	*0.047342382*	*CDH3/CLDN1/MDK/SCG2/SHH*	*5*
CC	*GO:0005788*	*endoplasmic reticulum lumen*	*7/59*	*311/19594*	*3.99E-05*	*0.002207936*	*0.002041771*	*ADAMTSL4/F5/PLOD3/SCG2/SERPINA1/SHH/TSPAN5*	*7*
CC	*GO:0062023*	*collagen-containing extracellular matrix*	*8/59*	*429/19594*	*4.13E-05*	*0.002207936*	*0.002041771*	*ADAMTSL4/COCH/EGFL6/MDK/PLOD3/SERPINA1/SHH/TIMP3*	*8*
CC	*GO:0005911*	*cell-cell junction*	*6/59*	*497/19594*	*0.00373503*	*0.133216085*	*0.123190477*	*BAIAP2L1/CDH3/CLDN1/DSG2/GRHL2/OCLN*	*6*
CC	*GO:0031093*	*platelet alpha granule lumen*	*2/59*	*67/19594*	*0.017386153*	*0.311370884*	*0.28793766*	*F5/SERPINA1*	*2*
CC	*GO:0033116*	*endoplasmic reticulum-Golgi intermediate compartment membrane*	*2/59*	*77/19594*	*0.022572529*	*0.311370884*	*0.28793766*	*F5/SERPINA1*	*2*
CC	*GO:0031091*	*platelet alpha granule*	*2/59*	*91/19594*	*0.030757984*	*0.311370884*	*0.28793766*	*F5/SERPINA1*	*2*
CC	*GO:0005614*	*interstitial matrix*	*1/59*	*11/19594*	*0.032636388*	*0.311370884*	*0.28793766*	*ADAMTSL4*	*1*
CC	*GO:1904090*	*peptidase inhibitor complex*	*1/59*	*11/19594*	*0.032636388*	*0.311370884*	*0.28793766*	*PLAU*	*1*
CC	*GO:1905286*	*serine-type peptidase complex*	*1/59*	*11/19594*	*0.032636388*	*0.311370884*	*0.28793766*	*PLAU*	*1*
CC	*GO:0030134*	*COPII-coated ER to Golgi transport vesicle*	*2/59*	*94/19594*	*0.032643994*	*0.311370884*	*0.28793766*	*F5/SERPINA1*	*2*
MF	*GO:0048018*	*receptor ligand activity*	*8/56*	*489/18410*	*0.000108444*	*0.008912285*	*0.006673982*	*BTC/CTF1/CXCL6/IL32/LIF/MDK/SCG2/SHH*	*8*
MF	*GO:0030546*	*signaling receptor activator activity*	*8/56*	*496/18410*	*0.000119628*	*0.008912285*	*0.006673982*	*BTC/CTF1/CXCL6/IL32/LIF/MDK/SCG2/SHH*	*8*

MF	*GO:00 05125*	*cytokine activity*	*5/56*	*235/1 8410*	*0.00073 0185*	*0.03626 5842*	*0.02715 7748*	*CTF1/CXCL6/IL32/LIF/SC G2*	*5*
MF	*GO:00 05229*	*intracellular calcium activated chloride channel activity*	*2/56*	*18/18 410*	*0.00134 7648*	*0.04015 9902*	*0.03007 3823*	*ANO5/TTYH3*	*2*
MF	*GO:00 61778*	*intracellular chloride channel activity*	*2/56*	*18/18 410*	*0.00134 7648*	*0.04015 9902*	*0.03007 3823*	*ANO5/TTYH3*	*2*
MF	*GO:00 04602*	*glutathione peroxidase activity*	*2/56*	*22/18 410*	*0.00201 8871*	*0.05013 5287*	*0.03754 391*	*GPX2/GSTM2*	*2*
MF	*GO:00 22839*	*ion gated channel activity*	*2/56*	*44/18 410*	*0.00792 1397*	*0.15302 8829*	*0.11459 5944*	*ANO5/TTYH3*	*2*
MF	*GO:00 02020*	*protease binding*	*3/56*	*136/1 8410*	*0.00821 6313*	*0.15302 8829*	*0.11459 5944*	*ADAMTSL4/SERPINA1/TI MP3*	*3*
MF	*GO:00 04601*	*peroxidase activity*	*2/56*	*52/18 410*	*0.01093 2439*	*0.16373 3866*	*0.12261 2433*	*GPX2/GSTM2*	*2*
MF	*GO:00 98632*	*cell-cell adhesion mediator activity*	*2/56*	*54/18 410*	*0.01175 2511*	*0.16373 3866*	*0.12261 2433*	*BAIAP2L1/DSG2*	*2*

Supplementary Table S10

34 key genes are overlapping between the genes screened by WGCNA and those screened by DEGs

ADAMTS1
CCL13
CCL18
CXCL10
CXCL9
DPP4
EGFL6
ERRFI1
FAM96A
FNDC1
GFPT2
HBA2
HBB
HLA-DQA1
HLA-DRB1
HSD11B1
ID4
JUNB
KRT18P55
LYVE1
NPR3
NPY1R
OGN
PDLIM3

PERP
PF4
PI16
PPBP
PTPN22
RASGEF1B
RGS18
SLC28A3
SLPI
SNX10

Supplementary Table S11

Narrowing down biomarkers using the LASSO regression model

LASSO_ GSE100927	*LASSO_ GSE135917*
CCL13	*CCL18*
DPP4	*CXCL10*
ERRFI1	*EGFL6*
FNDC1	*ERRFI1*
HBA2	*FNDC1*
HLA-DRB1	*HLA-DRB1*
HSD11B1	*HSD11B1*
NPR3	*OGN*
NPY1R	*PERP*
PERP	*PTPN22*
PI16	*SNX10*
RGS18	
SLPI	

Supplementary Table S12

The five DEGs (ERRFI1, FNDC1, HLA-DRB1, HSD11B1, PERP) in GSE135917, GSE38792, GSE100927, and GSE43292

GSE100927						
id	*logFC*	*AveExpr*	*t*	*P.Value*	*adj.P.Val*	*B*
ERRFI1	*-0.715371255*	*8.654875569*	*-7.316599325*	*5.33E-11*	*6.51E-10*	*14.581396*
FNDC1	*1.126381805*	*6.544819692*	*8.643518582*	*6.71E-14*	*1.56E-12*	*21.14531418*
HLA-DRB1	*1.45842232*	*10.11963909*	*10.6285076*	*2.42E-18*	*1.82E-16*	*31.22630701*
HSD11B1	*-0.85636489*	*6.248394819*	*-5.51144266*	*2.57E-07*	*1.52E-06*	*6.301257939*
PERP	*-0.872719061*	*6.964750912*	*-10.46416671*	*5.64E-18*	*3.74E-16*	*30.38999769*
GSE135917						
id	*logFC*	*AveExpr*	*t*	*P.Value*	*adj.P.Val*	*B*

ERRFI1	*-0.649599926*	*8.349925094*	*-3.115271359*	*0.003169201*	*0.020629553*	*-1.917557508*
FNDC1	*-0.778746615*	*7.023630068*	*-4.211060218*	*0.000117715*	*0.003050791*	*1.087167017*
HLA-DRB1	*0.907732309*	*7.527531125*	*2.683929737*	*0.010095534*	*0.044478648*	*-2.947750137*
HSD11B1	*0.781297576*	*7.766906399*	*3.956001478*	*0.000262845*	*0.004716337*	*0.347569115*
PERP	*0.587524233*	*8.540007785*	*2.950809494*	*0.004983047*	*0.028010722*	*-2.322487719*
GSE38792						
id	*logFC*	*AveExpr*	*t*	*P.Value*	*adj.P.Val*	*B*
ERRFI1	*-0.504470501*	*8.841316978*	*-2.250278246*	*0.035255514*	*0.034193906*	*-3.888608016*
FNDC1	*-0.025033821*	*7.002211037*	*-0.109890953*	*0.913537308*	*0.986650156*	*-4.890473462*
HLA-DRB1	*0.694118469*	*7.308334719*	*2.322289197*	*0.030329189*	*0.634193906*	*-3.834026566*
HSD11B1	*0.267250419*	*7.248966025*	*1.082261286*	*0.291385058*	*0.780648409*	*-4.629127822*
PERP	*0.090572717*	*8.065121398*	*0.662840453*	*0.514629549*	*0.873017674*	*-4.791589449*
GSE43292						
ERRFI1	*-0.603044918*	*9.47457683*	*-2.452104648*	*0.016880168*	*0.04027081*	*-3.520134618*
FNDC1	*0.166939712*	*7.71842864*	*1.624558018*	*0.109066805*	*0.228382188*	*-5.075576788*
HLA-DRB1	*0.490255281*	*8.380554899*	*1.863213574*	*0.066923239*	*0.1556053*	*-4.684525674*
HSD11B1	*-0.027278323*	*5.834374363*	*-0.674242312*	*0.502533523*	*0.679224725*	*-6.125829832*
PERP	*-0.48791868*	*7.679610978*	*-5.811885921*	*2.00E-07*	*2.13E-05*	*6.965517963*
